# Transcriptome analysis of phosphorus stress responsiveness in the seedlings of Dongxiang wild rice (*Oryza rufipogon* Griff.)

**DOI:** 10.1186/s40659-018-0155-x

**Published:** 2018-03-15

**Authors:** Qian-Wen Deng, Xiang-Dong Luo, Ya-Ling Chen, Yi Zhou, Fan-Tao Zhang, Biao-Lin Hu, Jian-Kun Xie

**Affiliations:** 1 College of Life Science, Jiangxi Normal University, Nanchang, 330022 China; 2 Rice Research Institute, Jiangxi Academy of Agricultural Science, Nanchang, 330200 China

**Keywords:** Dongxiang wild rice, Genetic resource, Response to phosphorus deficiency, Transcriptome analysis, RNA-sequencing

## Abstract

**Background:**

Low phosphorus availability is a major factor restricting rice growth. Dongxiang wild rice (*Oryza rufipogon* Griff.) has many useful genes lacking in cultivated rice, including stress resistance to phosphorus deficiency, cold, salt and drought, which is considered to be a precious germplasm resource for rice breeding. However, the molecular mechanism of regulation of phosphorus deficiency tolerance is not clear.

**Results:**

In this study, cDNA libraries were constructed from the leaf and root tissues of phosphorus stressed and untreated Dongxiang wild rice seedlings, and transcriptome sequencing was performed with the goal of elucidating the molecular mechanisms involved in phosphorus stress response. The results indicated that 1184 transcripts were differentially expressed in the leaves (323 up-regulated and 861 down-regulated) and 986 transcripts were differentially expressed in the roots (756 up-regulated and 230 down-regulated). 43 genes were up-regulated both in leaves and roots, 38 genes were up-regulated in roots but down-regulated in leaves, and only 2 genes were down-regulated in roots but up-regulated in leaves. Among these differentially expressed genes, the detection of many transcription factors and functional genes demonstrated that multiple regulatory pathways were involved in phosphorus deficiency tolerance. Meanwhile, the differentially expressed genes were also annotated with gene ontology terms and key pathways via functional classification and Kyoto Encyclopedia of Gene and Genomes pathway mapping, respectively. A set of the most important candidate genes was then identified by combining the differentially expressed genes found in the present study with previously identified phosphorus deficiency tolerance quantitative trait loci.

**Conclusion:**

The present work provides abundant genomic information for functional dissection of the phosphorus deficiency resistance of Dongxiang wild rice, which will be help to understand the biological regulatory mechanisms of phosphorus deficiency tolerance in Dongxiang wild rice.

**Electronic supplementary material:**

The online version of this article (10.1186/s40659-018-0155-x) contains supplementary material, which is available to authorized users.

## Background

Phosphorus (P) is an essential mineral element required for plant growth and development. It plays a crucial role in the processes of energy transfer, signal transduction, photosynthesis and respiration [1]. Rice is one of the most important food crops in the world, and it is also considered as a model organism for monocotyledon genomics research [2]. Although the total amount of P in the soil may be high, it is often present in unavailable forms or in forms that are only available outside the rhizosphere [3]. Thus, the deficiency of phosphorus in soil is a worldwide problem. According to the statistics, about 5.8 billion hm^2^ of arable land worldwide is deficient in P. And about 67 million hm^2^ of arable land in China is deficient in P, which resulted in yield reduction by 5–15% (about 25–75 billion Kg) [4]. Over the past several decades, P fertilizer application for crop growth has been increased rapidly, but P-use efficiency has decreased to a low level of 10–20% [5]. P fertilizer consumes non-renewable phosphate rock reserves, which are expected to be exhausted in the near future [6]. Furthermore, much of the applied P has caused serious environmental pollutions [7]. Therefore, it is a significant development direction to improve the status of P-deficiency in crops to explore the absorption and utilization of high P-use efficiency of crops.

Previous studies have found that there are significant genotypic differences in P-deficiency tolerance among different genotypes [8] which make it possible to breed varieties with improved P-deficiency tolerance. During the course of domestication from wild rice to cultivated rice, however, the number of alleles of cultivated rice reduced by 50–60% compared to wild rice [9]. Moreover, the wide application of commercial hybrids in recent decades has caused the loss of many excellent local varieties, and the genetic diversity of cultivated rice has become more and more narrow.

Dongxiang wild rice (*O. rufipogon* Griff., Hereinafter referred to as DXWR), a Chinese type of wild rice grown in Dongxiang County, Jiangxi Province (28°14′ N latitude and 116°30′ E longitude), is considered to be the northernmost region in the world where *O. rufipogon* is found, which is one of unique wild resources in Jiangxi province and one of the second class national protected wild plants in China [10]. Previous studies confirmed that DXWR has many useful genes lacking in cultivated rice, including P-deficiency tolerance [11], strong cold tolerance [12], high grain yield [13] and drought resistance [14]. Our previous results and relative reports indicated that DXWR was more resistant to low-P stress than low-P tolerant cultivated rice ‘Dalidao’ and ‘Liantangzao3’, suggesting that DXWR has strong resistance to low-phosphorus stress [15]. Therefore, it is considered to be a valuable resource for the exploitation and utilization of P-deficiency tolerance genes in rice. So far, there has been a great deal of quantitative trait loci (QTLs) for abiotic stress tolerance in DXWR, including P-deficiency tolerance [14, 16–20]. However, the regulatory mechanisms of P-deficiency tolerance have not been fully understood. Thus, a better understanding of P-deficiency tolerance mechanisms would be helpful for breeding P-deficiency tolerance rice cultivars.

In this study, the transcriptome of DXWR under P-deficiency stress was obtained by experiment. Then some candidate genes were found by combining the DEGs interval of this study with the previously identified QTLs interval associated with P-deficiency tolerance. The results will provide a basis for explaining the molecular mechanism of resistance, as well as cloning and utilizing the P-deficiency genes from wild rice.

## Methods

### Material and treatment

Dongxiang wild rice (*O. rufipogon* Griff., hereafter referred to as DXWR) was used as test material. Disinfection of rice seeds with 10% sodium hypochlorite, then soak the seeds with water at room temperature for 30 h. Selection of the uniformly germinated seeds of DXWR were grown in a plastic pot in a plant growth chamber at day/night temperature of 30 °C/26 °C (14 h day/10 h night) with relative humidity of 70% and 3000 lx of light intensity. Germinated seeds with coleoptiles 8–12 mm in length, adding Yoshida culture medium [21]. At two and half leaves stage (about 15 days), the seedlings were treated with P-deficiency. P-deficiency (0.016 mM NaH_2_PO_4_) and P-sufficiency (0.32 mM NaH_2_PO_4_) were as treatment and control, respectively. There were 10 seedlings per treatment with three replications. The growth culture solution was renewed every 3 days. After 9 days treatment under P-deficiency stress, leaves and roots under low P-stressed tissues (LLP and RLP) and control group stressed tissues (LCK and RCK) were collected and immediately frozen in liquid nitrogen, then stored at − 80 °C until RNA extraction.

### RNA extraction, cDNA library preparation, and transcriptome sequencing

Total RNA was extracted for three biological replicates from the sampled leaf or root tissues using the TRIzol kit following the instructions (Invitrogen) of manufacturer. The quality and quantity of the resulting RNA were examined using agarose gel electrophoresis and an ND-1000 spectrophotometer (NanoDrop Technology, USA). Magnetic beads with Oligo (dT) were used to isolate mRNA from the total RNA. Mixed with the fragmentation buffer, the mRNA is fragmented into short fragments. Then cDNA was synthesized using the mRNA fragments as templates. Short fragments were purified and resolved with EB buffer for end reparation and poly (A) addition. After that, the short fragments were connected with adapters. After agarose gel electrophoresis, the suitable fragments (200 bp) were selected for the PCR amplification as templates. The library was sequenced using the Illumina HiSeq™ 2000 platform.

### Reads filtration and assessment of differential gene expression

Before assembly, adaptor sequences, empty reads, low quality sequences with ‘N’ percentage over 10% and those containing more than 50% bases with a *Q* < 20 were removed using the Perl program written according to the custom method of program editing. After filtering, the remaining reads were called clean reads and used for downstream bioinformatics analysis. The retained high-quality reads were mapped to the Nipponbare reference genome [22] by TopHat. And then the resulting aligned reads were used to create a RABT (Reference Annotation Based Transcript) assembly using Cufflinks [23]. Expression levels for each gene were calculated by quantifying the reads according to the RPKM (reads per kilobase per million reads) method [24]. We used ‘FDR (false discovery rate) ≤ 0.001 and the absolute value of log_2_ RPKM ratio ≥ 1’ as the threshold to judge the significance of gene expression difference [25].

### Gene ontology (GO) term analysis

Blast2GO program was used to classify unigenes to GO terms based on molecular function, biological processes and cellular components [26] for leaf and root tissues, at *p* < 0.05.

### Validation of transcriptome sequencing

qRT-PCR was performed to confirm 15 randomly selected differentially expressed genes (DEGs) (10 up-regulated and 5 down-regulated genes) from each group of P-deficiency-induced genes identified from RNA sequencing using the SYBR premix Ex Taq kit on a StepOnePlus™ Real-Time PCR System. Diluted cDNA was amplified using gene specific primers (Additional file 1: Table S1) and SYBR Green real-time PCR master mix (Toyobo). All reactions were performed using one biological sample and three technical replicates, and each sample was conducted at least in triplicate and normalized using *OsActin1* as an internal control [27].

## Results and discussion

### Transcriptome sequencing statistics

To understand the molecular mechanism of DXWR under P-deficiency stress, we investigated the gene expression of DXWR in response to P-deficiency stress by transcriptome sequencing. The total RNA was extracted from the leaves of DXWR at seedling stage to construct cDNA library for transcriptome sequencing analysis. In total, 46.78, 48.82, 43.59, and 46.76 million high-quality paired end reads were generated by Illumina-sequencing the LCK, LLP, RCK, and RLP cDNA libraries, respectively (Table 1). The *O. sativa ssp. japonica cv.* Nipponbare genome has been completely sequenced through Sanger sequencing technology which is considered to be the best tool for assembling and annotating the rice genomes [28–30]. Therefore, in this study, we used the Nipponbare genome as reference for reads matching. The alignment results indicated that 73.37–75.20% (71.43–73.72% uniquely matched) of the total reads were mapped to the Nipponbare reference genome and 61.35–63.52% (38.08–39.57% uniquely matched) were mapped to the gene regions (Tables 1, 2). Meanwhile, there was a significant difference in the percentage of reads matched to the genome and the gene region, especially the unique matching reads number, which was similar to previous findings. The previous study reported that about 72% of the total reads mapped to the genome and 46% to the gene regions with 68 and 38% uniquely matched for deep transcriptome sequencing of rhizome and aerial-shoot in *Sorghum propinquum* [31]. This may be due to the fact that reads match the intergenic spacer or alternatively spliced region of mRNA. The 24.80–26.63% of reads remained unmapped, mainly attributable to gene intervals and the differences between DXWR and reference genome sequence (Table 1). Among more than 60% of the mapped genes, on average, at least 50% were covered by the uniquely mapped reads, and only approximately 15% of the genes had gene coverage of 20% or lower (Additional file 2: Figure S1), which suggested that a high quality of transcriptome data was obtained.

**Table 1:** The data of Illumina transcriptome reads mapped to the reference genome (≤ 3 bp mismatch)

Reads mapping	Reads number (%)			
	LCK	LLP	RCK	RLP
Total reads	46,784,432	48,816,718	43,588,908	46,760,112
Total BasePairs	4,678,443,200	4,881,671,800	4,358,890,800	4,676,011,200
Total mapped reads	34,881,473 (74.56)	36,708,638 (75.20)	31,979,139 (73.37)	34,819,568 (74.46)
Perfect match	25,627,140 (54.78)	26,844,106 (54.99)	23,267,595 (53.38)	25,316,122 (54.14)
≤ 3 bp mismatch	9,254,333 (19.78)	9,864,532 (20.21)	8,711,544 (19.99)	9,503,446 (20.32)
Unique match	34,230,815 (73.17)	35,986,019 (73.72)	31,136,589 (71.43)	34,001,830 (72.72)
Multi-position match	650,658 (1.39)	722,619 (1.48)	842,550 (1.93)	817,738 (1.75)
Total unmapped reads	11,902,959 (25.44)	12,108,080 (24.80)	11,609,769 (26.63)	11,940,544 (25.54)

*LLP* leaves under low phosphorus stress treatment, *RLP* roots under low phosphorus stress treatment, *LCK* leaves under phosphorus sufficiency stress treatment, *RCK* roots under phosphorus sufficiency stress treatment. (The same as below.)

**Table 2:** Summary of Illumina transcriptome reads mapped to the reference genes (≤ 5 bp mismatch)

	Reads number(%)			
	LCK	LLP	RCK	RLP
Total reads	46,784,432	48,816,718	43,588,908	46,760,112
Total BasePairs	4,678,443,200	4,881,671,800	4,358,890,800	4,676,011,200
Total mapped reads	29,634,341 (63.34)	31,008,106 (63.52)	26,743,293 (61.35)	29,478,172 (63.04)
Perfect match	22,232,426 (47.52)	23,129,717 (47.38)	19,983,467 (45.85)	21,989,061 (47.03)
≤ 5 bp mismatch	7,401,915 (15.82)	7,878,389 (16.14)	6,759,826 (15.51)	7,489,111 (16.02)
Unique match	17,816,293 (38.08)	18,899,219 (38.71)	16,812,042 (38.57)	18,502,324 (39.57)
Multi-position match	11,818,048 (25.26)	12,108,887 (24.80)	9,931,251 (22.78)	10,975,848 (23.47)
Total unmapped reads	17,150,091 (36.66)	17,808,612 (36.48)	16,845,615 (38.65)	17,281,940 (36.96)

Previous studies have shown that Asian cultivated rice was domesticated from wild varieties (*O. rufipogon*) [32]. Moreover, the sequence of wild rice W1943 has a great similarity with that of Nipponbare [33]. However, some of the sequences in the W1943 cDNAs that could not be matched to the genome may be located in the gap of genomic sequences or may be related to the W1943 specific gene [33], which suggests that the use of the Nipponbare genome to match wild rice (*O. rufipogon*) is limited.

### Analysis of differentially expressed genes

**Fig. 1:**
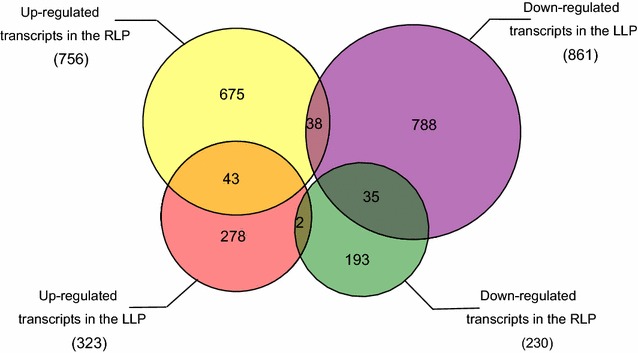
The number of up- and down-regulated transcripts in the LLP and RLP compared with the LCK and RCK. *LLP* leaves under low phosphorus stress treatment, *RLP* roots under low phosphorus stress treatment, *LCK* leaves under phosphorus sufficiency stress treatment, *RCK* roots under phosphorus sufficiency stress treatment. (The same as below.)

The raw data obtained from Illumina sequencing can be used to assess the level of gene expression [34]. Putative DEGs from the P-deficiency-stressed and control samples (LLP vs. LCK and RLP vs. RCK) were identified. In the LLP sample, 323 and 861 transcripts were up- (Additional file 3: Table S2) and down-regulated (Additional file 4: Table S3), respectively, when compared to the LCK sample. In the RLP sample, 756 and 230 transcripts were up- (Additional file 5: Table S4) and down-regulated (Additional file 6: Table S5), respectively, when compared to the RCK sample. Among these DEGs, 43 and 35 transcripts were up- (Additional file 7: Table S6) and down-regulated (Additional file 8: Table S7), respectively, in both the LLP and RLP samples, when compared to the control samples (LCK and RCK). 38 transcripts were up-regulated in the RLP sample but down-regulated in the LLP sample (Additional file 9: Table S8), and only 2 transcripts (*LOC_Os07g08680.1*, a putative protein and *LOC_Os11g37950.1*, a putative *WIP3*-Wound-induced protein precursor) were down-regulated in the RLP sample but up-regulated in the LLP sample. The overall distribution of the number of up and down regulated transcripts in roots and leaves can be seen in Fig. 1.

We detected that the expression of several genes were dramatically changed (the absolute value of log_2_ RPKM ratio > 3), including *LOC_Os01g02460.1*, *LOC_Os02g48850.1*, *LOC_Os01g27590.1* and *LOC_Os03g05334.1* (up regulated in both LLP and RLP), among them, *LOC_Os01g02460.1*, *LOC_Os02g48850.1* may be related to the synthesis of the receptor kinase LRK10 and a specific domain of *tigr01615* family proteins; *LOC_Os01g72360.1*, *LOC_Os01g72370.3*, *LOC_Os02g43410.1*, *LOC_Os07g27030.1*, *LOC_Os10g11889.1*, *LOC_Os10g11889.2* and *LOC_Os11g15624.1* (down regulated in both LLP and RLP), among them, *LOC_Os01g72370.3* may be related to the protein binding domain of helix loop helix DNA; *LOC_Os01g06900.1* and *LOC_Os05g25650.1* (up regulated in RLP but down regulated in LLP), the former function is not clear so far, and the latter may be related to the synthesis of Verticillium wilt resistance protein VE.

Furthermore, many of the detected DEGs represented genes that have been previously identified and demonstrated to play roles in responses to P-deficiency stress in cultivated rice (Table 3).

**Table 3:** The detected DEGs represented genes play roles in cultivated rice responses to P-deficiency stress

Gene name	Gene ID	Up or down Log_2_ ratio		References
		LLP vs. LCK	RLP vs. RCK	
*OsPHR2*	*LOC_Os07g25710*	None	None	[35]
*OsUPS*	*LOC_Os03g13740*	None	None	[36]
*OsPI1*	*LOC_Os05g34940*	None	None	[37]
*OsSPX1*	*LOC_Os06g40120*	Up (1.19)	Up (2.11)	[38]
*OsWRKY74*	*LOC_Os09g16510*	Down (− 1.22)	None	[39]
*OsPupK20*-*2*	*LOC_Os12g26380*	None	Up (2.65)	[40]
*OsPupK05*	*LOC_Os12g26290*	None	Up (2.11)	[40]
*OsPT6*	*LOC_Os08g45000*	None	Up (3.60)	[41]
*OsPT2*	*LOC_Os03g05640*	None	Up (2.16)	[41]
*OsBBX1*	*LOC_Os01g10580*	None	Up (3.32)	[42]
*OsBBX2*	*LOC_Os02g07930*	Down (− 1.48)	None	[42]
*OsBBX7*	*LOC_Os02g49230*	None	Down (− 1.26)	[42]
*OsBBX10*	*LOC_Os03g50310*	None	Up (2.42)	[42]
*OsBBX17*	*LOC_Os06g15330*	None	Up (3.32)	[42]
*OsBBX27*	*LOC_Os09g06464*	None	Up (2.39)	[42]
*OsRCI2*-*9*	*LOC_Os06g44220*	Up (4.96)	None	[43]
*OsbHLH172*	*LOC_Os06g12210*	Up (2.87)	None	[44]

Transcription factors (TFs) have been described as important regulators of transcription and have been reported to play essential roles in abiotic stress responses by regulating a large spectrum of downstream stress responsive genes. In previous studies, *OsPHR2* (*LOC_Os07g25710*) [35], *OsUPS* (*LOC_Os03g13740*) [36] and *OsPI1* (*LOC_Os05g34940*) [37] have been reported to be involved in response to P-deficiency stress. There were also some P-deficiency tolerance related genes had been found in DEGs, including *OsSPX1* (*LOC_Os06g40120*) [38], *OsWRKY74* (*LOC_Os09g16510*) [39], *OsPupK20*-*2* (*LOC_Os12g26380*) [40], *OsPupK05* (*LOC_Os12g26290*) [40], *OsPT6* (*LOC_Os08g45000*) [41], *OsBBX2* (*LOC_Os02g07930*) [42], *OsBBX7* (*LOC_Os02g49230*) [42], *OsRCI2*-*9* (*LOC_Os06g44220*) [43] and *OsbHLH172* (*LOC_Os06g12210*) [44]. Among them, only *OsSPX1* was up-regulated both in leaves and roots of P-deficiency treat, whereas, *OsPupK20*-*2*, *OsPupK05*, *OsPT6*, *OsPT2* and *OsBBX1* up-regulated only in roots; *OsRCI2*-*9* and *OsbHLH172* up-regulated only in leaves; *OsWRKY74* and *OsBBX2* down regulated in leaves; and *OsBBX7* down regulated in roots.

In this study, we found that some genes have been shown to be associated with P-deficiency tolerance, such as MYB family TF (*LOC_Os01g16810.1*), WRKY TFs (*LOC_Os01g60640.1*, *LOC_Os01g61080.1*, *LOC_Os01g53040.1*), NAC TFs (*LOC_Os01g66120.1* and *LOC_Os03g60080.1*), rice bHLH TF (*LOC_Os01g72370.3*), transcription elongation factor (*LOC_Os02g04160.1*), homeodomain leucine zipper TF (*LOC_Os03g10210.1*), NAC transcriptional activator (*LOC_Os03g21060.1*), heat shock TF (*LOC_Os03g25120.1*), Ethylene response TF (*LOC_Os04g46220.1*) and AP2 domain containing protein (*LOC_Os04g52090.1*). This study of gene promoter analysis showed that there were *PHR1* binding sites in response to P stress [45]. *PHR1*, a MYB TF, involved in response to P-deficiency stress [46], which was not very sensitive to P-deficiency stress, and acted a role in the downstream of the phosphorus signal transduction pathway. *PHR1* consists of a MYB domain and a coiled coil domain (perhaps to form a dimer with an imperfect palindromic sequence at specific promoters). It plays a role in maintaining the balance of P under the condition of adequate nutrition. It was also found that the increase of sulfur transporter and iron transporter was regulated by P stress [45]. This suggested that the transcription factors played an important role in the transcriptional regulation of downstream genes in plants under P-deficiency stress. Previous studies have found some zinc finger proteins responded to P-deficiency stress, for example, two C2H2- type zinc finger protein gene *ZOS3*-*12* (*LOC_Os03g32230*) and *ZOS5*-*08* (*LOC_Os05g37190*): the former was proved to be related to nitrogen stress in rice, and the latter was related to defoliation, which suggested that zinc finger proteins played an important role in the regulation of multiple stress tolerance.

Uptake, transport and translocation of P in plants are performed by P transporters (*Pht*). Under P-deficiency stress, lipid changed from phospholipid to galactose and sulfanilamide, phosphatase activity increased, and phosphate monoester decomposed into Pi and the related fatty acid and phosphate transporter gene will be induced [47]. In this study, we also found some transporter genes that have been shown to be associated with P-deficiency tolerance. In the LLP sample, we found three genes editing transmembrane amino acid transporter (*LOC_Os01g41420.1*, *LOC_Os06g12320.1*, *LOC_Os01g61044.2*), two genes editing inorganic phosphate transporter (*LOC_Os03g05620.1* and *LOC_Os06g21950.1*) and eight editing transporter protein (*LOC_Os01g50820.1*, *LOC_Os08g06010.1*, *LOC_Os01g36720.1*, *LOC_Os08g31670.1*, *LOC_Os11g42430.1*, *LOC_Os02g36450.1*, *LOC_Os03g03680.1* and *LOC_Os01g07310.1*). In the RLP sample, we found six up-regulated genes editing inorganic phosphate transporter (*LOC_Os06g21950.1*, *LOC_Os08g45000.1*, *LOC_Os10g30770.1*, *LOC_Os03g05640.1*, *LOC_Os10g30790.1* and *LOC_Os06g21920.1*), among which, *LOC_Os06g21950.1* was also up-regulated in the LLP sample. So far, there are 4 *Pht* families, namely *Pht1*, *Pht2*, *Pht3* and *Pht4*. *Pht1* gene family encodes a high affinity protein (Phosphate transporter) to regulate the rate of P crossing the plasma membrane. In the *Arabidopsis* genome, there are 9 members in the *Pht1* gene family, namely *Pht1;1 *~ *Pht1;9*, four members (*Pht1;1*, *Pht1;2*, *Pht1;3* and *Pht1;4*) expressed promoters, which were induced by P-deficiency stress. What’s more, *Pht1;1 *~ *Pht1;4* were related to plant uptake of Pi from soil. The *Pht1* gene family plays an important role in the process of P transport in plants, and express in flowers, cotyledons, pollen, leaf vascular tissues and shoots [48, 49]. *Pht2* and *Pht3* family encode Pi transporter associated with organelle. *Pht2;1* is the only member of the *Pht2* family and is a low affinity phosphate transporter, which is present in the chloroplast membrane [50, 51]. *Pht3* family has 3 members, and the protein is located in the mitochondria. The *Pht4* gene family consists of 6 members, and its structure is similar to that of P transporter SLC17/type1. The *Pht4* gene expressed in both roots and leaves, 5 of which were present in plastids, and the other in Golgi, suggesting that the *Pht4* gene family is associated with the transport of Pi in the cytosol, plastids and Golgi [52]. Although the function of a low affinity Pi transporter system is present in the root of the plant, the genes encoding these transporters remain to be identified.

The transcriptome changes of cultivated rice to P starvation have been reported in previous study [53]. In this study, we compared the DEGs between DXWR and cultivated rice. We found two genes encoding metallothioneins (*LOC_Os12g38270.2* and *LOC_Os12g38290.1*) were up-redulated in roots, which was same with previous research [53]. Metallothioneins affect metal tolerance and homeostasis and scavenge reactive oxygen species [54], which could be a mechanism to overcome the increase in certain ion concentration, such as iron, upon Pi starvation. Under Pi starvation, plants overaccumulated some ions, including iron [45, 55, 56]. We also found one gene encoded iron transporter (*LOC_Os09g23300.1*) was up-regulated both in roots and leaves and two genes encoded vacuolar iron transporters (*LOC_Os04g45520.1* and *LOC_Os09g23300.1*) were up-regulated in roots and leaves, respectively (*LOC_Os04g45520.1* was up-regulated in roots and *LOC_Os09g23300.1* was up-regulated in leaves). In addition, *LOC_Os08g06010.1*, a putative glycerol 3-phosphate permease, and *LOC_Os03g40670.1*, a putative glycerophosphoryl diester phosphodiesterase suggested to be involved in Pi remobilization [57], were also up-regulated both in roots and leaves after P-deficiency stress. One gene encoded MYB family TF (*LOC_Os02g22020.1*), one encoded transporter (*LOC_Os08g31670.1*) and one encoded glycerophosphoryl diester phosphodiesterase family protein (*LOC_Os02g31030.1*) which were up-regulated both in roots and leaves of DXWR but only up-regulated in leaves of cultivated rice.

**Fig. 2:**
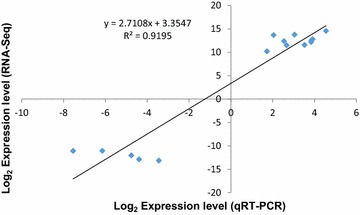
Comparison of the expression of thirty randomly selected genes using RNA-Seq and qRT-PCR. The gene expression values were transformed to log_2_ scale. The qRT-PCR log_2_-value (X-axis) was plotted against the RNA-Seq data log_2_-value (Y-axis)

To confirm the validity of the DEG data, quantitative RT-PCR was performed to investigate the expression patterns of 15 randomly selected genes under the same conditions. Expression trends were consistent for all transcripts in RNA-Seq analysis and quantitative RT-PCR analysis, with a correlation coefficient of (*R*^2^ = 0.9195) (Fig. 2). Thus, the DEGs detected in this study can be considered to be a high accuracy.

### Functional classification by gene ontology

**Fig. 3:**
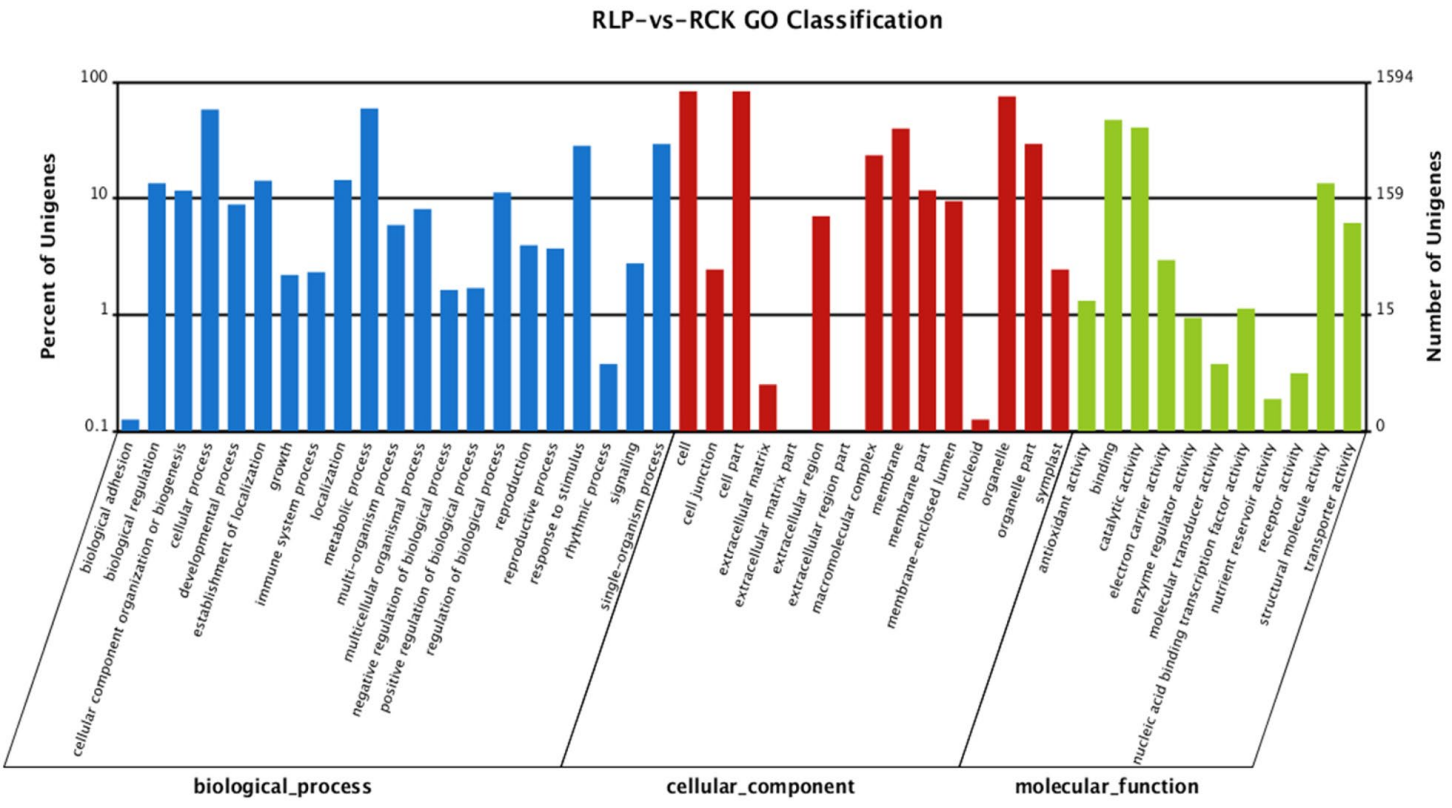
Gene ontology (GO) classification of the unigenes from the RLP-vs.-RCK

**Fig. 4:**
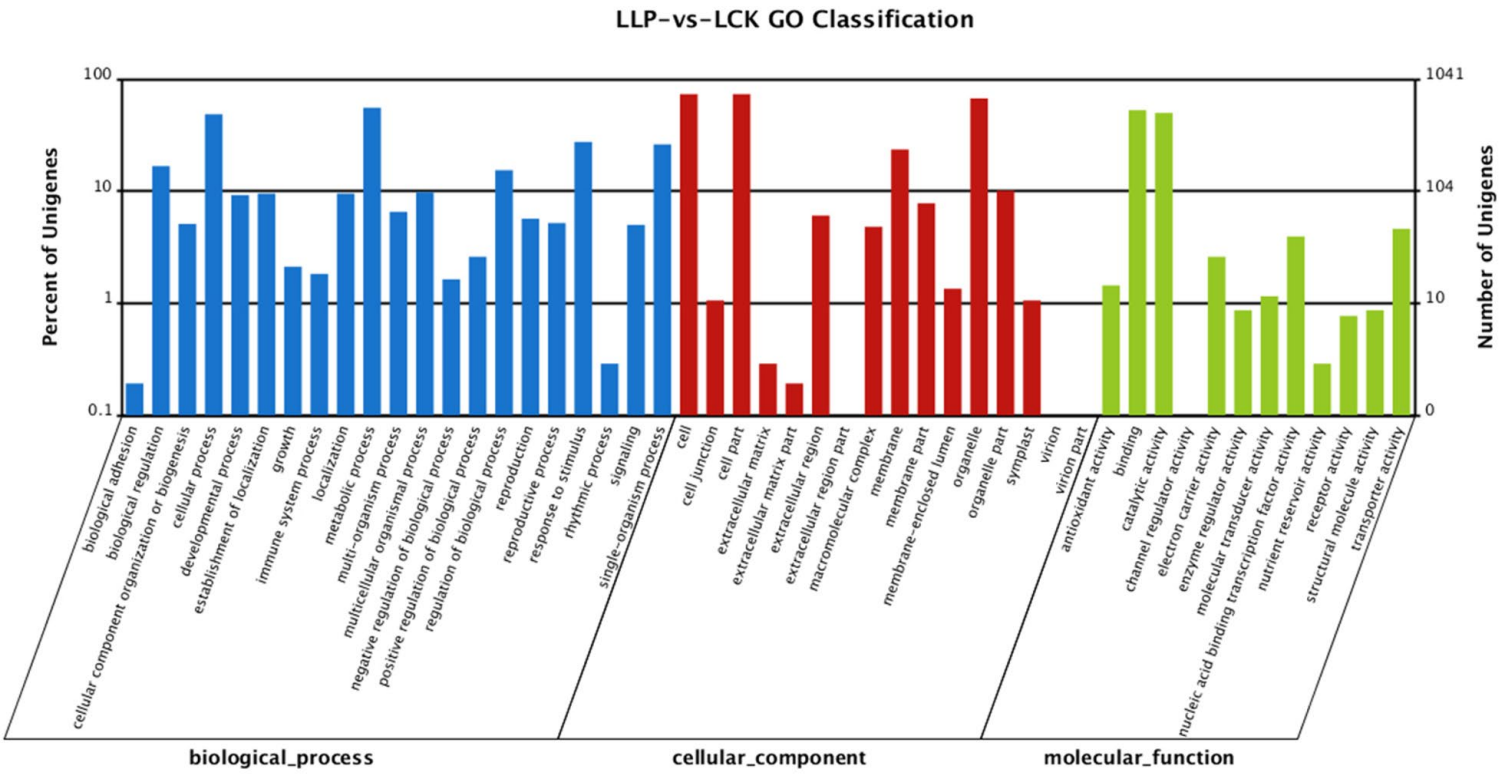
Gene ontology (GO) classification of the unigenes from the LLP–vs.-LCK

The GO gene ontology mapping software (WEGO) was used to classify the function and draw gene ontology tree, and the down and up regulated transcripts in roots and leaves were classified into the functional groups. In the present study, a total of 12,180 root transcripts in RLP vs. RCK and 6803 leaf transcripts in LLP vs. LCK were assigned GO terms. Among the 12,180 root transcripts, 1829 were annotated for their molecular function, 5865 transcripts were annotated for their cellular component, and 4486 were annotated for their biological process (Fig. 3). Among the 6803 leaf transcripts, 1241 were annotated for their molecular function, 2816 transcripts were annotated for their cellular component, and 2746 were annotated for their biological process (Fig. 4). In the classification of biological processes, cellular processes and metabolic processes were the most functional groups, which indicated that the DXWR had a wide range of metabolic activities under the P-deficiency stress. In the classification of cell components, the transcripts of cells, cell components and organelles were the most abundant. In the classification of molecular function, the transcription of immobilized and active were the most highly represented groups.

We also identified biological process, cellular component and molecular function GO terms that were over-represented (*p* < 0.05) among the DEGs of LLP vs. LCK and RLP vs. RCK, respectively (Additional file 10: Table S9, Additional file 11: Table S10). It was found that the expression of transcripts in the extracellular matrix increased in roots and leaves for the cellular component category (GO: 0005576, annotated as extracellular region), suggesting that the same cell components in the root and leaf are involved in the P-deficiency response.

### Kyoto encyclopedia of genes and genomes (KEGG) pathway mapping

**Fig. 5:**
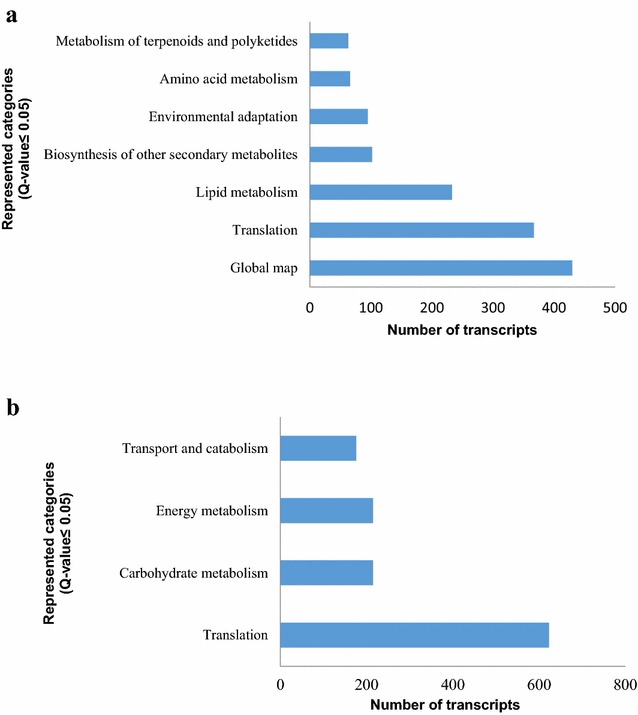
KEGG pathway assignments in the LLP vs. LCK (**a**) and RLP vs. RCK (**b**). The represented categories (*Q* ≤ 0.05) and the number of transcripts predicted to belong to each category are shown

KEGG pathway analysis showed that 700 of the 1184 leaf DEGs and 622 of the 986 root DEGs could be classified into 20 functional categories and 114 and 117 subcategories, respectively. Furthermore, the over-represented KEGG Orthology (KO) terms (*Q* < 0.05) could be classified into 7 and 4 categories, respectively (Fig. 5). As shown in Fig. 5, the most common KO terms represented by both the leaf and root DEGs was the translation KEGG pathways. The over-represented KO terms for the leaf and root DEGs were further classified into 16 and 17 subcategories, respectively (Additional file 12: Table S11, Additional file 13: Table S12). Among these subcategories, 2 subcategories were over-represented among the leaf DEGs, which are RNA transporters and mRNA monitoring pathways, suggesting that these pathway may regulate the expression of P-deficiency inducible genes. Leaf and root did not have the same subcategory. Moreover, 12 KO terms were exclusively enriched among the leaf DEGs and 7 KO terms were exclusively enriched among the root DEGs. This finding suggests that there could be considerable differences in the biochemical and physiological processes involved in the P-deficiency responses of leaves and roots, and these annotations provide a valuable resource for investigating the specific processes, functions, and pathways involved in such differences.

### DEGs mapped to previously identified P-deficiency responses related QTL intervals

Map based cloning is a method for identifying potential QTLs sites. Many QTLs have been identified in DXWR were associated with yield and multiple resistance. Based on the Gramene QTL database, a total of 57 QTLs related to P-deficiency stress have been found. We located 278 genes differentially regulated by P-deficiency stress on 10 of these identified QTL intervals in rice (Additional file 14: Table S13). Among them, the QTL Accession AQBD003, AQCI008, AQCI013, and AQCI011 had the greatest number of co-localized DEGs with 86, 33, 31 and 29 genes, respectively (Additional file 15: Table S14).

86 DEGs were co-localized within the AQBD003 interval, including two genes encoding PHD-finger family protein (*LOC_Os12g06480.1* and *LOC_Os12g06480.2*), four encoding NB-ARC domain protein (*LOC_Os12g31200.1*, *LOC_Os12g17410.1*, *LOC_Os12g10330.1* and *LOC_Os12g10410.1*), and one gene encoding helix-loop-helix DNA-binding domain containing protein (*LOC_Os12g32400.1*). Previous research has found that the NB-ARC domain of OsPDRH9 N protein response to light regulation, biological and abiotic stress [58].

33 DEGs were co-localized within the AQCI008 interval, including one gene encoding AP2 TF (*LOC_Os03g12950.1*), one encoding DHHC zinc finger protein domain (*LOC_Os03g16790.1*), and two genes encoding Ribosomal protein gene (*LOC_Os03g17580.1* and *LOC_Os03g15870.1*). In addition, this interval also included some energy related proteins, such as fructose-1,6-bisphosphatase, glyoxal oxidase, oxidoreductase and protein phosphatase 2C. Protein phosphatase 2C (PP2C) plays an important role in biological signal transduction. PP2C participates in stress response in higher plants. As a negative regulator of most signaling pathways, PP2C can directly bind to kinase or regulatory proteins, as well as directly bind to DNA regulating the expression of related genes [59].

31 DEGs were co-localized within the AQCI013 interval, including three genes encoding NB-ARC domain protein (*LOC_Os12g17410.1*, *LOC_Os12g10330.1* and *LOC_Os12g10410.1*). 29 DEGs were co-localized within the AQCI011 interval, including one gene encoding AP2 TF (*LOC_Os04g42570.1*) and another one encoding ethylene-responsive TF (*LOC_Os04g46220.1*). This interval also included one YABBY domains protein gene (*LOC_Os04g45330.1*). Study has shown that YABBY protein plays an important role in plant abiotic stress [60].

### Comparative analysis of the DEGs and previously identified P-deficiency-related QTL intervals in DXWR

Until now, a total of 23 P-deficiency-related QTLs on chromosomes 1, 2, 3, 7, 8, 9 and 11 were identified in DXWR [61]. Among the 23 QTLs, the QTL cluster (the region RM254-RM1233) on chromosome 11 was high significant for P-deficiency tolerant (LOD 3.31 and LOD 3.52 for *qFWS*-*4* and *qFWR*-*4*, respectively) [61].

In the present study, 13 DEGs were co-localized within the *qFWS*-*4* interval (Additional file 16: Table S15), including one gene encoding bZIP TF (*LOC_Os11g05640.1*), one encoding MYB-like DNA-binding domain protein (*LOC_Os11g03440.1*), one encoding calmodulin depedent protein kinases (*LOC_Os11g02240.1*), one encoding auxin-repressed protein (*LOC_Os11g44810.2*), regulating transcription of auxin-responsive genes [62]. And another one gene encoded homeobox domain containing protein (*LOC_Os11g06020.1*), whose products are transcription factors sharing a characteristic protein fold structure that bound DNA [63–65], regulating gene transcription. Furthermore, another gene, *LOC_Os11g44310.1*, which encoded calmodulin binding protein, regulated the expression of calmodulin to regulate the intracellular calcium concentration to control cells in many important biochemical reactions, which might play an important role in stress responses [66].

11 DEGs were co-localized within the *qFWR*-*4* interval (Additional file 17: Table S16), including one gene encoding phosphoglycerate mutase (*LOC_Os11g05260.1*), which was the key enzyme in sugar metabolism process, regulating the adaptation of plants to environment. Another one encoded nucleoside-triphosphatase (*LOC_Os11g03290.1*) [67], which maintained the connection between the nucleus cytoplasm and cytoplasm, including transcription and regulating biosynthesis to adapt to the environment. The interval also included *LOC_Os11g05400.1*, which encoded Ser/Thr protein phosphatase family protein, modified other proteins by chemically adding phosphate groups to them and, thus, regulated cellular pathways, signal transduction, and responses to biotic and abiotic stresses [68]. Meanwhile, *LOC_Os11g04300.1* and *LOC_Os11g03940.1* encoded retrotransposon proteins, whose replicative mode of transposition by means of an RNA intermediate rapidly increased the copy numbers of elements and thereby could increase genome size, which could induce mutations by inserting near or within genes to response to biotic and abiotic stresses. *LOC_Os11g04290.1* and *LOC_Os11g05380.1*, which encoded cytochrome P450, modified other proteins by chemically adding OH group to them or as an enzyme catalyzed reaction and [69], thus, regulated cellular pathways, signal transduction, and responses to biotic and abiotic stresses.

## Conclusion

DXWR has a lot of useful agronomic traits we need, such as resistance of cold, drought, salt and P-deficiency. Therefore, it is considered to be an important resource for rice breeding. In this study, we analyzed the transcriptome of roots and leaves of DXWR seedlings under P-deficiency stress. A large number of DEGs and some critical paths were identified, such as RNA transport and mRNA monitoring path. By combining the DEGs identified in the present study with previously identified P-deficiency resistance QTLs from rice, important candidate genes were identified, including a variety of transcription factors and some functional protein genes. These findings will be useful in the future studies of molecular adaptations to P-deficiency stress and will facilitate the genetic manipulation of rice to improve its P-deficiency resistance.

## Additional files

**Additional file 1: Table S1.** Primers used for real-time PCR in this study.

**Additional file 2: Figure S1.** Distribution of genes coverage in the leaves and roots of Dongxiang wild rice (DXWR) seedlings with or without low phosphorus treatment, respectively. A leaves without low phosphorus treatment (LCK). B leaves with low phosphorus treatment (LLP). C roots without low phosphorus treatment (RCK). D roots with low phosphorus treatment (RLP). Gene coverage is the percentage of a gene that is covered by reads and defined as the ratio of the number of bases in a gene covered by uniquely mapped reads to the number of total bases in the gene. The pie graph demonstrates the detailed percentage of the different gene coverage listing on the left of the pie graph.

**Additional file 3: Table S2.** List of up-regulated genes in LLP vs. LCK.

**Additional file 4: Table S3.** List of down-regulated genes in LLP vs. LCK.

**Additional file 5: Table S4.** List of up-regulated genes in RLP vs. RCK.

**Additional file 6: Table S5.** List of down-regulated genes in RLP vs. RCK.

**Additional file 7: Table S6.** List of up-regulated genes both in the LLP vs. LCK and RLP vs. RCK.

**Additional file 8: Table S7.** List of down-regulated genes both in the LLP vs. LCK and RLP vs. RCK.

**Additional file 9: Table S8.** List of down-regulated genes in the LLP vs. LCK but up-regulated in the RLP vs. RCK.

**Additional file 10: Table S9.** Significant GO terms of DEGs in the biological process, cellular component and molecular function category for LLP vs. LCK.

**Additional file 11: Table S10.** Significant GO terms of DEGs in the biological process, cellular component and molecular function category for RLP vs. RCK.

**Additional file 12: Table S11.** Significant KO terms of DEGs in the LLP vs. LCK (*Q*-value < 0.05).

**Additional file 13: Table S12.** Significant KO terms of DEGs in the RLP vs. RCK (*Q*-value < 0.05).

**Additional file 14: Table S13.** Previously identified P-deficiency responses related QTL intervals.

**Additional file 15: Table S14.** DEGs mapped to previously identified P-deficiency responses related QTL intervals.

**Additional file 16: Table S15.** Co-localized DEGs within the *qFWS-4* interval.

**Additional file 17: Table S16.** Co-localized DEGs within the *qFWR-4* interval.

## Abbreviations

P: phosphorus
DXWR: Dongxiang wild rice
DEGs: differentially expressed genes
FDR: false discovery rate
GO: gene ontology
KEGG: Kyoto Encyclopedia of Genes and Genomes
KO: KEGG Orthology
RPKM: reads per kilobase per million reads
QTLs: quantitative trait loci
TFs: transcription factors
qRT-PCR: quantitative real-time PCR
NAC: NAM, ATAF, and CUC
PP: protein phosphatase
AP2: APETALA2
bHLH: basic helix-loop-helix
WEGO: Web Gene Ontology Annotation Plot
bZIP: basic leucine zipper
