# Supplementary material for: Transcriptome analysis of phosphorus stress responsiveness in the seedlings of Dongxiang wild rice (Oryza rufipogon Griff.)
Source: Biol Res. 2018 Mar 15;51:7. doi: 10.1186/s40659-018-0155-x (PMC5853122; doi:10.1186/s40659-018-0155-x)
Supplement: Supplementary file 1 — Additional file 1: Table S1. Primers used for real-time PCR in this study. [file 40659_2018_155_MOESM1_ESM.docx]

| **Table S1** Primers used for real-time PCR in this study. | | |
| --- | --- | --- |
| Primer name | Gene ID | Primer sequence (5'-3') |
| 1590.1F | *LOC_Os01g27590.1* | TGGCAAGTTAAAGGGTTCCA |
| 1590.1R |  | TGCTGTAAGGTGGGCAATTT |
| 1694.1F | *LOC_Os01g19694.1* | AGGTCTTGACCCAAAGCAGA |
| 1694.1R |  | TGGCCAATTTTCTCAAGAGG |
| 1370.3F | *LOC_Os01g72370.3* | CCCAGTACACCAGCTTCACC |
| 1370.3R |  | TCTTCTTCCATCTGCAAGCTC |
| 8240.1F | *LOC_Os08g04240.1* | CCCCTTCTACTTCCCACTCG |
| 8240.1R |  | TGCATTATCGCATTGTACGC |
| 1359.2F | *LOC_Os01g38359.2* | CGAGTGCAGCCACAAACAG |
| 1359.2R |  | TGATCCGGTGATGACACAGT |
| 8210.1F | *LOC_Os08g04210.1* | CAGGGTGCCAGATCGACTAT |
| 8210.1R |  | ACTGAAATTAAACGTCAACACAAG |
| 9930.1F | *LOC_Os09g17930.1* | TCAGATCAGGCAGTGAGTGG |
| 9930.1R |  | ACGTGTAGGACCACGAAACC |
| 1930.1F | *LOC_Os01g71930.1* | CACTGCCGACGTACACACTA |
| 1930.1R |  | TGCACGACTCTGGTGATTCT |
| 7715.1F | *LOC_Os07g07715.1* | TGCCTCATCTGCTTCATCTG |
| 7715.1R |  | CGTTGGTGATGACTTTGGTG |
| 3510.1F | *LOC_Os03g12510.1* | TGCTTGAGACGATCAAGGAG |
| 3510.1R |  | ACGAGCACAATGCAACAAAG |
| 7340.1F | *LOC_Os07g41340.1* | GTAAAAGTGCGGTGCTGGAG |
| 7340.1R |  | ATCGTCTTCGTCTTCGTCGT |
| 8570.1F | *LOC_Os08g08570.1* | TCCAGAAGAAAAGGGACCTG |
| 8570.1R |  | GGTCCATCCTTGCATCAGTT |
| 7030.1F | *LOC_Os07g27030.1* | AACATCCTCCATCGTCTTGC |
| 7030.1R |  | ATATGCCACCCACCAAGTGT |
| 3427.1F | *LOC_Os03g19427.1* | TGATCAACTCCGTCATCGTC |
| 3427.1R |  | GCCATAATATAGTGCGCCTTTC |
| 9210.1F | *LOC_Os09g03210.1* | ATCTACGGCCCATACATCCA |
| 9210.1R |  | CTCCTCTTCCTCGTCCTCCT |
