# Supplementary material for: Transcriptome analysis of phosphorus stress responsiveness in the seedlings of Dongxiang wild rice (Oryza rufipogon Griff.)
Source: Biol Res. 2018 Mar 15;51:7. doi: 10.1186/s40659-018-0155-x (PMC5853122; doi:10.1186/s40659-018-0155-x)
Supplement: Supplementary file 2 — Additional file 2: Figure S1. Distribution of genes coverage in the leaves and roots of Dongxiang wild rice (DXWR) seedlings with or without low phosphorus treatment, respectively. A leaves without low phosphorus treatment (LCK). B leaves with low phosphorus treatment (LLP). C roots without low phosphorus treatment (RCK). D roots with low phosphorus treatment (RLP). Gene coverage is the percentage of a gene that is covered by reads and defined as the ratio of the number of bases in a gene covered by uniquely mapped reads to the number of total bases in the gene. The pie graph demonstrates the detailed percentage of the different gene coverage listing on the left of the pie graph. [file 40659_2018_155_MOESM2_ESM.docx]

**Fig. S1**  Distribution of genes coverage in the leaves and roots of Dongxiang wild rice (DXWR) seedlings with or without low phosphorus treatment, respectively.


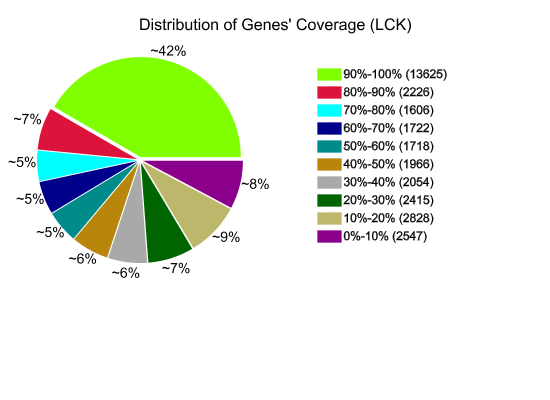


**A**


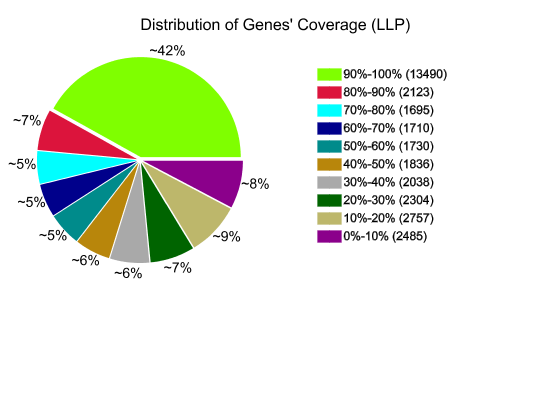


**B**


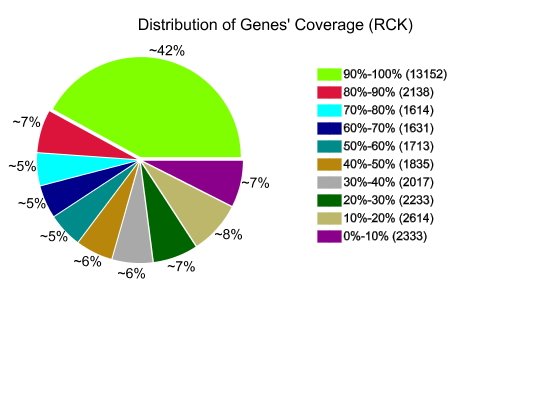


**C**


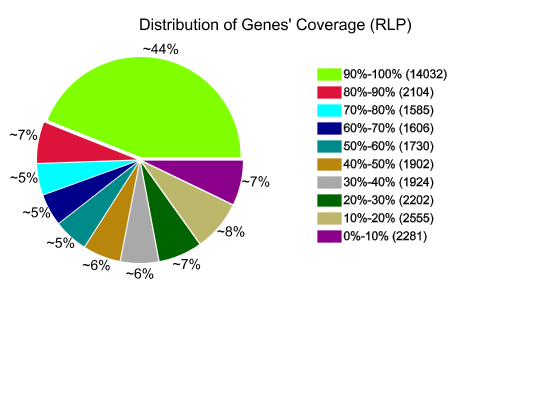


**D**

**A** leaves without low phosphorus treatment (LCK). **B** leaves with low phosphorus treatment (LLP). **C** roots without low phosphorus treatment (RCK). **D** roots with low phosphorus treatment (RLP). Gene coverage is the percentage of a gene that is covered by reads and defined as the ratio of the number of bases in a gene covered by uniquely mapped reads to the number of total bases in the gene. The pie graph demonstrates the detailed percentage of the different gene coverage listing on the left of the pie graph.
