# Supplementary material for: Transcriptome analysis of phosphorus stress responsiveness in the seedlings of Dongxiang wild rice (Oryza rufipogon Griff.)
Source: Biol Res. 2018 Mar 15;51:7. doi: 10.1186/s40659-018-0155-x (PMC5853122; doi:10.1186/s40659-018-0155-x)
Supplement: Supplementary file 3 — Additional file 3: Table S2. List of up-regulated genes in LLP vs. LCK. [file 40659_2018_155_MOESM3_ESM.docx]

| **Table S2** List of up-regulated genes in LLP vs. LCK. | | | | | | | | |
| --- | --- | --- | --- | --- | --- | --- | --- | --- |
| Gene ID | Gene  Length | LLP-  Expression | LCK-  Expression | LLP-RPKM | LCK-RPKM | log_2_ Ratio  (LCK/LLP) | *P*-value | FDR |
| *LOC_Os01g27590.1* | 1131 | 101 | 0 | 4.72514251 | 0.001 | -12.20614213 | 7.65E-30 | 3.14E-28 |
| *LOC_Os01g44170.1* | 1428 | 116 | 0 | 4.298193116 | 0.001 | -12.06951459 | 3.61E-34 | 1.73E-32 |
| *LOC_Os07g05360.2* | 252 | 15 | 0 | 3.149538059 | 0.001 | -11.62092453 | 4.86E-05 | 0.000332357 |
| *LOC_Os02g05410.1* | 2451 | 88 | 0 | 1.899745845 | 0.001 | -10.89159071 | 4.29E-26 | 1.53E-24 |
| *LOC_Os02g10390.1* | 810 | 16 | 0 | 1.045180037 | 0.001 | -10.02953576 | 2.50E-05 | 0.000181126 |
| *LOC_Os07g42790.1* | 897 | 14 | 0 | 0.825832053 | 0.001 | -9.689704604 | 9.44E-05 | 0.000610062 |
| *LOC_Os03g06120.2* | 1257 | 16 | 0 | 0.673505036 | 0.001 | -9.395544923 | 2.50E-05 | 0.000181163 |
| *LOC_Os03g51990.1* | 1656 | 17 | 0 | 0.543181201 | 0.001 | -9.085289741 | 1.29E-05 | 9.76E-05 |
| *LOC_Os02g14280.1* | 3117 | 26 | 0 | 0.441359713 | 0.001 | -8.785811136 | 3.27E-08 | 3.60E-07 |
| *LOC_Os02g56400.1* | 2241 | 18 | 0 | 0.424997907 | 0.001 | -8.731311925 | 6.63E-06 | 5.27E-05 |
| *LOC_Os06g12370.1* | 2046 | 16 | 0 | 0.413780953 | 0.001 | -8.692723428 | 2.50E-05 | 0.000181089 |
| *LOC_Os12g17410.1* | 2355 | 16 | 0 | 0.359488675 | 0.001 | -8.489802513 | 2.50E-05 | 0.000181052 |
| *LOC_Os07g46490.2* | 2082 | 14 | 0 | 0.355797959 | 0.001 | -8.474914426 | 9.44E-05 | 0.000609839 |
| *LOC_Os01g15380.1* | 2292 | 14 | 0 | 0.32319867 | 0.001 | -8.336277451 | 9.44E-05 | 0.00060995 |
| *LOC_Os06g24040.1* | 312 | 70 | 2 | 11.87133576 | 0.359797429 | -5.044153589 | 4.25E-18 | 1.03E-16 |
| *LOC_Os06g44220.1* | 237 | 33 | 1 | 7.367527004 | 0.236828687 | -4.959264692 | 5.47E-09 | 6.57E-08 |
| *LOC_Os02g48850.1* | 864 | 50 | 2 | 3.062050891 | 0.129926849 | -4.558726762 | 1.32E-12 | 2.25E-11 |
| *LOC_Os11g42850.1* | 1032 | 47 | 2 | 2.40976284 | 0.108775967 | -4.469459424 | 8.64E-12 | 1.38E-10 |
| *LOC_Os07g37385.1* | 930 | 46 | 2 | 2.617164529 | 0.120706234 | -4.438432528 | 1.61E-11 | 2.50E-10 |
| *LOC_Os11g13720.1* | 537 | 23 | 1 | 2.266259788 | 0.104522158 | -4.438432528 | 3.03E-06 | 2.55E-05 |
| *LOC_Os09g36680.1* | 759 | 12105 | 629 | 843.8770195 | 46.51483912 | -4.181269824 | 0 | 0 |
| *LOC_Os11g43500.1* | 1275 | 19 | 1 | 0.788496116 | 0.044022274 | -4.162798086 | 3.65E-05 | 0.000255972 |
| *LOC_Os04g13880.1* | 1935 | 17 | 1 | 0.464862051 | 0.029006924 | -4.002333414 | 0.000125326 | 0.000789362 |
| *LOC_Os11g43760.1* | 1923 | 135 | 8 | 3.714587789 | 0.233503479 | -3.991686169 | 1.47E-29 | 5.95E-28 |
| *LOC_Os01g51450.1* | 906 | 29 | 2 | 1.693658877 | 0.12390375 | -3.772851567 | 5.57E-07 | 5.24E-06 |
| *LOC_Os03g05334.1* | 192 | 282 | 22 | 77.7148516 | 6.431379038 | -3.594990306 | 1.20E-55 | 9.77E-54 |
| *LOC_Os02g31030.1* | 1164 | 25 | 2 | 1.136431258 | 0.096440548 | -3.558726762 | 6.11E-06 | 4.88E-05 |
| *LOC_Os06g38040.1* | 1869 | 24 | 2 | 0.679450907 | 0.060062492 | -3.499833073 | 1.11E-05 | 8.47E-05 |
| *LOC_Os08g31680.1* | 4284 | 35 | 3 | 0.432289537 | 0.039305601 | -3.459191089 | 9.44E-08 | 9.82E-07 |
| *LOC_Os11g36510.1* | 2802 | 22 | 2 | 0.415442279 | 0.040063097 | -3.374302191 | 3.59E-05 | 0.000252057 |
| *LOC_Os04g02530.1* | 420 | 22 | 2 | 2.771593492 | 0.26727809 | -3.374302191 | 3.59E-05 | 0.000252007 |
| *LOC_Os03g25010.1* | 1020 | 20 | 2 | 1.03749489 | 0.110055684 | -3.236798667 | 0.000115161 | 0.000730539 |
| *LOC_Os01g02460.1* | 915 | 29 | 3 | 1.676999937 | 0.184027537 | -3.187889067 | 3.08E-06 | 2.59E-05 |
| *LOC_Os04g34530.1* | 1074 | 83 | 9 | 4.089120921 | 0.470349711 | -3.119985002 | 1.92E-15 | 3.98E-14 |
| *LOC_Os03g06360.2* | 738 | 33 | 4 | 2.365994444 | 0.304218964 | -2.959264692 | 1.45E-06 | 1.29E-05 |
| *LOC_Os03g18060.1* | 5067 | 95 | 12 | 0.992039223 | 0.132926936 | -2.89976368 | 2.25E-16 | 4.98E-15 |
| *LOC_Os06g12210.1* | 264 | 31 | 4 | 6.213179625 | 0.850430286 | -2.869066883 | 4.40E-06 | 3.61E-05 |
| *LOC_Os05g50780.1* | 1506 | 23 | 3 | 0.808088649 | 0.11180956 | -2.853470028 | 9.11E-05 | 0.000590145 |
| *LOC_Os12g43380.1* | 534 | 30 | 4 | 2.972597719 | 0.420437445 | -2.821761168 | 7.63E-06 | 6.02E-05 |
| *LOC_Os08g12940.1* | 1131 | 30 | 4 | 1.403507676 | 0.198508926 | -2.821761168 | 7.63E-06 | 6.01E-05 |
| *LOC_Os04g43360.1* | 1551 | 37 | 5 | 1.262252003 | 0.180942614 | -2.802395843 | 6.55E-07 | 6.10E-06 |
| *LOC_Os02g03200.1* | 1905 | 66 | 9 | 1.833179947 | 0.265173538 | -2.78933969 | 2.34E-11 | 3.57E-10 |
| *LOC_Os02g22020.1* | 1239 | 245 | 34 | 10.4628722 | 1.54024662 | -2.76404567 | 3.72E-38 | 2.00E-36 |
| *LOC_Os11g10610.1* | 3468 | 99 | 14 | 1.510470502 | 0.226585232 | -2.73687227 | 4.24E-16 | 9.24E-15 |
| *LOC_Os01g71340.1* | 993 | 1960 | 281 | 104.4390626 | 15.88326292 | -2.717082191 | 2.89E-288 | 1.90E-285 |
| *LOC_Os06g38760.1* | 3615 | 69 | 10 | 1.009943158 | 0.15526528 | -2.701466934 | 1.81E-11 | 2.79E-10 |
| *LOC_Os01g03280.1* | 723 | 27 | 4 | 1.975975745 | 0.310530561 | -2.669758075 | 3.90E-05 | 0.000271609 |
| *LOC_Os12g01410.1* | 1302 | 66 | 10 | 2.68218725 | 0.431093693 | -2.637336597 | 8.91E-11 | 1.29E-09 |
| *LOC_Os11g33120.1* | 2811 | 26 | 4 | 0.489405274 | 0.079869653 | -2.615310291 | 6.66E-05 | 0.000444737 |
| *LOC_Os11g10550.1* | 3924 | 165 | 26 | 2.224903032 | 0.371900706 | -2.580753068 | 2.13E-24 | 7.03E-23 |
| *LOC_Os05g15770.1* | 894 | 3154 | 500 | 186.6724866 | 31.39172197 | -2.572053232 | 0 | 0 |
| *LOC_Os01g14550.1* | 723 | 25 | 4 | 1.829607171 | 0.310530561 | -2.558726762 | 0.000113429 | 0.000720843 |
| *LOC_Os12g30180.1* | 1437 | 25 | 4 | 0.920533044 | 0.156237714 | -2.558726762 | 0.000113429 | 0.000720713 |
| *LOC_Os11g41210.1* | 1488 | 30 | 5 | 1.06677902 | 0.188603491 | -2.499833073 | 2.77E-05 | 0.000198572 |
| *LOC_Os12g25200.1* | 1878 | 228 | 38 | 6.42385015 | 1.135718401 | -2.499833073 | 4.94E-32 | 2.19E-30 |
| *LOC_Os10g40430.1* | 441 | 47 | 8 | 5.639172905 | 1.018202247 | -2.469459424 | 1.51E-07 | 1.52E-06 |
| *LOC_Os11g34950.2* | 1128 | 40 | 7 | 1.876320546 | 0.348314532 | -2.429443745 | 1.69E-06 | 1.48E-05 |
| *LOC_Os06g21950.1* | 1659 | 45 | 8 | 1.435232533 | 0.270661357 | -2.406723669 | 4.20E-07 | 4.02E-06 |
| *LOC_Os08g37930.1* | 411 | 142 | 26 | 18.28111434 | 3.550701632 | -2.364177974 | 2.66E-19 | 6.90E-18 |
| *LOC_Os11g03640.1* | 2109 | 81 | 15 | 2.032191271 | 0.399206251 | -2.34782998 | 1.67E-11 | 2.58E-10 |
| *LOC_Os07g35940.1* | 1467 | 5283 | 979 | 190.5489848 | 37.45719325 | -2.346847217 | 0 | 0 |
| *LOC_Os12g09700.1* | 924 | 86 | 16 | 4.924732238 | 0.971920327 | -2.341135327 | 4.24E-12 | 6.96E-11 |
| *LOC_Os01g64120.1* | 498 | 91 | 17 | 9.668702378 | 1.916029681 | -2.335202371 | 1.08E-12 | 1.85E-11 |
| *LOC_Os11g35400.4* | 1596 | 32 | 6 | 1.06089703 | 0.211009018 | -2.329908072 | 3.16E-05 | 0.000223993 |
| *LOC_Os11g41380.1* | 1968 | 32 | 6 | 0.860361616 | 0.171123167 | -2.329908072 | 3.16E-05 | 0.000223948 |
| *LOC_Os08g20420.1* | 1185 | 37 | 7 | 1.652112116 | 0.331560162 | -2.316969016 | 7.73E-06 | 6.09E-05 |
| *LOC_Os02g39570.1* | 1455 | 30 | 6 | 1.090974008 | 0.231457315 | -2.236798667 | 8.60E-05 | 0.000561003 |
| *LOC_Os07g36080.2* | 579 | 40 | 8 | 3.65542241 | 0.775521919 | -2.236798667 | 5.16E-06 | 4.18E-05 |
| *LOC_Os01g51840.2* | 1725 | 40 | 8 | 1.226950479 | 0.260305618 | -2.236798667 | 5.16E-06 | 4.18E-05 |
| *LOC_Os09g38930.1* | 2133 | 30 | 6 | 0.744194647 | 0.157885791 | -2.236798667 | 8.60E-05 | 0.000561107 |
| *LOC_Os08g31670.1* | 1527 | 311 | 63 | 10.77649407 | 2.315709973 | -2.218361419 | 1.28E-37 | 6.73E-36 |
| *LOC_Os12g34062.1* | 378 | 44 | 9 | 6.159096648 | 1.336390449 | -2.20437719 | 2.09E-06 | 1.80E-05 |
| *LOC_Os01g42060.1* | 1908 | 29 | 6 | 0.804221668 | 0.176504399 | -2.187889067 | 0.000140955 | 0.000877956 |
| *LOC_Os10g26410.1* | 267 | 53 | 11 | 10.50317861 | 2.312405946 | -2.183359408 | 2.11E-07 | 2.10E-06 |
| *LOC_Os03g05440.1* | 1137 | 43 | 9 | 2.001078535 | 0.444288118 | -2.171210326 | 3.40E-06 | 2.84E-05 |
| *LOC_Os05g22840.1* | 516 | 66 | 14 | 6.767844573 | 1.522863535 | -2.15190977 | 8.84E-09 | 1.04E-07 |
| *LOC_Os05g13490.1* | 870 | 37 | 8 | 2.250290641 | 0.516123208 | -2.124323938 | 2.23E-05 | 0.000162753 |
| *LOC_Os08g17784.1* | 1869 | 36 | 8 | 1.019176361 | 0.240249968 | -2.084795574 | 3.60E-05 | 0.000252565 |
| *LOC_Os11g43040.1* | 1890 | 36 | 8 | 1.007852179 | 0.237580524 | -2.084795574 | 3.60E-05 | 0.000252615 |
| *LOC_Os02g57860.1* | 1485 | 475 | 107 | 16.92479038 | 4.044268471 | -2.065187289 | 7.34E-52 | 5.63E-50 |
| *LOC_Os08g29400.1* | 1464 | 44 | 10 | 1.590258561 | 0.383390703 | -2.052374096 | 5.73E-06 | 4.60E-05 |
| *LOC_Os01g68598.1* | 372 | 116 | 27 | 16.49951551 | 4.073835402 | -2.017964065 | 2.02E-13 | 3.67E-12 |
| *LOC_Os09g25390.1* | 2454 | 68 | 16 | 1.466190823 | 0.365955331 | -2.002333414 | 2.45E-08 | 2.75E-07 |
| *LOC_Os02g42060.1* | 444 | 55 | 13 | 6.554444069 | 1.643399066 | -1.995790568 | 5.91E-07 | 5.54E-06 |
| *LOC_Os06g10820.1* | 636 | 50 | 12 | 4.159767248 | 1.059026394 | -1.973764261 | 2.33E-06 | 1.99E-05 |
| *LOC_Os02g53240.1* | 411 | 131 | 32 | 16.86497168 | 4.370094316 | -1.948293574 | 2.27E-14 | 4.42E-13 |
| *LOC_Os06g05250.2* | 1992 | 48 | 12 | 1.27499372 | 0.338122885 | -1.914870572 | 5.82E-06 | 4.67E-05 |
| *LOC_Os08g14190.1* | 1047 | 104 | 26 | 5.255848039 | 1.393828434 | -1.914870572 | 1.86E-11 | 2.87E-10 |
| *LOC_Os09g37500.1* | 453 | 60 | 15 | 7.008243628 | 1.858556254 | -1.914870572 | 3.77E-07 | 3.64E-06 |
| *LOC_Os04g51520.1* | 966 | 44 | 11 | 2.410081297 | 0.639143258 | -1.914870572 | 1.46E-05 | 0.000109443 |
| *LOC_Os03g64310.1* | 312 | 60 | 15 | 10.17543065 | 2.698480715 | -1.914870572 | 3.77E-07 | 3.64E-06 |
| *LOC_Os03g27910.1* | 3981 | 63 | 16 | 0.83734516 | 0.225585125 | -1.892150496 | 2.38E-07 | 2.36E-06 |
| *LOC_Os03g03680.1* | 2136 | 55 | 14 | 1.362440621 | 0.367882764 | -1.888875364 | 1.47E-06 | 1.30E-05 |
| *LOC_Os03g41330.1* | 699 | 47 | 12 | 3.557761447 | 0.963577663 | -1.884496923 | 9.14E-06 | 7.12E-05 |
| *LOC_Os12g39360.1* | 1341 | 340 | 87 | 13.41548202 | 3.641439748 | -1.881318013 | 2.00E-33 | 9.28E-32 |
| *LOC_Os03g22620.1* | 1215 | 1107 | 284 | 48.20892922 | 13.11972451 | -1.87756296 | 9.48E-105 | 1.65E-102 |
| *LOC_Os08g35720.1* | 2238 | 35 | 9 | 0.827492573 | 0.225717422 | -1.874228588 | 0.000145562 | 0.000903787 |
| *LOC_Os11g45990.1* | 1902 | 953 | 250 | 26.51175822 | 7.3775498 | -1.845418692 | 2.22E-88 | 3.24E-86 |
| *LOC_Os08g01570.1* | 1890 | 49 | 13 | 1.371798799 | 0.386068352 | -1.829140698 | 8.91E-06 | 6.96E-05 |
| *LOC_Os04g56680.1* | 432 | 90 | 24 | 11.02338321 | 3.118244382 | -1.821761168 | 1.58E-09 | 2.01E-08 |
| *LOC_Os01g18620.1* | 1869 | 86 | 23 | 2.434699084 | 0.690718659 | -1.817573371 | 3.88E-09 | 4.76E-08 |
| *LOC_Os06g27860.1* | 1119 | 52 | 14 | 2.458835074 | 0.702231979 | -1.807955368 | 5.55E-06 | 4.47E-05 |
| *LOC_Os01g66710.1* | 1227 | 141 | 38 | 6.080379588 | 1.738287822 | -1.806494411 | 5.13E-14 | 9.69E-13 |
| *LOC_Os01g52230.1* | 825 | 211 | 58 | 13.53270607 | 3.945996527 | -1.777988766 | 7.39E-20 | 1.98E-18 |
| *LOC_Os03g14642.1* | 447 | 47 | 13 | 5.563479309 | 1.632369542 | -1.769019706 | 2.14E-05 | 0.000156597 |
| *LOC_Os02g07460.1* | 393 | 50 | 14 | 6.731837072 | 1.999484947 | -1.75137184 | 1.32E-05 | 0.000100157 |
| *LOC_Os03g06700.1* | 816 | 57 | 16 | 3.696075546 | 1.100556841 | -1.747760587 | 3.30E-06 | 2.76E-05 |
| *LOC_Os01g28500.1* | 504 | 688 | 194 | 72.22940615 | 21.60497893 | -1.741222485 | 1.77E-59 | 1.55E-57 |
| *LOC_Os11g42850.2* | 948 | 46 | 13 | 2.567471532 | 0.769693234 | -1.73799281 | 3.29E-05 | 0.000233119 |
| *LOC_Os05g38150.2* | 2151 | 56 | 16 | 1.377538543 | 0.417505524 | -1.722225494 | 5.06E-06 | 4.11E-05 |
| *LOC_Os03g40670.1* | 1176 | 655 | 188 | 29.47067755 | 8.972907303 | -1.715632817 | 1.40E-55 | 1.14E-53 |
| *LOC_Os06g11650.1* | 945 | 87 | 25 | 4.871285531 | 1.484878277 | -1.713957878 | 1.29E-08 | 1.49E-07 |
| *LOC_Os11g37950.1* | 453 | 66 | 19 | 7.709067991 | 2.354171255 | -1.711337178 | 7.88E-07 | 7.24E-06 |
| *LOC_Os01g25484.2* | 1791 | 1000 | 292 | 29.54340558 | 9.151028739 | -1.690830298 | 2.91E-82 | 3.86E-80 |
| *LOC_Os04g51920.2* | 690 | 41 | 12 | 3.144060601 | 0.976146067 | -1.687460076 | 0.000125985 | 0.000792807 |
| *LOC_Os07g19320.1* | 2874 | 75 | 22 | 1.380799567 | 0.429653714 | -1.684257644 | 1.88E-07 | 1.88E-06 |
| *LOC_Os02g47400.1* | 1194 | 44 | 13 | 1.949864768 | 0.611113221 | -1.673862473 | 7.72E-05 | 0.000508825 |
| *LOC_Os01g71350.1* | 999 | 43 | 13 | 2.277503798 | 0.730399585 | -1.640695609 | 0.000117304 | 0.000742267 |
| *LOC_Os08g30120.1* | 2904 | 46 | 14 | 0.838141533 | 0.270591455 | -1.631077606 | 7.17E-05 | 0.000476062 |
| *LOC_Os09g01310.1* | 1056 | 75 | 23 | 3.757971548 | 1.222493536 | -1.620127307 | 4.00E-07 | 3.85E-06 |
| *LOC_Os03g14140.1* | 756 | 179 | 55 | 12.5281625 | 4.083415262 | -1.617326636 | 4.01E-15 | 8.19E-14 |
| *LOC_Os02g52730.1* | 1998 | 122 | 38 | 3.23087748 | 1.067507086 | -1.597680396 | 1.38E-10 | 1.95E-09 |
| *LOC_Os11g04060.1* | 864 | 80 | 25 | 4.899281425 | 1.624085616 | -1.592942477 | 2.29E-07 | 2.27E-06 |
| *LOC_Os03g63330.1* | 1698 | 3030 | 956 | 94.41936711 | 31.60114801 | -1.579105843 | 1.11E-222 | 5.19E-220 |
| *LOC_Os11g27440.1* | 3756 | 57 | 18 | 0.802981269 | 0.268985937 | -1.577835585 | 1.52E-05 | 0.000113918 |
| *LOC_Os07g13490.1* | 663 | 60 | 19 | 4.788437954 | 1.608506152 | -1.573833655 | 9.34E-06 | 7.25E-05 |
| *LOC_Os06g08580.1* | 1338 | 395 | 126 | 15.62057889 | 5.285633975 | -1.563299492 | 4.00E-30 | 1.65E-28 |
| *LOC_Os01g42110.1* | 780 | 50 | 16 | 3.391810217 | 1.151351772 | -1.558726762 | 6.07E-05 | 0.000408025 |
| *LOC_Os01g71380.1* | 1005 | 908 | 291 | 47.80528693 | 16.25210356 | -1.556543717 | 1.14E-66 | 1.16E-64 |
| *LOC_Os11g39209.1* | 261 | 106 | 34 | 21.48926197 | 7.311745447 | -1.555328186 | 4.54E-09 | 5.49E-08 |
| *LOC_Os07g28610.1* | 456 | 190 | 61 | 22.04676641 | 7.508404236 | -1.553988843 | 3.75E-15 | 7.67E-14 |
| *LOC_Os06g43320.1* | 1581 | 77 | 25 | 2.577003436 | 0.887545839 | -1.537800923 | 7.55E-07 | 6.97E-06 |
| *LOC_Os08g06010.1* | 1500 | 2675 | 871 | 94.36016024 | 32.59189028 | -1.53366484 | 1.07E-188 | 3.79E-186 |
| *LOC_Os11g47890.1* | 1917 | 46 | 15 | 1.269672933 | 0.43918935 | -1.531541933 | 0.000148281 | 0.000919057 |
| *LOC_Os08g13440.2* | 675 | 655 | 214 | 51.34446933 | 17.79478127 | -1.528754682 | 2.34E-47 | 1.63E-45 |
| *LOC_Os09g11890.1* | 822 | 52 | 17 | 3.347246287 | 1.160806303 | -1.527847449 | 5.53E-05 | 0.000374409 |
| *LOC_Os03g15320.1* | 1695 | 171 | 56 | 5.33804893 | 1.854389579 | -1.525368165 | 1.89E-13 | 3.44E-12 |
| *LOC_Os02g55190.1* | 1047 | 137 | 45 | 6.923569051 | 2.412395367 | -1.521049559 | 5.18E-11 | 7.63E-10 |
| *LOC_Os05g46370.1* | 1251 | 146 | 48 | 6.175209393 | 2.153607631 | -1.519732631 | 1.24E-11 | 1.95E-10 |
| *LOC_Os09g28880.1* | 735 | 356 | 118 | 25.62824112 | 9.011089888 | -1.507960954 | 5.51E-26 | 1.95E-24 |
| *LOC_Os03g19670.1* | 1104 | 252 | 84 | 12.07779377 | 4.270639045 | -1.499833073 | 1.06E-18 | 2.65E-17 |
| *LOC_Os06g07040.1* | 552 | 48 | 16 | 4.601064295 | 1.626910112 | -1.499833073 | 0.000134339 | 0.000840594 |
| *LOC_Os04g08450.3* | 669 | 63 | 21 | 4.982766938 | 1.761877992 | -1.499833073 | 1.15E-05 | 8.82E-05 |
| *LOC_Os11g13750.1* | 462 | 607 | 203 | 69.51889461 | 24.66247829 | -1.495087361 | 1.20E-42 | 7.49E-41 |
| *LOC_Os06g11730.1* | 1032 | 143 | 48 | 7.331831621 | 2.610623204 | -1.489779408 | 3.92E-11 | 5.86E-10 |
| *LOC_Os10g19960.1* | 2487 | 175 | 59 | 3.723217488 | 1.331554296 | -1.483438635 | 3.08E-13 | 5.51E-12 |
| *LOC_Os08g42390.1* | 1164 | 222 | 75 | 10.09150957 | 3.616520546 | -1.480467748 | 2.31E-16 | 5.11E-15 |
| *LOC_Os01g74140.1* | 1233 | 145 | 49 | 6.222445021 | 2.230568974 | -1.480069818 | 3.56E-11 | 5.33E-10 |
| *LOC_Os12g29710.1* | 4575 | 281 | 95 | 3.249910223 | 1.165507736 | -1.479441284 | 2.68E-20 | 7.36E-19 |
| *LOC_Os03g03860.1* | 2742 | 53 | 18 | 1.022738398 | 0.368457761 | -1.472866025 | 7.40E-05 | 0.000488963 |
| *LOC_Os03g28170.1* | 237 | 100 | 34 | 22.3258394 | 8.052175366 | -1.471263921 | 4.62E-08 | 5.02E-07 |
| *LOC_Os09g26670.1* | 696 | 150 | 51 | 11.40349987 | 4.112856814 | -1.471263921 | 2.01E-11 | 3.08E-10 |
| *LOC_Os06g43220.1* | 1005 | 182 | 62 | 9.582116984 | 3.462647493 | -1.468468902 | 1.58E-13 | 2.89E-12 |
| *LOC_Os10g20890.1* | 381 | 73 | 25 | 10.13804062 | 3.682965806 | -1.460838941 | 3.56E-06 | 2.96E-05 |
| *LOC_Os03g56820.1* | 753 | 67 | 23 | 4.70799474 | 1.714413246 | -1.457397807 | 9.41E-06 | 7.30E-05 |
| *LOC_Os12g06150.1* | 1038 | 145 | 50 | 7.391401456 | 2.7036801 | -1.450923473 | 6.85E-11 | 9.99E-10 |
| *LOC_Os08g19420.1* | 1128 | 145 | 50 | 6.801661978 | 2.487960943 | -1.450923473 | 6.85E-11 | 9.99E-10 |
| *LOC_Os01g20730.1* | 513 | 66 | 23 | 6.807422611 | 2.516477922 | -1.435702736 | 1.38E-05 | 0.000103875 |
| *LOC_Os05g14580.1* | 552 | 60 | 21 | 5.751330368 | 2.135319522 | -1.429443745 | 3.65E-05 | 0.000255924 |
| *LOC_Os01g52240.1* | 798 | 67428 | 23625 | 4470.88531 | 1661.696019 | -1.427904049 | 0 | 0 |
| *LOC_Os11g11880.1* | 1623 | 154 | 54 | 5.020631464 | 1.867488318 | -1.426769611 | 3.12E-11 | 4.69E-10 |
| *LOC_Os06g09600.1* | 624 | 88 | 31 | 7.461982478 | 2.788430072 | -1.420105881 | 6.06E-07 | 5.66E-06 |
| *LOC_Os12g36410.1* | 1341 | 172 | 61 | 6.786655611 | 2.553193387 | -1.41039799 | 3.41E-12 | 5.62E-11 |
| *LOC_Os03g49600.1* | 1515 | 124 | 44 | 4.330770749 | 1.630131717 | -1.409635264 | 3.64E-09 | 4.46E-08 |
| *LOC_Os08g14070.1* | 273 | 56 | 20 | 10.8537927 | 4.111970614 | -1.4002974 | 8.68E-05 | 0.000565821 |
| *LOC_Os11g04480.1* | 927 | 1491 | 533 | 85.10479928 | 32.27231564 | -1.398943392 | 1.08E-92 | 1.68E-90 |
| *LOC_Os10g04800.1* | 156 | 106 | 38 | 35.9531883 | 13.67230229 | -1.394863513 | 6.38E-08 | 6.80E-07 |
| *LOC_Os04g09654.1* | 1116 | 295 | 106 | 13.98665826 | 5.331192008 | -1.391521262 | 1.65E-19 | 4.34E-18 |
| *LOC_Os09g23300.1* | 741 | 1100 | 396 | 78.54718398 | 29.99574353 | -1.388801761 | 3.96E-68 | 4.11E-66 |
| *LOC_Os12g38150.1* | 702 | 308 | 111 | 23.2150566 | 8.875003241 | -1.387241247 | 3.26E-20 | 8.88E-19 |
| *LOC_Os11g04894.1* | 441 | 152 | 55 | 18.23732514 | 7.000140449 | -1.381438372 | 1.21E-10 | 1.72E-09 |
| *LOC_Os07g48020.1* | 954 | 3345 | 1211 | 185.5256192 | 71.24894239 | -1.380677918 | 1.54E-201 | 5.87E-199 |
| *LOC_Os11g24630.1* | 666 | 334 | 121 | 26.5355675 | 10.1975019 | -1.379711628 | 1.26E-21 | 3.71E-20 |
| *LOC_Os11g37970.1* | 441 | 1246 | 454 | 149.4980732 | 57.78297753 | -1.371410438 | 1.65E-75 | 1.94E-73 |
| *LOC_Os03g16790.1* | 948 | 85 | 31 | 4.744240874 | 1.835422326 | -1.370065198 | 1.83E-06 | 1.59E-05 |
| *LOC_Os02g13660.1* | 660 | 7526 | 2774 | 603.3598692 | 235.9093613 | -1.354786074 | 0 | 0 |
| *LOC_Os07g08680.1* | 366 | 230 | 85 | 33.25086082 | 13.03528389 | -1.350969687 | 6.42E-15 | 1.29E-13 |
| *LOC_Os05g09724.1* | 753 | 3525 | 1306 | 247.6967382 | 97.34885648 | -1.347338933 | 1.40E-204 | 5.45E-202 |
| *LOC_Os01g71670.1* | 1005 | 1695 | 631 | 89.24004554 | 35.24081561 | -1.340443935 | 1.28E-98 | 2.11E-96 |
| *LOC_Os04g55600.1* | 333 | 118 | 44 | 18.74968243 | 7.416365017 | -1.338082003 | 3.19E-08 | 3.51E-07 |
| *LOC_Os08g33660.1* | 1074 | 1654 | 619 | 81.48681932 | 32.34960792 | -1.332818492 | 1.87E-95 | 2.97E-93 |
| *LOC_Os12g04880.1* | 1950 | 237 | 89 | 6.430872172 | 2.561757692 | -1.32788039 | 5.73E-15 | 1.16E-13 |
| *LOC_Os01g68598.2* | 366 | 101 | 38 | 14.60146497 | 5.827538681 | -1.325154542 | 3.87E-07 | 3.73E-06 |
| *LOC_Os09g29710.1* | 405 | 687 | 259 | 89.7548357 | 35.89445755 | -1.322228573 | 3.35E-40 | 1.91E-38 |
| *LOC_Os03g13030.1* | 1305 | 98 | 37 | 3.973486176 | 1.591379892 | -1.320127051 | 6.23E-07 | 5.82E-06 |
| *LOC_Os02g24205.1* | 462 | 90 | 34 | 10.3075791 | 4.130661389 | -1.319260827 | 1.83E-06 | 1.59E-05 |
| *LOC_Os06g10590.1* | 252 | 79 | 30 | 16.58756711 | 6.681952247 | -1.311760725 | 8.70E-06 | 6.80E-05 |
| *LOC_Os04g34600.1* | 690 | 1994 | 760 | 152.9087034 | 61.82258427 | -1.306464658 | 2.84E-111 | 5.34E-109 |
| *LOC_Os09g07440.1* | 2439 | 225 | 86 | 4.881202896 | 1.979107135 | -1.302387009 | 6.59E-14 | 1.24E-12 |
| *LOC_Os05g40285.1* | 558 | 68 | 26 | 6.448086521 | 2.61530174 | -1.301893695 | 4.17E-05 | 0.000289176 |
| *LOC_Os08g09040.1* | 675 | 81 | 31 | 6.349468727 | 2.577748689 | -1.300524265 | 7.67E-06 | 6.05E-05 |
| *LOC_Os11g05740.1* | 840 | 133 | 51 | 8.377771237 | 3.407795646 | -1.297727666 | 9.58E-09 | 1.13E-07 |
| *LOC_Os05g42340.1* | 915 | 65 | 25 | 3.758792962 | 1.533562811 | -1.293382196 | 6.76E-05 | 0.000450524 |
| *LOC_Os07g14660.1* | 618 | 98 | 38 | 8.390614013 | 3.451260772 | -1.281652903 | 1.11E-06 | 9.98E-06 |
| *LOC_Os02g09740.1* | 2157 | 134 | 52 | 3.287083949 | 1.353118564 | -1.280520045 | 1.20E-08 | 1.39E-07 |
| *LOC_Os05g02810.1* | 1224 | 152 | 59 | 6.57080097 | 2.705535567 | -1.280155036 | 1.26E-09 | 1.62E-08 |
| *LOC_Os04g14540.1* | 2298 | 278 | 108 | 6.401045496 | 2.637888198 | -1.278924143 | 2.05E-16 | 4.55E-15 |
| *LOC_Os02g10780.1* | 843 | 1265 | 492 | 79.39974238 | 32.75821144 | -1.277277737 | 7.03E-69 | 7.38E-67 |
| *LOC_Os06g29730.1* | 249 | 321 | 125 | 68.21216403 | 28.17690707 | -1.275515775 | 1.19E-18 | 2.98E-17 |
| *LOC_Os04g42830.1* | 1350 | 77 | 30 | 3.017957358 | 1.247297753 | -1.274766517 | 1.76E-05 | 0.000130045 |
| *LOC_Os07g23640.1* | 744 | 6134 | 2397 | 436.2415006 | 180.833027 | -1.27046883 | 0 | 0 |
| *LOC_Os11g11110.1* | 381 | 115 | 45 | 15.9708859 | 6.62933845 | -1.268507527 | 1.62E-07 | 1.62E-06 |
| *LOC_Os09g37369.1* | 426 | 69 | 27 | 8.570292295 | 3.557433732 | -1.268507527 | 5.21E-05 | 0.000354499 |
| *LOC_Os11g34820.1* | 252 | 69 | 27 | 14.48787507 | 6.013757022 | -1.268507527 | 5.21E-05 | 0.000354431 |
| *LOC_Os05g12410.1* | 720 | 7197 | 2835 | 528.9019262 | 221.0055706 | -1.258917496 | 0 | 0 |
| *LOC_Os06g16640.1* | 1230 | 12969 | 5110 | 557.90149 | 233.183836 | -1.258542618 | 0 | 0 |
| *LOC_Os10g37520.1* | 1371 | 129 | 51 | 4.978613334 | 2.08792731 | -1.253672486 | 3.78E-08 | 4.14E-07 |
| *LOC_Os09g36700.1* | 780 | 328 | 130 | 22.25027503 | 9.354733146 | -1.250054764 | 1.75E-18 | 4.33E-17 |
| *LOC_Os05g38740.1* | 312 | 116 | 46 | 19.67249926 | 8.27534086 | -1.249289611 | 2.00E-07 | 1.99E-06 |
| *LOC_Os02g06230.1* | 4362 | 68 | 27 | 0.824858386 | 0.347424752 | -1.247445911 | 7.37E-05 | 0.000487202 |
| *LOC_Os09g15820.1* | 960 | 1566 | 624 | 86.3130905 | 36.48345927 | -1.242336851 | 2.55E-81 | 3.29E-79 |
| *LOC_Os04g52440.1* | 1494 | 125 | 50 | 4.427061529 | 1.878460471 | -1.236798667 | 8.46E-08 | 8.86E-07 |
| *LOC_Os01g04920.1* | 1545 | 1685 | 676 | 57.70687597 | 24.55844507 | -1.232524012 | 2.65E-86 | 3.72E-84 |
| *LOC_Os03g04060.1* | 771 | 124 | 50 | 8.509880265 | 3.639973987 | -1.225210693 | 1.18E-07 | 1.21E-06 |
| *LOC_Os08g10320.2* | 3000 | 104 | 42 | 1.834290965 | 0.785797584 | -1.222992868 | 1.30E-06 | 1.16E-05 |
| *LOC_Os05g37830.1* | 501 | 89 | 36 | 9.399579452 | 4.033178362 | -1.220679002 | 7.95E-06 | 6.25E-05 |
| *LOC_Os04g55100.1* | 450 | 84 | 34 | 9.876951353 | 4.24081236 | -1.219725154 | 1.46E-05 | 0.000109461 |
| *LOC_Os08g20410.1* | 669 | 126 | 51 | 9.965533876 | 4.278846551 | -1.219725154 | 1.04E-07 | 1.07E-06 |
| *LOC_Os11g13890.2* | 738 | 252 | 102 | 18.06759394 | 7.757583585 | -1.219725154 | 4.73E-14 | 8.98E-13 |
| *LOC_Os03g60840.1* | 384 | 165 | 67 | 22.73572786 | 9.793236262 | -1.215103596 | 1.23E-09 | 1.59E-08 |
| *LOC_Os12g42020.1* | 1455 | 293 | 119 | 10.65517948 | 4.59057008 | -1.214809663 | 5.24E-16 | 1.13E-14 |
| *LOC_Os11g02240.1* | 1305 | 224 | 91 | 9.082254117 | 3.913934328 | -1.214430854 | 1.42E-12 | 2.41E-11 |
| *LOC_Os11g12330.1* | 2718 | 214 | 87 | 4.16601149 | 1.796604379 | -1.213394063 | 4.63E-12 | 7.56E-11 |
| *LOC_Os07g44030.1* | 1167 | 76 | 31 | 3.445869917 | 1.490985746 | -1.208601775 | 4.29E-05 | 0.00029636 |
| *LOC_Os02g40440.1* | 1104 | 1097 | 449 | 52.57674512 | 22.82758251 | -1.203646748 | 8.47E-55 | 6.82E-53 |
| *LOC_Os03g22020.1* | 981 | 239 | 98 | 12.89095333 | 5.607118338 | -1.201027536 | 4.10E-13 | 7.25E-12 |
| *LOC_Os09g31430.1* | 1503 | 2035 | 835 | 71.64098946 | 31.18244382 | -1.200051264 | 1.22E-99 | 2.01E-97 |
| *LOC_Os03g43580.1* | 1344 | 141 | 58 | 5.551060829 | 2.42220769 | -1.19644093 | 2.93E-08 | 3.25E-07 |
| *LOC_Os09g16090.1* | 1203 | 362 | 149 | 15.92205375 | 6.951896453 | -1.195547939 | 5.60E-19 | 1.43E-17 |
| *LOC_Os01g65830.1* | 1146 | 68 | 28 | 3.139644222 | 1.371374493 | -1.194978492 | 0.000126904 | 0.000798019 |
| *LOC_Os05g46460.3* | 1134 | 102 | 42 | 4.759301956 | 2.078829588 | -1.194978492 | 2.53E-06 | 2.16E-05 |
| *LOC_Os09g12290.1* | 2748 | 4160 | 1713 | 80.10004216 | 34.98833598 | -1.194928949 | 2.66E-200 | 1.00E-197 |
| *LOC_Os01g56260.1* | 1578 | 75 | 31 | 2.514840275 | 1.102649154 | -1.189492952 | 6.00E-05 | 0.000403769 |
| *LOC_Os07g23660.1* | 744 | 1101 | 456 | 78.30158006 | 34.40127673 | -1.186579312 | 8.39E-54 | 6.69E-52 |
| *LOC_Os06g40120.1* | 888 | 1846 | 765 | 109.9954886 | 48.35385714 | -1.185741472 | 6.83E-89 | 1.01E-86 |
| *LOC_Os01g59840.1* | 1545 | 433 | 180 | 14.82912599 | 6.539230937 | -1.181240691 | 5.25E-22 | 1.57E-20 |
| *LOC_Os12g03870.1* | 1398 | 101 | 42 | 3.822701129 | 1.68626091 | -1.180764632 | 3.52E-06 | 2.93E-05 |
| *LOC_Os06g07790.1* | 2814 | 388 | 162 | 7.295646369 | 3.23127243 | -1.174933412 | 1.01E-19 | 2.67E-18 |
| *LOC_Os06g50930.1* | 513 | 98 | 41 | 10.10799115 | 4.485895427 | -1.172028412 | 5.60E-06 | 4.50E-05 |
| *LOC_Os04g08390.1* | 3045 | 662 | 277 | 11.50341625 | 5.105933165 | -1.171815813 | 2.08E-32 | 9.34E-31 |
| *LOC_Os03g17260.1* | 1227 | 191 | 80 | 8.236542562 | 3.659553309 | -1.170371306 | 2.18E-10 | 3.02E-09 |
| *LOC_Os03g47190.1* | 645 | 88 | 37 | 7.219034211 | 3.219768618 | -1.164848825 | 1.87E-05 | 0.000138287 |
| *LOC_Os01g73110.1* | 993 | 114 | 48 | 6.074516909 | 2.713155233 | -1.162798086 | 1.11E-06 | 1.00E-05 |
| *LOC_Os11g06980.1* | 1887 | 5597 | 2362 | 156.9421324 | 70.25716913 | -1.159515355 | 2.53E-256 | 1.44E-253 |
| *LOC_Os02g52870.1* | 987 | 71 | 30 | 3.80625025 | 1.706030361 | -1.157727096 | 0.000133439 | 0.000835407 |
| *LOC_Os12g29690.1* | 4515 | 139 | 59 | 1.628970382 | 0.733460805 | -1.151168596 | 9.33E-08 | 9.71E-07 |
| *LOC_Os09g39750.1* | 1560 | 219 | 93 | 7.428064376 | 3.346116087 | -1.150498821 | 1.98E-11 | 3.03E-10 |
| *LOC_Os09g07450.1* | 609 | 313 | 133 | 27.19463207 | 12.25792619 | -1.149606984 | 1.06E-15 | 2.24E-14 |
| *LOC_Os10g35840.1* | 1614 | 12662 | 5381 | 415.1020912 | 187.1294389 | -1.149429661 | 0 | 0 |
| *LOC_Os06g35630.1* | 1587 | 677 | 288 | 22.57188788 | 10.18587201 | -1.147957594 | 4.32E-32 | 1.92E-30 |
| *LOC_Os01g59150.1* | 1344 | 192 | 82 | 7.558891341 | 3.424500527 | -1.142281068 | 4.30E-10 | 5.77E-09 |
| *LOC_Os05g38390.1* | 1542 | 91 | 39 | 3.122577033 | 1.419589855 | -1.137262994 | 1.95E-05 | 0.000143633 |
| *LOC_Os01g07310.1* | 1071 | 84 | 36 | 4.14997956 | 1.88666887 | -1.137262994 | 4.12E-05 | 0.000285673 |
| *LOC_Os01g46140.1* | 414 | 420 | 180 | 53.67908344 | 24.40365169 | -1.137262994 | 3.15E-20 | 8.59E-19 |
| *LOC_Os02g24700.1* | 291 | 114 | 49 | 20.72850615 | 9.451173694 | -1.133050742 | 1.84E-06 | 1.60E-05 |
| *LOC_Os12g03070.1* | 837 | 123 | 53 | 7.775633745 | 3.554128005 | -1.129464623 | 7.64E-07 | 7.04E-06 |
| *LOC_Os02g50740.1* | 1287 | 104 | 45 | 4.275736516 | 1.962531429 | -1.123457194 | 6.08E-06 | 4.86E-05 |
| *LOC_Os05g30700.1* | 1122 | 208 | 90 | 9.809042596 | 4.502277985 | -1.123457194 | 1.43E-10 | 2.02E-09 |
| *LOC_Os03g01300.1* | 420 | 3280 | 1422 | 413.2193933 | 190.0347219 | -1.120644922 | 3.96E-143 | 9.86E-141 |
| *LOC_Os09g28470.1* | 1533 | 108 | 47 | 3.727672442 | 1.720831538 | -1.115169223 | 4.58E-06 | 3.74E-05 |
| *LOC_Os01g14670.1* | 684 | 2573 | 1121 | 199.0397543 | 91.98820927 | -1.113535751 | 1.44E-111 | 2.73E-109 |
| *LOC_Os08g39460.1* | 1272 | 78 | 34 | 3.244618453 | 1.500287391 | -1.11280995 | 0.000103825 | 0.000664943 |
| *LOC_Os06g47000.2* | 1767 | 78 | 34 | 2.335684591 | 1.080003148 | -1.11280995 | 0.000103825 | 0.000664823 |
| *LOC_Os04g58200.1* | 1164 | 1749 | 764 | 79.50473084 | 36.8402893 | -1.109756318 | 4.97E-76 | 5.84E-74 |
| *LOC_Os03g02740.1* | 2715 | 215 | 94 | 4.190103672 | 1.943303681 | -1.10847457 | 1.12E-10 | 1.60E-09 |
| *LOC_Os12g07160.2* | 2997 | 96 | 42 | 1.694886547 | 0.786584168 | -1.10751565 | 1.75E-05 | 0.000129472 |
| *LOC_Os09g37480.1* | 435 | 128 | 56 | 15.56957849 | 7.225724913 | -1.10751565 | 6.85E-07 | 6.35E-06 |
| *LOC_Os09g31350.1* | 1371 | 137 | 60 | 5.287364549 | 2.456385071 | -1.10601206 | 2.85E-07 | 2.80E-06 |
| *LOC_Os02g37700.1* | 1308 | 89 | 39 | 3.600297634 | 1.673553178 | -1.105201784 | 3.68E-05 | 0.000257945 |
| *LOC_Os06g47800.1* | 1563 | 1404 | 616 | 47.52961235 | 22.12098126 | -1.103411252 | 7.68E-61 | 7.01E-59 |
| *LOC_Os03g14840.1* | 1527 | 189 | 83 | 6.549059099 | 3.050855997 | -1.102073565 | 1.77E-09 | 2.24E-08 |
| *LOC_Os01g02060.2* | 1353 | 166 | 73 | 6.491819467 | 3.028361506 | -1.100085445 | 1.81E-08 | 2.06E-07 |
| *LOC_Os04g30800.2* | 648 | 227 | 100 | 18.53561472 | 8.66178995 | -1.09756287 | 4.83E-11 | 7.14E-10 |
| *LOC_Os05g47700.1* | 285 | 304 | 134 | 56.43972202 | 26.39019456 | -1.096708895 | 2.75E-14 | 5.31E-13 |
| *LOC_Os05g27350.1* | 279 | 102 | 45 | 19.34425956 | 9.052967561 | -1.095442818 | 1.14E-05 | 8.71E-05 |
| *LOC_Os05g42360.1* | 420 | 120 | 53 | 15.11778268 | 7.082869382 | -1.093840713 | 1.95E-06 | 1.69E-05 |
| *LOC_Os11g34840.1* | 996 | 86 | 38 | 4.568727497 | 2.141444937 | -1.093207814 | 5.82E-05 | 0.000392451 |
| *LOC_Os10g33550.1* | 534 | 131 | 58 | 12.98034337 | 6.096342949 | -1.090312579 | 7.02E-07 | 6.51E-06 |
| *LOC_Os06g51060.1* | 1050 | 1153 | 511 | 58.10267811 | 27.31582079 | -1.088867889 | 3.00E-49 | 2.18E-47 |
| *LOC_Os01g54470.1* | 1236 | 216 | 96 | 9.246799117 | 4.359487291 | -1.084795574 | 2.10E-10 | 2.92E-09 |
| *LOC_Os03g42600.1* | 318 | 182 | 81 | 30.28310556 | 14.29685632 | -1.08281521 | 5.85E-09 | 7.01E-08 |
| *LOC_Os07g03120.1* | 618 | 137 | 61 | 11.72973592 | 5.540181766 | -1.082165318 | 4.58E-07 | 4.36E-06 |
| *LOC_Os05g51820.1* | 684 | 101 | 45 | 7.813064588 | 3.692657821 | -1.081228959 | 1.55E-05 | 0.0001158 |
| *LOC_Os06g46754.2* | 477 | 400 | 179 | 44.37085064 | 21.06285828 | -1.074910985 | 8.60E-18 | 2.06E-16 |
| *LOC_Os06g03080.1* | 1329 | 96 | 43 | 3.822103071 | 1.816043004 | -1.073568318 | 2.81E-05 | 0.000201812 |
| *LOC_Os04g46079.1* | 801 | 214 | 96 | 14.13635359 | 6.726999116 | -1.071375058 | 3.84E-10 | 5.19E-09 |
| *LOC_Os06g48170.1* | 900 | 122 | 55 | 7.172548006 | 3.43006882 | -1.064248196 | 2.69E-06 | 2.28E-05 |
| *LOC_Os05g37980.1* | 594 | 82 | 37 | 7.304383215 | 3.496213398 | -1.062969211 | 0.000125017 | 0.000787555 |
| *LOC_Os06g03030.1* | 636 | 82 | 37 | 6.822018286 | 3.265331381 | -1.062969211 | 0.000125017 | 0.000787695 |
| *LOC_Os09g19650.1* | 1449 | 1249 | 564 | 45.60896273 | 21.84707865 | -1.061876982 | 3.77E-51 | 2.86E-49 |
| *LOC_Os02g02660.1* | 6189 | 321 | 145 | 2.744357545 | 1.315013385 | -1.061390969 | 2.64E-14 | 5.10E-13 |
| *LOC_Os11g13930.1* | 1254 | 190 | 86 | 8.017005968 | 3.849316031 | -1.058461426 | 5.24E-09 | 6.30E-08 |
| *LOC_Os12g02380.1* | 690 | 353 | 160 | 27.06959493 | 13.0152809 | -1.056466851 | 1.77E-15 | 3.68E-14 |
| *LOC_Os03g02190.1* | 1032 | 1095 | 497 | 56.14234703 | 27.03082775 | -1.054483685 | 1.44E-44 | 9.47E-43 |
| *LOC_Os10g39680.1* | 786 | 647 | 295 | 43.55498586 | 21.06600212 | -1.04792133 | 9.38E-27 | 3.43E-25 |
| *LOC_Os09g01000.1* | 297 | 4685 | 2138 | 834.6593991 | 404.0488781 | -1.046657767 | 1.91E-182 | 6.57E-180 |
| *LOC_Os12g12990.1* | 2376 | 276 | 126 | 6.146371242 | 2.976506001 | -1.046115106 | 3.03E-12 | 5.01E-11 |
| *LOC_Os11g11890.1* | 2178 | 376 | 172 | 9.134528012 | 4.432545733 | -1.043194669 | 4.36E-16 | 9.51E-15 |
| *LOC_Os07g30690.1* | 1473 | 365 | 167 | 13.11131526 | 6.363504828 | -1.042918934 | 1.18E-15 | 2.49E-14 |
| *LOC_Os03g04530.1* | 1569 | 2266 | 1040 | 76.41754905 | 37.20429244 | -1.038434905 | 3.99E-88 | 5.73E-86 |
| *LOC_Os03g09930.1* | 1971 | 4035 | 1854 | 108.3210989 | 52.79657611 | -1.036798002 | 5.05E-155 | 1.36E-152 |
| *LOC_Os08g10304.1* | 240 | 176 | 81 | 38.80230889 | 18.94333462 | -1.034452188 | 3.50E-08 | 3.84E-07 |
| *LOC_Os02g41580.1* | 918 | 1751 | 806 | 100.9251974 | 49.28048965 | -1.034197912 | 4.53E-68 | 4.68E-66 |
| *LOC_Os01g36720.1* | 1458 | 419 | 193 | 15.2059179 | 7.429890935 | -1.033219969 | 1.68E-17 | 3.97E-16 |
| *LOC_Os07g09760.1* | 4944 | 258 | 119 | 2.761196958 | 1.350986947 | -1.031280064 | 2.63E-11 | 3.99E-10 |
| *LOC_Os03g08540.1* | 897 | 104 | 48 | 6.134752393 | 3.003526361 | -1.03034779 | 2.48E-05 | 0.000179545 |
| *LOC_Os05g02900.1* | 663 | 130 | 60 | 10.3749489 | 5.079493111 | -1.03034779 | 2.37E-06 | 2.02E-05 |
| *LOC_Os08g20660.2* | 3201 | 164 | 76 | 2.710905111 | 1.332633025 | -1.024495064 | 1.29E-07 | 1.32E-06 |
| *LOC_Os01g46169.1* | 888 | 123 | 57 | 7.329060186 | 3.602836414 | -1.024495064 | 4.92E-06 | 4.00E-05 |
| *LOC_Os10g08550.3* | 1437 | 123 | 57 | 4.529022578 | 2.226387429 | -1.024495064 | 4.92E-06 | 4.01E-05 |
| *LOC_Os06g49390.1* | 4437 | 110 | 51 | 1.311775148 | 0.64515401 | -1.023804944 | 1.59E-05 | 0.000118867 |
| *LOC_Os08g24000.1* | 867 | 138 | 64 | 8.422017342 | 4.143272812 | -1.023395029 | 1.32E-06 | 1.18E-05 |
| *LOC_Os07g40020.1* | 1422 | 159 | 74 | 5.916347442 | 2.920887143 | -1.018300162 | 2.31E-07 | 2.29E-06 |
| *LOC_Os09g08720.1* | 975 | 762 | 355 | 41.35295017 | 20.43649395 | -1.016842545 | 8.48E-30 | 3.47E-28 |
| *LOC_Os01g67190.1* | 837 | 354 | 165 | 22.37865322 | 11.06473813 | -1.016153908 | 1.18E-14 | 2.35E-13 |
| *LOC_Os03g50130.2* | 303 | 92 | 43 | 16.06576245 | 7.965416342 | -1.012167774 | 9.37E-05 | 0.000606317 |
| *LOC_Os04g58080.1* | 1605 | 143 | 67 | 4.71429921 | 2.343054657 | -1.008652719 | 1.14E-06 | 1.02E-05 |
| *LOC_Os07g13660.1* | 1518 | 147 | 69 | 5.12391251 | 2.551290858 | -1.00601846 | 8.53E-07 | 7.80E-06 |
| *LOC_Os10g27280.1* | 828 | 196 | 92 | 12.52511947 | 6.236488764 | -1.00601846 | 1.28E-08 | 1.48E-07 |
| *LOC_Os05g22614.1* | 1488 | 98 | 46 | 3.484811465 | 1.735152116 | -1.00601846 | 6.00E-05 | 0.000404043 |
| *LOC_Os10g40710.1* | 786 | 1420 | 667 | 95.59208643 | 47.63058785 | -1.005002836 | 4.53E-53 | 3.57E-51 |
| *LOC_Os01g55150.1* | 798 | 898 | 423 | 59.54284583 | 29.75227158 | -1.000928354 | 5.68E-34 | 2.69E-32 |
| *LOC_Os11g44310.1* | 1629 | 104 | 49 | 3.378068076 | 1.688331212 | -1.000600446 | 3.85E-05 | 0.000268456 |
