# Supplementary material for: Transcriptome analysis of phosphorus stress responsiveness in the seedlings of Dongxiang wild rice (Oryza rufipogon Griff.)
Source: Biol Res. 2018 Mar 15;51:7. doi: 10.1186/s40659-018-0155-x (PMC5853122; doi:10.1186/s40659-018-0155-x)
Supplement: Supplementary file 4 — Additional file 4: Table S3. List of down-regulated genes in LLP vs. LCK. [file 40659_2018_155_MOESM4_ESM.docx]

| **Table S3** List of down-regulated genes in LLP vs. LCK. | | | | | | | | |
| --- | --- | --- | --- | --- | --- | --- | --- | --- |
| Gene ID | Gene  Length | LLP-  Expression | LCK-  Expression | LLP-RPKM | LCK-RPKM | Log_2_ Ratio  (LCK/LLP) | *P*-value | FDR |
| *LOC_Os10g11889.1* | 180 | 0 | 92 | 0.001 | 28.68784831 | 14.80815214 | 1.25E-29 | 5.07E-28 |
| *LOC_Os05g33560.1* | 645 | 0 | 280 | 0.001 | 24.36581657 | 14.57257095 | 1.14E-88 | 1.67E-86 |
| *LOC_Os01g05530.1* | 1881 | 0 | 701 | 0.001 | 20.91760107 | 14.35242979 | 7.09E-221 | 3.22E-218 |
| *LOC_Os06g31800.1* | 408 | 0 | 125 | 0.001 | 17.19620064 | 14.06980223 | 5.41E-40 | 3.07E-38 |
| *LOC_Os11g15624.1* | 390 | 0 | 83 | 0.001 | 11.94527463 | 13.5441524 | 8.36E-27 | 3.06E-25 |
| *LOC_Os01g05540.1* | 1200 | 0 | 233 | 0.001 | 10.89826412 | 13.41181074 | 6.57E-74 | 7.50E-72 |
| *LOC_Os04g24770.1* | 402 | 0 | 69 | 0.001 | 9.633978912 | 13.23391605 | 2.08E-22 | 6.36E-21 |
| *LOC_Os11g20160.1* | 1107 | 0 | 165 | 0.001 | 8.366021513 | 13.03032599 | 1.48E-52 | 1.16E-50 |
| *LOC_Os04g11120.1* | 327 | 0 | 48 | 0.001 | 8.239031028 | 13.00825896 | 8.19E-16 | 1.75E-14 |
| *LOC_Os03g15340.1* | 363 | 0 | 50 | 0.001 | 7.731184418 | 12.91647374 | 1.93E-16 | 4.29E-15 |
| *LOC_Os08g40720.1* | 1527 | 0 | 177 | 0.001 | 6.506042306 | 12.66756449 | 2.53E-56 | 2.11E-54 |
| *LOC_Os01g06310.1* | 375 | 0 | 41 | 0.001 | 6.136704944 | 12.5832485 | 1.29E-13 | 2.38E-12 |
| *LOC_Os12g12600.1* | 465 | 0 | 38 | 0.001 | 4.586836897 | 12.16328389 | 1.13E-12 | 1.93E-11 |
| *LOC_Os07g27030.1* | 846 | 0 | 67 | 0.001 | 4.445156885 | 12.11801862 | 8.85E-22 | 2.62E-20 |
| *LOC_Os07g03770.1* | 1068 | 0 | 81 | 0.001 | 4.256929128 | 12.05559736 | 3.55E-26 | 1.27E-24 |
| *LOC_Os08g20480.1* | 3312 | 0 | 206 | 0.001 | 3.491077949 | 11.76945685 | 1.98E-65 | 1.98E-63 |
| *LOC_Os12g36240.1* | 258 | 0 | 15 | 0.001 | 3.263279004 | 11.67210663 | 1.89E-05 | 0.000139398 |
| *LOC_Os10g31540.1* | 582 | 0 | 33 | 0.001 | 3.182538081 | 11.63596206 | 4.21E-11 | 6.26E-10 |
| *LOC_Os01g72360.1* | 657 | 0 | 37 | 0.001 | 3.160960058 | 11.62614709 | 2.33E-12 | 3.89E-11 |
| *LOC_Os04g09390.1* | 684 | 0 | 35 | 0.001 | 2.872067194 | 11.48787379 | 9.91E-12 | 1.57E-10 |
| *LOC_Os03g12879.1* | 771 | 0 | 38 | 0.001 | 2.76638023 | 11.43378375 | 1.13E-12 | 1.93E-11 |
| *LOC_Os08g15420.1* | 2238 | 0 | 100 | 0.001 | 2.507971353 | 11.29230515 | 3.84E-32 | 1.71E-30 |
| *LOC_Os05g45460.1* | 588 | 0 | 24 | 0.001 | 2.290955056 | 11.16173344 | 2.82E-08 | 3.13E-07 |
| *LOC_Os03g51530.1* | 351 | 0 | 14 | 0.001 | 2.238739556 | 11.12847099 | 3.90E-05 | 0.000271485 |
| *LOC_Os01g72370.3* | 744 | 0 | 29 | 0.001 | 2.187800494 | 11.09526547 | 7.59E-10 | 9.92E-09 |
| *LOC_Os01g19694.1* | 906 | 0 | 35 | 0.001 | 2.16831563 | 11.08235906 | 9.91E-12 | 1.57E-10 |
| *LOC_Os07g42430.1* | 369 | 0 | 14 | 0.001 | 2.129532749 | 11.0563212 | 3.90E-05 | 0.000271431 |
| *LOC_Os01g13610.1* | 981 | 0 | 37 | 0.001 | 2.11697325 | 11.04778732 | 2.33E-12 | 3.88E-11 |
| *LOC_Os03g19770.1* | 621 | 0 | 20 | 0.001 | 1.807677903 | 10.81992192 | 5.09E-07 | 4.81E-06 |
| *LOC_Os05g20930.1* | 957 | 0 | 28 | 0.001 | 1.642210207 | 10.68142309 | 1.56E-09 | 1.99E-08 |
| *LOC_Os09g37967.1* | 1053 | 0 | 30 | 0.001 | 1.599099683 | 10.64304416 | 3.68E-10 | 4.98E-09 |
| *LOC_Os05g26040.1* | 4197 | 0 | 108 | 0.001 | 1.444333352 | 10.49618804 | 1.18E-34 | 5.76E-33 |
| *LOC_Os01g73720.1* | 993 | 0 | 24 | 0.001 | 1.356577616 | 10.40575588 | 2.82E-08 | 3.13E-07 |
| *LOC_Os02g11870.1* | 543 | 0 | 13 | 0.001 | 1.343773822 | 10.39207462 | 8.03E-05 | 0.000527185 |
| *LOC_Os01g22900.3* | 1689 | 0 | 40 | 0.001 | 1.329269364 | 10.37641777 | 2.67E-13 | 4.79E-12 |
| *LOC_Os07g10230.1* | 852 | 0 | 19 | 0.001 | 1.251689646 | 10.28966118 | 1.05E-06 | 9.46E-06 |
| *LOC_Os08g06810.1* | 1521 | 0 | 30 | 0.001 | 1.107069011 | 10.11252944 | 3.68E-10 | 4.98E-09 |
| *LOC_Os12g12290.1* | 1584 | 0 | 31 | 0.001 | 1.098472453 | 10.10128298 | 1.79E-10 | 2.50E-09 |
| *LOC_Os12g31160.1* | 2808 | 0 | 54 | 0.001 | 1.079392286 | 10.07600357 | 1.07E-17 | 2.55E-16 |
| *LOC_Os01g14650.1* | 756 | 0 | 14 | 0.001 | 1.039414794 | 10.02155578 | 3.90E-05 | 0.000271538 |
| *LOC_Os04g54960.1* | 870 | 0 | 16 | 0.001 | 1.032246416 | 10.01157169 | 9.17E-06 | 7.14E-05 |
| *LOC_Os04g41750.1* | 1134 | 0 | 20 | 0.001 | 0.989918851 | 9.951166455 | 5.09E-07 | 4.81E-06 |
| *LOC_Os05g15880.1* | 882 | 0 | 15 | 0.001 | 0.954564607 | 9.898699035 | 1.89E-05 | 0.000139456 |
| *LOC_Os02g40070.1* | 2103 | 0 | 35 | 0.001 | 0.934138831 | 9.867493167 | 9.91E-12 | 1.57E-10 |
| *LOC_Os12g05990.1* | 924 | 0 | 15 | 0.001 | 0.911175306 | 9.831584839 | 1.89E-05 | 0.000139427 |
| *LOC_Os11g15130.1* | 864 | 0 | 14 | 0.001 | 0.909487945 | 9.828910705 | 3.90E-05 | 0.00027186 |
| *LOC_Os01g43490.1* | 1257 | 0 | 20 | 0.001 | 0.893053284 | 9.802602445 | 5.09E-07 | 4.81E-06 |
| *LOC_Os12g28710.1* | 1344 | 0 | 21 | 0.001 | 0.877006232 | 9.776443285 | 2.47E-07 | 2.43E-06 |
| *LOC_Os05g14370.1* | 927 | 0 | 14 | 0.001 | 0.847678084 | 9.727372678 | 3.90E-05 | 0.000271645 |
| *LOC_Os02g54450.1* | 942 | 0 | 14 | 0.001 | 0.834180026 | 9.704214957 | 3.90E-05 | 0.000271752 |
| *LOC_Os06g48020.1* | 960 | 0 | 14 | 0.001 | 0.81853915 | 9.676907611 | 3.90E-05 | 0.000271592 |
| *LOC_Os03g27850.1* | 1176 | 0 | 17 | 0.001 | 0.811379916 | 9.664233781 | 4.45E-06 | 3.65E-05 |
| *LOC_Os02g43410.1* | 2019 | 0 | 29 | 0.001 | 0.806202857 | 9.654999085 | 7.59E-10 | 9.92E-09 |
| *LOC_Os06g13350.1* | 1128 | 0 | 16 | 0.001 | 0.796147502 | 9.636891933 | 9.17E-06 | 7.14E-05 |
| *LOC_Os06g10930.1* | 1671 | 0 | 23 | 0.001 | 0.77256324 | 9.593509223 | 5.81E-08 | 6.24E-07 |
| *LOC_Os01g67410.1* | 2088 | 0 | 28 | 0.001 | 0.752679678 | 9.55589221 | 1.56E-09 | 1.99E-08 |
| *LOC_Os07g36750.1* | 2607 | 0 | 34 | 0.001 | 0.732015942 | 9.515731259 | 2.04E-11 | 3.12E-10 |
| *LOC_Os09g04210.1* | 1236 | 0 | 16 | 0.001 | 0.726581215 | 9.504980257 | 9.17E-06 | 7.14E-05 |
| *LOC_Os06g41500.1* | 1863 | 0 | 24 | 0.001 | 0.723071161 | 9.497993827 | 2.82E-08 | 3.13E-07 |
| *LOC_Os11g46860.1* | 1437 | 0 | 18 | 0.001 | 0.703069715 | 9.45752394 | 2.16E-06 | 1.86E-05 |
| *LOC_Os05g48700.1* | 1062 | 0 | 13 | 0.001 | 0.687070796 | 9.424314952 | 8.03E-05 | 0.000527087 |
| *LOC_Os05g34325.1* | 1545 | 0 | 18 | 0.001 | 0.653923094 | 9.352977164 | 2.16E-06 | 1.86E-05 |
| *LOC_Os11g24060.1* | 1641 | 0 | 19 | 0.001 | 0.649871772 | 9.344011275 | 1.05E-06 | 9.46E-06 |
| *LOC_Os01g10680.2* | 1563 | 0 | 18 | 0.001 | 0.64639231 | 9.336266223 | 2.16E-06 | 1.86E-05 |
| *LOC_Os10g30390.1* | 1575 | 0 | 18 | 0.001 | 0.641467416 | 9.325232173 | 2.16E-06 | 1.86E-05 |
| *LOC_Os03g05070.1* | 1554 | 0 | 17 | 0.001 | 0.614017234 | 9.262135338 | 4.45E-06 | 3.65E-05 |
| *LOC_Os03g56310.3* | 1602 | 0 | 16 | 0.001 | 0.56058326 | 9.130784853 | 9.17E-06 | 7.13E-05 |
| *LOC_Os05g30250.2* | 1515 | 0 | 14 | 0.001 | 0.518678273 | 9.018696129 | 3.90E-05 | 0.000271806 |
| *LOC_Os02g17240.1* | 1542 | 0 | 14 | 0.001 | 0.509596358 | 8.993211157 | 3.90E-05 | 0.000271699 |
| *LOC_Os08g09880.1* | 2010 | 0 | 18 | 0.001 | 0.502642378 | 8.9733885 | 2.16E-06 | 1.86E-05 |
| *LOC_Os09g29510.1* | 2271 | 0 | 17 | 0.001 | 0.420159745 | 8.714794136 | 4.45E-06 | 3.65E-05 |
| *LOC_Os06g13650.3* | 3522 | 0 | 25 | 0.001 | 0.398412826 | 8.638120281 | 1.37E-08 | 1.58E-07 |
| *LOC_Os12g31200.1* | 3027 | 0 | 21 | 0.001 | 0.389394244 | 8.605087748 | 2.47E-07 | 2.43E-06 |
| *LOC_Os08g28600.1* | 2901 | 0 | 20 | 0.001 | 0.386958972 | 8.5960368 | 5.09E-07 | 4.81E-06 |
| *LOC_Os07g28760.1* | 2352 | 0 | 16 | 0.001 | 0.381825843 | 8.57677094 | 9.17E-06 | 7.14E-05 |
| *LOC_Os04g58590.1* | 3039 | 0 | 20 | 0.001 | 0.369387291 | 8.52899042 | 5.09E-07 | 4.81E-06 |
| *LOC_Os04g42570.1* | 1977 | 0 | 13 | 0.001 | 0.369079001 | 8.527785847 | 8.03E-05 | 0.000526989 |
| *LOC_Os05g31530.1* | 3780 | 0 | 16 | 0.001 | 0.237580524 | 7.892272766 | 9.17E-06 | 7.14E-05 |
| *LOC_Os06g13280.1* | 1035 | 4 | 883 | 0.204491746 | 47.88538764 | 7.871399055 | 9.02E-269 | 5.50E-266 |
| *LOC_Os05g27650.1* | 4551 | 0 | 18 | 0.001 | 0.221997622 | 7.794400415 | 2.16E-06 | 1.86E-05 |
| *LOC_Os03g45550.1* | 4695 | 0 | 13 | 0.001 | 0.155414097 | 7.279973561 | 8.03E-05 | 0.000527283 |
| *LOC_Os01g45914.1* | 165 | 5 | 392 | 1.603401194 | 133.3474688 | 6.377911177 | 2.28E-114 | 4.52E-112 |
| *LOC_Os05g45430.1* | 1416 | 2 | 121 | 0.074734801 | 4.796282672 | 6.003992665 | 2.00E-35 | 9.96E-34 |
| *LOC_Os06g32020.1* | 408 | 2 | 114 | 0.259373722 | 15.68293498 | 5.918019442 | 2.81E-33 | 1.30E-31 |
| *LOC_Os05g28100.1* | 1116 | 1 | 44 | 0.047412401 | 2.212947626 | 5.544561046 | 3.57E-13 | 6.35E-12 |
| *LOC_Os01g03330.1* | 780 | 2 | 88 | 0.135672409 | 6.332434745 | 5.544561046 | 2.49E-25 | 8.58E-24 |
| *LOC_Os10g39980.1* | 1458 | 2 | 85 | 0.072581947 | 3.272231759 | 5.494520364 | 2.04E-24 | 6.76E-23 |
| *LOC_Os01g06900.1* | 3093 | 2 | 71 | 0.034214186 | 1.288430753 | 5.234876547 | 3.60E-20 | 9.81E-19 |
| *LOC_Os01g05560.1* | 1151 | 6 | 210 | 0.275824011 | 10.24062881 | 5.214412445 | 2.84E-57 | 2.40E-55 |
| *LOC_Os10g26340.1* | 1668 | 3 | 104 | 0.095165898 | 3.4996124 | 5.200606645 | 6.08E-29 | 2.40E-27 |
| *LOC_Os06g31890.1* | 408 | 22 | 676 | 2.853110947 | 92.99705304 | 5.026577245 | 5.66E-178 | 1.87E-175 |
| *LOC_Os01g19370.1* | 768 | 1 | 25 | 0.068896145 | 1.827096318 | 4.728985617 | 1.97E-07 | 1.96E-06 |
| *LOC_Os12g25450.1* | 1080 | 19 | 471 | 0.930863471 | 24.4782184 | 4.716785164 | 3.21E-120 | 6.73E-118 |
| *LOC_Os01g37000.1* | 1221 | 5 | 118 | 0.216675837 | 5.424366149 | 4.645844382 | 7.23E-31 | 3.07E-29 |
| *LOC_Os04g52810.1* | 852 | 1 | 23 | 0.062103567 | 1.515203256 | 4.608691384 | 7.76E-07 | 7.14E-06 |
| *LOC_Os06g08640.1* | 1302 | 1 | 23 | 0.040639201 | 0.991515495 | 4.608691384 | 7.76E-07 | 7.14E-06 |
| *LOC_Os06g11840.1* | 1503 | 1 | 23 | 0.035204417 | 0.858917614 | 4.608691384 | 7.76E-07 | 7.14E-06 |
| *LOC_Os01g52880.1* | 1365 | 7 | 153 | 0.271344817 | 6.291315039 | 4.535162348 | 4.25E-39 | 2.34E-37 |
| *LOC_Os01g06870.1* | 1521 | 2 | 43 | 0.069575594 | 1.586798916 | 4.511394182 | 8.71E-12 | 1.39E-10 |
| *LOC_Os05g07600.1* | 639 | 2 | 42 | 0.165609513 | 3.689190536 | 4.47744685 | 1.72E-11 | 2.66E-10 |
| *LOC_Os05g01380.1* | 930 | 3 | 63 | 0.170684643 | 3.802246375 | 4.47744685 | 1.09E-16 | 2.46E-15 |
| *LOC_Os02g45380.1* | 1650 | 1 | 21 | 0.032068024 | 0.71436144 | 4.47744685 | 3.04E-06 | 2.56E-05 |
| *LOC_Os06g44970.1* | 1893 | 2 | 41 | 0.055903053 | 1.215670551 | 4.442681432 | 3.39E-11 | 5.07E-10 |
| *LOC_Os11g13800.2* | 1344 | 1 | 20 | 0.039369226 | 0.835244031 | 4.407057523 | 6.01E-06 | 4.81E-05 |
| *LOC_Os03g04190.1* | 1548 | 1 | 19 | 0.034181033 | 0.688914456 | 4.333056941 | 1.18E-05 | 9.03E-05 |
| *LOC_Os06g13180.1* | 1116 | 1 | 19 | 0.047412401 | 0.95559102 | 4.333056941 | 1.18E-05 | 9.03E-05 |
| *LOC_Os05g25650.1* | 297 | 1 | 19 | 0.178155688 | 3.590705652 | 4.333056941 | 1.18E-05 | 9.03E-05 |
| *LOC_Os10g11889.2* | 180 | 2 | 38 | 0.587913771 | 11.84932865 | 4.333056941 | 2.58E-10 | 3.55E-09 |
| *LOC_Os12g17430.1* | 846 | 2 | 37 | 0.125088036 | 2.454788131 | 4.294582793 | 5.06E-10 | 6.75E-09 |
| *LOC_Os06g49830.1* | 1080 | 2 | 35 | 0.097985628 | 1.81897589 | 4.214412445 | 1.94E-09 | 2.44E-08 |
| *LOC_Os01g06740.1* | 849 | 1 | 17 | 0.062323015 | 1.123890201 | 4.172592269 | 4.57E-05 | 0.000314172 |
| *LOC_Os01g23430.1* | 2232 | 1 | 17 | 0.0237062 | 0.427501246 | 4.172592269 | 4.57E-05 | 0.000314111 |
| *LOC_Os10g40510.1* | 402 | 7 | 119 | 0.921357402 | 16.61512305 | 4.172592269 | 3.75E-29 | 1.49E-27 |
| *LOC_Os04g38370.1* | 573 | 1 | 17 | 0.092342477 | 1.665240455 | 4.172592269 | 4.57E-05 | 0.000314233 |
| *LOC_Os04g45240.1* | 636 | 1 | 17 | 0.083195345 | 1.500287391 | 4.172592269 | 4.57E-05 | 0.00031405 |
| *LOC_Os07g23850.1* | 810 | 2 | 33 | 0.130647505 | 2.286712547 | 4.129523547 | 7.41E-09 | 8.81E-08 |
| *LOC_Os11g24440.1* | 486 | 1 | 16 | 0.108872921 | 1.847848523 | 4.085129428 | 8.94E-05 | 0.000580252 |
| *LOC_Os04g59210.1* | 1008 | 1 | 16 | 0.052492301 | 0.890926966 | 4.085129428 | 8.94E-05 | 0.000580358 |
| *LOC_Os11g09360.1* | 705 | 1 | 16 | 0.075052822 | 1.273836003 | 4.085129428 | 8.94E-05 | 0.000580571 |
| *LOC_Os01g06560.1* | 810 | 1 | 16 | 0.065323752 | 1.108709114 | 4.085129428 | 8.94E-05 | 0.000580464 |
| *LOC_Os05g06940.1* | 1737 | 4 | 63 | 0.121847414 | 2.035745037 | 4.062409351 | 9.68E-16 | 2.05E-14 |
| *LOC_Os12g36220.1* | 282 | 4 | 63 | 0.750528218 | 12.53932315 | 4.062409351 | 9.68E-16 | 2.05E-14 |
| *LOC_Os06g44100.1* | 1260 | 2 | 31 | 0.083987682 | 1.380936798 | 4.039325738 | 2.81E-08 | 3.12E-07 |
| *LOC_Os02g42150.2* | 2718 | 2 | 30 | 0.038934687 | 0.619518751 | 3.992020023 | 5.46E-08 | 5.88E-07 |
| *LOC_Os04g40990.1* | 1704 | 4 | 60 | 0.124207135 | 1.976352073 | 3.992020023 | 7.06E-15 | 1.42E-13 |
| *LOC_Os10g31530.1* | 570 | 2 | 29 | 0.18565698 | 2.855655381 | 3.943110423 | 1.06E-07 | 1.09E-06 |
| *LOC_Os10g21090.1* | 1995 | 3 | 42 | 0.079567277 | 1.181650503 | 3.89248435 | 1.39E-10 | 1.96E-09 |
| *LOC_Os08g44940.1* | 648 | 3 | 41 | 0.244964071 | 3.55133388 | 3.857718932 | 2.67E-10 | 3.67E-09 |
| *LOC_Os09g36160.1* | 948 | 2 | 27 | 0.111629197 | 1.598593639 | 3.84001693 | 3.96E-07 | 3.81E-06 |
| *LOC_Os01g03680.1* | 540 | 17 | 222 | 1.665755684 | 23.07500843 | 3.792082453 | 1.15E-49 | 8.44E-48 |
| *LOC_Os12g28177.1* | 1533 | 2 | 26 | 0.069030971 | 0.951949361 | 3.785569146 | 7.64E-07 | 7.04E-06 |
| *LOC_Os04g13590.1* | 1134 | 2 | 26 | 0.093319646 | 1.286894507 | 3.785569146 | 7.64E-07 | 7.04E-06 |
| *LOC_Os03g52860.1* | 2613 | 40 | 506 | 0.809984529 | 10.86910441 | 3.746194907 | 3.61E-110 | 6.72E-108 |
| *LOC_Os05g01680.1* | 993 | 2 | 25 | 0.106570472 | 1.413101684 | 3.728985617 | 1.47E-06 | 1.30E-05 |
| *LOC_Os03g02939.1* | 969 | 5 | 61 | 0.273024971 | 3.533366699 | 3.69393867 | 2.55E-14 | 4.95E-13 |
| *LOC_Os01g16810.1* | 1017 | 2 | 24 | 0.104055535 | 1.324563985 | 3.670091928 | 2.82E-06 | 2.38E-05 |
| *LOC_Os03g60960.1* | 1413 | 3 | 36 | 0.112340211 | 1.430022901 | 3.670091928 | 6.98E-09 | 8.32E-08 |
| *LOC_Os08g38560.1* | 3315 | 6 | 70 | 0.095768759 | 1.185215059 | 3.629449944 | 4.83E-16 | 1.05E-14 |
| *LOC_Os11g47447.1* | 1155 | 2 | 23 | 0.091622925 | 1.117708376 | 3.608691384 | 5.40E-06 | 4.35E-05 |
| *LOC_Os02g52630.1* | 1827 | 2 | 23 | 0.057922539 | 0.706597249 | 3.608691384 | 5.40E-06 | 4.35E-05 |
| *LOC_Os01g50420.1* | 1626 | 3 | 34 | 0.097624058 | 1.173656557 | 3.587629768 | 2.54E-08 | 2.84E-07 |
| *LOC_Os01g41420.1* | 1896 | 4 | 45 | 0.111629197 | 1.332161366 | 3.576982524 | 1.26E-10 | 1.79E-09 |
| *LOC_Os11g31530.1* | 603 | 3 | 33 | 0.263244972 | 3.071703421 | 3.544561046 | 4.84E-08 | 5.23E-07 |
| *LOC_Os10g33250.1* | 1866 | 2 | 22 | 0.056711939 | 0.661749612 | 3.544561046 | 1.03E-05 | 7.94E-05 |
| *LOC_Os06g40870.1* | 618 | 2 | 22 | 0.171237021 | 1.998098342 | 3.544561046 | 1.03E-05 | 7.94E-05 |
| *LOC_Os06g18880.1* | 2775 | 2 | 22 | 0.038134947 | 0.444981901 | 3.544561046 | 1.03E-05 | 7.94E-05 |
| *LOC_Os09g13540.1* | 1728 | 4 | 43 | 0.122482036 | 1.396713629 | 3.511394182 | 4.53E-10 | 6.07E-09 |
| *LOC_Os08g29770.1* | 1551 | 34 | 352 | 1.159907246 | 12.73836003 | 3.457098205 | 3.52E-72 | 3.93E-70 |
| *LOC_Os04g48930.4* | 1470 | 3 | 31 | 0.107984162 | 1.183660112 | 3.454363237 | 1.74E-07 | 1.75E-06 |
| *LOC_Os05g03884.1* | 936 | 10 | 100 | 0.565301703 | 5.996623812 | 3.407057523 | 2.84E-21 | 8.20E-20 |
| *LOC_Os08g31860.1* | 1158 | 14 | 135 | 0.639698922 | 6.54346619 | 3.354590103 | 7.11E-28 | 2.70E-26 |
| *LOC_Os06g44750.1* | 792 | 2 | 19 | 0.133616766 | 1.34651462 | 3.333056941 | 7.02E-05 | 0.000466172 |
| *LOC_Os02g36070.1* | 1503 | 2 | 19 | 0.070408835 | 0.709540638 | 3.333056941 | 7.02E-05 | 0.00046626 |
| *LOC_Os04g49510.4* | 831 | 2 | 19 | 0.127345943 | 1.283320793 | 3.333056941 | 7.02E-05 | 0.000466348 |
| *LOC_Os11g42430.1* | 1389 | 4 | 37 | 0.152375059 | 1.495140935 | 3.294582793 | 2.02E-08 | 2.29E-07 |
| *LOC_Os02g36190.1* | 1557 | 18 | 163 | 0.611702189 | 5.875998084 | 3.26393258 | 1.42E-32 | 6.41E-31 |
| *LOC_Os01g71080.1* | 1275 | 3 | 27 | 0.124499387 | 1.188601388 | 3.255054429 | 2.18E-06 | 1.87E-05 |
| *LOC_Os05g24340.1* | 1236 | 8 | 72 | 0.342474041 | 3.269615469 | 3.255054429 | 4.20E-15 | 8.56E-14 |
| *LOC_Os09g37200.1* | 1359 | 3 | 27 | 0.11680406 | 1.115133753 | 3.255054429 | 2.18E-06 | 1.87E-05 |
| *LOC_Os04g35950.1* | 5751 | 2 | 18 | 0.018401057 | 0.17567574 | 3.255054429 | 0.000132016 | 0.000827226 |
| *LOC_Os05g49640.1* | 1758 | 2 | 18 | 0.060195949 | 0.574693504 | 3.255054429 | 0.000132016 | 0.000828252 |
| *LOC_Os01g58130.1* | 339 | 2 | 18 | 0.312166604 | 2.980268967 | 3.255054429 | 0.000132016 | 0.000827519 |
| *LOC_Os04g09920.1* | 1509 | 2 | 18 | 0.070128879 | 0.669523645 | 3.255054429 | 0.000132016 | 0.000827372 |
| *LOC_Os04g45890.1* | 1314 | 2 | 18 | 0.080536133 | 0.768882176 | 3.255054429 | 0.000132016 | 0.000827812 |
| *LOC_Os01g52470.1* | 2562 | 2 | 18 | 0.041305417 | 0.394344723 | 3.255054429 | 0.000132016 | 0.000827959 |
| *LOC_Os03g14060.1* | 3855 | 2 | 18 | 0.027451227 | 0.262078127 | 3.255054429 | 0.000132016 | 0.000827665 |
| *LOC_Os04g03790.1* | 5877 | 2 | 18 | 0.018006547 | 0.171909338 | 3.255054429 | 0.000132016 | 0.000828105 |
| *LOC_Os01g09220.1* | 1362 | 55 | 490 | 2.136691018 | 20.19303631 | 3.240407653 | 3.28E-94 | 5.21E-92 |
| *LOC_Os05g04410.1* | 1050 | 4 | 35 | 0.201570436 | 1.870946629 | 3.214412445 | 7.05E-08 | 7.49E-07 |
| *LOC_Os12g08025.1* | 504 | 4 | 34 | 0.419938408 | 3.786439607 | 3.172592269 | 1.31E-07 | 1.34E-06 |
| *LOC_Os04g56040.1* | 663 | 4 | 34 | 0.319229197 | 2.87837943 | 3.172592269 | 1.31E-07 | 1.34E-06 |
| *LOC_Os05g03640.1* | 1107 | 6 | 50 | 0.286787205 | 2.535158034 | 3.144023117 | 1.46E-10 | 2.06E-09 |
| *LOC_Os10g05820.1* | 660 | 3 | 25 | 0.240510179 | 2.126075715 | 3.144023117 | 7.58E-06 | 5.98E-05 |
| *LOC_Os07g35570.1* | 1011 | 3 | 25 | 0.157009612 | 1.387942603 | 3.144023117 | 7.58E-06 | 5.98E-05 |
| *LOC_Os11g37230.2* | 465 | 4 | 32 | 0.455159049 | 3.862599493 | 3.085129428 | 4.50E-07 | 4.30E-06 |
| *LOC_Os08g15530.1* | 615 | 4 | 32 | 0.344144646 | 2.920502055 | 3.085129428 | 4.50E-07 | 4.30E-06 |
| *LOC_Os07g33720.1* | 2598 | 3 | 24 | 0.061099584 | 0.518507149 | 3.085129428 | 1.41E-05 | 0.000105983 |
| *LOC_Os02g09070.1* | 699 | 3 | 24 | 0.227091156 | 1.927155326 | 3.085129428 | 1.41E-05 | 0.000106006 |
| *LOC_Os12g36210.1* | 234 | 9 | 71 | 2.03508613 | 17.03041162 | 3.064951546 | 3.67E-14 | 7.03E-13 |
| *LOC_Os10g31990.1* | 4554 | 17 | 134 | 0.197520437 | 1.651560266 | 3.063755777 | 1.57E-25 | 5.45E-24 |
| *LOC_Os09g27135.1* | 1158 | 4 | 31 | 0.182771121 | 1.502573718 | 3.039325738 | 8.31E-07 | 7.60E-06 |
| *LOC_Os01g06876.1* | 669 | 3 | 23 | 0.237274616 | 1.929675896 | 3.023728883 | 2.60E-05 | 0.000187689 |
| *LOC_Os02g50050.1* | 573 | 3 | 23 | 0.277027431 | 2.252972381 | 3.023728883 | 2.60E-05 | 0.00018765 |
| *LOC_Os09g35010.1* | 657 | 11 | 83 | 0.885897463 | 7.090802293 | 3.00073724 | 4.99E-16 | 1.08E-14 |
| *LOC_Os01g72150.1* | 693 | 4 | 30 | 0.305409751 | 2.429800817 | 2.992020023 | 1.53E-06 | 1.35E-05 |
| *LOC_Os06g36390.2* | 693 | 4 | 30 | 0.305409751 | 2.429800817 | 2.992020023 | 1.53E-06 | 1.35E-05 |
| *LOC_Os10g08970.1* | 2319 | 4 | 30 | 0.091267338 | 0.72611124 | 2.992020023 | 1.53E-06 | 1.35E-05 |
| *LOC_Os08g36700.1* | 1143 | 4 | 30 | 0.185169692 | 1.473186322 | 2.992020023 | 1.53E-06 | 1.35E-05 |
| *LOC_Os01g02430.1* | 1893 | 5 | 37 | 0.139757632 | 1.097068546 | 2.972654698 | 9.19E-08 | 9.58E-07 |
| *LOC_Os03g41280.1* | 843 | 3 | 22 | 0.188299784 | 1.464798073 | 2.959598546 | 4.79E-05 | 0.000327825 |
| *LOC_Os08g24750.1* | 1791 | 4 | 29 | 0.118173622 | 0.908835046 | 2.943110423 | 2.80E-06 | 2.37E-05 |
| *LOC_Os01g03320.1* | 561 | 10 | 72 | 0.943177173 | 7.203644776 | 2.933126334 | 8.65E-14 | 1.61E-12 |
| *LOC_Os01g03360.1* | 765 | 29 | 206 | 2.005823454 | 15.11431395 | 2.91364896 | 1.21E-36 | 6.17E-35 |
| *LOC_Os02g43370.1* | 2025 | 4 | 28 | 0.104518004 | 0.77609638 | 2.89248435 | 5.11E-06 | 4.14E-05 |
| *LOC_Os01g09230.1* | 843 | 4 | 28 | 0.251066379 | 1.864288456 | 2.89248435 | 5.11E-06 | 4.14E-05 |
| *LOC_Os01g15910.1* | 2262 | 3 | 21 | 0.070175384 | 0.521085931 | 2.89248435 | 8.77E-05 | 0.000571043 |
| *LOC_Os02g36140.2* | 2490 | 3 | 21 | 0.063749686 | 0.473372039 | 2.89248435 | 8.77E-05 | 0.000570937 |
| *LOC_Os04g11030.1* | 246 | 3 | 21 | 0.645271212 | 4.791448685 | 2.89248435 | 8.77E-05 | 0.000571148 |
| *LOC_Os03g60750.2* | 792 | 4 | 28 | 0.267233532 | 1.984337334 | 2.89248435 | 5.11E-06 | 4.14E-05 |
| *LOC_Os05g25370.1* | 2541 | 60 | 413 | 1.249403527 | 9.122797613 | 2.868236803 | 9.62E-71 | 1.04E-68 |
| *LOC_Os01g12070.1* | 1923 | 4 | 27 | 0.11006186 | 0.788074243 | 2.84001693 | 9.30E-06 | 7.22E-05 |
| *LOC_Os04g01810.1* | 1494 | 22 | 148 | 0.779162829 | 5.560242994 | 2.835151175 | 4.92E-26 | 1.74E-24 |
| *LOC_Os07g48030.1* | 945 | 58 | 390 | 3.247523687 | 23.16410112 | 2.834478746 | 4.36E-66 | 4.39E-64 |
| *LOC_Os01g52740.1* | 780 | 6 | 40 | 0.407017226 | 2.87837943 | 2.822095022 | 6.13E-08 | 6.56E-07 |
| *LOC_Os07g03900.1* | 2031 | 3 | 20 | 0.078156927 | 0.552716877 | 2.822095022 | 0.000159978 | 0.000985003 |
| *LOC_Os03g61480.1* | 501 | 3 | 20 | 0.316839757 | 2.240654646 | 2.822095022 | 0.000159978 | 0.000985346 |
| *LOC_Os04g28860.1* | 2883 | 3 | 20 | 0.055059562 | 0.389374949 | 2.822095022 | 0.000159978 | 0.000984831 |
| *LOC_Os03g22600.1* | 2016 | 3 | 20 | 0.078738451 | 0.556829354 | 2.822095022 | 0.000159978 | 0.000985174 |
| *LOC_Os04g59260.1* | 1017 | 130 | 848 | 6.763609755 | 46.80126081 | 2.790682069 | 2.28E-139 | 5.45E-137 |
| *LOC_Os09g04339.1* | 948 | 6 | 39 | 0.334887591 | 2.309079701 | 2.785569146 | 1.11E-07 | 1.14E-06 |
| *LOC_Os09g23820.1* | 1608 | 4 | 26 | 0.131622486 | 0.907548738 | 2.785569146 | 1.68E-05 | 0.000125189 |
| *LOC_Os04g28780.1* | 2454 | 10 | 64 | 0.215616297 | 1.463821324 | 2.763201333 | 9.75E-12 | 1.54E-10 |
| *LOC_Os03g48000.2* | 1335 | 4 | 25 | 0.158538545 | 1.051093612 | 2.728985617 | 3.04E-05 | 0.000216518 |
| *LOC_Os12g05180.1* | 1719 | 5 | 31 | 0.153904129 | 1.012204983 | 2.717397643 | 3.26E-06 | 2.73E-05 |
| *LOC_Os08g30780.1* | 2967 | 5 | 31 | 0.089167913 | 0.586444343 | 2.717397643 | 3.26E-06 | 2.73E-05 |
| *LOC_Os01g02570.1* | 2019 | 117 | 724 | 3.066236755 | 20.12727132 | 2.714610595 | 8.80E-116 | 1.75E-113 |
| *LOC_Os01g48040.1* | 2319 | 6 | 37 | 0.136901007 | 0.895537196 | 2.709620293 | 3.56E-07 | 3.46E-06 |
| *LOC_Os10g20390.1* | 975 | 24 | 148 | 1.302455123 | 8.520003111 | 2.709620293 | 7.12E-25 | 2.41E-23 |
| *LOC_Os05g47940.1* | 1533 | 7 | 42 | 0.241608399 | 1.537764353 | 2.670091928 | 7.03E-08 | 7.46E-07 |
| *LOC_Os03g57720.1* | 435 | 13 | 78 | 1.581285315 | 10.06440256 | 2.670091928 | 1.46E-13 | 2.67E-12 |
| *LOC_Os03g56900.2* | 591 | 4 | 24 | 0.358120064 | 2.279325843 | 2.670091928 | 5.46E-05 | 0.000370276 |
| *LOC_Os01g43720.1* | 1143 | 6 | 36 | 0.277754537 | 1.767823587 | 2.670091928 | 6.37E-07 | 5.94E-06 |
| *LOC_Os09g33530.1* | 1998 | 23 | 133 | 0.609099853 | 3.7362748 | 2.616849907 | 1.05E-21 | 3.09E-20 |
| *LOC_Os08g08020.1* | 1263 | 9 | 51 | 0.377046837 | 2.266467413 | 2.587629768 | 4.88E-09 | 5.88E-08 |
| *LOC_Os02g15870.3* | 1185 | 11 | 62 | 0.491168467 | 2.936675722 | 2.579894119 | 1.10E-10 | 1.58E-09 |
| *LOC_Os07g40290.1* | 1818 | 11 | 61 | 0.320151063 | 1.883296112 | 2.556435147 | 1.93E-10 | 2.70E-09 |
| *LOC_Os08g27720.1* | 1059 | 23 | 127 | 1.149179892 | 6.731167759 | 2.550252158 | 3.07E-20 | 8.40E-19 |
| *LOC_Os04g25490.1* | 1428 | 8 | 44 | 0.296427111 | 1.729446464 | 2.544561046 | 7.68E-08 | 8.11E-07 |
| *LOC_Os09g15700.1* | 2904 | 6 | 33 | 0.109322809 | 0.637822715 | 2.544561046 | 3.56E-06 | 2.96E-05 |
| *LOC_Os01g02790.1* | 1464 | 8 | 43 | 0.28913792 | 1.648580022 | 2.511394182 | 1.35E-07 | 1.37E-06 |
| *LOC_Os12g17160.1* | 1017 | 8 | 43 | 0.416222139 | 2.37317714 | 2.511394182 | 1.35E-07 | 1.37E-06 |
| *LOC_Os01g36294.1* | 1089 | 104 | 557 | 5.053143156 | 28.70846481 | 2.506223227 | 6.37E-82 | 8.35E-80 |
| *LOC_Os02g42950.1* | 771 | 6 | 32 | 0.4117684 | 2.329583352 | 2.500166927 | 6.27E-06 | 5.00E-05 |
| *LOC_Os03g41170.1* | 816 | 7 | 37 | 0.453904014 | 2.545037694 | 2.487227871 | 1.21E-06 | 1.08E-05 |
| *LOC_Os07g15460.1* | 1557 | 12 | 63 | 0.40780146 | 2.271091284 | 2.47744685 | 2.07E-10 | 2.87E-09 |
| *LOC_Os04g34190.1* | 3390 | 70 | 366 | 1.092583114 | 6.059880233 | 2.471546249 | 1.46E-53 | 1.16E-51 |
| *LOC_Os01g45110.1* | 1476 | 19 | 99 | 0.681119613 | 3.764709681 | 2.466558534 | 1.56E-15 | 3.25E-14 |
| *LOC_Os01g37540.1* | 6084 | 6 | 31 | 0.052181696 | 0.285992828 | 2.454363237 | 1.10E-05 | 8.43E-05 |
| *LOC_Os01g02810.1* | 1926 | 9 | 46 | 0.247253455 | 1.34055366 | 2.438766382 | 8.08E-08 | 8.49E-07 |
| *LOC_Os07g11739.1* | 1554 | 52 | 265 | 1.770551125 | 9.571445111 | 2.434538259 | 1.45E-38 | 7.85E-37 |
| *LOC_Os07g24820.1* | 324 | 11 | 56 | 1.796403189 | 9.701204744 | 2.433052731 | 3.15E-09 | 3.88E-08 |
| *LOC_Os01g54270.1* | 2406 | 14 | 71 | 0.307885017 | 1.656324323 | 2.427521625 | 2.48E-11 | 3.77E-10 |
| *LOC_Os11g19840.1* | 1125 | 19 | 96 | 0.893628932 | 4.789623371 | 2.422164415 | 8.11E-15 | 1.62E-13 |
| *LOC_Os02g52000.1* | 942 | 5 | 25 | 0.280850528 | 1.489607189 | 2.407057523 | 0.000100869 | 0.000647531 |
| *LOC_Os10g12500.1* | 921 | 6 | 30 | 0.344705143 | 1.828286608 | 2.407057523 | 1.92E-05 | 0.000141312 |
| *LOC_Os05g10730.1* | 4461 | 6 | 30 | 0.071166428 | 0.377460651 | 2.407057523 | 1.92E-05 | 0.000141283 |
| *LOC_Os01g52340.1* | 1524 | 8 | 40 | 0.277754537 | 1.473186322 | 2.407057523 | 7.17E-07 | 6.64E-06 |
| *LOC_Os06g47360.1* | 387 | 9 | 45 | 1.230517195 | 6.526558009 | 2.407057523 | 1.40E-07 | 1.42E-06 |
| *LOC_Os01g64360.1* | 522 | 13 | 65 | 1.317737763 | 6.989168442 | 2.407057523 | 2.15E-10 | 2.99E-09 |
| *LOC_Os03g59774.1* | 162 | 39 | 194 | 12.7381317 | 67.21549001 | 2.399640051 | 3.46E-28 | 1.33E-26 |
| *LOC_Os10g10420.1* | 1347 | 12 | 59 | 0.471378525 | 2.458482208 | 2.382809976 | 1.87E-09 | 2.35E-08 |
| *LOC_Os04g30490.1* | 1452 | 40 | 195 | 1.457637449 | 7.537904808 | 2.370531646 | 6.14E-28 | 2.34E-26 |
| *LOC_Os07g24830.1* | 288 | 353 | 1717 | 64.85423786 | 334.6266002 | 2.367279378 | 1.32E-232 | 6.60E-230 |
| *LOC_Os11g35860.1* | 1656 | 6 | 29 | 0.191711012 | 0.98292486 | 2.358147922 | 3.33E-05 | 0.000235583 |
| *LOC_Os01g50400.1* | 1257 | 8 | 38 | 0.336752518 | 1.696801239 | 2.333056941 | 2.15E-06 | 1.85E-05 |
| *LOC_Os05g44930.1* | 2844 | 20 | 95 | 0.372097323 | 1.874893774 | 2.333056941 | 4.23E-14 | 8.06E-13 |
| *LOC_Os09g37580.1* | 3732 | 7 | 33 | 0.099245894 | 0.496312209 | 2.322168625 | 1.11E-05 | 8.49E-05 |
| *LOC_Os02g03900.1* | 1638 | 13 | 61 | 0.419938408 | 2.090251729 | 2.315427047 | 1.89E-09 | 2.37E-08 |
| *LOC_Os06g12260.1* | 2115 | 6 | 28 | 0.150105644 | 0.743071002 | 2.307521849 | 5.76E-05 | 0.000389055 |
| *LOC_Os12g36830.1* | 483 | 14 | 65 | 1.533688098 | 7.553511236 | 2.300142319 | 6.44E-10 | 8.49E-09 |
| *LOC_Os09g20090.1* | 1734 | 16 | 74 | 0.488232889 | 2.395329594 | 2.294582793 | 4.41E-11 | 6.54E-10 |
| *LOC_Os12g16520.1* | 1530 | 21 | 95 | 0.726246423 | 3.485096662 | 2.262667613 | 1.23E-13 | 2.26E-12 |
| *LOC_Os01g56080.1* | 549 | 6 | 27 | 0.57827584 | 2.760413059 | 2.255054429 | 9.91E-05 | 0.000637643 |
| *LOC_Os01g74360.1* | 267 | 6 | 27 | 1.189039087 | 5.675905504 | 2.255054429 | 9.91E-05 | 0.000637875 |
| *LOC_Os10g08780.1* | 321 | 18 | 81 | 2.967041461 | 14.16324084 | 2.255054429 | 8.77E-12 | 1.40E-10 |
| *LOC_Os09g07730.1* | 1959 | 6 | 27 | 0.162058926 | 0.773592021 | 2.255054429 | 9.91E-05 | 0.000637759 |
| *LOC_Os07g03250.1* | 945 | 25 | 112 | 1.399794693 | 6.652254682 | 2.24862816 | 1.01E-15 | 2.14E-14 |
| *LOC_Os01g50410.1* | 1356 | 17 | 76 | 0.663354034 | 3.145839465 | 2.2455941 | 4.35E-11 | 6.47E-10 |
| *LOC_Os09g35030.1* | 717 | 32 | 143 | 2.361494645 | 11.19436686 | 2.245000764 | 1.21E-19 | 3.20E-18 |
| *LOC_Os03g12950.1* | 981 | 11 | 49 | 0.593307475 | 2.803559169 | 2.240407653 | 1.39E-07 | 1.41E-06 |
| *LOC_Os07g41439.1* | 720 | 9 | 40 | 0.661402992 | 3.118244382 | 2.237132521 | 2.11E-06 | 1.82E-05 |
| *LOC_Os02g45880.1* | 708 | 7 | 31 | 0.52314361 | 2.457599386 | 2.231970816 | 3.26E-05 | 0.000230785 |
| *LOC_Os10g36610.1* | 270 | 7 | 31 | 1.371798799 | 6.444371723 | 2.231970816 | 3.26E-05 | 0.000230739 |
| *LOC_Os03g41390.1* | 717 | 15 | 66 | 1.106950615 | 5.166630859 | 2.222632951 | 1.08E-09 | 1.39E-08 |
| *LOC_Os03g02160.2* | 2448 | 10 | 44 | 0.216144769 | 1.008843771 | 2.222632951 | 7.05E-07 | 6.53E-06 |
| *LOC_Os04g49370.1* | 618 | 23 | 101 | 1.969225738 | 9.173087842 | 2.219778954 | 4.14E-14 | 7.90E-13 |
| *LOC_Os03g10210.1* | 720 | 23 | 99 | 1.690252092 | 7.717654845 | 2.190924092 | 1.17E-13 | 2.17E-12 |
| *LOC_Os02g40454.2* | 663 | 7 | 30 | 0.558651095 | 2.539746555 | 2.184665101 | 5.55E-05 | 0.000375644 |
| *LOC_Os01g69930.1* | 2235 | 7 | 30 | 0.16572066 | 0.753401327 | 2.184665101 | 5.55E-05 | 0.000375716 |
| *LOC_Os09g03740.1* | 402 | 15 | 63 | 1.974337291 | 8.796241615 | 2.155518756 | 5.16E-09 | 6.20E-08 |
| *LOC_Os11g37210.1* | 687 | 7 | 29 | 0.539134899 | 2.369321059 | 2.135755501 | 9.40E-05 | 0.00060772 |
| *LOC_Os04g48290.1* | 1683 | 17 | 70 | 0.534467065 | 2.334514511 | 2.126949603 | 9.92E-10 | 1.29E-08 |
| *LOC_Os01g03180.1* | 954 | 22 | 90 | 1.220198393 | 5.295131969 | 2.117550905 | 4.52E-12 | 7.38E-11 |
| *LOC_Os05g49940.1* | 423 | 36 | 147 | 4.50316931 | 19.50561379 | 2.114876771 | 8.37E-19 | 2.11E-17 |
| *LOC_Os01g43774.1* | 1953 | 12 | 49 | 0.325113606 | 1.408239398 | 2.114876771 | 3.79E-07 | 3.66E-06 |
| *LOC_Os03g55230.1* | 1038 | 14 | 57 | 0.713652554 | 3.082195314 | 2.11066452 | 4.28E-08 | 4.66E-07 |
| *LOC_Os11g05360.1* | 1059 | 12 | 48 | 0.599572118 | 2.544063405 | 2.085129428 | 6.33E-07 | 5.90E-06 |
| *LOC_Os01g53040.1* | 960 | 16 | 64 | 0.881870656 | 3.741893258 | 2.085129428 | 8.14E-09 | 9.64E-08 |
| *LOC_Os05g34260.1* | 1668 | 67 | 268 | 2.125371726 | 9.018231954 | 2.085129428 | 1.79E-32 | 8.05E-31 |
| *LOC_Os03g55300.1* | 729 | 12 | 48 | 0.870983364 | 3.695697045 | 2.085129428 | 6.33E-07 | 5.90E-06 |
| *LOC_Os01g43400.1* | 477 | 7 | 28 | 0.776489886 | 3.294748781 | 2.085129428 | 0.00015831 | 0.000976431 |
| *LOC_Os02g51970.1* | 927 | 8 | 32 | 0.456632055 | 1.937549907 | 2.085129428 | 5.19E-05 | 0.000353201 |
| *LOC_Os02g13430.1* | 3015 | 14 | 56 | 0.245695307 | 1.042517525 | 2.085129428 | 7.15E-08 | 7.58E-07 |
| *LOC_Os01g58280.1* | 2220 | 99 | 385 | 2.359599865 | 9.733979084 | 2.044487443 | 7.91E-45 | 5.20E-43 |
| *LOC_Os05g04470.1* | 999 | 9 | 35 | 0.476686841 | 1.966460421 | 2.044487443 | 2.86E-05 | 0.000204572 |
| *LOC_Os03g50960.1* | 372 | 17 | 66 | 2.418032445 | 9.958264317 | 2.042060706 | 7.57E-09 | 9.00E-08 |
| *LOC_Os01g43740.1* | 1605 | 54 | 208 | 1.780224877 | 7.273960727 | 2.030681644 | 9.69E-25 | 3.26E-23 |
| *LOC_Os03g59100.1* | 1632 | 19 | 73 | 0.616012591 | 2.510645293 | 2.027026473 | 1.44E-09 | 1.84E-08 |
| *LOC_Os04g58810.1* | 990 | 67 | 257 | 3.580929332 | 14.57070557 | 2.024664786 | 4.29E-30 | 1.77E-28 |
| *LOC_Os06g35670.1* | 420 | 12 | 46 | 1.511778268 | 6.147396067 | 2.023728883 | 1.75E-06 | 1.53E-05 |
| *LOC_Os09g19280.1* | 1440 | 12 | 46 | 0.440935328 | 1.79299052 | 2.023728883 | 1.75E-06 | 1.53E-05 |
| *LOC_Os10g38340.1* | 720 | 16 | 61 | 1.175827542 | 4.755322683 | 2.015866765 | 3.70E-08 | 4.05E-07 |
| *LOC_Os11g01240.1* | 1656 | 20 | 76 | 0.639036708 | 2.575941011 | 2.011128846 | 8.04E-10 | 1.05E-08 |
| *LOC_Os05g50070.1* | 1863 | 10 | 38 | 0.284016314 | 1.144862672 | 2.011128846 | 1.57E-05 | 0.000117452 |
| *LOC_Os02g53970.1* | 2373 | 14 | 53 | 0.312166604 | 1.2536052 | 2.00569496 | 3.25E-07 | 3.16E-06 |
| *LOC_Os06g42754.1* | 663 | 9 | 34 | 0.718265693 | 2.87837943 | 2.002667267 | 4.75E-05 | 0.000325462 |
| *LOC_Os04g51040.1* | 2283 | 9 | 34 | 0.208589643 | 0.835902568 | 2.002667267 | 4.75E-05 | 0.000325525 |
| *LOC_Os06g09310.1* | 966 | 13 | 49 | 0.712069474 | 2.847092697 | 1.999399554 | 9.67E-07 | 8.78E-06 |
| *LOC_Os06g38990.1* | 3219 | 43 | 162 | 0.706811523 | 2.824728368 | 1.998714676 | 3.16E-19 | 8.16E-18 |
| *LOC_Os06g12320.1* | 1242 | 459 | 1729 | 19.55452325 | 78.13687734 | 1.998501238 | 1.99E-189 | 7.12E-187 |
| *LOC_Os02g43930.3* | 1263 | 8 | 30 | 0.335152744 | 1.333216125 | 1.992020023 | 0.000144221 | 0.000896247 |
| *LOC_Os10g38590.1* | 723 | 16 | 60 | 1.17094859 | 4.657958413 | 1.992020023 | 6.08E-08 | 6.52E-07 |
| *LOC_Os11g05640.1* | 1185 | 8 | 30 | 0.35721343 | 1.420972123 | 1.992020023 | 0.000144221 | 0.000896404 |
| *LOC_Os01g06890.1* | 2289 | 12 | 45 | 0.277390508 | 1.103441655 | 1.992020023 | 2.89E-06 | 2.44E-05 |
| *LOC_Os01g48360.2* | 267 | 8 | 30 | 1.58538545 | 6.306561671 | 1.992020023 | 0.000144221 | 0.000896562 |
| *LOC_Os03g52680.1* | 747 | 221 | 826 | 15.65408956 | 62.06433396 | 1.98722484 | 8.23E-91 | 1.25E-88 |
| *LOC_Os08g20130.1* | 1008 | 11 | 41 | 0.577415311 | 2.283000351 | 1.983249814 | 8.64E-06 | 6.76E-05 |
| *LOC_Os03g25990.1* | 795 | 20 | 74 | 1.331125519 | 5.22453021 | 1.972654698 | 2.16E-09 | 2.71E-08 |
| *LOC_Os05g49240.1* | 258 | 82 | 303 | 16.81706833 | 65.91823589 | 1.970751406 | 5.06E-34 | 2.40E-32 |
| *LOC_Os06g38890.1* | 2595 | 9 | 33 | 0.183510657 | 0.713771546 | 1.959598546 | 7.85E-05 | 0.000516868 |
| *LOC_Os12g40470.1* | 735 | 10 | 36 | 0.719894413 | 2.749146067 | 1.933126334 | 4.27E-05 | 0.000295142 |
| *LOC_Os02g52170.1* | 561 | 10 | 36 | 0.943177173 | 3.601822388 | 1.933126334 | 4.27E-05 | 0.0002952 |
| *LOC_Os03g62480.1* | 1431 | 12 | 43 | 0.443708506 | 1.68659759 | 1.926431682 | 7.78E-06 | 6.12E-05 |
| *LOC_Os09g19229.1* | 2565 | 16 | 57 | 0.330056854 | 1.247297753 | 1.918019442 | 2.66E-07 | 2.62E-06 |
| *LOC_Os02g07650.1* | 726 | 11 | 39 | 0.801700597 | 3.015161923 | 1.911100028 | 2.33E-05 | 0.000169495 |
| *LOC_Os09g20850.1* | 1338 | 13 | 46 | 0.514095002 | 1.929675896 | 1.908251666 | 4.26E-06 | 3.50E-05 |
| *LOC_Os10g18820.1* | 615 | 13 | 46 | 1.118470101 | 4.198221705 | 1.908251666 | 4.26E-06 | 3.50E-05 |
| *LOC_Os10g05020.1* | 1593 | 18 | 63 | 0.597878411 | 2.219767187 | 1.89248435 | 8.07E-08 | 8.49E-07 |
| *LOC_Os08g07730.1* | 1410 | 12 | 42 | 0.450316931 | 1.671909754 | 1.89248435 | 1.27E-05 | 9.62E-05 |
| *LOC_Os02g34410.1* | 1254 | 40 | 140 | 1.68779073 | 6.266328423 | 1.89248435 | 9.75E-16 | 2.06E-14 |
| *LOC_Os02g11680.1* | 1020 | 83 | 290 | 4.305603793 | 15.95807419 | 1.889999086 | 6.13E-31 | 2.62E-29 |
| *LOC_Os07g48450.1* | 1041 | 84 | 293 | 4.269575513 | 15.7979067 | 1.887568859 | 3.43E-31 | 1.48E-29 |
| *LOC_Os01g02770.1* | 1992 | 152 | 528 | 4.037480114 | 14.87740693 | 1.881596034 | 1.16E-54 | 9.37E-53 |
| *LOC_Os02g37330.1* | 402 | 11 | 38 | 1.447847346 | 5.305669546 | 1.873625322 | 3.79E-05 | 0.000264844 |
| *LOC_Os02g11640.1* | 1506 | 158 | 545 | 5.551217678 | 20.31206998 | 1.871461099 | 5.78E-56 | 4.79E-54 |
| *LOC_Os11g03440.1* | 927 | 69 | 238 | 3.938451476 | 14.41052744 | 1.871422734 | 2.37E-25 | 8.19E-24 |
| *LOC_Os03g20330.1* | 534 | 70 | 240 | 6.936061343 | 25.22624669 | 1.862737006 | 2.12E-25 | 7.34E-24 |
| *LOC_Os03g17250.1* | 1698 | 14 | 48 | 0.436261102 | 1.586668519 | 1.862737006 | 3.78E-06 | 3.13E-05 |
| *LOC_Os02g15560.1* | 2226 | 14 | 48 | 0.33278138 | 1.210315879 | 1.862737006 | 3.78E-06 | 3.13E-05 |
| *LOC_Os07g09490.1* | 1500 | 12 | 41 | 0.423297915 | 1.534176236 | 1.857718932 | 2.06E-05 | 0.000150943 |
| *LOC_Os04g39290.1* | 669 | 17 | 58 | 1.344556158 | 4.866139215 | 1.855647582 | 3.83E-07 | 3.70E-06 |
| *LOC_Os11g10470.1* | 306 | 25 | 85 | 4.322895375 | 15.59122191 | 1.850664174 | 7.76E-10 | 1.01E-08 |
| *LOC_Os03g60080.1* | 951 | 390 | 1325 | 21.69902562 | 78.20202788 | 1.849575758 | 2.20E-131 | 4.98E-129 |
| *LOC_Os02g47920.1* | 1011 | 19 | 64 | 0.994394212 | 3.553133064 | 1.837201914 | 1.15E-07 | 1.18E-06 |
| *LOC_Os02g13290.1* | 891 | 19 | 64 | 1.128319358 | 4.031669504 | 1.837201914 | 1.15E-07 | 1.18E-06 |
| *LOC_Os10g40530.1* | 399 | 41 | 138 | 5.437097281 | 19.41282969 | 1.83610188 | 5.75E-15 | 1.16E-13 |
| *LOC_Os02g06470.1* | 1248 | 11 | 37 | 0.466373905 | 1.664063108 | 1.835151175 | 6.14E-05 | 0.000412338 |
| *LOC_Os08g29660.1* | 960 | 85 | 285 | 4.684937863 | 16.66311842 | 1.830556601 | 3.34E-29 | 1.33E-27 |
| *LOC_Os01g52980.1* | 1086 | 46 | 154 | 2.241218243 | 7.959275715 | 1.828354012 | 1.90E-16 | 4.23E-15 |
| *LOC_Os06g27820.1* | 684 | 12 | 40 | 0.928284902 | 3.282362507 | 1.822095022 | 3.32E-05 | 0.000235051 |
| *LOC_Os02g45930.1* | 528 | 130 | 430 | 13.0276347 | 45.71062787 | 1.810954464 | 1.26E-42 | 7.84E-41 |
| *LOC_Os07g46060.1* | 3369 | 17 | 56 | 0.266995568 | 0.932974276 | 1.805021508 | 9.89E-07 | 8.96E-06 |
| *LOC_Os07g08160.1* | 579 | 541 | 1772 | 49.4395881 | 171.778105 | 1.796807532 | 2.82E-168 | 8.75E-166 |
| *LOC_Os02g53280.1* | 1098 | 11 | 36 | 0.530086187 | 1.840275373 | 1.79562281 | 9.90E-05 | 0.000637118 |
| *LOC_Os01g52514.1* | 1209 | 27 | 87 | 1.181662914 | 4.039016296 | 1.773185421 | 1.52E-09 | 1.94E-08 |
| *LOC_Os07g38450.1* | 1914 | 14 | 45 | 0.387027874 | 1.319633202 | 1.769627602 | 1.57E-05 | 0.000117003 |
| *LOC_Os04g34050.1* | 900 | 14 | 45 | 0.823079279 | 2.806419944 | 1.769627602 | 1.57E-05 | 0.000117028 |
| *LOC_Os03g59120.1* | 1578 | 19 | 61 | 0.63709287 | 2.169728981 | 1.767939252 | 4.71E-07 | 4.48E-06 |
| *LOC_Os11g30240.1* | 1758 | 19 | 61 | 0.571861518 | 1.94757243 | 1.767939252 | 4.71E-07 | 4.48E-06 |
| *LOC_Os02g37300.1* | 366 | 39 | 125 | 5.638189443 | 19.16953514 | 1.765511493 | 4.80E-13 | 8.44E-12 |
| *LOC_Os01g07600.1* | 1215 | 11 | 35 | 0.47904085 | 1.616867457 | 1.754980826 | 0.000158695 | 0.000978461 |
| *LOC_Os01g47570.1* | 546 | 29 | 92 | 2.810357037 | 9.457532411 | 1.750710389 | 7.30E-10 | 9.56E-09 |
| *LOC_Os03g08930.1* | 990 | 18 | 57 | 0.962040716 | 3.231635087 | 1.74809444 | 1.37E-06 | 1.22E-05 |
| *LOC_Os03g40194.1* | 2526 | 30 | 95 | 0.628411394 | 2.110925532 | 1.74809444 | 4.01E-10 | 5.41E-09 |
| *LOC_Os07g49080.1* | 2028 | 13 | 41 | 0.339181022 | 1.134745737 | 1.742241714 | 4.61E-05 | 0.000316812 |
| *LOC_Os03g43880.1* | 1326 | 119 | 375 | 4.748534304 | 15.87341597 | 1.74105845 | 1.77E-35 | 8.85E-34 |
| *LOC_Os04g46220.1* | 957 | 149 | 469 | 8.238164753 | 27.50702097 | 1.73940502 | 6.70E-44 | 4.30E-42 |
| *LOC_Os09g28370.1* | 6942 | 22 | 69 | 0.167685 | 0.557888148 | 1.734222266 | 1.22E-07 | 1.25E-06 |
| *LOC_Os03g28270.1* | 4275 | 358 | 1122 | 4.431013263 | 14.73124293 | 1.733170611 | 4.55E-102 | 7.77E-100 |
| *LOC_Os01g31370.1* | 1611 | 15 | 47 | 0.492665171 | 1.63751381 | 1.732827684 | 1.35E-05 | 0.00010211 |
| *LOC_Os02g09790.1* | 2742 | 30 | 94 | 0.578908527 | 1.924168306 | 1.732827684 | 6.33E-10 | 8.36E-09 |
| *LOC_Os03g54130.1* | 1053 | 740 | 2313 | 37.18428979 | 123.2905856 | 1.729297518 | 1.03E-207 | 4.06E-205 |
| *LOC_Os07g36610.1* | 2655 | 16 | 50 | 0.318868486 | 1.057031994 | 1.728985617 | 7.33E-06 | 5.79E-05 |
| *LOC_Os01g40070.1* | 1656 | 388 | 1210 | 12.39731213 | 41.01169242 | 1.726007918 | 2.68E-109 | 4.94E-107 |
| *LOC_Os05g24520.1* | 1497 | 17 | 53 | 0.600873794 | 1.987177783 | 1.725587041 | 3.98E-06 | 3.29E-05 |
| *LOC_Os07g48550.1* | 906 | 580 | 1805 | 33.87317753 | 111.8231346 | 1.723003459 | 1.05E-161 | 2.97E-159 |
| *LOC_Os03g31430.1* | 1836 | 37 | 115 | 1.066314192 | 3.515667686 | 1.721166113 | 9.69E-12 | 1.54E-10 |
| *LOC_Os02g43790.1* | 912 | 38 | 118 | 2.204676641 | 7.262227048 | 1.719844964 | 5.34E-12 | 8.68E-11 |
| *LOC_Os11g09820.1* | 2469 | 171 | 531 | 3.664638694 | 12.07135674 | 1.719844964 | 1.00E-48 | 7.19E-47 |
| *LOC_Os07g45180.1* | 2793 | 32 | 99 | 0.606226874 | 1.989513601 | 1.714486048 | 3.01E-10 | 4.12E-09 |
| *LOC_Os12g38170.1* | 702 | 322 | 994 | 24.27028644 | 79.47525425 | 1.711314591 | 4.66E-89 | 6.90E-87 |
| *LOC_Os11g31540.1* | 753 | 12 | 37 | 0.843222938 | 2.757969135 | 1.709620293 | 0.000135879 | 0.00084903 |
| *LOC_Os03g58850.1* | 411 | 12 | 37 | 1.544882902 | 5.052921553 | 1.709620293 | 0.000135879 | 0.00084888 |
| *LOC_Os01g74040.1* | 1005 | 50 | 154 | 2.632449721 | 8.600769579 | 1.708059779 | 4.27E-15 | 8.71E-14 |
| *LOC_Os03g04480.1* | 1308 | 13 | 40 | 0.525886171 | 1.716464797 | 1.706617804 | 7.32E-05 | 0.000484342 |
| *LOC_Os01g02370.1* | 1278 | 13 | 40 | 0.538230917 | 1.756757398 | 1.706617804 | 7.32E-05 | 0.000484251 |
| *LOC_Os01g74370.1* | 1029 | 13 | 40 | 0.668473384 | 2.181861958 | 1.706617804 | 7.32E-05 | 0.000484432 |
| *LOC_Os03g19070.1* | 645 | 83 | 255 | 6.808861813 | 22.19029723 | 1.704443433 | 5.94E-24 | 1.92E-22 |
| *LOC_Os03g20440.1* | 648 | 30 | 92 | 2.449640712 | 7.968846754 | 1.701800788 | 1.56E-09 | 1.99E-08 |
| *LOC_Os05g36350.1* | 1656 | 17 | 52 | 0.543181201 | 1.762485955 | 1.698106305 | 6.29E-06 | 5.02E-05 |
| *LOC_Os02g44310.1* | 402 | 38 | 115 | 5.00165447 | 16.05663152 | 1.682691965 | 2.04E-11 | 3.13E-10 |
| *LOC_Os01g66010.1* | 1467 | 81 | 245 | 2.92153469 | 9.37386348 | 1.681917364 | 1.14E-22 | 3.51E-21 |
| *LOC_Os06g35940.1* | 522 | 51 | 154 | 5.169586607 | 16.55895293 | 1.679490626 | 8.96E-15 | 1.78E-13 |
| *LOC_Os04g45330.1* | 801 | 52 | 157 | 3.435001808 | 11.00144647 | 1.679310458 | 4.95E-15 | 1.01E-13 |
| *LOC_Os03g31044.2* | 1851 | 55 | 166 | 1.57221673 | 5.033665161 | 1.678809145 | 8.36E-16 | 1.78E-14 |
| *LOC_Os12g36880.1* | 477 | 106 | 319 | 11.75827542 | 37.53660218 | 1.674621587 | 7.06E-29 | 2.78E-27 |
| *LOC_Os04g54370.1* | 1341 | 20 | 60 | 0.789146001 | 2.511337757 | 1.670091928 | 1.59E-06 | 1.40E-05 |
| *LOC_Os03g60500.1* | 702 | 16 | 48 | 1.205976966 | 3.837839239 | 1.670091928 | 1.82E-05 | 0.000134696 |
| *LOC_Os12g39400.1* | 744 | 13 | 39 | 0.924541817 | 2.942214457 | 1.670091928 | 0.00011556 | 0.000732805 |
| *LOC_Os11g41500.1* | 471 | 21 | 63 | 2.359144431 | 7.507620232 | 1.670091928 | 8.66E-07 | 7.91E-06 |
| *LOC_Os08g14600.1* | 702 | 18 | 54 | 1.356724087 | 4.317569144 | 1.670091928 | 5.37E-06 | 4.33E-05 |
| *LOC_Os07g46910.1* | 888 | 27 | 81 | 1.60881809 | 5.119820168 | 1.670091928 | 2.31E-08 | 2.60E-07 |
| *LOC_Os06g36920.1* | 1650 | 14 | 42 | 0.448952334 | 1.42872288 | 1.670091928 | 6.23E-05 | 0.000417924 |
| *LOC_Os01g62100.1* | 5409 | 40 | 120 | 0.391290363 | 1.245222382 | 1.670091928 | 9.67E-12 | 1.53E-10 |
| *LOC_Os06g07220.1* | 741 | 127 | 380 | 9.068629423 | 28.7837943 | 1.666300349 | 7.31E-34 | 3.43E-32 |
| *LOC_Os01g60640.1* | 843 | 39 | 116 | 2.447897196 | 7.723480747 | 1.657708204 | 2.73E-11 | 4.13E-10 |
| *LOC_Os01g61080.1* | 1668 | 32 | 95 | 1.015102914 | 3.196761327 | 1.654985036 | 1.79E-09 | 2.26E-08 |
| *LOC_Os01g22900.1* | 1887 | 841 | 2492 | 23.58197845 | 74.12399046 | 1.65225579 | 1.05E-209 | 4.19E-207 |
| *LOC_Os09g09540.1* | 1812 | 21 | 62 | 0.613221317 | 1.920508129 | 1.647008315 | 1.35E-06 | 1.20E-05 |
| *LOC_Os01g01260.1* | 6084 | 20 | 59 | 0.173938986 | 0.544308931 | 1.645844382 | 2.48E-06 | 2.12E-05 |
| *LOC_Os10g20380.1* | 609 | 37 | 109 | 3.214700915 | 10.04596959 | 1.643860387 | 1.40E-10 | 1.98E-09 |
| *LOC_Os01g41240.1* | 909 | 35 | 103 | 2.037324949 | 6.359983591 | 1.642346938 | 4.61E-10 | 6.18E-09 |
| *LOC_Os01g28790.1* | 354 | 151 | 444 | 22.56991002 | 70.39833079 | 1.641140555 | 1.77E-38 | 9.58E-37 |
| *LOC_Os08g13699.1* | 309 | 15 | 44 | 2.56855531 | 7.992393367 | 1.637670451 | 5.28E-05 | 0.000358388 |
| *LOC_Os03g59180.1* | 789 | 78 | 227 | 5.230867772 | 16.14847471 | 1.626275696 | 3.38E-20 | 9.21E-19 |
| *LOC_Os03g25120.1* | 918 | 21 | 61 | 1.210410705 | 3.729664849 | 1.623549342 | 2.10E-06 | 1.82E-05 |
| *LOC_Os03g19600.1* | 1773 | 20 | 58 | 0.596866773 | 1.836123596 | 1.621182328 | 3.86E-06 | 3.20E-05 |
| *LOC_Os04g34320.1* | 1989 | 178 | 515 | 4.735233088 | 14.53299418 | 1.617824619 | 1.72E-43 | 1.09E-41 |
| *LOC_Os07g44850.1* | 1056 | 18 | 52 | 0.901913171 | 2.76389843 | 1.615644144 | 1.31E-05 | 9.90E-05 |
| *LOC_Os08g10310.1* | 3075 | 17 | 49 | 0.292522949 | 0.894403754 | 1.612376431 | 2.41E-05 | 0.000175007 |
| *LOC_Os01g17050.1* | 708 | 16 | 46 | 1.195756822 | 3.646760379 | 1.608691384 | 4.45E-05 | 0.000306507 |
| *LOC_Os04g46980.1* | 1392 | 85 | 244 | 3.230991629 | 9.838598654 | 1.606475829 | 3.03E-21 | 8.74E-20 |
| *LOC_Os04g01740.1* | 2112 | 22 | 63 | 0.55116916 | 1.674284626 | 1.602977732 | 1.78E-06 | 1.55E-05 |
| *LOC_Os09g14830.1* | 6084 | 43 | 123 | 0.373968819 | 1.134745737 | 1.601379178 | 2.21E-11 | 3.38E-10 |
| *LOC_Os04g05520.1* | 6084 | 14 | 40 | 0.12175729 | 0.369023004 | 1.5997026 | 0.000152493 | 0.000944008 |
| *LOC_Os07g41060.1* | 675 | 14 | 40 | 1.097439039 | 3.326127341 | 1.5997026 | 0.000152493 | 0.000943843 |
| *LOC_Os01g72140.1* | 696 | 28 | 80 | 2.128653309 | 6.451540101 | 1.5997026 | 7.34E-08 | 7.77E-07 |
| *LOC_Os08g31200.1* | 1494 | 26 | 74 | 0.920828798 | 2.780121497 | 1.594143075 | 2.45E-07 | 2.42E-06 |
| *LOC_Os10g37760.2* | 684 | 45 | 128 | 3.481068381 | 10.50356002 | 1.593276331 | 1.03E-11 | 1.63E-10 |
| *LOC_Os04g27060.1* | 1068 | 32 | 91 | 1.58538545 | 4.782475934 | 1.592924068 | 1.02E-08 | 1.20E-07 |
| *LOC_Os07g26750.1* | 555 | 19 | 54 | 1.811409997 | 5.461141512 | 1.592089416 | 1.10E-05 | 8.44E-05 |
| *LOC_Os02g52010.1* | 987 | 114 | 324 | 6.111444063 | 18.4251279 | 1.592089416 | 2.32E-27 | 8.65E-26 |
| *LOC_Os02g58170.1* | 2580 | 297 | 843 | 6.091060116 | 18.339628 | 1.590199128 | 2.12E-68 | 2.21E-66 |
| *LOC_Os03g22210.1* | 549 | 24 | 68 | 2.313103361 | 6.952151409 | 1.587629768 | 8.18E-07 | 7.50E-06 |
| *LOC_Os04g32940.1* | 3087 | 24 | 68 | 0.411368236 | 1.236388443 | 1.587629768 | 8.18E-07 | 7.49E-06 |
| *LOC_Os02g04130.1* | 768 | 36 | 102 | 2.480261221 | 7.454552976 | 1.587629768 | 1.44E-09 | 1.84E-08 |
| *LOC_Os04g32570.1* | 708 | 17 | 48 | 1.270491624 | 3.805315178 | 1.582629087 | 3.74E-05 | 0.000261418 |
| *LOC_Os06g10300.1* | 1557 | 17 | 48 | 0.577718735 | 1.730355264 | 1.582629087 | 3.74E-05 | 0.000261366 |
| *LOC_Os11g28910.1* | 2142 | 17 | 48 | 0.419938408 | 1.257779247 | 1.582629087 | 3.74E-05 | 0.000261314 |
| *LOC_Os02g57924.1* | 273 | 79 | 223 | 15.31160041 | 45.84847234 | 1.582248579 | 3.74E-19 | 9.65E-18 |
| *LOC_Os06g49185.1* | 1782 | 39 | 110 | 1.158011973 | 3.46471598 | 1.581086922 | 3.68E-10 | 4.98E-09 |
| *LOC_Os02g33400.1* | 1704 | 22 | 62 | 0.683139241 | 2.042230476 | 1.579894119 | 2.74E-06 | 2.32E-05 |
| *LOC_Os06g09220.1* | 1404 | 16 | 45 | 0.602988483 | 1.798987143 | 1.576982524 | 6.90E-05 | 0.000459308 |
| *LOC_Os01g16750.1* | 1236 | 16 | 45 | 0.684948083 | 2.043509668 | 1.576982524 | 6.90E-05 | 0.000459394 |
| *LOC_Os11g10590.1* | 267 | 31 | 87 | 6.143368618 | 18.28902885 | 1.573876613 | 2.87E-08 | 3.18E-07 |
| *LOC_Os02g10120.1* | 2781 | 8443 | 23661 | 160.6393517 | 477.5455037 | 1.571812857 | 0 | 0 |
| *LOC_Os04g46630.1* | 795 | 90 | 252 | 5.990064837 | 17.79164342 | 1.570556255 | 3.11E-21 | 8.95E-20 |
| *LOC_Os11g35040.1* | 1329 | 40 | 112 | 1.592542946 | 4.730158521 | 1.570556255 | 3.11E-10 | 4.25E-09 |
| *LOC_Os01g12690.1* | 1185 | 49 | 137 | 2.187932262 | 6.48910603 | 1.568451666 | 3.45E-12 | 5.69E-11 |
| *LOC_Os01g62670.1* | 1548 | 22 | 61 | 0.75198273 | 2.211777992 | 1.556435147 | 4.22E-06 | 3.47E-05 |
| *LOC_Os04g33520.1* | 1068 | 30 | 83 | 1.486298859 | 4.362038489 | 1.553278263 | 7.99E-08 | 8.41E-07 |
| *LOC_Os05g37520.1* | 1455 | 30 | 83 | 1.090974008 | 3.20182619 | 1.553278263 | 7.99E-08 | 8.42E-07 |
| *LOC_Os01g02760.1* | 2139 | 101 | 279 | 2.498427386 | 7.321095506 | 1.551039257 | 6.00E-23 | 1.88E-21 |
| *LOC_Os06g46920.1* | 1104 | 50 | 138 | 2.396387654 | 7.01604986 | 1.549797695 | 4.43E-12 | 7.25E-11 |
| *LOC_Os04g39350.1* | 369 | 89 | 245 | 12.76203064 | 37.2668231 | 1.546033936 | 3.00E-20 | 8.19E-19 |
| *LOC_Os06g13190.1* | 282 | 60 | 165 | 11.25792327 | 32.84108445 | 1.544561046 | 4.21E-14 | 8.03E-13 |
| *LOC_Os02g39510.1* | 765 | 20 | 55 | 1.38332652 | 4.035375083 | 1.544561046 | 1.42E-05 | 0.000106967 |
| *LOC_Os03g32100.1* | 1077 | 20 | 55 | 0.98258569 | 2.866352775 | 1.544561046 | 1.42E-05 | 0.00010699 |
| *LOC_Os05g51240.1* | 900 | 172 | 473 | 10.11211686 | 29.49859185 | 1.544561046 | 1.43E-37 | 7.51E-36 |
| *LOC_Os04g34290.1* | 2451 | 425 | 1167 | 9.174908911 | 26.72453753 | 1.542399242 | 5.83E-90 | 8.82E-88 |
| *LOC_Os06g08710.1* | 3081 | 54 | 148 | 0.927381021 | 2.696203516 | 1.539695291 | 9.60E-13 | 1.65E-11 |
| *LOC_Os06g36390.1* | 786 | 18 | 49 | 1.211730673 | 3.499098658 | 1.52991427 | 4.81E-05 | 0.000329114 |
| *LOC_Os07g40000.1* | 681 | 43 | 117 | 3.341007773 | 9.643205093 | 1.529229393 | 2.82E-10 | 3.87E-09 |
| *LOC_Os02g36150.1* | 1548 | 81 | 220 | 2.768663689 | 7.976904233 | 1.526639138 | 4.99E-18 | 1.21E-16 |
| *LOC_Os05g33090.1* | 552 | 35 | 95 | 3.354942715 | 9.659778792 | 1.525702019 | 1.42E-08 | 1.64E-07 |
| *LOC_Os01g53730.1* | 528 | 854 | 2317 | 85.58153871 | 246.3058716 | 1.525079497 | 7.85E-174 | 2.53E-171 |
| *LOC_Os04g54180.1* | 2346 | 52 | 141 | 1.17282031 | 3.373445968 | 1.524241062 | 4.75E-12 | 7.75E-11 |
| *LOC_Os11g40210.1* | 1482 | 45 | 122 | 1.606646945 | 4.620556453 | 1.524013669 | 1.30E-10 | 1.85E-09 |
| *LOC_Os05g42150.1* | 1890 | 55 | 149 | 1.539774162 | 4.424937266 | 1.522938235 | 1.22E-12 | 2.09E-11 |
| *LOC_Os05g50100.1* | 531 | 17 | 46 | 1.693988832 | 4.862347172 | 1.521228542 | 8.86E-05 | 0.000575604 |
| *LOC_Os07g35810.1* | 1836 | 20 | 54 | 0.57638605 | 1.650835261 | 1.518088835 | 2.18E-05 | 0.000159105 |
| *LOC_Os03g07190.1* | 798 | 888 | 2394 | 58.87978519 | 168.3851966 | 1.515920998 | 5.77E-178 | 1.89E-175 |
| *LOC_Os09g25880.1* | 414 | 52 | 140 | 6.645981759 | 18.98061798 | 1.513972726 | 7.20E-12 | 1.15E-10 |
| *LOC_Os01g67810.1* | 1674 | 64 | 172 | 2.022929104 | 5.767075631 | 1.511394182 | 3.17E-14 | 6.11E-13 |
| *LOC_Os01g71270.1* | 1311 | 46 | 123 | 1.856569803 | 5.266051153 | 1.504081977 | 1.65E-10 | 2.32E-09 |
| *LOC_Os08g34300.1* | 3249 | 49 | 131 | 0.797999301 | 2.263102571 | 1.503842585 | 4.24E-11 | 6.30E-10 |
| *LOC_Os04g48490.1* | 1296 | 203 | 542 | 8.287951077 | 23.47345076 | 1.501942552 | 3.83E-41 | 2.25E-39 |
| *LOC_Os02g31860.1* | 1806 | 309 | 825 | 9.053090793 | 25.64004932 | 1.501916709 | 1.01E-61 | 9.44E-60 |
| *LOC_Os02g07370.1* | 1752 | 24 | 64 | 0.724825197 | 2.05035247 | 1.500166927 | 4.49E-06 | 3.67E-05 |
| *LOC_Os10g31330.1* | 645 | 18 | 48 | 1.476620634 | 4.176997126 | 1.500166927 | 7.35E-05 | 0.00048592 |
| *LOC_Os03g12730.1* | 3024 | 259 | 690 | 4.531835318 | 12.80707514 | 1.498773692 | 9.39E-52 | 7.19E-50 |
| *LOC_Os01g52690.1* | 1989 | 134 | 356 | 3.564726032 | 10.0461086 | 1.494773668 | 2.12E-27 | 7.91E-26 |
| *LOC_Os04g32960.1* | 3171 | 32 | 85 | 0.533961419 | 1.504545539 | 1.494520364 | 1.29E-07 | 1.32E-06 |
| *LOC_Os01g64470.1* | 717 | 26 | 69 | 1.918714399 | 5.401477716 | 1.493214166 | 2.05E-06 | 1.77E-05 |
| *LOC_Os02g45710.1* | 639 | 179 | 475 | 14.82205141 | 41.72298821 | 1.493097354 | 5.83E-36 | 2.95E-34 |
| *LOC_Os08g07080.1* | 1512 | 57 | 151 | 1.994707437 | 5.605415496 | 1.490644153 | 1.95E-12 | 3.28E-11 |
| *LOC_Os06g28630.1* | 255 | 34 | 90 | 7.054965252 | 19.81002313 | 1.489519683 | 5.93E-08 | 6.36E-07 |
| *LOC_Os03g17870.1* | 219 | 151 | 399 | 36.48286825 | 102.2613295 | 1.486969625 | 2.72E-30 | 1.13E-28 |
| *LOC_Os02g09480.1* | 906 | 173 | 457 | 10.10355123 | 28.31200694 | 1.486551555 | 1.91E-34 | 9.22E-33 |
| *LOC_Os02g07930.1* | 669 | 55 | 145 | 4.350034628 | 12.16534804 | 1.483678804 | 6.34E-12 | 1.02E-10 |
| *LOC_Os12g42200.1* | 2409 | 19 | 50 | 0.417323598 | 1.164972995 | 1.481058104 | 6.08E-05 | 0.000409018 |
| *LOC_Os01g42234.1* | 1599 | 56 | 147 | 1.853086558 | 5.16002166 | 1.47744685 | 5.29E-12 | 8.60E-11 |
| *LOC_Os02g06960.1* | 1221 | 29 | 76 | 1.256719854 | 3.493659553 | 1.475075946 | 7.76E-07 | 7.14E-06 |
| *LOC_Os04g54210.1* | 285 | 21 | 55 | 3.898796587 | 10.83179627 | 1.474171718 | 2.75E-05 | 0.000197273 |
| *LOC_Os09g26580.1* | 630 | 138 | 361 | 11.59030006 | 32.16246348 | 1.472459998 | 3.49E-27 | 1.30E-25 |
| *LOC_Os09g21180.1* | 1002 | 39 | 102 | 2.059458419 | 5.713669347 | 1.472152551 | 1.04E-08 | 1.22E-07 |
| *LOC_Os04g57880.1* | 1461 | 1162 | 3037 | 42.08351962 | 116.6748442 | 1.471166267 | 1.79E-215 | 7.71E-213 |
| *LOC_Os02g54900.1* | 1917 | 62 | 162 | 1.711298301 | 4.743244975 | 1.47078312 | 5.27E-13 | 9.22E-12 |
| *LOC_Os01g01840.1* | 1089 | 73 | 190 | 3.546917792 | 9.792833596 | 1.465160477 | 6.31E-15 | 1.27E-13 |
| *LOC_Os02g36590.3* | 1968 | 20 | 52 | 0.53772601 | 1.48306745 | 1.463641051 | 5.03E-05 | 0.000343237 |
| *LOC_Os07g30610.1* | 1449 | 92 | 239 | 3.359507263 | 9.257893258 | 1.46243428 | 2.40E-18 | 5.90E-17 |
| *LOC_Os04g51460.1* | 843 | 87 | 226 | 5.460693745 | 15.04747111 | 1.462364894 | 1.98E-17 | 4.66E-16 |
| *LOC_Os04g04390.1* | 5175 | 42 | 109 | 0.429432668 | 1.182221348 | 1.46099633 | 3.99E-09 | 4.88E-08 |
| *LOC_Os01g03310.1* | 582 | 227 | 589 | 20.63759165 | 56.80348271 | 1.460704764 | 8.06E-43 | 5.04E-41 |
| *LOC_Os08g07720.1* | 1449 | 27 | 70 | 0.985942349 | 2.711516854 | 1.459524942 | 2.56E-06 | 2.18E-05 |
| *LOC_Os05g44770.1* | 3141 | 297 | 769 | 5.003163037 | 13.7417188 | 1.457650095 | 3.27E-55 | 2.66E-53 |
| *LOC_Os09g06220.1* | 1572 | 34 | 88 | 1.144412302 | 3.142047774 | 1.457098205 | 1.35E-07 | 1.37E-06 |
| *LOC_Os02g57930.1* | 4686 | 86 | 221 | 0.971073962 | 2.647113989 | 1.446767232 | 7.90E-17 | 1.80E-15 |
| *LOC_Os06g39370.1* | 1146 | 438 | 1118 | 20.22300249 | 54.75702438 | 1.437046841 | 8.23E-78 | 1.00E-75 |
| *LOC_Os02g13304.1* | 390 | 20 | 51 | 2.713448174 | 7.339867545 | 1.435626675 | 7.59E-05 | 0.00050107 |
| *LOC_Os03g43970.1* | 1329 | 31 | 79 | 1.234220783 | 3.336451099 | 1.434713865 | 8.00E-07 | 7.34E-06 |
| *LOC_Os11g10120.1* | 807 | 24 | 61 | 1.573598197 | 4.242667077 | 1.430904264 | 1.55E-05 | 0.000115623 |
| *LOC_Os02g13510.1* | 3060 | 917 | 2329 | 15.85638024 | 42.71994803 | 1.429846428 | 7.61E-159 | 2.11E-156 |
| *LOC_Os02g32620.1* | 1569 | 41 | 104 | 1.382665274 | 3.720429244 | 1.428017141 | 1.61E-08 | 1.85E-07 |
| *LOC_Os02g57770.1* | 984 | 73 | 185 | 3.925399873 | 10.55259532 | 1.426686329 | 4.65E-14 | 8.82E-13 |
| *LOC_Os07g08150.1* | 603 | 105 | 266 | 9.213574023 | 24.75979121 | 1.426166345 | 1.48E-19 | 3.90E-18 |
| *LOC_Os03g14910.1* | 486 | 57 | 144 | 6.205756472 | 16.6306367 | 1.422164415 | 3.27E-11 | 4.91E-10 |
| *LOC_Os11g35220.1* | 1134 | 38 | 96 | 1.773073278 | 4.751610487 | 1.422164415 | 6.33E-08 | 6.76E-07 |
| *LOC_Os09g33850.1* | 522 | 21 | 53 | 2.128653309 | 5.698860422 | 1.420732459 | 6.25E-05 | 0.000418779 |
| *LOC_Os03g30910.1* | 2325 | 21 | 53 | 0.477917001 | 1.279486082 | 1.420732459 | 6.25E-05 | 0.0004187 |
| *LOC_Os07g35680.1* | 2097 | 227 | 572 | 5.727743606 | 15.31017842 | 1.418452277 | 6.62E-40 | 3.75E-38 |
| *LOC_Os06g50230.1* | 459 | 336 | 843 | 38.73314256 | 103.0854907 | 1.412200826 | 1.27E-57 | 1.08E-55 |
| *LOC_Os10g34602.1* | 1146 | 42 | 105 | 1.93919202 | 5.142654347 | 1.407057523 | 1.98E-08 | 2.25E-07 |
| *LOC_Os01g51670.1* | 507 | 26 | 65 | 2.713448174 | 7.195948574 | 1.407057523 | 1.05E-05 | 8.07E-05 |
| *LOC_Os07g08669.1* | 207 | 20 | 50 | 5.112293661 | 13.55758427 | 1.407057523 | 0.000114117 | 0.000724566 |
| *LOC_Os01g45840.1* | 759 | 34 | 85 | 2.370245243 | 6.28578907 | 1.407057523 | 4.50E-07 | 4.30E-06 |
| *LOC_Os05g08480.1* | 1635 | 58 | 145 | 1.877009104 | 4.977747913 | 1.407057523 | 4.02E-11 | 6.00E-10 |
| *LOC_Os02g28074.3* | 627 | 20 | 50 | 1.68779073 | 4.475948874 | 1.407057523 | 0.000114117 | 0.000724436 |
| *LOC_Os07g02460.1* | 285 | 22 | 55 | 4.084453567 | 10.83179627 | 1.407057523 | 5.13E-05 | 0.000349641 |
| *LOC_Os09g16540.1* | 2172 | 20 | 50 | 0.487221357 | 1.292090214 | 1.407057523 | 0.000114117 | 0.000724696 |
| *LOC_Os07g35880.1* | 1803 | 199 | 496 | 5.840008674 | 15.44075754 | 1.402701117 | 2.87E-34 | 1.38E-32 |
| *LOC_Os01g65100.1* | 1638 | 249 | 620 | 8.043435658 | 21.2451815 | 1.401251901 | 2.36E-42 | 1.45E-40 |
| *LOC_Os11g25920.1* | 750 | 25 | 62 | 1.763741313 | 4.63994764 | 1.395469548 | 1.90E-05 | 0.000140269 |
| *LOC_Os06g21380.1* | 1572 | 5584 | 13808 | 187.9528911 | 493.0158598 | 1.391262951 | 0 | 0 |
| *LOC_Os07g03880.1* | 2079 | 140 | 346 | 3.563113764 | 9.341234253 | 1.390474638 | 4.33E-24 | 1.41E-22 |
| *LOC_Os01g21130.1* | 477 | 34 | 84 | 3.771522304 | 9.884246343 | 1.389984009 | 6.69E-07 | 6.22E-06 |
| *LOC_Os11g07580.1* | 360 | 94 | 232 | 13.81597362 | 36.17163483 | 1.388521571 | 1.27E-16 | 2.85E-15 |
| *LOC_Os08g24770.1* | 2151 | 155 | 382 | 3.812829896 | 9.967944384 | 1.38643385 | 2.53E-26 | 9.12E-25 |
| *LOC_Os05g09650.1* | 684 | 35 | 86 | 2.70749763 | 7.057079391 | 1.382111165 | 5.51E-07 | 5.18E-06 |
| *LOC_Os03g47810.1* | 1920 | 35 | 86 | 0.964546031 | 2.514084533 | 1.382111165 | 5.51E-07 | 5.18E-06 |
| *LOC_Os01g02580.1* | 1941 | 137 | 336 | 3.734660895 | 9.716198878 | 1.379414767 | 3.56E-23 | 1.12E-21 |
| *LOC_Os04g44950.1* | 924 | 1598 | 3913 | 91.50839669 | 237.6952649 | 1.37713713 | 7.14E-251 | 3.95E-248 |
| *LOC_Os02g56080.1* | 825 | 29 | 71 | 1.859945385 | 4.830444025 | 1.376895552 | 5.82E-06 | 4.67E-05 |
| *LOC_Os01g53750.1* | 1425 | 207 | 506 | 7.686198985 | 19.93050514 | 1.374636045 | 6.44E-34 | 3.03E-32 |
| *LOC_Os01g26039.1* | 2097 | 1226 | 2994 | 30.93486194 | 80.13754232 | 1.37324467 | 1.29E-191 | 4.67E-189 |
| *LOC_Os09g07320.1* | 573 | 34 | 83 | 3.139644222 | 8.130291635 | 1.372706018 | 9.91E-07 | 8.97E-06 |
| *LOC_Os01g69840.1* | 255 | 200 | 488 | 41.4997956 | 107.4143477 | 1.372010575 | 1.12E-32 | 5.06E-31 |
| *LOC_Os07g45970.1* | 5211 | 23 | 56 | 0.233540876 | 0.603183715 | 1.368922394 | 6.27E-05 | 0.000420402 |
| *LOC_Os01g71310.1* | 1590 | 83 | 201 | 2.762085452 | 7.095476839 | 1.361141687 | 3.31E-14 | 6.36E-13 |
| *LOC_Os04g23220.1* | 3195 | 74 | 179 | 1.225510396 | 3.144595743 | 1.359491839 | 8.66E-13 | 1.49E-11 |
| *LOC_Os03g59720.1* | 297 | 103 | 249 | 18.35003588 | 47.05714249 | 1.358630833 | 3.34E-17 | 7.77E-16 |
| *LOC_Os03g60580.1* | 453 | 36 | 87 | 4.204946177 | 10.77962627 | 1.358147922 | 6.69E-07 | 6.21E-06 |
| *LOC_Os02g53180.2* | 966 | 128 | 309 | 7.011145592 | 17.95411517 | 1.356592456 | 6.15E-21 | 1.74E-19 |
| *LOC_Os10g09550.1* | 1839 | 29 | 70 | 0.83439638 | 2.136480653 | 1.356431449 | 8.61E-06 | 6.74E-05 |
| *LOC_Os04g54630.1* | 585 | 318 | 767 | 28.76255064 | 73.59056742 | 1.35532924 | 1.71E-49 | 1.24E-47 |
| *LOC_Os03g53020.1* | 900 | 85 | 205 | 4.997267053 | 12.78480197 | 1.355218591 | 2.25E-14 | 4.37E-13 |
| *LOC_Os05g37530.1* | 438 | 39 | 94 | 4.711363781 | 12.04582076 | 1.35431606 | 2.51E-07 | 2.48E-06 |
| *LOC_Os01g02840.1* | 1911 | 37 | 89 | 1.024465127 | 2.614038462 | 1.351409493 | 5.49E-07 | 5.17E-06 |
| *LOC_Os02g33680.1* | 1518 | 42 | 101 | 1.463975003 | 3.734498212 | 1.351023488 | 9.46E-08 | 9.84E-07 |
| *LOC_Os04g31550.1* | 396 | 80 | 192 | 10.68934129 | 27.21376915 | 1.348163833 | 1.85E-13 | 3.38E-12 |
| *LOC_Os11g08020.1* | 1536 | 30 | 72 | 1.033442176 | 2.631018697 | 1.348163833 | 7.05E-06 | 5.59E-05 |
| *LOC_Os12g16340.1* | 2688 | 48 | 115 | 0.944861418 | 2.401326589 | 1.345656978 | 1.35E-08 | 1.56E-07 |
| *LOC_Os01g07610.1* | 1047 | 38 | 91 | 1.920406014 | 4.87839952 | 1.344996554 | 4.50E-07 | 4.30E-06 |
| *LOC_Os01g28450.1* | 495 | 28 | 67 | 2.993015561 | 7.597177222 | 1.343863696 | 1.55E-05 | 0.000116097 |
| *LOC_Os05g03760.1* | 1806 | 2281 | 5453 | 66.82880291 | 169.4729563 | 1.342513136 | 0 | 0 |
| *LOC_Os05g43930.1* | 1104 | 85 | 203 | 4.073859011 | 10.32071103 | 1.341074409 | 4.81E-14 | 9.11E-13 |
| *LOC_Os04g35030.1* | 1218 | 34 | 81 | 1.477024745 | 3.732676773 | 1.337516589 | 2.16E-06 | 1.86E-05 |
| *LOC_Os11g10510.1* | 1140 | 29 | 69 | 1.346013107 | 3.397245195 | 1.335672889 | 1.27E-05 | 9.63E-05 |
| *LOC_Os01g71710.1* | 1089 | 58 | 138 | 2.818099068 | 7.112689665 | 1.335672889 | 6.03E-10 | 7.98E-09 |
| *LOC_Os03g59180.2* | 690 | 401 | 953 | 30.75044637 | 77.52226685 | 1.334003405 | 1.22E-59 | 1.08E-57 |
| *LOC_Os12g06480.1* | 2187 | 32 | 76 | 0.774207435 | 1.950506774 | 1.333056941 | 4.73E-06 | 3.86E-05 |
| *LOC_Os06g43090.1* | 936 | 275 | 653 | 15.54579683 | 39.15795349 | 1.332780801 | 2.34E-41 | 1.40E-39 |
| *LOC_Os05g27730.1* | 1464 | 1460 | 3465 | 52.76767043 | 132.8448785 | 1.332016411 | 2.16E-211 | 8.88E-209 |
| *LOC_Os07g47450.1* | 318 | 35 | 83 | 5.823674147 | 14.64986512 | 1.330885842 | 1.76E-06 | 1.54E-05 |
| *LOC_Os05g45410.1* | 1380 | 76 | 180 | 2.914007387 | 7.321095506 | 1.329055011 | 1.84E-12 | 3.09E-11 |
| *LOC_Os01g02830.1* | 2121 | 38 | 90 | 0.947979772 | 2.381685949 | 1.329055011 | 6.61E-07 | 6.15E-06 |
| *LOC_Os08g02070.1* | 669 | 186 | 440 | 14.7110262 | 36.91553887 | 1.32733033 | 3.09E-28 | 1.19E-26 |
| *LOC_Os08g05480.1* | 900 | 160 | 378 | 9.406620336 | 23.57392753 | 1.325443757 | 1.97E-24 | 6.53E-23 |
| *LOC_Os06g15750.1* | 3276 | 69 | 163 | 1.114451929 | 2.792713375 | 1.325333125 | 2.24E-11 | 3.41E-10 |
| *LOC_Os07g12810.1* | 1446 | 61 | 144 | 2.232120749 | 5.589550096 | 1.324317092 | 3.34E-10 | 4.55E-09 |
| *LOC_Os10g27040.1* | 3783 | 28 | 66 | 0.391631695 | 0.979242486 | 1.322168625 | 2.29E-05 | 0.000166765 |
| *LOC_Os01g10440.1* | 1443 | 31 | 73 | 1.136714776 | 2.83948241 | 1.320757676 | 8.49E-06 | 6.65E-05 |
| *LOC_Os03g32050.1* | 1008 | 48 | 113 | 2.519630447 | 6.292171699 | 1.320345889 | 2.89E-08 | 3.20E-07 |
| *LOC_Os10g38470.1* | 699 | 142 | 334 | 10.74898139 | 26.81957829 | 1.319086601 | 1.27E-21 | 3.74E-20 |
| *LOC_Os02g32520.1* | 2817 | 302 | 710 | 5.672522647 | 14.1466678 | 1.318399903 | 4.22E-44 | 2.72E-42 |
| *LOC_Os10g34700.1* | 1458 | 23 | 54 | 0.834692391 | 2.078829588 | 1.316454974 | 0.000137177 | 0.000856838 |
| *LOC_Os03g26704.1* | 3684 | 29 | 68 | 0.416518714 | 1.036029078 | 1.314611274 | 1.87E-05 | 0.000137824 |
| *LOC_Os09g24580.1* | 486 | 148 | 347 | 16.11319224 | 40.07521484 | 1.314467915 | 2.73E-22 | 8.29E-21 |
| *LOC_Os05g46830.1* | 681 | 44 | 103 | 3.418705629 | 8.489317304 | 1.312198336 | 1.36E-07 | 1.39E-06 |
| *LOC_Os10g25030.1* | 990 | 556 | 1298 | 29.71636879 | 73.59056742 | 1.308263023 | 3.07E-78 | 3.76E-76 |
| *LOC_Os11g38360.1* | 2565 | 24 | 56 | 0.495085281 | 1.225415336 | 1.307521849 | 0.000111665 | 0.000710782 |
| *LOC_Os04g43840.1* | 753 | 27 | 63 | 1.897251612 | 4.6960015 | 1.307521849 | 4.11E-05 | 0.000285408 |
| *LOC_Os08g20492.1* | 1206 | 61 | 142 | 2.676323883 | 6.608816451 | 1.30413921 | 7.08E-10 | 9.28E-09 |
| *LOC_Os02g02910.1* | 1956 | 104 | 242 | 2.813329702 | 6.944311108 | 1.303552947 | 8.08E-16 | 1.73E-14 |
| *LOC_Os04g17479.1* | 321 | 34 | 79 | 5.604411649 | 13.81353119 | 1.301447335 | 4.62E-06 | 3.78E-05 |
| *LOC_Os07g10420.1* | 690 | 216 | 501 | 16.56383146 | 40.75409831 | 1.298908719 | 6.29E-31 | 2.69E-29 |
| *LOC_Os04g52640.1* | 3069 | 47 | 109 | 0.810321033 | 1.993481746 | 1.298724901 | 7.48E-08 | 7.91E-07 |
| *LOC_Os11g41650.2* | 915 | 47 | 109 | 2.71789645 | 6.686333855 | 1.298724901 | 7.48E-08 | 7.91E-07 |
| *LOC_Os12g06480.2* | 2376 | 41 | 95 | 0.913047902 | 2.244191033 | 1.297433031 | 5.29E-07 | 4.99E-06 |
| *LOC_Os01g12440.1* | 1143 | 57 | 132 | 2.638668106 | 6.482019818 | 1.296633533 | 3.31E-09 | 4.07E-08 |
| *LOC_Os06g49750.1* | 1314 | 70 | 162 | 2.818764655 | 6.919939587 | 1.295696414 | 5.64E-11 | 8.29E-10 |
| *LOC_Os11g01330.1* | 3396 | 5342 | 12338 | 83.23238599 | 203.9199603 | 1.292786098 | 0 | 0 |
| *LOC_Os01g68524.1* | 2139 | 63 | 145 | 1.558425003 | 3.804870424 | 1.287758594 | 6.89E-10 | 9.05E-09 |
| *LOC_Os01g66020.1* | 1941 | 67 | 154 | 1.82644 | 4.453257819 | 1.285826778 | 2.15E-10 | 2.98E-09 |
| *LOC_Os02g45620.1* | 846 | 64 | 147 | 4.002817164 | 9.752806897 | 1.284801772 | 5.65E-10 | 7.49E-09 |
| *LOC_Os12g03150.1* | 936 | 126 | 289 | 7.122801456 | 17.33024282 | 1.282775187 | 3.53E-18 | 8.64E-17 |
| *LOC_Os05g34380.1* | 1410 | 116 | 266 | 4.353063666 | 10.58876177 | 1.282430868 | 7.60E-17 | 1.74E-15 |
| *LOC_Os03g14915.1* | 552 | 239 | 548 | 22.90946597 | 55.72167135 | 1.282294703 | 4.86E-33 | 2.23E-31 |
| *LOC_Os11g47200.1* | 3144 | 86 | 197 | 1.447344971 | 3.516951202 | 1.280916492 | 7.78E-13 | 1.35E-11 |
| *LOC_Os11g35060.1* | 2388 | 45 | 103 | 0.997089938 | 2.420948528 | 1.279776858 | 2.36E-07 | 2.33E-06 |
| *LOC_Os09g10054.1* | 2775 | 343 | 785 | 6.540143463 | 15.87776329 | 1.279613505 | 1.87E-46 | 1.29E-44 |
| *LOC_Os08g38700.1* | 993 | 1441 | 3288 | 76.78402514 | 185.8511334 | 1.275269391 | 1.05E-187 | 3.70E-185 |
| *LOC_Os01g70490.1* | 2313 | 97 | 221 | 2.218974155 | 5.362895007 | 1.273119145 | 4.21E-14 | 8.03E-13 |
| *LOC_Os10g18370.1* | 891 | 47 | 107 | 2.791105781 | 6.740447452 | 1.272007562 | 1.57E-07 | 1.58E-06 |
| *LOC_Os04g49739.1* | 1131 | 40 | 91 | 1.871343568 | 4.516078071 | 1.270995973 | 1.37E-06 | 1.21E-05 |
| *LOC_Os03g47530.1* | 1083 | 37 | 84 | 1.807712703 | 4.35344922 | 1.267993485 | 3.65E-06 | 3.03E-05 |
| *LOC_Os04g59160.1* | 1047 | 37 | 84 | 1.869869014 | 4.503138019 | 1.267993485 | 3.65E-06 | 3.03E-05 |
| *LOC_Os06g18850.1* | 3438 | 105 | 238 | 1.61599335 | 3.885561062 | 1.265701673 | 5.97E-15 | 1.20E-13 |
| *LOC_Os06g11150.1* | 1248 | 42 | 95 | 1.780700364 | 4.272594466 | 1.262667613 | 9.08E-07 | 8.28E-06 |
| *LOC_Os08g40850.1* | 1158 | 411 | 927 | 18.77973263 | 44.93180117 | 1.258560373 | 3.44E-53 | 2.72E-51 |
| *LOC_Os04g12830.1* | 1725 | 75 | 169 | 2.300532147 | 5.49895618 | 1.257190173 | 6.24E-11 | 9.13E-10 |
| *LOC_Os06g36320.1* | 2412 | 44 | 99 | 0.965231564 | 2.303777566 | 1.255054429 | 6.04E-07 | 5.65E-06 |
| *LOC_Os01g46770.1* | 6084 | 28 | 63 | 0.24351458 | 0.581211231 | 1.255054429 | 7.11E-05 | 0.00047233 |
| *LOC_Os12g26850.1* | 3591 | 142 | 319 | 2.092324699 | 4.986064952 | 1.252794922 | 3.06E-19 | 7.90E-18 |
| *LOC_Os09g31514.1* | 1044 | 203 | 456 | 10.28849099 | 24.51585238 | 1.252683525 | 7.90E-27 | 2.89E-25 |
| *LOC_Os04g52090.1* | 669 | 185 | 415 | 14.63193466 | 34.81806507 | 1.250715493 | 1.67E-24 | 5.56E-23 |
| *LOC_Os04g11440.1* | 720 | 46 | 103 | 3.380504183 | 8.029479284 | 1.248067999 | 4.02E-07 | 3.86E-06 |
| *LOC_Os04g01710.1* | 1152 | 708 | 1583 | 32.51898046 | 77.12782589 | 1.245969418 | 3.56E-88 | 5.15E-86 |
| *LOC_Os08g41000.1* | 1332 | 164 | 366 | 6.514720165 | 15.42266816 | 1.243277261 | 1.32E-21 | 3.86E-20 |
| *LOC_Os06g12640.1* | 2037 | 57 | 127 | 1.480607582 | 3.499414167 | 1.2409241 | 2.07E-08 | 2.34E-07 |
| *LOC_Os05g03920.1* | 2007 | 159 | 354 | 4.191851551 | 9.900076334 | 1.239852022 | 7.25E-21 | 2.04E-19 |
| *LOC_Os05g46000.1* | 621 | 142 | 316 | 12.099095 | 28.56131086 | 1.239163056 | 8.92E-19 | 2.24E-17 |
| *LOC_Os02g52270.1* | 399 | 58 | 129 | 7.69150347 | 18.14677558 | 1.238375688 | 1.69E-08 | 1.93E-07 |
| *LOC_Os08g37730.1* | 1092 | 36 | 80 | 1.74435954 | 4.111970614 | 1.237132521 | 9.36E-06 | 7.27E-05 |
| *LOC_Os05g47650.1* | 1185 | 36 | 80 | 1.607460437 | 3.789258996 | 1.237132521 | 9.36E-06 | 7.26E-05 |
| *LOC_Os06g13720.1* | 1197 | 32 | 71 | 1.414529374 | 3.3292534 | 1.234876547 | 3.09E-05 | 0.000219903 |
| *LOC_Os01g63690.1* | 1425 | 125 | 277 | 4.641424508 | 10.91057297 | 1.233087309 | 1.58E-16 | 3.53E-15 |
| *LOC_Os11g11790.1* | 2043 | 335 | 742 | 8.676260497 | 20.38535094 | 1.232387519 | 1.40E-41 | 8.41E-40 |
| *LOC_Os01g50820.1* | 1551 | 28 | 62 | 0.955217732 | 2.243688414 | 1.231970816 | 0.000102964 | 0.000659906 |
| *LOC_Os01g34790.1* | 753 | 89 | 197 | 6.25390346 | 14.68432215 | 1.231447816 | 3.73E-12 | 6.14E-11 |
| *LOC_Os10g04860.1* | 4077 | 353 | 780 | 4.581314816 | 10.73832502 | 1.228935368 | 1.94E-43 | 1.23E-41 |
| *LOC_Os08g01940.1* | 2787 | 72 | 159 | 1.366946981 | 3.202158386 | 1.228087381 | 4.81E-10 | 6.44E-09 |
| *LOC_Os10g34083.1* | 225 | 29 | 64 | 6.819799744 | 15.96541124 | 1.227148433 | 8.35E-05 | 0.000546225 |
| *LOC_Os10g37350.1* | 246 | 29 | 64 | 6.237621717 | 14.60251028 | 1.227148433 | 8.35E-05 | 0.000546124 |
| *LOC_Os04g44924.1* | 930 | 1406 | 3101 | 79.99420278 | 187.155016 | 1.226266359 | 1.11E-166 | 3.34E-164 |
| *LOC_Os11g04860.1* | 1449 | 990 | 2183 | 36.15121946 | 84.56058989 | 1.225941128 | 7.12E-118 | 1.44E-115 |
| *LOC_Os02g43010.1* | 1491 | 44 | 97 | 1.561461122 | 3.651545735 | 1.225610651 | 1.25E-06 | 1.12E-05 |
| *LOC_Os07g03860.1* | 1965 | 69 | 152 | 1.857987032 | 4.341738743 | 1.224532484 | 1.26E-09 | 1.62E-08 |
| *LOC_Os03g62200.1* | 1440 | 125 | 275 | 4.593076336 | 10.71896506 | 1.222632951 | 3.20E-16 | 7.02E-15 |
| *LOC_Os02g12890.1* | 1647 | 96 | 211 | 3.084137815 | 7.190705624 | 1.221266116 | 9.10E-13 | 1.57E-11 |
| *LOC_Os03g62340.1* | 2817 | 3374 | 7407 | 63.37447487 | 147.5836175 | 1.219558791 | 0 | 0 |
| *LOC_Os04g34410.1* | 2484 | 41 | 90 | 0.873350167 | 2.03363764 | 1.219430519 | 3.33E-06 | 2.78E-05 |
| *LOC_Os08g32970.1* | 966 | 36 | 79 | 1.971884698 | 4.590210674 | 1.218985174 | 1.35E-05 | 0.000101746 |
| *LOC_Os07g07290.1* | 630 | 36 | 79 | 3.023556537 | 7.038323034 | 1.218985174 | 1.35E-05 | 0.000101768 |
| *LOC_Os11g10280.1* | 2373 | 31 | 68 | 0.691226052 | 1.608399125 | 1.218395959 | 5.49E-05 | 0.000371978 |
| *LOC_Os08g40870.1* | 1182 | 62 | 136 | 2.775430493 | 6.458089888 | 1.218395959 | 1.07E-08 | 1.25E-07 |
| *LOC_Os08g42930.1* | 1461 | 31 | 68 | 1.122710076 | 2.612410078 | 1.218395959 | 5.49E-05 | 0.000372049 |
| *LOC_Os09g16510.1* | 1086 | 150 | 329 | 7.308320358 | 17.00390721 | 1.218254511 | 5.20E-19 | 1.33E-17 |
| *LOC_Os10g40360.1* | 1473 | 283 | 620 | 10.1657595 | 23.62498799 | 1.21659559 | 2.38E-34 | 1.15E-32 |
| *LOC_Os07g33270.1* | 591 | 142 | 311 | 12.71326226 | 29.53626404 | 1.216153078 | 5.22E-18 | 1.26E-16 |
| *LOC_Os07g42730.1* | 465 | 153 | 335 | 17.40983361 | 40.43658844 | 1.21575887 | 2.85E-19 | 7.37E-18 |
| *LOC_Os04g55500.1* | 1953 | 229 | 501 | 6.204251316 | 14.39852936 | 1.214592433 | 5.30E-28 | 2.02E-26 |
| *LOC_Os07g28480.1* | 699 | 571 | 1249 | 43.22301673 | 100.2923751 | 1.214340254 | 3.00E-67 | 3.08E-65 |
| *LOC_Os10g25040.2* | 978 | 131 | 286 | 7.087426748 | 16.41382626 | 1.211577763 | 1.36E-16 | 3.06E-15 |
| *LOC_Os08g42700.1* | 3681 | 608 | 1324 | 8.739647256 | 20.18853575 | 1.207889321 | 1.35E-70 | 1.45E-68 |
| *LOC_Os02g57090.1* | 3000 | 68 | 148 | 1.199344093 | 2.769001011 | 1.207119952 | 3.15E-09 | 3.88E-08 |
| *LOC_Os11g44380.1* | 666 | 29 | 63 | 2.3039864 | 5.309443137 | 1.204428356 | 0.000120134 | 0.000758546 |
| *LOC_Os10g34930.1* | 690 | 35 | 76 | 2.683954172 | 6.182258427 | 1.203773924 | 2.38E-05 | 0.000172899 |
| *LOC_Os03g18550.1* | 987 | 437 | 948 | 23.42720224 | 53.91055941 | 1.202383207 | 1.07E-50 | 8.01E-49 |
| *LOC_Os11g09110.1* | 1884 | 77 | 167 | 2.162549062 | 4.975288011 | 1.202047179 | 3.54E-10 | 4.80E-09 |
| *LOC_Os02g15120.1* | 600 | 89 | 193 | 7.848648843 | 18.05463497 | 1.201853034 | 1.53E-11 | 2.38E-10 |
| *LOC_Os07g03870.1* | 2283 | 259 | 561 | 6.002746387 | 13.79239237 | 1.200178101 | 1.32E-30 | 5.55E-29 |
| *LOC_Os02g02970.1* | 1794 | 31 | 67 | 0.914314059 | 2.096211106 | 1.197022308 | 7.88E-05 | 0.00051872 |
| *LOC_Os11g25780.1* | 645 | 367 | 793 | 30.10665404 | 69.00747335 | 1.19667023 | 2.20E-42 | 1.36E-40 |
| *LOC_Os05g45170.1* | 711 | 38 | 82 | 2.827939658 | 6.473317451 | 1.194753919 | 1.27E-05 | 9.63E-05 |
| *LOC_Os03g12670.1* | 234 | 45 | 97 | 10.17543065 | 23.26690039 | 1.193189173 | 2.08E-06 | 1.79E-05 |
| *LOC_Os01g02690.1* | 1926 | 34 | 73 | 0.934068608 | 2.127400373 | 1.187491145 | 4.19E-05 | 0.00029031 |
| *LOC_Os04g05650.1* | 585 | 70 | 150 | 6.331379072 | 14.39189715 | 1.184665101 | 4.22E-09 | 5.13E-08 |
| *LOC_Os01g18840.1* | 807 | 72 | 154 | 4.720794592 | 10.71099557 | 1.181990967 | 2.80E-09 | 3.47E-08 |
| *LOC_Os01g43851.1* | 1587 | 544 | 1159 | 18.13752881 | 40.99106131 | 1.176331437 | 1.54E-59 | 1.36E-57 |
| *LOC_Os01g72530.1* | 456 | 31 | 66 | 3.597103994 | 8.123847206 | 1.175327237 | 0.000112624 | 0.000716368 |
| *LOC_Os12g06870.1* | 2877 | 187 | 398 | 3.439203603 | 7.764721151 | 1.174859588 | 1.72E-21 | 5.00E-20 |
| *LOC_Os09g39060.1* | 852 | 676 | 1436 | 41.98201153 | 94.6013859 | 1.172090025 | 6.32E-73 | 7.11E-71 |
| *LOC_Os01g63480.1* | 1452 | 122 | 259 | 4.445794219 | 10.01188382 | 1.171200378 | 1.89E-14 | 3.69E-13 |
| *LOC_Os04g57200.1* | 414 | 90 | 191 | 11.50266074 | 25.89498595 | 1.170705159 | 5.00E-11 | 7.37E-10 |
| *LOC_Os04g44180.1* | 885 | 33 | 70 | 1.972998757 | 4.439534374 | 1.170018325 | 7.38E-05 | 0.000487517 |
| *LOC_Os09g24850.1* | 4086 | 42 | 89 | 0.543884986 | 1.222571586 | 1.168545436 | 7.84E-06 | 6.16E-05 |
| *LOC_Os02g35530.1* | 1176 | 84 | 178 | 3.779445671 | 8.495625 | 1.168545436 | 2.40E-10 | 3.31E-09 |
| *LOC_Os09g24490.1* | 1092 | 35 | 74 | 1.695905109 | 3.803572818 | 1.165299776 | 4.84E-05 | 0.000330896 |
| *LOC_Os03g21060.1* | 1179 | 237 | 501 | 10.63630257 | 23.85099901 | 1.165052972 | 2.60E-26 | 9.32E-25 |
| *LOC_Os03g14110.1* | 1200 | 223 | 471 | 9.83285782 | 22.03039656 | 1.163812777 | 8.59E-25 | 2.90E-23 |
| *LOC_Os09g23220.1* | 999 | 64 | 135 | 3.389773094 | 7.584918767 | 1.161945025 | 4.09E-08 | 4.46E-07 |
| *LOC_Os04g51450.1* | 960 | 55 | 116 | 3.031430382 | 6.782181531 | 1.161750709 | 3.70E-07 | 3.58E-06 |
| *LOC_Os03g11950.1* | 1317 | 95 | 200 | 3.816752272 | 8.523674848 | 1.159130009 | 2.54E-11 | 3.85E-10 |
| *LOC_Os01g49614.2* | 1287 | 38 | 80 | 1.562288342 | 3.488944763 | 1.159130009 | 2.57E-05 | 0.000185692 |
| *LOC_Os01g43010.2* | 2811 | 87 | 183 | 1.637625339 | 3.65403664 | 1.15788577 | 1.83E-10 | 2.56E-09 |
| *LOC_Os05g10930.1* | 417 | 185 | 389 | 23.47425488 | 52.35958552 | 1.157374312 | 1.40E-20 | 3.91E-19 |
| *LOC_Os02g28220.1* | 1305 | 50 | 105 | 2.027288865 | 4.516078071 | 1.155518756 | 1.48E-06 | 1.31E-05 |
| *LOC_Os06g14030.1* | 2577 | 293 | 615 | 6.016021009 | 13.39501952 | 1.154815173 | 1.67E-31 | 7.31E-30 |
| *LOC_Os02g03320.1* | 1683 | 102 | 214 | 3.206802387 | 7.136944361 | 1.154171072 | 6.07E-12 | 9.81E-11 |
| *LOC_Os07g10840.1* | 2292 | 561 | 1177 | 12.95103242 | 28.82335318 | 1.154171072 | 1.12E-58 | 9.59E-57 |
| *LOC_Os01g40870.1* | 1524 | 698 | 1464 | 24.23408339 | 53.91861939 | 1.15374604 | 1.67E-72 | 1.87E-70 |
| *LOC_Os01g57610.1* | 1833 | 52 | 109 | 1.501056437 | 3.337695296 | 1.152874034 | 9.74E-07 | 8.84E-06 |
| *LOC_Os02g37290.1* | 354 | 44 | 92 | 6.576662523 | 14.58704152 | 1.149259765 | 7.30E-06 | 5.77E-05 |
| *LOC_Os04g29550.1* | 552 | 45 | 94 | 4.313497776 | 9.55809691 | 1.147865183 | 5.92E-06 | 4.74E-05 |
| *LOC_Os11g44810.2* | 363 | 34 | 71 | 4.955967326 | 10.97828187 | 1.147413706 | 8.48E-05 | 0.000553657 |
| *LOC_Os01g65010.1* | 2475 | 57 | 119 | 1.218584907 | 2.698698774 | 1.147057177 | 3.45E-07 | 3.35E-06 |
| *LOC_Os02g41460.1* | 630 | 46 | 96 | 3.863433352 | 8.552898876 | 1.146529972 | 4.80E-06 | 3.91E-05 |
| *LOC_Os03g26690.1* | 2454 | 36 | 75 | 0.776218671 | 1.715415614 | 1.144023117 | 5.55E-05 | 0.00037569 |
| *LOC_Os01g61044.2* | 1371 | 48 | 100 | 1.852507287 | 4.093975119 | 1.144023117 | 3.16E-06 | 2.65E-05 |
| *LOC_Os01g46720.1* | 1953 | 4298 | 8951 | 116.4448566 | 257.2479766 | 1.143512813 | 0 | 0 |
| *LOC_Os01g10890.1* | 1386 | 426 | 887 | 16.26306925 | 35.92055541 | 1.143210102 | 5.32E-44 | 3.43E-42 |
| *LOC_Os04g54010.1* | 2157 | 442 | 920 | 10.84247094 | 23.93978997 | 1.142716919 | 1.53E-45 | 1.02E-43 |
| *LOC_Os01g59890.1* | 1815 | 37 | 77 | 1.078651712 | 2.381204801 | 1.142462603 | 4.49E-05 | 0.000309373 |
| *LOC_Os03g31180.1* | 2220 | 199 | 414 | 4.743034071 | 10.4671879 | 1.141991765 | 2.26E-21 | 6.55E-20 |
| *LOC_Os12g44110.2* | 1764 | 602 | 1252 | 18.05735154 | 39.83716292 | 1.141528598 | 3.05E-61 | 2.82E-59 |
| *LOC_Os10g33130.1* | 3147 | 101 | 210 | 1.698168471 | 3.745460364 | 1.141163463 | 1.46E-11 | 2.28E-10 |
| *LOC_Os12g22145.1* | 207 | 76 | 158 | 19.42671591 | 42.84196629 | 1.140982662 | 4.85E-09 | 5.84E-08 |
| *LOC_Os07g01904.2* | 294 | 64 | 133 | 11.51831062 | 25.39141854 | 1.140411863 | 8.09E-08 | 8.50E-07 |
| *LOC_Os12g15680.1* | 1797 | 39 | 81 | 1.148345763 | 2.529994607 | 1.139577212 | 2.95E-05 | 0.000210164 |
| *LOC_Os08g29809.1* | 2778 | 105 | 218 | 1.999922655 | 4.404604375 | 1.139068235 | 6.46E-12 | 1.04E-10 |
| *LOC_Os02g57280.8* | 522 | 41 | 85 | 4.155942174 | 9.139681809 | 1.136968359 | 1.93E-05 | 0.000142239 |
| *LOC_Os02g20980.2* | 864 | 169 | 350 | 10.34973201 | 22.73719862 | 1.135461103 | 3.74E-18 | 9.13E-17 |
| *LOC_Os10g10480.1* | 4110 | 140 | 289 | 1.802363386 | 3.946741429 | 1.130772093 | 3.68E-15 | 7.53E-14 |
| *LOC_Os02g21040.1* | 1545 | 50 | 103 | 1.712370207 | 3.741893258 | 1.127773765 | 2.93E-06 | 2.47E-05 |
| *LOC_Os12g01010.1* | 687 | 34 | 70 | 2.618655225 | 5.719050832 | 1.126949603 | 0.00011981 | 0.000757041 |
| *LOC_Os12g43660.1* | 2934 | 188 | 387 | 3.390422974 | 7.403439116 | 1.126730332 | 1.10E-19 | 2.92E-18 |
| *LOC_Os04g35540.1* | 1593 | 87 | 179 | 2.889745654 | 6.306957564 | 1.126001709 | 7.06E-10 | 9.26E-09 |
| *LOC_Os01g40680.1* | 1620 | 35 | 72 | 1.143165666 | 2.494595506 | 1.125771412 | 9.69E-05 | 0.000624554 |
| *LOC_Os04g41410.1* | 1455 | 73 | 150 | 2.65470342 | 5.786432874 | 1.124123559 | 1.76E-08 | 2.02E-07 |
| *LOC_Os05g39880.1* | 429 | 38 | 78 | 4.686865027 | 10.20516343 | 1.122604133 | 5.13E-05 | 0.000349377 |
| *LOC_Os08g19670.1* | 399 | 117 | 240 | 15.51561907 | 33.76144293 | 1.121655304 | 1.08E-12 | 1.85E-11 |
| *LOC_Os12g06464.1* | 744 | 98 | 201 | 6.96962293 | 15.16372066 | 1.121471275 | 7.38E-11 | 1.07E-09 |
| *LOC_Os04g37490.1* | 1074 | 216 | 443 | 10.6415677 | 23.15165801 | 1.121404814 | 3.78E-22 | 1.14E-20 |
| *LOC_Os02g04160.1* | 318 | 60 | 123 | 9.983441394 | 21.71004107 | 1.120753337 | 3.64E-07 | 3.53E-06 |
| *LOC_Os06g44160.1* | 429 | 141 | 289 | 17.39073602 | 37.81143887 | 1.120503758 | 5.82E-15 | 1.17E-13 |
| *LOC_Os02g04510.1* | 1797 | 410 | 840 | 12.07235289 | 26.23698111 | 1.119894846 | 1.88E-40 | 1.08E-38 |
| *LOC_Os06g37690.1* | 2421 | 64 | 131 | 1.398753953 | 3.037100476 | 1.118552429 | 1.59E-07 | 1.60E-06 |
| *LOC_Os09g28650.1* | 939 | 67 | 137 | 3.775420702 | 8.189127419 | 1.11707232 | 8.53E-08 | 8.92E-07 |
| *LOC_Os07g07320.1* | 708 | 318 | 650 | 23.76566684 | 51.5303097 | 1.11654238 | 1.55E-31 | 6.78E-30 |
| *LOC_Os12g08220.1* | 1332 | 713 | 1457 | 28.32314316 | 61.39570358 | 1.116156323 | 1.64E-68 | 1.71E-66 |
| *LOC_Os11g29810.1* | 726 | 48 | 98 | 3.498329877 | 7.57656073 | 1.114876771 | 6.24E-06 | 4.98E-05 |
| *LOC_Os03g18910.1* | 2016 | 410 | 836 | 10.7609217 | 23.27546699 | 1.11300846 | 6.93E-40 | 3.92E-38 |
| *LOC_Os08g39300.3* | 687 | 103 | 210 | 7.932984945 | 17.15715249 | 1.112874418 | 3.69E-11 | 5.51E-10 |
| *LOC_Os02g56050.1* | 528 | 52 | 106 | 5.211053879 | 11.26820129 | 1.112610164 | 2.70E-06 | 2.29E-05 |
| *LOC_Os01g66120.1* | 912 | 353 | 719 | 20.48028564 | 44.25034955 | 1.111453015 | 1.83E-34 | 8.82E-33 |
| *LOC_Os02g44880.1* | 1596 | 57 | 116 | 1.889722835 | 4.079507688 | 1.110220409 | 9.51E-07 | 8.64E-06 |
| *LOC_Os03g08330.1* | 564 | 61 | 124 | 5.722777664 | 12.34028628 | 1.1085884 | 4.14E-07 | 3.97E-06 |
| *LOC_Os04g02850.1* | 2424 | 431 | 876 | 9.408075568 | 20.28402534 | 1.108372428 | 1.92E-41 | 1.15E-39 |
| *LOC_Os01g20830.1* | 747 | 344 | 699 | 24.36654665 | 52.52175477 | 1.108013318 | 2.15E-33 | 9.99E-32 |
| *LOC_Os02g36450.1* | 1557 | 66 | 134 | 2.242908028 | 4.830575112 | 1.106824499 | 1.46E-07 | 1.48E-06 |
| *LOC_Os02g33420.1* | 2469 | 304 | 617 | 6.514913234 | 14.02641641 | 1.106328593 | 1.34E-29 | 5.40E-28 |
| *LOC_Os12g09130.1* | 816 | 36 | 73 | 2.334363502 | 5.021290586 | 1.105028985 | 0.00011014 | 0.00070183 |
| *LOC_Os03g42760.1* | 1623 | 42 | 85 | 1.369263127 | 2.939564944 | 1.102202941 | 3.09E-05 | 0.000219632 |
| *LOC_Os12g18080.1* | 4239 | 43 | 87 | 0.536736564 | 1.151962893 | 1.101808169 | 2.50E-05 | 0.000181148 |
| *LOC_Os09g23590.1* | 297 | 130 | 263 | 23.16023946 | 49.7029256 | 1.101680604 | 2.04E-13 | 3.70E-12 |
| *LOC_Os02g02640.1* | 4425 | 49 | 99 | 0.585920843 | 1.255754009 | 1.099776204 | 7.08E-06 | 5.60E-05 |
| *LOC_Os06g07760.1* | 465 | 393 | 794 | 44.71937652 | 95.84074991 | 1.099739123 | 2.87E-37 | 1.50E-35 |
| *LOC_Os01g42090.1* | 765 | 51 | 103 | 3.527482626 | 7.557156973 | 1.099204613 | 4.65E-06 | 3.80E-05 |
| *LOC_Os03g37411.1* | 1503 | 59 | 119 | 2.077060628 | 4.443965047 | 1.097304142 | 8.74E-07 | 7.98E-06 |
| *LOC_Os07g07150.1* | 954 | 61 | 123 | 3.383277361 | 7.236680358 | 1.096906595 | 5.76E-07 | 5.41E-06 |
| *LOC_Os01g02390.1* | 1980 | 129 | 260 | 3.447312566 | 7.370395812 | 1.096269985 | 3.47E-13 | 6.17E-12 |
| *LOC_Os02g52040.1* | 936 | 654 | 1314 | 36.97073137 | 78.79563688 | 1.091732162 | 7.59E-60 | 6.75E-58 |
| *LOC_Os08g10080.1* | 882 | 332 | 667 | 19.91707877 | 42.44630618 | 1.091632947 | 3.23E-31 | 1.39E-29 |
| *LOC_Os12g42220.1* | 804 | 113 | 227 | 7.436670461 | 15.8471972 | 1.091498953 | 1.27E-11 | 1.99E-10 |
| *LOC_Os04g57850.1* | 1677 | 330 | 662 | 10.41206857 | 22.1568277 | 1.08949462 | 6.66E-31 | 2.84E-29 |
| *LOC_Os10g01110.1* | 1383 | 169 | 338 | 6.46577618 | 13.71756965 | 1.085129428 | 1.90E-16 | 4.23E-15 |
| *LOC_Os12g01160.1* | 1104 | 44 | 88 | 2.108821135 | 4.474002809 | 1.085129428 | 2.83E-05 | 0.000202466 |
| *LOC_Os06g46120.1* | 525 | 46 | 92 | 4.636120023 | 9.835833708 | 1.085129428 | 1.85E-05 | 0.000136949 |
| *LOC_Os01g73910.2* | 984 | 916 | 1832 | 49.25570252 | 104.4992142 | 1.085129428 | 6.34E-82 | 8.35E-80 |
| *LOC_Os03g24930.1* | 1344 | 152 | 304 | 5.984122312 | 12.69570927 | 1.085129428 | 6.06E-15 | 1.22E-13 |
| *LOC_Os04g35130.1* | 1473 | 88 | 176 | 3.161084227 | 6.706448202 | 1.085129428 | 2.99E-09 | 3.69E-08 |
| *LOC_Os03g02860.1* | 582 | 71 | 142 | 6.454929547 | 13.6945578 | 1.085129428 | 1.00E-07 | 1.04E-06 |
| *LOC_Os07g47620.1* | 516 | 55 | 110 | 5.639870478 | 11.96535635 | 1.085129428 | 2.80E-06 | 2.37E-05 |
| *LOC_Os03g53180.1* | 459 | 67 | 134 | 7.72357307 | 16.38606852 | 1.085129428 | 2.30E-07 | 2.28E-06 |
| *LOC_Os05g25770.1* | 981 | 268 | 535 | 14.45512758 | 30.61028889 | 1.082435318 | 4.89E-25 | 1.66E-23 |
| *LOC_Os07g43322.1* | 1464 | 153 | 305 | 5.529762723 | 11.69341643 | 1.080407017 | 6.81E-15 | 1.37E-13 |
| *LOC_Os03g61310.1* | 2394 | 107 | 213 | 2.364916297 | 4.993880101 | 1.078372061 | 8.29E-11 | 1.20E-09 |
| *LOC_Os02g03020.1* | 444 | 81 | 161 | 9.652908537 | 20.35286536 | 1.076196303 | 1.75E-08 | 2.00E-07 |
| *LOC_Os02g47770.1* | 1197 | 77 | 153 | 3.403711306 | 7.174306623 | 1.07573073 | 4.01E-08 | 4.37E-07 |
| *LOC_Os03g16230.2* | 810 | 71 | 141 | 4.637986416 | 9.770499064 | 1.074933661 | 1.39E-07 | 1.41E-06 |
| *LOC_Os04g54080.1* | 2499 | 56 | 111 | 1.185708446 | 2.493098149 | 1.072190372 | 3.16E-06 | 2.65E-05 |
| *LOC_Os06g01360.1* | 1413 | 51 | 101 | 1.909783587 | 4.012008695 | 1.070915568 | 9.00E-06 | 7.02E-05 |
| *LOC_Os12g16540.1* | 1287 | 100 | 198 | 4.111285112 | 8.635138289 | 1.070629858 | 4.82E-10 | 6.44E-09 |
| *LOC_Os03g08900.1* | 1470 | 48 | 95 | 1.727746592 | 3.627345506 | 1.070022535 | 1.69E-05 | 0.000125588 |
| *LOC_Os04g45810.1* | 831 | 141 | 279 | 8.977888994 | 18.84455269 | 1.069699387 | 1.49E-13 | 2.74E-12 |
| *LOC_Os07g48330.1* | 1605 | 46 | 91 | 1.516487858 | 3.182357818 | 1.069362112 | 2.58E-05 | 0.000186143 |
| *LOC_Os02g11130.1* | 1506 | 320 | 633 | 11.24297251 | 23.59181706 | 1.069263022 | 8.84E-29 | 3.47E-27 |
| *LOC_Os01g60830.1* | 513 | 1012 | 2001 | 104.38048 | 218.9335792 | 1.068641305 | 3.74E-87 | 5.32E-85 |
| *LOC_Os09g17630.1* | 3000 | 125 | 247 | 2.204676641 | 4.621238174 | 1.067712375 | 3.94E-12 | 6.47E-11 |
| *LOC_Os03g05620.1* | 1584 | 833 | 1645 | 27.82569155 | 58.28990919 | 1.066828611 | 8.77E-72 | 9.76E-70 |
| *LOC_Os03g63580.1* | 1584 | 39 | 77 | 1.30276347 | 2.728463834 | 1.066513749 | 0.000113488 | 0.000720955 |
| *LOC_Os06g24730.1* | 1461 | 955 | 1882 | 34.58671363 | 72.30229068 | 1.063823417 | 1.72E-81 | 2.24E-79 |
| *LOC_Os05g50230.1* | 921 | 221 | 435 | 12.69663942 | 26.51015582 | 1.062098459 | 4.52E-20 | 1.22E-18 |
| *LOC_Os05g29735.1* | 447 | 60 | 118 | 7.102314012 | 14.81689277 | 1.060881881 | 1.89E-06 | 1.65E-05 |
| *LOC_Os12g43640.1* | 3024 | 692 | 1360 | 12.10822409 | 25.24293071 | 1.059892136 | 4.77E-59 | 4.16E-57 |
| *LOC_Os04g38920.1* | 912 | 253 | 497 | 14.67850501 | 30.58751562 | 1.059237895 | 1.29E-22 | 3.97E-21 |
| *LOC_Os07g05560.1* | 1779 | 54 | 106 | 1.606105074 | 3.344356538 | 1.05816238 | 6.64E-06 | 5.28E-05 |
| *LOC_Os10g38610.1* | 705 | 80 | 157 | 6.004225746 | 12.49951578 | 1.057822082 | 4.10E-08 | 4.47E-07 |
| *LOC_Os02g15360.1* | 714 | 52 | 102 | 3.853552449 | 8.018342697 | 1.057115051 | 1.01E-05 | 7.80E-05 |
| *LOC_Os01g03340.1* | 756 | 176 | 345 | 12.3181933 | 25.61415028 | 1.056150361 | 4.23E-16 | 9.23E-15 |
| *LOC_Os01g65030.1* | 1287 | 50 | 98 | 2.055642556 | 4.273957335 | 1.055983082 | 1.54E-05 | 0.000115072 |
| *LOC_Os10g41420.1* | 639 | 50 | 98 | 4.140237824 | 8.608111252 | 1.055983082 | 1.54E-05 | 0.000115096 |
| *LOC_Os03g38950.1* | 816 | 3031 | 5935 | 196.5404382 | 408.2378031 | 1.054583605 | 3.33E-249 | 1.81E-246 |
| *LOC_Os01g71474.1* | 1047 | 832 | 1629 | 42.04678431 | 87.32871229 | 1.054460598 | 8.55E-70 | 9.12E-68 |
| *LOC_Os03g47580.1* | 795 | 71 | 139 | 4.725495593 | 9.813644583 | 1.054323381 | 2.65E-07 | 2.60E-06 |
| *LOC_Os03g18560.1* | 513 | 210 | 411 | 21.65998104 | 44.96836635 | 1.053878494 | 7.91E-19 | 2.00E-17 |
| *LOC_Os12g16490.1* | 3570 | 46 | 90 | 0.681782356 | 1.415001652 | 1.053420568 | 3.57E-05 | 0.000250938 |
| *LOC_Os01g04130.1* | 447 | 188 | 367 | 22.25391724 | 46.08304785 | 1.050176829 | 6.91E-17 | 1.58E-15 |
| *LOC_Os09g02270.1* | 804 | 228 | 445 | 15.00496341 | 31.06609142 | 1.049900939 | 3.84E-20 | 1.04E-18 |
| *LOC_Os05g38530.1* | 1941 | 165 | 322 | 4.497949253 | 9.311357258 | 1.049724092 | 5.44E-15 | 1.10E-13 |
| *LOC_Os07g23260.1* | 1218 | 61 | 119 | 2.64995616 | 5.483809086 | 1.049209853 | 2.12E-06 | 1.83E-05 |
| *LOC_Os12g06910.2* | 2679 | 81 | 158 | 1.59981015 | 3.310297507 | 1.049060173 | 4.59E-08 | 4.98E-07 |
| *LOC_Os07g37230.1* | 1305 | 322 | 628 | 13.05574029 | 27.01044789 | 1.048833298 | 9.94E-28 | 3.77E-26 |
| *LOC_Os11g39020.1* | 2073 | 10809 | 21066 | 275.8940644 | 570.3815006 | 1.047812772 | 0 | 0 |
| *LOC_Os10g42960.1* | 2166 | 887 | 1727 | 21.66812389 | 44.75242145 | 1.046391501 | 5.56E-73 | 6.29E-71 |
| *LOC_Os03g04070.1* | 951 | 55 | 107 | 3.060118997 | 6.315182629 | 1.045236701 | 7.43E-06 | 5.87E-05 |
| *LOC_Os02g52990.1* | 387 | 47 | 91 | 6.426034241 | 13.19815064 | 1.038335216 | 3.99E-05 | 0.000277798 |
| *LOC_Os10g09990.1* | 1587 | 740 | 1432 | 24.67237375 | 50.64641915 | 1.037563744 | 5.20E-60 | 4.66E-58 |
| *LOC_Os03g18150.1* | 1176 | 592 | 1144 | 26.6360933 | 54.60109551 | 1.035547399 | 3.78E-48 | 2.69E-46 |
| *LOC_Os09g38410.1* | 660 | 160 | 309 | 12.82720955 | 26.27829584 | 1.034664361 | 3.84E-14 | 7.35E-13 |
| *LOC_Os03g20730.1* | 498 | 218 | 421 | 23.16238592 | 47.4499115 | 1.034621526 | 1.01E-18 | 2.54E-17 |
| *LOC_Os07g47400.1* | 933 | 84 | 162 | 4.763802903 | 9.745767007 | 1.032662008 | 4.64E-08 | 5.03E-07 |
| *LOC_Os02g56900.1* | 759 | 454 | 875 | 31.6497453 | 64.7066522 | 1.031720147 | 5.14E-37 | 2.67E-35 |
| *LOC_Os08g38460.1* | 504 | 55 | 106 | 5.774153108 | 11.8047823 | 1.031690169 | 1.02E-05 | 7.89E-05 |
| *LOC_Os02g46220.1* | 1029 | 810 | 1561 | 41.65103392 | 85.14716292 | 1.031606152 | 1.18E-64 | 1.17E-62 |
| *LOC_Os01g08120.1* | 1998 | 41 | 79 | 1.085786694 | 2.219291047 | 1.031358171 | 0.000142244 | 0.000885202 |
| *LOC_Os09g36110.1* | 2310 | 109 | 210 | 2.496724716 | 5.102581716 | 1.031190621 | 5.06E-10 | 6.75E-09 |
| *LOC_Os06g03560.1* | 2301 | 1072 | 2065 | 24.6509868 | 50.37164002 | 1.030966303 | 7.51E-85 | 1.03E-82 |
| *LOC_Os01g10040.1* | 1473 | 105 | 202 | 3.771748225 | 7.697173505 | 1.029095393 | 1.15E-09 | 1.48E-08 |
| *LOC_Os06g18000.1* | 1530 | 219 | 421 | 7.573712697 | 15.444481 | 1.028018791 | 1.53E-18 | 3.81E-17 |
| *LOC_Os01g66160.1* | 1554 | 51 | 98 | 1.736502065 | 3.539628758 | 1.02741393 | 2.37E-05 | 0.000172274 |
| *LOC_Os11g08210.1* | 990 | 356 | 684 | 19.0270275 | 38.77962104 | 1.027248512 | 4.21E-29 | 1.67E-27 |
| *LOC_Os04g44900.1* | 2022 | 291 | 559 | 7.614966203 | 15.5171983 | 1.026958558 | 4.55E-24 | 1.48E-22 |
| *LOC_Os05g37600.1* | 1461 | 162 | 311 | 5.867065559 | 11.94793433 | 1.026050195 | 4.75E-14 | 9.00E-13 |
| *LOC_Os11g06020.1* | 2037 | 4974 | 9548 | 129.2024932 | 263.0898147 | 1.025921487 | 0 | 0 |
| *LOC_Os11g29870.1* | 729 | 49 | 94 | 3.556515405 | 7.237406714 | 1.025008435 | 3.61E-05 | 0.00025294 |
| *LOC_Os09g31200.1* | 495 | 440 | 844 | 47.03310168 | 95.70175485 | 1.024868903 | 2.16E-35 | 1.07E-33 |
| *LOC_Os09g12240.1* | 3645 | 106 | 203 | 1.538737277 | 3.125943751 | 1.02254489 | 1.28E-09 | 1.64E-08 |
| *LOC_Os03g21030.1* | 1077 | 593 | 1135 | 29.1336657 | 59.15109817 | 1.021717715 | 8.92E-47 | 6.17E-45 |
| *LOC_Os08g39210.1* | 2091 | 56 | 107 | 1.417066191 | 2.872184926 | 1.019241492 | 1.14E-05 | 8.71E-05 |
| *LOC_Os05g12450.1* | 1473 | 274 | 523 | 9.842466797 | 19.92882051 | 1.017764481 | 2.60E-22 | 7.91E-21 |
| *LOC_Os08g14940.1* | 2904 | 962 | 1836 | 17.52809032 | 35.48613648 | 1.017586687 | 4.48E-74 | 5.13E-72 |
| *LOC_Os02g37000.1* | 849 | 76 | 145 | 4.736549109 | 9.586122305 | 1.017111004 | 3.32E-07 | 3.22E-06 |
| *LOC_Os01g64890.1* | 1257 | 292 | 557 | 12.29146691 | 24.87153395 | 1.016838386 | 1.26E-23 | 4.03E-22 |
| *LOC_Os02g40840.1* | 2313 | 491 | 936 | 11.23212691 | 22.71343768 | 1.015914933 | 1.58E-38 | 8.53E-37 |
| *LOC_Os01g65560.1* | 567 | 73 | 139 | 6.812334172 | 13.75987203 | 1.014245941 | 6.17E-07 | 5.76E-06 |
| *LOC_Os07g40900.1* | 1029 | 135 | 257 | 6.941838987 | 14.01846308 | 1.01393838 | 1.16E-11 | 1.83E-10 |
| *LOC_Os10g41550.1* | 1635 | 197 | 375 | 6.375358507 | 12.87348598 | 1.013824394 | 2.44E-16 | 5.39E-15 |
| *LOC_Os08g38880.3* | 1164 | 103 | 196 | 4.682096784 | 9.451173694 | 1.013338745 | 3.22E-09 | 3.97E-08 |
| *LOC_Os08g40919.1* | 483 | 51 | 97 | 5.587006644 | 11.27216292 | 1.012616928 | 3.25E-05 | 0.000230117 |
| *LOC_Os09g02710.1* | 684 | 2236 | 4248 | 172.97042 | 348.5868983 | 1.010993006 | 4.42E-167 | 1.35E-164 |
| *LOC_Os08g13570.1* | 1581 | 169 | 321 | 5.656020529 | 11.39608858 | 1.010679478 | 3.90E-14 | 7.45E-13 |
| *LOC_Os01g41730.1* | 738 | 148 | 281 | 10.6111266 | 21.37138223 | 1.010102382 | 1.52E-12 | 2.57E-11 |
| *LOC_Os05g05820.1* | 1107 | 59 | 112 | 2.820074186 | 5.678753997 | 1.0098413 | 8.31E-06 | 6.52E-05 |
| *LOC_Os10g14870.1* | 798 | 1581 | 2995 | 104.8298878 | 210.6573366 | 1.006848063 | 1.35E-117 | 2.72E-115 |
| *LOC_Os08g26710.1* | 1524 | 194 | 367 | 6.735547534 | 13.51648451 | 1.004852838 | 8.23E-16 | 1.76E-14 |
| *LOC_Os07g26660.1* | 852 | 64 | 121 | 3.974628311 | 7.971286695 | 1.003992665 | 4.00E-06 | 3.30E-05 |
| *LOC_Os04g49690.1* | 1542 | 54 | 102 | 1.8529578 | 3.712773467 | 1.002667267 | 2.37E-05 | 0.000172251 |
| *LOC_Os05g27780.1* | 522 | 815 | 1538 | 82.61202127 | 165.3744779 | 1.001312966 | 9.34E-61 | 8.50E-59 |
| *LOC_Os01g74450.1* | 759 | 2330 | 4396 | 162.4315122 | 325.0862206 | 1.000990859 | 4.81E-170 | 1.51E-167 |
