# Supplementary material for: Transcriptome analysis of phosphorus stress responsiveness in the seedlings of Dongxiang wild rice (Oryza rufipogon Griff.)
Source: Biol Res. 2018 Mar 15;51:7. doi: 10.1186/s40659-018-0155-x (PMC5853122; doi:10.1186/s40659-018-0155-x)
Supplement: Supplementary file 5 — Additional file 5: Table S4. List of up-regulated genes in RLP vs. RCK. [file 40659_2018_155_MOESM5_ESM.docx]

| **Table S4** List of up-regulated genes in RLP vs. RCK. | | | | | | | | |
| --- | --- | --- | --- | --- | --- | --- | --- | --- |
| Gene ID | Gene  Length | RLP-  Expression | RCK-  Expression | RLP-RPKM | RCK-RPKM | Log_2_ Ratio  (RCK/RLP) | *P*-value | FDR |
| *LOC_Os01g33615.1* | 324 | 246 | 0 | 41.03588605 | 0.001 | -15.32459848 | 9.16E-70 | 1.89E-67 |
| *LOC_Os08g08570.1* | 579 | 264 | 0 | 24.64331233 | 0.001 | -14.58890856 | 8.11E-75 | 1.79E-72 |
| *LOC_Os07g41340.1* | 288 | 75 | 0 | 14.07480848 | 0.001 | -13.78082767 | 9.25E-22 | 5.54E-20 |
| *LOC_Os03g12510.1* | 510 | 124 | 0 | 13.14090354 | 0.001 | -13.68177685 | 1.62E-35 | 1.71E-33 |
| *LOC_Os12g10330.1* | 1923 | 395 | 0 | 11.10175221 | 0.001 | -13.43849978 | 1.36E-111 | 5.46E-109 |
| *LOC_Os12g10340.1* | 1956 | 341 | 0 | 9.422350312 | 0.001 | -13.20187126 | 1.96E-96 | 6.45E-94 |
| *LOC_Os02g51020.1* | 546 | 91 | 0 | 9.007877425 | 0.001 | -13.13697148 | 2.98E-26 | 2.17E-24 |
| *LOC_Os04g02880.1* | 249 | 41 | 0 | 8.899348781 | 0.001 | -13.11948405 | 3.24E-12 | 1.00E-10 |
| *LOC_Os07g07715.1* | 420 | 55 | 0 | 7.077617977 | 0.001 | -12.78904818 | 3.81E-16 | 1.61E-14 |
| *LOC_Os12g38100.1* | 549 | 71 | 0 | 6.989719095 | 0.001 | -12.77101876 | 1.23E-20 | 6.88E-19 |
| *LOC_Os01g71930.1* | 1899 | 211 | 0 | 6.005251617 | 0.001 | -12.55200898 | 6.13E-60 | 1.09E-57 |
| *LOC_Os07g08669.1* | 207 | 22 | 0 | 5.74415372 | 0.001 | -12.48787864 | 6.99E-07 | 1.14E-05 |
| *LOC_Os09g17930.1* | 1584 | 158 | 0 | 5.391078156 | 0.001 | -12.39635811 | 4.63E-45 | 6.08E-43 |
| *LOC_Os08g08650.1* | 597 | 58 | 0 | 5.250823022 | 0.001 | -12.35832786 | 5.47E-17 | 2.46E-15 |
| *LOC_Os10g09620.1* | 2121 | 205 | 0 | 5.223804447 | 0.001 | -12.35088518 | 2.96E-58 | 5.11E-56 |
| *LOC_Os12g38270.2* | 246 | 22 | 0 | 4.833495204 | 0.001 | -12.23885109 | 6.99E-07 | 1.13E-05 |
| *LOC_Os08g29809.1* | 2778 | 246 | 0 | 4.786042865 | 0.001 | -12.2246176 | 9.16E-70 | 1.90E-67 |
| *LOC_Os01g33869.1* | 2340 | 185 | 0 | 4.272967496 | 0.001 | -12.06102263 | 1.22E-52 | 1.85E-50 |
| *LOC_Os07g41400.1* | 342 | 27 | 0 | 4.266889307 | 0.001 | -12.05896897 | 2.76E-08 | 5.59E-07 |
| *LOC_Os01g40820.1* | 1881 | 118 | 0 | 3.390524836 | 0.001 | -11.7272929 | 7.85E-34 | 7.89E-32 |
| *LOC_Os03g58730.1* | 864 | 52 | 0 | 3.252844626 | 0.001 | -11.6674862 | 2.65E-15 | 1.05E-13 |
| *LOC_Os10g37160.1* | 1551 | 92 | 0 | 3.205898348 | 0.001 | -11.64651297 | 1.56E-26 | 1.16E-24 |
| *LOC_Os01g08890.1* | 525 | 31 | 0 | 3.191362288 | 0.001 | -11.63995668 | 2.08E-09 | 4.89E-08 |
| *LOC_Os10g27310.1* | 273 | 16 | 0 | 3.167605248 | 0.001 | -11.62917684 | 3.38E-05 | 0.000391631 |
| *LOC_Os08g04210.1* | 828 | 47 | 0 | 3.067900282 | 0.001 | -11.58303588 | 6.70E-14 | 2.39E-12 |
| *LOC_Os01g06510.1* | 1767 | 97 | 0 | 2.966940951 | 0.001 | -11.5347605 | 6.17E-28 | 4.81E-26 |
| *LOC_Os01g38359.2* | 1254 | 68 | 0 | 2.930792655 | 0.001 | -11.51707519 | 8.53E-20 | 4.53E-18 |
| *LOC_Os04g28470.1* | 1881 | 100 | 0 | 2.873326132 | 0.001 | -11.48850604 | 8.87E-29 | 7.21E-27 |
| *LOC_Os08g06415.1* | 591 | 31 | 0 | 2.834966499 | 0.001 | -11.46911597 | 2.08E-09 | 4.89E-08 |
| *LOC_Os01g71810.1* | 957 | 49 | 0 | 2.767310306 | 0.001 | -11.43426871 | 1.84E-14 | 6.86E-13 |
| *LOC_Os01g27590.1* | 1131 | 57 | 0 | 2.723867444 | 0.001 | -11.41144078 | 1.04E-16 | 4.59E-15 |
| *LOC_Os04g29580.1* | 2325 | 117 | 0 | 2.719797829 | 0.001 | -11.4092837 | 1.50E-33 | 1.48E-31 |
| *LOC_Os01g24550.1* | 1086 | 54 | 0 | 2.687433044 | 0.001 | -11.3920131 | 7.26E-16 | 3.01E-14 |
| *LOC_Os04g51300.1* | 1062 | 51 | 0 | 2.595490105 | 0.001 | -11.34179127 | 5.05E-15 | 1.98E-13 |
| *LOC_Os05g45170.1* | 711 | 31 | 0 | 2.356491141 | 0.001 | -11.20242454 | 2.08E-09 | 4.88E-08 |
| *LOC_Os06g39780.1* | 1503 | 64 | 0 | 2.301413793 | 0.001 | -11.16830469 | 1.13E-18 | 5.61E-17 |
| *LOC_Os01g71800.1* | 1092 | 45 | 0 | 2.22722244 | 0.001 | -11.12102994 | 2.44E-13 | 8.35E-12 |
| *LOC_Os01g73540.1* | 594 | 23 | 0 | 2.0927392 | 0.001 | -11.03117682 | 3.66E-07 | 6.26E-06 |
| *LOC_Os07g29960.1* | 1215 | 46 | 0 | 2.046233884 | 0.001 | -10.99875534 | 1.28E-13 | 4.48E-12 |
| *LOC_Os09g26210.1* | 513 | 17 | 0 | 1.791039956 | 0.001 | -10.80658181 | 1.77E-05 | 0.000218945 |
| *LOC_Os01g73656.1* | 1083 | 34 | 0 | 1.696774695 | 0.001 | -10.7285793 | 2.99E-10 | 7.71E-09 |
| *LOC_Os01g03280.1* | 723 | 22 | 0 | 1.644591729 | 0.001 | -10.68351376 | 6.99E-07 | 1.14E-05 |
| *LOC_Os10g31420.1* | 693 | 21 | 0 | 1.637795895 | 0.001 | -10.67753986 | 1.33E-06 | 2.06E-05 |
| *LOC_Os12g26850.1* | 3591 | 107 | 0 | 1.610430885 | 0.001 | -10.65323103 | 9.61E-31 | 8.41E-29 |
| *LOC_Os07g38960.1* | 792 | 23 | 0 | 1.5695544 | 0.001 | -10.61613932 | 3.66E-07 | 6.26E-06 |
| *LOC_Os08g04480.1* | 564 | 16 | 0 | 1.533255732 | 0.001 | -10.58238263 | 3.38E-05 | 0.000391499 |
| *LOC_Os02g26800.1* | 2511 | 69 | 0 | 1.485169755 | 0.001 | -10.53641212 | 4.47E-20 | 2.41E-18 |
| *LOC_Os10g15300.1* | 669 | 18 | 0 | 1.45418649 | 0.001 | -10.50599658 | 9.27E-06 | 0.000121833 |
| *LOC_Os05g45150.1* | 1323 | 34 | 0 | 1.388969762 | 0.001 | -10.43979948 | 2.99E-10 | 7.71E-09 |
| *LOC_Os12g10410.1* | 2820 | 69 | 0 | 1.322433069 | 0.001 | -10.36897899 | 4.47E-20 | 2.41E-18 |
| *LOC_Os03g31750.3* | 2679 | 64 | 0 | 1.291162722 | 0.001 | -10.33445512 | 1.13E-18 | 5.60E-17 |
| *LOC_Os01g72410.1* | 1803 | 41 | 0 | 1.229028201 | 0.001 | -10.2633023 | 3.24E-12 | 1.00E-10 |
| *LOC_Os01g08880.1* | 669 | 15 | 0 | 1.211822075 | 0.001 | -10.24296218 | 6.45E-05 | 0.000702403 |
| *LOC_Os08g04240.1* | 825 | 18 | 0 | 1.179213045 | 0.001 | -10.20360867 | 9.27E-06 | 0.00012174 |
| *LOC_Os04g28480.2* | 1947 | 42 | 0 | 1.165888604 | 0.001 | -10.18721424 | 1.70E-12 | 5.39E-11 |
| *LOC_Os04g30110.1* | 804 | 17 | 0 | 1.14279042 | 0.001 | -10.15834513 | 1.77E-05 | 0.000218867 |
| *LOC_Os01g24050.1* | 2214 | 44 | 0 | 1.074110045 | 0.001 | -10.06892609 | 4.66E-13 | 1.55E-11 |
| *LOC_Os12g44210.1* | 1224 | 24 | 0 | 1.059750285 | 0.001 | -10.04950864 | 1.92E-07 | 3.44E-06 |
| *LOC_Os03g53410.1* | 1224 | 24 | 0 | 1.059750285 | 0.001 | -10.04950864 | 1.92E-07 | 3.43E-06 |
| *LOC_Os11g08536.1* | 780 | 15 | 0 | 1.039370472 | 0.001 | -10.02149426 | 6.45E-05 | 0.000701074 |
| *LOC_Os03g41170.1* | 816 | 15 | 0 | 0.993515892 | 0.001 | -9.956399235 | 6.45E-05 | 0.000700412 |
| *LOC_Os04g30060.1* | 1038 | 19 | 0 | 0.989304457 | 0.001 | -9.950270766 | 4.86E-06 | 6.81E-05 |
| *LOC_Os03g53550.1* | 846 | 15 | 0 | 0.958284832 | 0.001 | -9.904310724 | 6.45E-05 | 0.000700633 |
| *LOC_Os08g17500.1* | 1029 | 18 | 0 | 0.945433199 | 0.001 | -9.884831716 | 9.27E-06 | 0.000121786 |
| *LOC_Os01g02190.1* | 861 | 15 | 0 | 0.941589975 | 0.001 | -9.87895515 | 6.45E-05 | 0.000702181 |
| *LOC_Os04g38540.1* | 1092 | 18 | 0 | 0.890888976 | 0.001 | -9.799101842 | 9.27E-06 | 0.000121694 |
| *LOC_Os03g45519.1* | 3768 | 62 | 0 | 0.889312739 | 0.001 | -9.796547042 | 4.12E-18 | 1.98E-16 |
| *LOC_Os09g04430.1* | 984 | 16 | 0 | 0.87881731 | 0.001 | -9.779419476 | 3.38E-05 | 0.000391894 |
| *LOC_Os09g19350.1* | 1920 | 28 | 0 | 0.788189275 | 0.001 | -9.622398308 | 1.45E-08 | 3.05E-07 |
| *LOC_Os12g32900.1* | 1284 | 17 | 0 | 0.715579048 | 0.001 | -9.482967336 | 1.77E-05 | 0.000218788 |
| *LOC_Os12g09540.1* | 1557 | 20 | 0 | 0.694248742 | 0.001 | -9.439308847 | 2.55E-06 | 3.77E-05 |
| *LOC_Os05g12550.1* | 1185 | 15 | 0 | 0.684142589 | 0.001 | -9.418153233 | 6.45E-05 | 0.000701295 |
| *LOC_Os08g39240.1* | 2151 | 26 | 0 | 0.653290971 | 0.001 | -9.35158189 | 5.27E-08 | 1.03E-06 |
| *LOC_Os02g57290.4* | 1932 | 22 | 0 | 0.615445041 | 0.001 | -9.265486221 | 6.99E-07 | 1.14E-05 |
| *LOC_Os01g61044.2* | 1371 | 15 | 0 | 0.591326746 | 0.001 | -9.207811721 | 6.45E-05 | 0.000701517 |
| *LOC_Os07g11700.1* | 1395 | 15 | 0 | 0.581153382 | 0.001 | -9.18277517 | 6.45E-05 | 0.000700854 |
| *LOC_Os12g23980.1* | 2202 | 23 | 0 | 0.564526378 | 0.001 | -9.140897184 | 3.66E-07 | 6.25E-06 |
| *LOC_Os04g51750.1* | 3894 | 40 | 0 | 0.555185049 | 0.001 | -9.116824908 | 6.18E-12 | 1.86E-10 |
| *LOC_Os05g08890.1* | 2433 | 23 | 0 | 0.510927696 | 0.001 | -8.996975333 | 3.66E-07 | 6.25E-06 |
| *LOC_Os03g31630.1* | 2367 | 22 | 0 | 0.502340439 | 0.001 | -8.972521609 | 6.99E-07 | 1.13E-05 |
| *LOC_Os06g26234.3* | 2331 | 20 | 0 | 0.463725994 | 0.001 | -8.857128787 | 2.55E-06 | 3.77E-05 |
| *LOC_Os03g46884.1* | 1884 | 16 | 0 | 0.459000124 | 0.001 | -8.842350732 | 3.38E-05 | 0.000391368 |
| *LOC_Os01g23440.1* | 2070 | 15 | 0 | 0.391646845 | 0.001 | -8.613409525 | 6.45E-05 | 0.000701738 |
| *LOC_Os10g03100.1* | 2502 | 16 | 0 | 0.345625992 | 0.001 | -8.433067907 | 3.38E-05 | 0.000391763 |
| *LOC_Os01g06900.1* | 3093 | 17 | 0 | 0.297059003 | 0.001 | -8.214605705 | 1.77E-05 | 0.000219024 |
| *LOC_Os09g14690.1* | 4218 | 16 | 0 | 0.205015702 | 0.001 | -7.679590602 | 3.38E-05 | 0.000392026 |
| *LOC_Os07g36010.1* | 5439 | 15 | 0 | 0.149054784 | 0.001 | -7.219698867 | 6.45E-05 | 0.000701959 |
| *LOC_Os04g13630.1* | 7578 | 18 | 0 | 0.128378301 | 0.001 | -7.004257558 | 9.27E-06 | 0.000121879 |
| *LOC_Os06g01590.1* | 1083 | 146 | 2 | 7.286150161 | 0.109845197 | -6.051613032 | 2.75E-38 | 3.08E-36 |
| *LOC_Os03g05334.1* | 192 | 395 | 7 | 111.190987 | 2.168584479 | -5.680142394 | 2.50E-99 | 8.46E-97 |
| *LOC_Os07g46130.1* | 2478 | 93 | 2 | 2.028408234 | 0.048007405 | -5.400947284 | 8.65E-24 | 5.61E-22 |
| *LOC_Os01g19460.1* | 3396 | 90 | 2 | 1.432348 | 0.035030138 | -5.353641569 | 5.65E-23 | 3.56E-21 |
| *LOC_Os07g47640.1* | 354 | 86 | 2 | 13.13012642 | 0.336051832 | -5.288053227 | 6.87E-22 | 4.14E-20 |
| *LOC_Os12g23200.1* | 627 | 161 | 4 | 13.87816522 | 0.379465227 | -5.192705351 | 1.07E-39 | 1.25E-37 |
| *LOC_Os11g07020.1* | 1167 | 80 | 2 | 3.705039558 | 0.101938602 | -5.183716568 | 2.89E-20 | 1.58E-18 |
| *LOC_Os01g52240.1* | 798 | 1132 | 29 | 76.66855071 | 2.161596559 | -5.14846572 | 4.96E-270 | 5.19E-267 |
| *LOC_Os12g42010.1* | 1155 | 73 | 2 | 3.415974296 | 0.102997704 | -5.051613032 | 2.24E-18 | 1.09E-16 |
| *LOC_Os07g11110.1* | 1179 | 36 | 1 | 1.650298154 | 0.050450529 | -5.031713474 | 1.53E-09 | 3.65E-08 |
| *LOC_Os02g49680.1* | 1164 | 142 | 4 | 6.593394816 | 0.204402661 | -5.011535592 | 1.42E-34 | 1.45E-32 |
| *LOC_Os11g14970.1* | 6084 | 69 | 2 | 0.612962073 | 0.019553312 | -4.970312929 | 2.67E-17 | 1.23E-15 |
| *LOC_Os02g48850.1* | 864 | 63 | 2 | 3.940946373 | 0.137687903 | -4.839068396 | 1.09E-15 | 4.45E-14 |
| *LOC_Os01g66000.1* | 636 | 31 | 1 | 2.634379247 | 0.093523859 | -4.815984783 | 3.37E-08 | 6.76E-07 |
| *LOC_Os08g44780.1* | 333 | 31 | 1 | 5.03142703 | 0.178622145 | -4.815984783 | 3.37E-08 | 6.76E-07 |
| *LOC_Os01g21590.1* | 1644 | 60 | 2 | 1.972527903 | 0.072361526 | -4.768679068 | 6.89E-15 | 2.66E-13 |
| *LOC_Os12g08770.1* | 450 | 321 | 11 | 38.55371538 | 1.45398426 | -4.728786341 | 2.83E-74 | 6.22E-72 |
| *LOC_Os08g02630.1* | 618 | 113 | 4 | 9.882428631 | 0.384991419 | -4.681967435 | 8.14E-27 | 6.13E-25 |
| *LOC_Os01g02460.1* | 915 | 28 | 1 | 1.653905363 | 0.065006748 | -4.669143395 | 2.14E-07 | 3.80E-06 |
| *LOC_Os10g39150.1* | 519 | 27 | 1 | 2.811707404 | 0.114607272 | -4.616675975 | 3.95E-07 | 6.71E-06 |
| *LOC_Os01g56680.1* | 375 | 81 | 3 | 11.67420914 | 0.475849394 | -4.616675975 | 2.12E-19 | 1.10E-17 |
| *LOC_Os07g04840.1* | 765 | 731 | 28 | 51.6451639 | 2.177088731 | -4.568161147 | 5.80E-164 | 3.51E-161 |
| *LOC_Os05g48630.2* | 429 | 156 | 6 | 19.65355075 | 0.831904535 | -4.562228191 | 4.86E-36 | 5.23E-34 |
| *LOC_Os01g22430.1* | 1302 | 128 | 5 | 5.313402352 | 0.228422328 | -4.539860378 | 1.04E-29 | 8.69E-28 |
| *LOC_Os01g38229.1* | 1962 | 307 | 12 | 8.45693691 | 0.363799231 | -4.538920817 | 1.90E-69 | 3.88E-67 |
| *LOC_Os05g48630.1* | 450 | 73 | 3 | 8.76766736 | 0.396541162 | -4.466650531 | 2.78E-17 | 1.27E-15 |
| *LOC_Os06g38780.1* | 2793 | 24 | 1 | 0.464423326 | 0.021296518 | -4.446750973 | 2.47E-06 | 3.68E-05 |
| *LOC_Os06g21950.1* | 1659 | 402 | 17 | 13.09644385 | 0.609511731 | -4.425377323 | 4.09E-89 | 1.21E-86 |
| *LOC_Os06g21590.1* | 726 | 429 | 20 | 31.93701996 | 1.638599842 | -4.284694215 | 4.54E-93 | 1.37E-90 |
| *LOC_Os07g47780.1* | 588 | 230 | 11 | 21.14093681 | 1.112743056 | -4.247846905 | 2.83E-50 | 4.08E-48 |
| *LOC_Os03g16050.1* | 1221 | 242 | 12 | 10.71207045 | 0.584581565 | -4.195689209 | 2.11E-52 | 3.18E-50 |
| *LOC_Os10g25430.1* | 1134 | 20 | 1 | 0.953214542 | 0.052452535 | -4.183716568 | 2.80E-05 | 0.000331558 |
| *LOC_Os09g07580.1* | 2310 | 20 | 1 | 0.467941684 | 0.025749426 | -4.183716568 | 2.80E-05 | 0.000331671 |
| *LOC_Os09g13440.1* | 693 | 79 | 4 | 6.161232178 | 0.343325681 | -4.165569221 | 7.30E-18 | 3.44E-16 |
| *LOC_Os08g01380.1* | 420 | 136 | 7 | 17.501019 | 0.991352904 | -4.141896392 | 8.95E-30 | 7.51E-28 |
| *LOC_Os01g41710.1* | 786 | 2480 | 128 | 170.5308093 | 9.686501662 | -4.137912878 | 0 | 0 |
| *LOC_Os04g51720.1* | 3114 | 38 | 2 | 0.659536305 | 0.038202424 | -4.109715986 | 4.42E-09 | 1.00E-07 |
| *LOC_Os11g30240.1* | 1758 | 37 | 2 | 1.137513531 | 0.06766914 | -4.071241838 | 8.04E-09 | 1.76E-07 |
| *LOC_Os02g57010.1* | 951 | 37 | 2 | 2.102785266 | 0.125091849 | -4.071241838 | 8.04E-09 | 1.76E-07 |
| *LOC_Os06g24070.1* | 1368 | 37 | 2 | 1.46180467 | 0.086960781 | -4.071241838 | 8.04E-09 | 1.76E-07 |
| *LOC_Os01g71340.1* | 993 | 976 | 53 | 53.12198409 | 3.174725313 | -4.064605356 | 2.72E-202 | 1.87E-199 |
| *LOC_Os06g49160.1* | 720 | 55 | 3 | 4.128610486 | 0.247838226 | -4.058185686 | 1.42E-12 | 4.53E-11 |
| *LOC_Os12g32850.1* | 1569 | 55 | 3 | 1.89458225 | 0.113730735 | -4.058185686 | 1.42E-12 | 4.52E-11 |
| *LOC_Os07g13969.1* | 366 | 18 | 1 | 2.658062191 | 0.16251687 | -4.031713474 | 9.32E-05 | 0.000975364 |
| *LOC_Os07g02590.1* | 1773 | 18 | 1 | 0.548703193 | 0.033548322 | -4.031713474 | 9.32E-05 | 0.000975956 |
| *LOC_Os09g25100.1* | 1215 | 18 | 1 | 0.800700216 | 0.048955699 | -4.031713474 | 9.32E-05 | 0.000976253 |
| *LOC_Os01g02420.1* | 1743 | 18 | 1 | 0.55814731 | 0.034125745 | -4.031713474 | 9.32E-05 | 0.00097566 |
| *LOC_Os07g42104.1* | 834 | 18 | 1 | 1.166487724 | 0.071320353 | -4.031713474 | 9.32E-05 | 0.000975068 |
| *LOC_Os04g33830.1* | 429 | 396 | 23 | 49.88978266 | 3.188967385 | -3.967583137 | 1.33E-81 | 3.56E-79 |
| *LOC_Os07g37550.1* | 801 | 1151 | 67 | 77.66342259 | 4.975329183 | -3.9643714 | 5.45E-234 | 4.94E-231 |
| *LOC_Os08g33820.1* | 735 | 1070 | 64 | 78.68105179 | 5.179313134 | -3.925183554 | 4.73E-216 | 3.47E-213 |
| *LOC_Os07g12810.1* | 1446 | 50 | 3 | 1.868854238 | 0.123404926 | -3.920682162 | 2.75E-11 | 7.84E-10 |
| *LOC_Os03g22370.1* | 387 | 66 | 4 | 9.217362946 | 0.614792499 | -3.906182592 | 1.66E-14 | 6.22E-13 |
| *LOC_Os07g41350.1* | 288 | 33 | 2 | 6.19291573 | 0.41306371 | -3.906182592 | 8.68E-08 | 1.64E-06 |
| *LOC_Os03g39610.1* | 792 | 363 | 22 | 24.77166292 | 1.652254841 | -3.906182592 | 4.41E-74 | 9.64E-72 |
| *LOC_Os04g38410.1* | 759 | 491 | 30 | 34.96338194 | 2.351034556 | -3.894477091 | 1.91E-99 | 6.54E-97 |
| *LOC_Os01g65690.1* | 843 | 65 | 4 | 4.167345428 | 0.282235702 | -3.884156286 | 2.99E-14 | 1.09E-12 |
| *LOC_Os07g05480.1* | 402 | 209 | 13 | 28.09919973 | 1.923520561 | -3.868707887 | 5.86E-43 | 7.40E-41 |
| *LOC_Os12g38270.1* | 324 | 6019 | 375 | 1004.044708 | 68.8439517 | -3.866349789 | 0 | 0 |
| *LOC_Os04g10760.1* | 972 | 32 | 2 | 1.779333812 | 0.122389247 | -3.861788473 | 1.57E-07 | 2.85E-06 |
| *LOC_Os11g09820.1* | 2469 | 126 | 8 | 2.75818361 | 0.192729605 | -3.839068396 | 2.79E-26 | 2.04E-24 |
| *LOC_Os11g40970.1* | 3021 | 63 | 4 | 1.127102836 | 0.078756934 | -3.839068396 | 9.68E-14 | 3.41E-12 |
| *LOC_Os11g20090.1* | 1107 | 141 | 9 | 6.884068926 | 0.483586783 | -3.831414824 | 3.26E-29 | 2.67E-27 |
| *LOC_Os07g37240.1* | 873 | 445 | 29 | 27.54986566 | 1.975892387 | -3.801469003 | 1.20E-88 | 3.52E-86 |
| *LOC_Os09g08930.1* | 2934 | 30 | 2 | 0.552630517 | 0.040546131 | -3.768679068 | 5.09E-07 | 8.47E-06 |
| *LOC_Os12g31460.1* | 708 | 44 | 3 | 3.358869548 | 0.252038874 | -3.736257591 | 9.35E-10 | 2.31E-08 |
| *LOC_Os12g38040.1* | 1926 | 322 | 22 | 9.035939348 | 0.679431897 | -3.733273732 | 1.13E-63 | 2.16E-61 |
| *LOC_Os10g35370.1* | 1209 | 102 | 7 | 4.559818845 | 0.344390587 | -3.726858893 | 4.63E-21 | 2.66E-19 |
| *LOC_Os04g06700.1* | 2310 | 29 | 2 | 0.678515442 | 0.051498852 | -3.719769468 | 9.14E-07 | 1.46E-05 |
| *LOC_Os12g19470.2* | 528 | 158 | 11 | 16.17323447 | 1.239191131 | -3.706137602 | 8.80E-32 | 8.14E-30 |
| *LOC_Os01g39960.1* | 1623 | 86 | 6 | 2.863872305 | 0.219893435 | -3.703090727 | 7.24E-18 | 3.41E-16 |
| *LOC_Os06g18820.1* | 873 | 325 | 23 | 20.12068841 | 1.567087065 | -3.682522425 | 1.43E-63 | 2.72E-61 |
| *LOC_Os05g25650.1* | 297 | 28 | 2 | 5.095365008 | 0.400546628 | -3.669143395 | 1.64E-06 | 2.50E-05 |
| *LOC_Os07g25430.1* | 450 | 261 | 19 | 31.34741344 | 2.511427358 | -3.641766956 | 6.80E-51 | 1.01E-48 |
| *LOC_Os10g31320.1* | 669 | 27 | 2 | 2.181279735 | 0.177821149 | -3.616675975 | 2.93E-06 | 4.28E-05 |
| *LOC_Os10g36170.1* | 276 | 81 | 6 | 15.8616972 | 1.293069006 | -3.616675975 | 1.31E-16 | 5.74E-15 |
| *LOC_Os08g45000.1* | 1605 | 241 | 18 | 8.115508259 | 0.66707859 | -3.604752807 | 1.00E-46 | 1.36E-44 |
| *LOC_Os05g33280.1* | 1086 | 40 | 3 | 1.990691144 | 0.164312636 | -3.598754067 | 9.57E-09 | 2.07E-07 |
| *LOC_Os10g10180.1* | 1095 | 26 | 2 | 1.283314044 | 0.108641414 | -3.562228191 | 5.24E-06 | 7.30E-05 |
| *LOC_Os06g38730.1* | 3009 | 26 | 2 | 0.4670086 | 0.03953551 | -3.562228191 | 5.24E-06 | 7.30E-05 |
| *LOC_Os12g19381.1* | 528 | 1539 | 120 | 157.5354927 | 13.5184487 | -3.542675393 | 1.04E-285 | 1.12E-282 |
| *LOC_Os12g33300.1* | 1122 | 205 | 16 | 9.87494584 | 0.848216389 | -3.541268572 | 2.15E-39 | 2.48E-37 |
| *LOC_Os04g03450.1* | 4833 | 25 | 2 | 0.279574098 | 0.024614597 | -3.505644662 | 9.34E-06 | 0.000122372 |
| *LOC_Os06g09610.1* | 798 | 25 | 2 | 1.693210042 | 0.149075625 | -3.505644662 | 9.34E-06 | 0.000122418 |
| *LOC_Os08g38920.1* | 705 | 85 | 7 | 6.516336861 | 0.59059322 | -3.463824487 | 8.29E-17 | 3.67E-15 |
| *LOC_Os01g19740.1* | 375 | 362 | 30 | 52.17362604 | 4.758493941 | -3.454743764 | 4.83E-67 | 9.63E-65 |
| *LOC_Os12g38640.1* | 573 | 84 | 7 | 7.923159201 | 0.726646108 | -3.446750973 | 1.46E-16 | 6.40E-15 |
| *LOC_Os02g35970.1* | 2289 | 24 | 2 | 0.566681673 | 0.051971319 | -3.446750973 | 1.66E-05 | 0.000206872 |
| *LOC_Os07g23570.1* | 1560 | 24 | 2 | 0.831496378 | 0.076257916 | -3.446750973 | 1.66E-05 | 0.000206798 |
| *LOC_Os03g10770.1* | 975 | 24 | 2 | 1.330394204 | 0.122012665 | -3.446750973 | 1.66E-05 | 0.000206723 |
| *LOC_Os12g23180.1* | 1131 | 104 | 9 | 4.969863407 | 0.473324994 | -3.392303189 | 5.99E-20 | 3.21E-18 |
| *LOC_Os01g50170.1* | 1362 | 23 | 2 | 0.912692426 | 0.087343868 | -3.385350429 | 2.94E-05 | 0.00034679 |
| *LOC_Os12g12120.1* | 3057 | 23 | 2 | 0.406636272 | 0.038914736 | -3.385350429 | 2.94E-05 | 0.000346553 |
| *LOC_Os05g51750.1* | 1608 | 23 | 2 | 0.773064107 | 0.07398156 | -3.385350429 | 2.94E-05 | 0.000346671 |
| *LOC_Os03g39830.1* | 357 | 23 | 2 | 3.482036652 | 0.333227867 | -3.385350429 | 2.94E-05 | 0.000346317 |
| *LOC_Os11g04300.1* | 3075 | 23 | 2 | 0.404255962 | 0.038686943 | -3.385350429 | 2.94E-05 | 0.000346435 |
| *LOC_Os01g06990.1* | 561 | 34 | 3 | 3.275591791 | 0.318081146 | -3.364288813 | 2.99E-07 | 5.19E-06 |
| *LOC_Os08g34300.1* | 3249 | 67 | 6 | 1.114548084 | 0.109845197 | -3.342915162 | 3.88E-13 | 1.30E-11 |
| *LOC_Os01g71070.1* | 1269 | 44 | 4 | 1.873979228 | 0.187489911 | -3.321220091 | 5.59E-09 | 1.25E-07 |
| *LOC_Os04g56080.1* | 2547 | 22 | 2 | 0.466839348 | 0.046706851 | -3.321220091 | 5.21E-05 | 0.000580321 |
| *LOC_Os06g15330.1* | 1347 | 22 | 2 | 0.882731863 | 0.088316517 | -3.321220091 | 5.21E-05 | 0.000580134 |
| *LOC_Os01g10580.1* | 1074 | 22 | 2 | 1.107113427 | 0.110765688 | -3.321220091 | 5.21E-05 | 0.000579946 |
| *LOC_Os07g45490.1* | 2895 | 281 | 26 | 5.246038459 | 0.534200529 | -3.295775075 | 2.67E-50 | 3.88E-48 |
| *LOC_Os04g16680.1* | 1179 | 332 | 31 | 15.21941631 | 1.563966414 | -3.282631594 | 6.05E-59 | 1.06E-56 |
| *LOC_Os09g30340.1* | 426 | 341 | 32 | 43.26318594 | 4.468069429 | -3.275416402 | 2.21E-60 | 3.97E-58 |
| *LOC_Os06g49350.1* | 6657 | 21 | 2 | 0.170496103 | 0.017870264 | -3.254105895 | 9.18E-05 | 0.000963587 |
| *LOC_Os11g07225.1* | 4017 | 21 | 2 | 0.282547313 | 0.029614725 | -3.254105895 | 9.18E-05 | 0.00096388 |
| *LOC_Os06g01210.1* | 465 | 546 | 52 | 63.46194934 | 6.651658198 | -3.254105895 | 3.63E-95 | 1.13E-92 |
| *LOC_Os10g29020.1* | 939 | 31 | 3 | 1.784307988 | 0.190035701 | -3.231022282 | 1.62E-06 | 2.48E-05 |
| *LOC_Os12g15314.1* | 1488 | 340 | 33 | 12.34950937 | 1.319138945 | -3.226785289 | 2.09E-59 | 3.68E-57 |
| *LOC_Os01g43430.1* | 399 | 41 | 4 | 5.553728939 | 0.596302499 | -3.219340477 | 3.02E-08 | 6.07E-07 |
| *LOC_Os03g19380.1* | 396 | 111 | 11 | 15.14961203 | 1.652254841 | -3.19677272 | 3.55E-20 | 1.93E-18 |
| *LOC_Os03g21560.1* | 498 | 50 | 5 | 5.426432184 | 0.597200545 | -3.183716568 | 1.00E-09 | 2.46E-08 |
| *LOC_Os01g38190.1* | 1308 | 128 | 13 | 5.289028947 | 0.59117375 | -3.161348755 | 7.71E-23 | 4.83E-21 |
| *LOC_Os01g04860.1* | 972 | 59 | 6 | 3.280646716 | 0.367167742 | -3.159469021 | 3.39E-11 | 9.57E-10 |
| *LOC_Os10g39800.1* | 948 | 49 | 5 | 2.793582239 | 0.313719274 | -3.154570222 | 1.75E-09 | 4.14E-08 |
| *LOC_Os04g12600.1* | 2415 | 39 | 4 | 0.872812968 | 0.098519543 | -3.147190692 | 9.19E-08 | 1.73E-06 |
| *LOC_Os12g38020.1* | 4299 | 87 | 9 | 1.093768787 | 0.12452444 | -3.134806967 | 7.98E-16 | 3.29E-14 |
| *LOC_Os12g31410.1* | 696 | 29 | 3 | 2.251969356 | 0.256384372 | -3.134806967 | 4.97E-06 | 6.94E-05 |
| *LOC_Os02g21230.1* | 1257 | 29 | 3 | 1.24691382 | 0.141959843 | -3.134806967 | 4.97E-06 | 6.94E-05 |
| *LOC_Os04g54600.1* | 906 | 29 | 3 | 1.729989704 | 0.196957531 | -3.134806967 | 4.97E-06 | 6.95E-05 |
| *LOC_Os03g13140.1* | 501 | 57 | 6 | 6.149089979 | 0.712349392 | -3.109715986 | 1.02E-10 | 2.77E-09 |
| *LOC_Os02g45520.1* | 2094 | 38 | 4 | 0.980800407 | 0.113622109 | -3.109715986 | 1.60E-07 | 2.90E-06 |
| *LOC_Os05g28090.1* | 594 | 28 | 3 | 2.547682504 | 0.300409971 | -3.084180894 | 8.65E-06 | 0.000114371 |
| *LOC_Os10g40030.6* | 882 | 28 | 3 | 1.715786176 | 0.202316919 | -3.084180894 | 8.65E-06 | 0.000114328 |
| *LOC_Os03g03720.1* | 1335 | 399 | 43 | 16.1534521 | 1.915873029 | -3.075768654 | 2.02E-66 | 4.00E-64 |
| *LOC_Os09g26810.1* | 795 | 37 | 4 | 2.515407281 | 0.299276349 | -3.071241838 | 2.78E-07 | 4.86E-06 |
| *LOC_Os09g01140.1* | 1194 | 37 | 4 | 1.674831481 | 0.199266915 | -3.071241838 | 2.78E-07 | 4.86E-06 |
| *LOC_Os03g46070.1* | 690 | 494 | 54 | 38.69470824 | 4.655048421 | -3.055268202 | 2.48E-81 | 6.53E-79 |
| *LOC_Os08g10020.2* | 393 | 99 | 11 | 13.61495977 | 1.664867473 | -3.031713474 | 2.59E-17 | 1.19E-15 |
| *LOC_Os02g46980.1* | 1842 | 27 | 3 | 0.792223747 | 0.096874877 | -3.031713474 | 1.50E-05 | 0.000189175 |
| *LOC_Os08g08500.1* | 690 | 36 | 4 | 2.819857281 | 0.344818402 | -3.031713474 | 4.81E-07 | 8.05E-06 |
| *LOC_Os06g15400.1* | 471 | 126 | 14 | 14.45850389 | 1.768017919 | -3.031713474 | 1.12E-21 | 6.70E-20 |
| *LOC_Os10g10170.1* | 2793 | 53 | 6 | 1.025601511 | 0.127779107 | -3.004746427 | 9.15E-10 | 2.26E-08 |
| *LOC_Os07g46060.1* | 3369 | 35 | 4 | 0.561488352 | 0.070621756 | -2.99107149 | 8.31E-07 | 1.34E-05 |
| *LOC_Os11g07020.6* | 618 | 61 | 7 | 5.334762358 | 0.673734984 | -2.985170888 | 5.35E-11 | 1.49E-09 |
| *LOC_Os10g30770.1* | 1581 | 633 | 73 | 21.63941712 | 2.746442582 | -2.978025603 | 3.42E-101 | 1.22E-98 |
| *LOC_Os07g34260.1* | 1200 | 60 | 7 | 2.702363227 | 0.346973517 | -2.961324146 | 9.19E-11 | 2.51E-09 |
| *LOC_Os04g24460.1* | 1089 | 76 | 9 | 3.771893577 | 0.491579953 | -2.939790985 | 3.20E-13 | 1.08E-11 |
| *LOC_Os08g44680.1* | 612 | 801 | 95 | 70.73833154 | 9.233188816 | -2.937591297 | 7.21E-126 | 3.19E-123 |
| *LOC_Os07g37030.1* | 678 | 612 | 74 | 48.78602641 | 6.492045569 | -2.90972295 | 1.17E-95 | 3.78E-93 |
| *LOC_Os10g37180.1* | 495 | 82 | 10 | 8.953284228 | 1.201639884 | -2.897412382 | 5.56E-14 | 1.99E-12 |
| *LOC_Os02g57700.1* | 2601 | 787 | 96 | 16.35340146 | 2.195383595 | -2.897045797 | 5.57E-122 | 2.43E-119 |
| *LOC_Os10g07970.1* | 1230 | 57 | 7 | 2.504629333 | 0.338510748 | -2.887323565 | 4.62E-10 | 1.17E-08 |
| *LOC_Os08g06530.1* | 783 | 89 | 11 | 6.143303378 | 0.835623138 | -2.878090285 | 5.65E-15 | 2.21E-13 |
| *LOC_Os10g39300.1* | 1185 | 1469 | 182 | 67.00036424 | 9.135505246 | -2.874612513 | 2.47E-224 | 1.94E-221 |
| *LOC_Os02g22020.1* | 1239 | 450 | 56 | 19.6297571 | 2.688414656 | -2.868214742 | 9.23E-70 | 1.90E-67 |
| *LOC_Os11g43760.1* | 1923 | 32 | 4 | 0.899382457 | 0.123725792 | -2.861788473 | 4.22E-06 | 6.00E-05 |
| *LOC_Os01g61044.1* | 1380 | 40 | 5 | 1.566587378 | 0.215511501 | -2.861788473 | 2.37E-07 | 4.19E-06 |
| *LOC_Os12g14580.1* | 933 | 87 | 11 | 5.03977708 | 0.701278582 | -2.84530035 | 1.64E-14 | 6.17E-13 |
| *LOC_Os05g38570.1* | 1611 | 71 | 9 | 2.381971312 | 0.332297063 | -2.841610591 | 4.64E-12 | 1.41E-10 |
| *LOC_Os06g21920.1* | 636 | 84 | 11 | 7.138317959 | 1.028762448 | -2.794674277 | 8.02E-14 | 2.85E-12 |
| *LOC_Os04g46880.1* | 1548 | 30 | 4 | 1.047427608 | 0.153698125 | -2.768679068 | 1.23E-05 | 0.000157581 |
| *LOC_Os01g31690.1* | 1002 | 277 | 37 | 14.94120986 | 2.196410627 | -2.766077273 | 5.55E-42 | 6.84E-40 |
| *LOC_Os01g64960.1* | 807 | 89 | 12 | 5.960602906 | 0.884478428 | -2.752559403 | 2.32E-14 | 8.55E-13 |
| *LOC_Os10g18820.1* | 615 | 111 | 15 | 9.754872138 | 1.450760348 | -2.749313743 | 1.44E-17 | 6.72E-16 |
| *LOC_Os06g45630.1* | 3186 | 36 | 5 | 0.610703554 | 0.093347731 | -2.709785379 | 1.98E-06 | 2.99E-05 |
| *LOC_Os01g61630.1* | 1998 | 36 | 5 | 0.973824586 | 0.148851787 | -2.709785379 | 1.98E-06 | 2.99E-05 |
| *LOC_Os02g17760.1* | 1620 | 43 | 6 | 1.434587886 | 0.220300645 | -2.703090727 | 1.92E-07 | 3.43E-06 |
| *LOC_Os01g59080.1* | 630 | 207 | 29 | 17.75838692 | 2.738022308 | -2.697294435 | 6.02E-31 | 5.31E-29 |
| *LOC_Os02g47020.1* | 1212 | 396 | 56 | 17.65900723 | 2.748305082 | -2.683790171 | 1.68E-57 | 2.83E-55 |
| *LOC_Os02g14590.1* | 1416 | 28 | 4 | 1.06873122 | 0.168025916 | -2.669143395 | 3.53E-05 | 0.000407176 |
| *LOC_Os04g53200.2* | 783 | 35 | 5 | 2.415905823 | 0.379828699 | -2.669143395 | 3.35E-06 | 4.84E-05 |
| *LOC_Os10g41999.1* | 369 | 70 | 10 | 10.25286861 | 1.611955942 | -2.669143395 | 3.09E-11 | 8.76E-10 |
| *LOC_Os09g16380.1* | 4161 | 35 | 5 | 0.454615299 | 0.071474615 | -2.669143395 | 3.35E-06 | 4.84E-05 |
| *LOC_Os02g15750.1* | 1437 | 140 | 20 | 5.265565092 | 0.827852112 | -2.669143395 | 3.63E-21 | 2.09E-19 |
| *LOC_Os01g19320.1* | 411 | 42 | 6 | 5.523078129 | 0.86833831 | -2.669143395 | 3.23E-07 | 5.58E-06 |
| *LOC_Os02g53240.1* | 411 | 84 | 12 | 11.04615626 | 1.736676621 | -2.669143395 | 3.13E-13 | 1.06E-11 |
| *LOC_Os05g12240.1* | 1179 | 56 | 8 | 2.567130462 | 0.403604236 | -2.669143395 | 3.12E-09 | 7.22E-08 |
| *LOC_Os12g26380.1* | 591 | 615 | 89 | 56.24207732 | 8.957401878 | -2.650497642 | 3.26E-87 | 9.29E-85 |
| *LOC_Os12g08730.1* | 519 | 386 | 56 | 40.19700215 | 6.418007243 | -2.646890588 | 2.85E-55 | 4.57E-53 |
| *LOC_Os02g02000.1* | 1464 | 55 | 8 | 2.030464174 | 0.325033739 | -2.643148186 | 5.23E-09 | 1.17E-07 |
| *LOC_Os12g13470.1* | 609 | 273 | 40 | 24.22808411 | 3.906809476 | -2.632617519 | 2.53E-39 | 2.90E-37 |
| *LOC_Os11g24450.1* | 930 | 34 | 5 | 1.9759215 | 0.31979126 | -2.627323219 | 5.64E-06 | 7.80E-05 |
| *LOC_Os06g37750.1* | 2409 | 178 | 27 | 3.993529718 | 0.666663223 | -2.582634402 | 1.23E-25 | 8.63E-24 |
| *LOC_Os11g07160.1* | 3039 | 59 | 9 | 1.049288782 | 0.176153527 | -2.574506521 | 2.43E-09 | 5.67E-08 |
| *LOC_Os12g33830.1* | 1128 | 39 | 6 | 1.868655423 | 0.316389225 | -2.562228191 | 1.52E-06 | 2.34E-05 |
| *LOC_Os12g38290.1* | 231 | 2320 | 360 | 542.8123539 | 92.69793392 | -2.549844466 | 0 | 0 |
| *LOC_Os08g20420.1* | 1185 | 45 | 7 | 2.052427768 | 0.351365586 | -2.546286647 | 2.48E-07 | 4.36E-06 |
| *LOC_Os02g52590.1* | 1524 | 38 | 6 | 1.347635205 | 0.234177851 | -2.524753485 | 2.54E-06 | 3.77E-05 |
| *LOC_Os11g05400.1* | 1719 | 2809 | 445 | 88.3180722 | 15.39797705 | -2.519967856 | 0 | 0 |
| *LOC_Os05g39320.1* | 1818 | 309 | 49 | 9.186251235 | 1.603177964 | -2.518541657 | 3.08E-42 | 3.85E-40 |
| *LOC_Os05g39310.1* | 1818 | 126 | 20 | 3.745850018 | 0.654358353 | -2.517140301 | 4.52E-18 | 2.16E-16 |
| *LOC_Os07g10230.1* | 852 | 75 | 12 | 4.757681738 | 0.837763018 | -2.505644662 | 3.09E-11 | 8.77E-10 |
| *LOC_Os10g31670.1* | 582 | 393 | 63 | 36.49583328 | 6.438683812 | -2.502894051 | 6.35E-53 | 9.77E-51 |
| *LOC_Os10g23900.1* | 1581 | 56 | 9 | 1.914387612 | 0.33860251 | -2.499218393 | 1.11E-08 | 2.39E-07 |
| *LOC_Os01g65150.1* | 1614 | 448 | 72 | 15.00196686 | 2.653435283 | -2.499218393 | 5.26E-60 | 9.41E-58 |
| *LOC_Os02g40200.1* | 3096 | 112 | 18 | 1.955198201 | 0.345820781 | -2.499218393 | 4.28E-16 | 1.81E-14 |
| *LOC_Os09g17740.1* | 798 | 2398 | 386 | 162.4127073 | 28.77159557 | -2.496947379 | 0 | 0 |
| *LOC_Os02g19650.1* | 1191 | 31 | 5 | 1.406771789 | 0.249711059 | -2.494056688 | 2.64E-05 | 0.000313829 |
| *LOC_Os12g33610.1* | 2043 | 111 | 18 | 2.936488676 | 0.524063209 | -2.486279338 | 7.07E-16 | 2.93E-14 |
| *LOC_Os10g39320.1* | 1242 | 172 | 28 | 7.484806363 | 1.34096045 | -2.480698305 | 8.76E-24 | 5.67E-22 |
| *LOC_Os05g40180.1* | 1776 | 49 | 8 | 1.491168898 | 0.267933217 | -2.476498317 | 1.12E-07 | 2.07E-06 |
| *LOC_Os07g14030.1* | 531 | 85 | 14 | 8.651633685 | 1.568241883 | -2.463824487 | 2.37E-12 | 7.45E-11 |
| *LOC_Os01g01340.1* | 387 | 404 | 67 | 56.42143379 | 10.29777436 | -2.453910765 | 3.57E-53 | 5.52E-51 |
| *LOC_Os01g02130.1* | 528 | 72 | 12 | 7.370081529 | 1.35184487 | -2.446750973 | 1.39E-10 | 3.70E-09 |
| *LOC_Os09g18470.1* | 849 | 30 | 5 | 1.909797334 | 0.35030138 | -2.446750973 | 4.38E-05 | 0.000495367 |
| *LOC_Os01g64120.1* | 498 | 77 | 13 | 8.356705563 | 1.552721417 | -2.428135295 | 3.82E-11 | 1.07E-09 |
| *LOC_Os02g51080.1* | 1392 | 337 | 57 | 13.0847185 | 2.435651533 | -2.42550324 | 4.84E-44 | 6.23E-42 |
| *LOC_Os01g43760.1* | 1416 | 47 | 8 | 1.793941691 | 0.336051832 | -2.416377324 | 3.04E-07 | 5.28E-06 |
| *LOC_Os03g50310.1* | 1266 | 47 | 8 | 2.006494024 | 0.3758684 | -2.416377324 | 3.04E-07 | 5.28E-06 |
| *LOC_Os02g07360.1* | 1788 | 99 | 17 | 2.992549883 | 0.565536892 | -2.403682252 | 8.21E-14 | 2.91E-12 |
| *LOC_Os07g05360.1* | 414 | 192 | 33 | 25.06539805 | 4.741253021 | -2.402356854 | 1.79E-25 | 1.25E-23 |
| *LOC_Os01g06920.1* | 2991 | 64 | 11 | 1.156477744 | 0.218753901 | -2.402356854 | 2.25E-09 | 5.26E-08 |
| *LOC_Os06g39690.1* | 1146 | 29 | 5 | 1.367688195 | 0.259516467 | -2.397841373 | 7.24E-05 | 0.000779975 |
| *LOC_Os12g14699.1* | 1965 | 856 | 148 | 23.54425367 | 4.480007019 | -2.393802093 | 1.17E-107 | 4.60E-105 |
| *LOC_Os02g12660.1* | 2379 | 69 | 12 | 1.567575138 | 0.300031144 | -2.385350429 | 6.14E-10 | 1.53E-08 |
| *LOC_Os09g06464.1* | 1008 | 69 | 12 | 3.699663942 | 0.708109217 | -2.385350429 | 6.14E-10 | 1.53E-08 |
| *LOC_Os05g04240.1* | 1098 | 40 | 7 | 1.968934956 | 0.379206029 | -2.376361646 | 3.07E-06 | 4.46E-05 |
| *LOC_Os02g05890.1* | 498 | 51 | 9 | 5.534960827 | 1.074960981 | -2.364288813 | 1.34E-07 | 2.47E-06 |
| *LOC_Os03g57149.1* | 933 | 34 | 6 | 1.969568054 | 0.38251559 | -2.364288813 | 1.90E-05 | 0.000233458 |
| *LOC_Os10g09990.1* | 1587 | 45 | 8 | 1.532531131 | 0.299842088 | -2.353641569 | 8.19E-07 | 1.32E-05 |
| *LOC_Os10g26700.1* | 681 | 39 | 7 | 3.095217794 | 0.611407078 | -2.33983577 | 5.03E-06 | 7.02E-05 |
| *LOC_Os02g18080.1* | 3558 | 44 | 8 | 0.668375391 | 0.133740695 | -2.321220091 | 1.34E-06 | 2.07E-05 |
| *LOC_Os03g63560.1* | 1548 | 49 | 9 | 1.710798426 | 0.345820781 | -2.306573315 | 3.58E-07 | 6.13E-06 |
| *LOC_Os12g35450.1* | 390 | 38 | 7 | 5.266143725 | 1.06761082 | -2.302361064 | 8.21E-06 | 0.000109119 |
| *LOC_Os10g42020.1* | 408 | 276 | 51 | 36.56138484 | 7.435146783 | -2.297887588 | 3.43E-34 | 3.47E-32 |
| *LOC_Os09g10750.1* | 720 | 54 | 10 | 4.053544841 | 0.82612742 | -2.29474788 | 9.62E-08 | 1.80E-06 |
| *LOC_Os11g06980.1* | 1887 | 1932 | 358 | 55.33615003 | 11.28471669 | -2.293852074 | 9.24E-229 | 7.41E-226 |
| *LOC_Os12g16340.1* | 2688 | 145 | 27 | 2.915496042 | 0.597467152 | -2.286810061 | 1.48E-18 | 7.25E-17 |
| *LOC_Os10g39270.1* | 1194 | 32 | 6 | 1.448502902 | 0.298900373 | -2.276825972 | 5.08E-05 | 0.000567024 |
| *LOC_Os09g03680.1* | 1218 | 64 | 12 | 2.839921947 | 0.586021421 | -2.276825972 | 7.01E-09 | 1.55E-07 |
| *LOC_Os03g29250.1* | 744 | 170 | 32 | 12.34950937 | 2.558330076 | -2.271179409 | 2.40E-21 | 1.40E-19 |
| *LOC_Os05g14360.1* | 1431 | 37 | 7 | 1.397448489 | 0.290963117 | -2.263886916 | 1.34E-05 | 0.000169718 |
| *LOC_Os07g42324.1* | 363 | 289 | 55 | 43.02936489 | 9.012299131 | -2.255354442 | 5.80E-35 | 5.99E-33 |
| *LOC_Os04g14990.1* | 1197 | 177 | 34 | 7.991951399 | 1.689523747 | -2.241931182 | 7.65E-22 | 4.60E-20 |
| *LOC_Os01g52230.1* | 825 | 281 | 54 | 18.40882586 | 3.893313225 | -2.241327291 | 8.61E-34 | 8.61E-32 |
| *LOC_Os05g15770.1* | 894 | 9142 | 1765 | 552.6846672 | 117.4320722 | -2.234630108 | 0 | 0 |
| *LOC_Os12g44050.1* | 408 | 128 | 25 | 16.95600456 | 3.644679796 | -2.217932283 | 5.41E-16 | 2.27E-14 |
| *LOC_Os04g13000.1* | 2754 | 51 | 10 | 1.000875269 | 0.215981025 | -2.21228572 | 4.07E-07 | 6.89E-06 |
| *LOC_Os10g38350.1* | 696 | 172 | 34 | 13.35650791 | 2.905689548 | -2.200590386 | 8.07E-21 | 4.58E-19 |
| *LOC_Os09g24530.1* | 1488 | 96 | 19 | 3.486920293 | 0.759504241 | -2.19882346 | 3.24E-12 | 1.00E-10 |
| *LOC_Os04g09390.1* | 684 | 106 | 21 | 8.375745676 | 1.826176403 | -2.197391504 | 2.43E-13 | 8.33E-12 |
| *LOC_Os01g47070.1* | 906 | 682 | 136 | 40.68458546 | 8.928741391 | -2.18795356 | 2.08E-77 | 4.95E-75 |
| *LOC_Os05g45030.1* | 816 | 155 | 31 | 10.26633089 | 2.259701473 | -2.183716568 | 9.75E-19 | 4.87E-17 |
| *LOC_Os12g01449.1* | 2790 | 65 | 13 | 1.259165662 | 0.277152425 | -2.183716568 | 1.28E-08 | 2.73E-07 |
| *LOC_Os01g47030.1* | 6087 | 50 | 10 | 0.443956502 | 0.097718374 | -2.183716568 | 6.54E-07 | 1.07E-05 |
| *LOC_Os08g27010.1* | 816 | 50 | 10 | 3.311719641 | 0.728935959 | -2.183716568 | 6.54E-07 | 1.07E-05 |
| *LOC_Os12g35630.1* | 3372 | 277 | 56 | 4.439825706 | 0.987824958 | -2.168175717 | 4.69E-32 | 4.39E-30 |
| *LOC_Os03g05640.1* | 1587 | 9934 | 2019 | 338.3147612 | 75.67264704 | -2.160522309 | 0 | 0 |
| *LOC_Os06g36650.1* | 3588 | 54 | 11 | 0.813420369 | 0.182355885 | -2.157244356 | 2.81E-07 | 4.91E-06 |
| *LOC_Os08g39430.1* | 732 | 34 | 7 | 2.510392069 | 0.568809044 | -2.141896392 | 5.63E-05 | 0.000622239 |
| *LOC_Os02g39620.1* | 237 | 97 | 20 | 22.12061039 | 5.019508377 | -2.13977322 | 5.76E-12 | 1.74E-10 |
| *LOC_Os07g39210.1* | 495 | 43 | 9 | 4.6950149 | 1.081475896 | -2.118128226 | 6.29E-06 | 8.63E-05 |
| *LOC_Os04g45090.2* | 711 | 143 | 30 | 10.87026558 | 2.509754189 | -2.114769214 | 9.06E-17 | 4.00E-15 |
| *LOC_Os12g31200.1* | 3027 | 529 | 111 | 9.445326378 | 2.181172892 | -2.114496518 | 5.88E-58 | 9.99E-56 |
| *LOC_Os06g40120.1* | 888 | 1923 | 404 | 117.0415425 | 27.06125496 | -2.112720037 | 9.22E-206 | 6.63E-203 |
| *LOC_Os12g36220.1* | 282 | 732 | 154 | 140.2928995 | 32.48262708 | -2.11070177 | 2.30E-79 | 5.66E-77 |
| *LOC_Os12g26290.1* | 1857 | 38 | 8 | 1.105975257 | 0.256246308 | -2.109715986 | 2.37E-05 | 0.00028531 |
| *LOC_Os11g34570.1* | 681 | 66 | 14 | 5.238060881 | 1.222814155 | -2.09882767 | 2.24E-08 | 4.59E-07 |
| *LOC_Os11g47970.1* | 1401 | 466 | 99 | 17.97717722 | 4.20316649 | -2.096617997 | 1.01E-50 | 1.48E-48 |
| *LOC_Os02g04340.1* | 2010 | 80 | 17 | 2.151134907 | 0.503074608 | -2.096253726 | 7.12E-10 | 1.77E-08 |
| *LOC_Os06g24180.1* | 1590 | 80 | 17 | 2.719359223 | 0.635962241 | -2.096253726 | 7.12E-10 | 1.77E-08 |
| *LOC_Os10g13700.2* | 1605 | 512 | 109 | 17.24124576 | 4.039531461 | -2.093604148 | 1.83E-55 | 2.94E-53 |
| *LOC_Os01g42330.1* | 3783 | 498 | 107 | 7.114865912 | 1.68239113 | -2.080323418 | 1.43E-53 | 2.23E-51 |
| *LOC_Os02g14150.1* | 2757 | 51 | 11 | 0.999786178 | 0.237320608 | -2.074782196 | 1.14E-06 | 1.79E-05 |
| *LOC_Os08g14450.1* | 1719 | 120 | 26 | 3.772932953 | 0.899657086 | -2.06823935 | 6.17E-14 | 2.21E-12 |
| *LOC_Os07g44160.1* | 1656 | 322 | 70 | 10.50919033 | 2.514300845 | -2.063422334 | 7.38E-35 | 7.60E-33 |
| *LOC_Os12g31160.1* | 2808 | 730 | 159 | 14.05074897 | 3.368057945 | -2.060658171 | 8.18E-77 | 1.89E-74 |
| *LOC_Os03g17070.1* | 636 | 257 | 56 | 21.83985376 | 5.237336099 | -2.0600581 | 4.45E-28 | 3.51E-26 |
| *LOC_Os02g31030.1* | 1164 | 87 | 19 | 4.039615134 | 0.970912638 | -2.056804455 | 2.10E-10 | 5.48E-09 |
| *LOC_Os04g18530.1* | 324 | 50 | 11 | 8.340627245 | 2.019422583 | -2.046213044 | 1.80E-06 | 2.74E-05 |
| *LOC_Os03g46060.1* | 669 | 1470 | 324 | 118.7585634 | 28.8070261 | -2.04353891 | 3.16E-151 | 1.78E-148 |
| *LOC_Os10g37920.1* | 1620 | 113 | 25 | 3.769963515 | 0.917919356 | -2.038111245 | 5.54E-13 | 1.84E-11 |
| *LOC_Os01g38229.2* | 1959 | 1286 | 290 | 35.47972548 | 8.805278478 | -2.01055431 | 8.44E-130 | 3.88E-127 |
| *LOC_Os04g24340.1* | 1089 | 62 | 14 | 3.077071076 | 0.764679926 | -2.008629861 | 1.39E-07 | 2.55E-06 |
| *LOC_Os07g48010.1* | 948 | 132 | 30 | 7.525568482 | 1.882315641 | -1.999291996 | 1.32E-14 | 5.02E-13 |
| *LOC_Os05g31160.1* | 1248 | 44 | 10 | 1.905512532 | 0.476611973 | -1.999291996 | 1.06E-05 | 0.000137486 |
| *LOC_Os05g49620.1* | 834 | 57 | 13 | 3.693877793 | 0.927164587 | -1.994238769 | 5.13E-07 | 8.53E-06 |
| *LOC_Os10g35100.1* | 315 | 377 | 86 | 64.68513884 | 16.23930472 | -1.993944431 | 7.48E-39 | 8.49E-37 |
| *LOC_Os09g36060.1* | 1548 | 35 | 8 | 1.221998875 | 0.307396249 | -1.99107149 | 9.49E-05 | 0.00099068 |
| *LOC_Os02g14460.1* | 1005 | 83 | 19 | 4.463604933 | 1.124519713 | -1.988900391 | 1.28E-09 | 3.07E-08 |
| *LOC_Os07g33200.1* | 1449 | 48 | 11 | 1.790385575 | 0.451547907 | -1.987319355 | 4.48E-06 | 6.32E-05 |
| *LOC_Os04g13260.1* | 336 | 48 | 11 | 7.721037793 | 1.947300348 | -1.987319355 | 4.48E-06 | 6.33E-05 |
| *LOC_Os07g41630.1* | 591 | 39 | 9 | 3.566570757 | 0.905804684 | -1.97726569 | 3.97E-05 | 0.000453351 |
| *LOC_Os02g57810.1* | 1596 | 39 | 9 | 1.320703833 | 0.335420156 | -1.97726569 | 3.97E-05 | 0.000453201 |
| *LOC_Os07g48690.1* | 2079 | 112 | 26 | 2.911637147 | 0.743872309 | -1.968703677 | 2.24E-12 | 7.08E-11 |
| *LOC_Os08g40680.1* | 894 | 43 | 10 | 2.599588787 | 0.66533752 | -1.966125133 | 1.67E-05 | 0.000207345 |
| *LOC_Os10g08620.1* | 1200 | 43 | 10 | 1.936693646 | 0.495676452 | -1.966125133 | 1.67E-05 | 0.00020727 |
| *LOC_Os06g47970.1* | 570 | 94 | 22 | 8.913057662 | 2.295764621 | -1.956945706 | 1.58E-10 | 4.18E-09 |
| *LOC_Os06g10830.1* | 2043 | 81 | 19 | 2.142843088 | 0.553177832 | -1.953710962 | 3.11E-09 | 7.21E-08 |
| *LOC_Os01g17170.1* | 1227 | 148 | 35 | 6.519148454 | 1.696692013 | -1.941958821 | 1.18E-15 | 4.82E-14 |
| *LOC_Os03g03990.1* | 1167 | 38 | 9 | 1.75989379 | 0.458723709 | -1.939790985 | 6.23E-05 | 0.000679938 |
| *LOC_Os07g36870.1* | 1245 | 63 | 15 | 2.734921821 | 0.716640654 | -1.932177801 | 2.26E-07 | 4.01E-06 |
| *LOC_Os08g05720.1* | 750 | 46 | 11 | 3.314898892 | 0.872390556 | -1.92591881 | 1.10E-05 | 0.000141728 |
| *LOC_Os10g40200.1* | 1146 | 209 | 50 | 9.856787339 | 2.595164671 | -1.925291415 | 2.74E-21 | 1.59E-19 |
| *LOC_Os10g38340.1* | 720 | 204 | 49 | 15.31339162 | 4.04802436 | -1.919503971 | 9.75E-21 | 5.50E-19 |
| *LOC_Os06g04150.1* | 981 | 66 | 16 | 3.636207401 | 0.970131283 | -1.906182592 | 1.50E-07 | 2.73E-06 |
| *LOC_Os01g58990.1* | 1536 | 41 | 10 | 1.442667869 | 0.387247228 | -1.897412382 | 4.07E-05 | 0.000463291 |
| *LOC_Os02g52670.1* | 729 | 41 | 10 | 3.039695263 | 0.815928316 | -1.897412382 | 4.07E-05 | 0.000463597 |
| *LOC_Os01g65290.1* | 1278 | 41 | 10 | 1.733910678 | 0.465423899 | -1.897412382 | 4.07E-05 | 0.000463444 |
| *LOC_Os01g38359.1* | 1269 | 319 | 78 | 13.5863494 | 3.656053265 | -1.893798868 | 4.84E-31 | 4.29E-29 |
| *LOC_Os01g66900.1* | 903 | 49 | 12 | 2.932797301 | 0.790447499 | -1.891535816 | 7.19E-06 | 9.70E-05 |
| *LOC_Os10g17489.1* | 1458 | 159 | 39 | 5.894043253 | 1.591060217 | -1.889269209 | 3.63E-16 | 1.54E-14 |
| *LOC_Os12g12130.1* | 3078 | 126 | 31 | 2.212461122 | 0.599063159 | -1.884872086 | 4.60E-13 | 1.54E-11 |
| *LOC_Os04g13210.1* | 3624 | 308 | 76 | 4.593420938 | 1.247397694 | -1.8806475 | 9.26E-30 | 7.75E-28 |
| *LOC_Os04g07600.1* | 2163 | 97 | 24 | 2.4237562 | 0.65998529 | -1.876738814 | 2.59E-10 | 6.74E-09 |
| *LOC_Os02g46030.1* | 1476 | 101 | 25 | 3.698356178 | 1.007472464 | -1.876143766 | 1.11E-10 | 3.00E-09 |
| *LOC_Os12g36410.1* | 1341 | 96 | 24 | 3.869155404 | 1.064540032 | -1.861788473 | 3.99E-10 | 1.02E-08 |
| *LOC_Os06g38640.1* | 1818 | 48 | 12 | 1.426990483 | 0.392615012 | -1.861788473 | 1.11E-05 | 0.00014371 |
| *LOC_Os10g34930.1* | 690 | 104 | 26 | 8.146254367 | 2.24131961 | -1.861788473 | 7.36E-11 | 2.02E-09 |
| *LOC_Os06g15680.1* | 1635 | 52 | 13 | 1.718934408 | 0.472939 | -1.861788473 | 4.70E-06 | 6.60E-05 |
| *LOC_Os08g20486.1* | 3399 | 76 | 19 | 1.208470758 | 0.332492589 | -1.861788473 | 2.77E-08 | 5.60E-07 |
| *LOC_Os04g53490.1* | 1029 | 59 | 15 | 3.09891993 | 0.867072511 | -1.837540926 | 1.30E-06 | 2.01E-05 |
| *LOC_Os03g40670.1* | 1176 | 1148 | 292 | 52.76042492 | 14.76913511 | -1.836870841 | 4.19E-103 | 1.55E-100 |
| *LOC_Os06g01950.1* | 1092 | 55 | 14 | 2.72216076 | 0.762579157 | -1.835793264 | 3.06E-06 | 4.45E-05 |
| *LOC_Os10g28080.1* | 861 | 548 | 140 | 34.39942041 | 9.671735653 | -1.830537539 | 6.69E-50 | 9.58E-48 |
| *LOC_Os12g36880.1* | 477 | 974 | 249 | 110.3606618 | 31.04992116 | -1.829564503 | 2.75E-87 | 7.91E-85 |
| *LOC_Os03g62630.1* | 660 | 129 | 33 | 10.56378353 | 2.974058713 | -1.828621609 | 7.26E-13 | 2.38E-11 |
| *LOC_Os09g23300.1* | 741 | 887 | 228 | 64.69625325 | 18.30189977 | -1.821688753 | 3.42E-79 | 8.37E-77 |
| *LOC_Os12g31660.1* | 669 | 136 | 35 | 10.98718681 | 3.111870104 | -1.819968297 | 2.07E-13 | 7.11E-12 |
| *LOC_Os04g39360.1* | 366 | 322 | 83 | 47.54977919 | 13.48890018 | -1.817665919 | 1.03E-29 | 8.60E-28 |
| *LOC_Os01g73170.1* | 1134 | 2553 | 660 | 121.6778363 | 34.61867285 | -1.813444081 | 4.80E-223 | 3.68E-220 |
| *LOC_Os06g49420.2* | 1191 | 58 | 15 | 2.632024638 | 0.749133177 | -1.812878872 | 1.99E-06 | 3.00E-05 |
| *LOC_Os02g29960.1* | 1614 | 150 | 39 | 5.022979977 | 1.437277445 | -1.805204944 | 1.67E-14 | 6.27E-13 |
| *LOC_Os02g52730.1* | 1998 | 361 | 94 | 9.765296548 | 2.798413604 | -1.803054648 | 8.91E-33 | 8.63E-31 |
| *LOC_Os09g17920.1* | 3213 | 142 | 37 | 2.3886435 | 0.684968393 | -1.802082227 | 8.94E-14 | 3.16E-12 |
| *LOC_Os10g35810.1* | 711 | 92 | 24 | 6.993457579 | 2.007803351 | -1.800387928 | 2.19E-09 | 5.13E-08 |
| *LOC_Os10g29470.1* | 1101 | 46 | 12 | 2.258105512 | 0.648296177 | -1.800387928 | 2.64E-05 | 0.000314153 |
| *LOC_Os01g72740.1* | 1572 | 304 | 80 | 10.45188831 | 3.027031769 | -1.787787891 | 1.52E-27 | 1.17E-25 |
| *LOC_Os02g55610.1* | 522 | 57 | 15 | 5.901712796 | 1.709229146 | -1.787787891 | 3.05E-06 | 4.44E-05 |
| *LOC_Os01g50680.1* | 1281 | 91 | 24 | 3.839423165 | 1.114401391 | -1.784620612 | 3.34E-09 | 7.70E-08 |
| *LOC_Os02g26330.1* | 396 | 60 | 16 | 8.188979477 | 2.403279768 | -1.768679068 | 1.97E-06 | 2.98E-05 |
| *LOC_Os10g38640.1* | 714 | 485 | 131 | 36.71277774 | 10.91321265 | -1.750206408 | 8.45E-42 | 1.03E-39 |
| *LOC_Os08g06010.1* | 1500 | 4780 | 1300 | 172.2306164 | 51.55035103 | -1.740287468 | 0 | 0 |
| *LOC_Os05g15530.1* | 1023 | 44 | 12 | 2.324613529 | 0.697726384 | -1.736257591 | 6.17E-05 | 0.000674692 |
| *LOC_Os01g47280.1* | 582 | 44 | 12 | 4.086047492 | 1.226415964 | -1.736257591 | 6.17E-05 | 0.000674906 |
| *LOC_Os07g44010.1* | 513 | 128 | 35 | 13.48547731 | 4.058169784 | -1.732505456 | 5.78E-12 | 1.74E-10 |
| *LOC_Os05g39230.1* | 2601 | 87 | 24 | 1.80780931 | 0.548845899 | -1.719769468 | 1.76E-08 | 3.67E-07 |
| *LOC_Os07g36690.1* | 2670 | 58 | 16 | 1.174060428 | 0.356441494 | -1.719769468 | 4.56E-06 | 6.43E-05 |
| *LOC_Os07g08610.1* | 1338 | 76 | 21 | 3.069949257 | 0.933561031 | -1.717398563 | 1.47E-07 | 2.69E-06 |
| *LOC_Os03g03910.1* | 1479 | 159 | 44 | 5.810355012 | 1.76955488 | -1.715239809 | 2.40E-14 | 8.82E-13 |
| *LOC_Os06g51060.1* | 1050 | 981 | 273 | 50.49558716 | 15.46510531 | -1.707140658 | 5.00E-80 | 1.26E-77 |
| *LOC_Os10g38360.1* | 702 | 2430 | 678 | 187.086685 | 57.44762985 | -1.703387608 | 7.18E-195 | 4.77E-192 |
| *LOC_Os01g49740.1* | 1830 | 57 | 16 | 1.683439388 | 0.520053983 | -1.694678487 | 6.88E-06 | 9.37E-05 |
| *LOC_Os05g33400.1* | 1314 | 579 | 163 | 23.81534716 | 7.378562714 | -1.690479856 | 2.62E-47 | 3.59E-45 |
| *LOC_Os01g39770.1* | 873 | 117 | 33 | 7.243447826 | 2.248429268 | -1.687759073 | 1.05E-10 | 2.83E-09 |
| *LOC_Os07g35740.1* | 1992 | 85 | 24 | 2.306233678 | 0.716640654 | -1.686216908 | 3.98E-08 | 7.89E-07 |
| *LOC_Os05g01950.1* | 396 | 46 | 13 | 6.278217599 | 1.952664812 | -1.684910711 | 5.98E-05 | 0.000657366 |
| *LOC_Os06g48000.1* | 906 | 162 | 46 | 9.664080416 | 3.020015471 | -1.67807652 | 3.42E-14 | 1.25E-12 |
| *LOC_Os02g42450.1* | 579 | 771 | 220 | 71.96967352 | 22.6007916 | -1.671015809 | 1.91E-61 | 3.56E-59 |
| *LOC_Os10g02750.1* | 1008 | 140 | 40 | 7.506564521 | 2.360364058 | -1.669143395 | 2.26E-12 | 7.14E-11 |
| *LOC_Os05g43390.1* | 1452 | 63 | 18 | 2.345025941 | 0.737369929 | -1.669143395 | 2.83E-06 | 4.15E-05 |
| *LOC_Os01g67010.1* | 1320 | 517 | 148 | 21.16851195 | 6.669101357 | -1.666355577 | 1.34E-41 | 1.62E-39 |
| *LOC_Os07g39990.1* | 492 | 87 | 25 | 9.557138243 | 3.022417392 | -1.660875779 | 3.85E-08 | 7.67E-07 |
| *LOC_Os01g67030.1* | 1257 | 739 | 213 | 31.7748039 | 10.07914886 | -1.656509407 | 2.91E-58 | 5.04E-56 |
| *LOC_Os06g37450.1* | 1173 | 222 | 64 | 10.22889406 | 3.245349662 | -1.656204339 | 1.35E-18 | 6.63E-17 |
| *LOC_Os05g35470.1* | 936 | 249 | 72 | 14.3779582 | 4.575474944 | -1.651865403 | 1.31E-20 | 7.34E-19 |
| *LOC_Os04g10000.1* | 825 | 138 | 40 | 9.040633343 | 2.883935722 | -1.648384835 | 5.03E-12 | 1.53E-10 |
| *LOC_Os01g51570.1* | 1017 | 6505 | 1887 | 345.7005466 | 110.3647747 | -1.647243107 | 0 | 0 |
| *LOC_Os07g05000.1* | 1134 | 55 | 16 | 2.621339991 | 0.839240554 | -1.643148186 | 1.56E-05 | 0.000195545 |
| *LOC_Os07g37420.1* | 798 | 79 | 23 | 5.350543734 | 1.714369684 | -1.642007265 | 2.07E-07 | 3.69E-06 |
| *LOC_Os04g45520.1* | 570 | 247 | 72 | 23.4204813 | 7.513411486 | -1.640230703 | 2.89E-20 | 1.58E-18 |
| *LOC_Os06g31800.1* | 408 | 1011 | 297 | 133.9259423 | 43.29879597 | -1.629036634 | 3.07E-77 | 7.25E-75 |
| *LOC_Os05g33200.1* | 873 | 51 | 15 | 3.157400334 | 1.022013304 | -1.627323219 | 3.65E-05 | 0.000419054 |
| *LOC_Os06g36840.1* | 1074 | 149 | 44 | 7.498177298 | 2.436845128 | -1.621525375 | 1.31E-12 | 4.22E-11 |
| *LOC_Os11g05380.1* | 1563 | 47 | 14 | 1.625221647 | 0.532780832 | -1.609022402 | 8.59E-05 | 0.000909344 |
| *LOC_Os01g71190.1* | 543 | 77 | 23 | 7.664160903 | 2.51946042 | -1.605013057 | 4.59E-07 | 7.71E-06 |
| *LOC_Os03g15870.1* | 972 | 137 | 41 | 7.617772884 | 2.508979573 | -1.602268551 | 1.57E-11 | 4.59E-10 |
| *LOC_Os10g35230.1* | 1386 | 50 | 15 | 1.949757018 | 0.643735652 | -1.598754067 | 5.45E-05 | 0.000605725 |
| *LOC_Os01g04920.1* | 1545 | 970 | 293 | 33.9325868 | 11.28024858 | -1.588872555 | 1.26E-71 | 2.69E-69 |
| *LOC_Os04g08370.1* | 2871 | 142 | 43 | 2.673184105 | 0.890870949 | -1.585270838 | 9.66E-12 | 2.86E-10 |
| *LOC_Os02g02190.1* | 1602 | 66 | 20 | 2.22666633 | 0.742586445 | -1.584254497 | 3.85E-06 | 5.51E-05 |
| *LOC_Os08g25900.1* | 825 | 56 | 17 | 3.668662806 | 1.225672682 | -1.581680554 | 2.20E-05 | 0.000266697 |
| *LOC_Os02g53130.1* | 2670 | 295 | 90 | 5.971514248 | 2.004983402 | -1.574506521 | 1.23E-22 | 7.63E-21 |
| *LOC_Os04g55600.1* | 333 | 121 | 37 | 19.63879583 | 6.609019363 | -1.571198344 | 4.30E-10 | 1.09E-08 |
| *LOC_Os03g43810.1* | 1485 | 49 | 15 | 1.783377753 | 0.600819942 | -1.569607721 | 8.11E-05 | 0.000863484 |
| *LOC_Os01g72160.1* | 687 | 147 | 45 | 11.56469853 | 3.896146786 | -1.569607721 | 5.91E-12 | 1.78E-10 |
| *LOC_Os03g21370.1* | 621 | 209 | 64 | 18.18982011 | 6.130104916 | -1.569147605 | 2.14E-16 | 9.24E-15 |
| *LOC_Os07g33660.1* | 519 | 124 | 38 | 12.9130266 | 4.355076343 | -1.56805727 | 2.77E-10 | 7.17E-09 |
| *LOC_Os09g32840.1* | 1893 | 702 | 217 | 20.04288416 | 6.818496997 | -1.555564461 | 8.69E-51 | 1.28E-48 |
| *LOC_Os07g01030.1* | 1440 | 794 | 246 | 29.80106115 | 10.16136727 | -1.552269165 | 4.78E-57 | 8.00E-55 |
| *LOC_Os02g10780.1* | 843 | 2076 | 648 | 133.0986017 | 45.72218378 | -1.541529198 | 9.09E-145 | 4.75E-142 |
| *LOC_Os01g25240.1* | 1179 | 64 | 20 | 2.933863385 | 1.00901059 | -1.539860378 | 8.40E-06 | 0.000111267 |
| *LOC_Os07g28400.1* | 330 | 307 | 96 | 50.28033399 | 17.30361433 | -1.538920817 | 8.89E-23 | 5.52E-21 |
| *LOC_Os05g10650.1* | 1629 | 389 | 122 | 12.90631425 | 4.454698134 | -1.53467748 | 2.44E-28 | 1.95E-26 |
| *LOC_Os01g25484.2* | 1791 | 573 | 180 | 17.2915034 | 5.978007464 | -1.532326705 | 6.82E-41 | 8.11E-39 |
| *LOC_Os11g04490.1* | 1014 | 54 | 17 | 2.878256692 | 0.997218898 | -1.529213134 | 4.82E-05 | 0.000539891 |
| *LOC_Os11g15180.1* | 1098 | 95 | 30 | 4.676220521 | 1.625168696 | -1.524753485 | 6.50E-08 | 1.25E-06 |
| *LOC_Os08g01510.1* | 1455 | 60 | 19 | 2.228753177 | 0.776730111 | -1.520751555 | 1.94E-05 | 0.000238215 |
| *LOC_Os12g43410.1* | 453 | 192 | 61 | 22.90744988 | 8.009606248 | -1.516013636 | 1.65E-14 | 6.22E-13 |
| *LOC_Os05g49060.1* | 894 | 415 | 132 | 25.08905457 | 8.782455261 | -1.51436188 | 1.41E-29 | 1.17E-27 |
| *LOC_Os04g30030.1* | 1392 | 757 | 241 | 29.3920828 | 10.2981056 | -1.513048626 | 1.57E-52 | 2.38E-50 |
| *ChrSy.fgenesh.mRNA.28* | 1674 | 269 | 86 | 8.685014435 | 3.055783146 | -1.506986081 | 1.39E-19 | 7.29E-18 |
| *LOC_Os01g02750.1* | 1212 | 75 | 24 | 3.344508945 | 1.177845035 | -1.505644662 | 2.03E-06 | 3.06E-05 |
| *LOC_Os05g06920.1* | 1680 | 184 | 59 | 5.919462308 | 2.088922192 | -1.502707379 | 8.40E-14 | 2.97E-12 |
| *LOC_Os06g48250.1* | 1515 | 193 | 62 | 6.885229081 | 2.434213072 | -1.5000492 | 2.25E-14 | 8.31E-13 |
| *LOC_Os04g26960.1* | 1554 | 84 | 27 | 2.921473759 | 1.033456696 | -1.499218393 | 5.30E-07 | 8.80E-06 |
| *LOC_Os05g27100.1* | 495 | 115 | 37 | 12.5564352 | 4.446067572 | -1.497825158 | 4.22E-09 | 9.61E-08 |
| *LOC_Os01g66830.1* | 1242 | 528 | 170 | 22.97661488 | 8.141545592 | -1.496791656 | 1.38E-36 | 1.50E-34 |
| *LOC_Os12g35590.1* | 327 | 118 | 38 | 19.50329424 | 6.912185389 | -1.496504009 | 2.71E-09 | 6.30E-08 |
| *LOC_Os02g16800.1* | 837 | 93 | 30 | 6.005251617 | 2.13194173 | -1.494056688 | 1.38E-07 | 2.54E-06 |
| *LOC_Os08g41910.1* | 942 | 151 | 49 | 8.663627332 | 3.094031358 | -1.485483368 | 2.09E-11 | 6.03E-10 |
| *LOC_Os03g53710.1* | 1236 | 104 | 34 | 4.547666273 | 1.636213532 | -1.47476535 | 3.38E-08 | 6.76E-07 |
| *LOC_Os01g03340.1* | 756 | 2936 | 961 | 209.8978422 | 75.61032867 | -1.473032105 | 7.70E-191 | 5.01E-188 |
| *LOC_Os08g18894.1* | 498 | 61 | 20 | 6.620247264 | 2.388802179 | -1.470597715 | 2.64E-05 | 0.000314156 |
| *LOC_Os02g31030.2* | 1167 | 76 | 25 | 3.51978758 | 1.274232525 | -1.465859796 | 2.75E-06 | 4.05E-05 |
| *LOC_Os03g01820.1* | 903 | 76 | 25 | 4.548828467 | 1.646765622 | -1.465859796 | 2.75E-06 | 4.05E-05 |
| *LOC_Os02g38820.1* | 797 | 233 | 77 | 15.80051774 | 5.746612821 | -1.459188077 | 1.97E-16 | 8.53E-15 |
| *LOC_Os03g04060.1* | 771 | 157 | 52 | 11.00573351 | 4.011700469 | -1.455969503 | 1.68E-11 | 4.89E-10 |
| *LOC_Os05g08750.1* | 1584 | 87 | 29 | 2.96850506 | 1.088986145 | -1.446750973 | 6.62E-07 | 1.08E-05 |
| *LOC_Os03g34040.1* | 981 | 78 | 26 | 4.297336019 | 1.576463334 | -1.446750973 | 2.55E-06 | 3.77E-05 |
| *LOC_Os05g15690.1* | 795 | 81 | 27 | 5.506702426 | 2.020115352 | -1.446750973 | 1.62E-06 | 2.48E-05 |
| *LOC_Os12g42044.3* | 402 | 54 | 18 | 7.260080313 | 2.663336161 | -1.446750973 | 9.57E-05 | 0.000996962 |
| *LOC_Os12g38750.2* | 1824 | 54 | 18 | 1.60008349 | 0.586985272 | -1.446750973 | 9.57E-05 | 0.000997263 |
| *LOC_Os05g34630.1* | 1212 | 78 | 26 | 3.478289303 | 1.275998788 | -1.446750973 | 2.55E-06 | 3.77E-05 |
| *LOC_Os05g46460.3* | 1134 | 102 | 34 | 4.861394166 | 1.783386177 | -1.446750973 | 7.08E-08 | 1.35E-06 |
| *LOC_Os08g26840.1* | 1641 | 140 | 47 | 4.610979303 | 1.703604626 | -1.436482638 | 3.18E-10 | 8.16E-09 |
| *LOC_Os09g26670.1* | 696 | 101 | 34 | 7.843065689 | 2.905689548 | -1.432537114 | 1.02E-07 | 1.90E-06 |
| *LOC_Os06g39330.1* | 1410 | 101 | 34 | 3.871470723 | 1.434297819 | -1.432537114 | 1.02E-07 | 1.91E-06 |
| *LOC_Os05g20660.1* | 504 | 92 | 31 | 9.865770513 | 3.65856429 | -1.431154118 | 3.90E-07 | 6.63E-06 |
| *LOC_Os06g40150.1* | 732 | 59 | 20 | 4.356268591 | 1.625168696 | -1.422503427 | 5.57E-05 | 0.000617088 |
| *LOC_Os04g44150.1* | 1077 | 109 | 37 | 5.469964564 | 2.04345724 | -1.420519432 | 3.86E-08 | 7.68E-07 |
| *LOC_Os12g38010.1* | 237 | 5073 | 1725 | 1156.885118 | 432.9325975 | -1.418031271 | 0 | 0 |
| *LOC_Os03g48030.1* | 753 | 126 | 43 | 9.043765383 | 3.396667322 | -1.412803642 | 3.85E-09 | 8.82E-08 |
| *LOC_Os01g73700.1* | 975 | 492 | 168 | 27.27308119 | 10.24906387 | -1.411985555 | 1.71E-31 | 1.55E-29 |
| *LOC_Os04g47930.1* | 1542 | 152 | 52 | 5.32761622 | 2.005850235 | -1.409276268 | 1.03E-10 | 2.78E-09 |
| *LOC_Os07g06450.1* | 1047 | 76 | 26 | 3.923201629 | 1.477087422 | -1.409276268 | 5.30E-06 | 7.38E-05 |
| *LOC_Os02g03720.1* | 924 | 73 | 25 | 4.26996787 | 1.609339131 | -1.407756842 | 8.33E-06 | 0.000110362 |
| *LOC_Os04g27060.1* | 1068 | 70 | 24 | 3.542423706 | 1.336655602 | -1.406108989 | 1.31E-05 | 0.000166716 |
| *LOC_Os07g04730.1* | 2088 | 64 | 22 | 1.656621136 | 0.626717353 | -1.402356854 | 3.24E-05 | 0.000376973 |
| *LOC_Os01g59020.1* | 1536 | 450 | 155 | 15.83415954 | 6.002332039 | -1.399445259 | 1.41E-28 | 1.14E-26 |
| *LOC_Os02g39930.1* | 588 | 2493 | 859 | 229.1493716 | 86.89511683 | -1.398941319 | 2.06E-150 | 1.13E-147 |
| *LOC_Os08g41090.1* | 996 | 87 | 30 | 4.720996 | 1.791601635 | -1.397841373 | 1.26E-06 | 1.97E-05 |
| *LOC_Os10g37330.1* | 1128 | 113 | 39 | 5.414309303 | 2.056529961 | -1.396565216 | 3.26E-08 | 6.54E-07 |
| *LOC_Os08g31670.1* | 1527 | 223 | 77 | 7.8929535 | 2.999378139 | -1.395901832 | 7.18E-15 | 2.77E-13 |
| *LOC_Os02g30690.1* | 978 | 130 | 45 | 7.184196719 | 2.736863847 | -1.392303189 | 3.25E-09 | 7.51E-08 |
| *LOC_Os10g37880.1* | 927 | 78 | 27 | 4.547666273 | 1.732461386 | -1.392303189 | 4.86E-06 | 6.81E-05 |
| *LOC_Os06g02580.1* | 1104 | 98 | 34 | 4.797673846 | 1.831847758 | -1.389035476 | 3.02E-07 | 5.25E-06 |
| *LOC_Os07g43810.1* | 795 | 1320 | 458 | 89.73885435 | 34.26714191 | -1.388906899 | 1.13E-79 | 2.82E-77 |
| *LOC_Os03g28160.2* | 606 | 69 | 24 | 6.153896459 | 2.35569007 | -1.385350429 | 1.88E-05 | 0.00023122 |
| *LOC_Os08g09010.1* | 678 | 6180 | 2151 | 492.6432078 | 188.7079732 | -1.384387786 | 0 | 0 |
| *LOC_Os01g54515.1* | 1587 | 336 | 117 | 11.44289911 | 4.385190541 | -1.383741176 | 2.17E-21 | 1.27E-19 |
| *LOC_Os02g21300.1* | 1020 | 215 | 75 | 11.39231557 | 4.373615755 | -1.381162632 | 3.50E-14 | 1.27E-12 |
| *LOC_Os01g68650.1* | 882 | 455 | 159 | 27.88152536 | 10.72279672 | -1.378628253 | 2.89E-28 | 2.30E-26 |
| *LOC_Os06g48020.1* | 960 | 60 | 21 | 3.377954034 | 1.301150687 | -1.376361646 | 7.33E-05 | 0.000788041 |
| *LOC_Os07g35560.2* | 1677 | 97 | 34 | 3.126168552 | 1.205939132 | -1.374238474 | 4.32E-07 | 7.28E-06 |
| *LOC_Os01g47350.1* | 552 | 77 | 27 | 7.539201758 | 2.909405263 | -1.373687511 | 6.96E-06 | 9.46E-05 |
| *LOC_Os11g18110.1* | 1710 | 94 | 33 | 2.971019221 | 1.14788231 | -1.371983205 | 6.75E-07 | 1.10E-05 |
| *LOC_Os10g39680.1* | 786 | 1714 | 603 | 117.8587932 | 45.63250392 | -1.368925675 | 1.04E-100 | 3.67E-98 |
| *LOC_Os07g01540.1* | 3051 | 602 | 212 | 10.66419314 | 4.133074056 | -1.367487695 | 1.92E-36 | 2.07E-34 |
| *LOC_Os08g43190.2* | 1110 | 88 | 31 | 4.28482818 | 1.661185948 | -1.367023781 | 1.65E-06 | 2.52E-05 |
| *LOC_Os01g20160.1* | 1665 | 264 | 93 | 8.569656361 | 3.322371896 | -1.367023781 | 7.64E-17 | 3.40E-15 |
| *LOC_Os12g22284.1* | 2142 | 139 | 49 | 3.507268801 | 1.360680457 | -1.366019701 | 1.60E-09 | 3.82E-08 |
| *LOC_Os07g03730.1* | 498 | 85 | 30 | 9.224934712 | 3.583203269 | -1.364288813 | 2.58E-06 | 3.82E-05 |
| *LOC_Os01g10400.1* | 318 | 68 | 24 | 11.5572767 | 4.489145228 | -1.364288813 | 2.69E-05 | 0.00031978 |
| *LOC_Os12g08760.1* | 1071 | 1758 | 622 | 88.71623817 | 34.54462222 | -1.360737058 | 2.53E-102 | 9.28E-100 |
| *LOC_Os01g09540.1* | 912 | 65 | 23 | 3.852052846 | 1.500073474 | -1.36059433 | 4.23E-05 | 0.000480365 |
| *LOC_Os08g42690.1* | 996 | 79 | 28 | 4.286881425 | 1.672161526 | -1.358214299 | 6.34E-06 | 8.69E-05 |
| *LOC_Os10g01134.1* | 1419 | 76 | 27 | 2.894709024 | 1.1317771 | -1.354828484 | 9.93E-06 | 0.000129434 |
| *LOC_Os01g02940.6* | 2016 | 242 | 86 | 6.487816479 | 2.537391363 | -1.354386955 | 2.30E-15 | 9.16E-14 |
| *LOC_Os08g37840.1* | 996 | 298 | 106 | 16.17076791 | 6.330325775 | -1.353036539 | 1.49E-18 | 7.31E-17 |
| *LOC_Os04g39380.1* | 399 | 73 | 26 | 9.888346647 | 3.875966243 | -1.351173313 | 1.56E-05 | 0.000195731 |
| *LOC_Os01g43320.1* | 1365 | 129 | 46 | 5.107763463 | 2.004493785 | -1.349453772 | 8.55E-09 | 1.86E-07 |
| *LOC_Os07g22510.1* | 789 | 274 | 98 | 18.76926551 | 7.38802925 | -1.345110712 | 4.87E-17 | 2.21E-15 |
| *LOC_Os10g39390.1* | 1218 | 223 | 80 | 9.895353033 | 3.906809476 | -1.340760278 | 4.48E-14 | 1.62E-12 |
| *LOC_Os08g10760.1* | 660 | 331 | 119 | 27.10552207 | 10.72463597 | -1.337658116 | 4.10E-20 | 2.22E-18 |
| *LOC_Os11g15340.2* | 594 | 377 | 136 | 34.30272514 | 13.61858535 | -1.332746345 | 1.41E-22 | 8.68E-21 |
| *LOC_Os05g02900.1* | 663 | 72 | 26 | 5.869386195 | 2.332595069 | -1.331273756 | 2.22E-05 | 0.000268022 |
| *LOC_Os03g55590.1* | 1047 | 725 | 262 | 37.4252787 | 14.88449633 | -1.330202656 | 8.05E-42 | 9.85E-40 |
| *LOC_Os02g28900.1* | 1485 | 315 | 114 | 11.46457127 | 4.56623156 | -1.328106477 | 5.07E-19 | 2.56E-17 |
| *LOC_Os01g71420.1* | 1149 | 569 | 206 | 26.76492039 | 10.66416179 | -1.327572788 | 4.41E-33 | 4.28E-31 |
| *LOC_Os04g30540.1* | 1092 | 69 | 25 | 3.415074408 | 1.361748495 | -1.32645674 | 3.48E-05 | 0.000401786 |
| *LOC_Os11g07840.1* | 1386 | 149 | 54 | 5.810275915 | 2.317448348 | -1.326069491 | 1.00E-09 | 2.46E-08 |
| *LOC_Os12g09620.1* | 504 | 322 | 117 | 34.5301968 | 13.80812974 | -1.322340631 | 2.72E-19 | 1.39E-17 |
| *LOC_Os09g30360.1* | 777 | 74 | 27 | 5.147358528 | 2.066913392 | -1.316354336 | 2.01E-05 | 0.000245411 |
| *LOC_Os04g46990.1* | 1392 | 167 | 61 | 6.484118664 | 2.606574447 | -1.314755428 | 1.30E-10 | 3.48E-09 |
| *LOC_Os11g24630.1* | 666 | 246 | 90 | 19.96340402 | 8.037996523 | -1.312449882 | 6.24E-15 | 2.42E-13 |
| *LOC_Os04g15650.1* | 2229 | 71 | 26 | 1.721559346 | 0.693813607 | -1.311095874 | 3.15E-05 | 0.000367439 |
| *LOC_Os03g03020.1* | 633 | 161 | 59 | 13.74661863 | 5.544058897 | -1.310062301 | 3.12E-10 | 8.03E-09 |
| *LOC_Os08g06170.1* | 1590 | 106 | 39 | 3.60315097 | 1.458972199 | -1.304306708 | 3.79E-07 | 6.45E-06 |
| *LOC_Os03g55260.1* | 1557 | 440 | 162 | 15.27347232 | 6.188792698 | -1.303298183 | 2.90E-25 | 2.01E-23 |
| *LOC_Os03g49720.1* | 789 | 76 | 28 | 5.206073645 | 2.1108655 | -1.302361064 | 1.81E-05 | 0.000223687 |
| *LOC_Os03g29930.1* | 861 | 95 | 35 | 5.963403173 | 2.417933913 | -1.302361064 | 1.59E-06 | 2.43E-05 |
| *LOC_Os01g57480.1* | 2484 | 443 | 164 | 9.638864008 | 3.927098462 | -1.295399357 | 3.34E-25 | 2.30E-23 |
| *LOC_Os03g62510.1* | 1794 | 81 | 30 | 2.440261108 | 0.994668466 | -1.29474788 | 1.04E-05 | 0.000135761 |
| *LOC_Os07g26100.1* | 552 | 116 | 43 | 11.35775849 | 4.633497271 | -1.293504713 | 1.28E-07 | 2.35E-06 |
| *LOC_Os03g41330.1* | 699 | 151 | 56 | 11.67544628 | 4.765301515 | -1.29283829 | 1.63E-09 | 3.88E-08 |
| *LOC_Os04g35660.1* | 603 | 140 | 52 | 12.54828696 | 5.129388162 | -1.290631772 | 6.72E-09 | 1.49E-07 |
| *LOC_Os04g45810.1* | 831 | 78 | 29 | 5.073028441 | 2.075756984 | -1.289209696 | 1.63E-05 | 0.000204226 |
| *LOC_Os02g26890.1* | 1521 | 164 | 61 | 5.82758145 | 2.38550403 | -1.28860314 | 3.58E-10 | 9.19E-09 |
| *LOC_Os06g38490.1* | 2901 | 86 | 32 | 1.602228456 | 0.656117744 | -1.288053227 | 6.03E-06 | 8.30E-05 |
| *LOC_Os04g45490.1* | 2055 | 583 | 217 | 15.3331169 | 6.280980446 | -1.287589313 | 2.25E-32 | 2.14E-30 |
| *LOC_Os11g03290.1* | 1374 | 1866 | 695 | 73.40043351 | 30.08691129 | -1.286652576 | 1.04E-99 | 3.60E-97 |
| *LOC_Os02g33070.1* | 297 | 228 | 85 | 41.49082935 | 17.02323169 | -1.285287551 | 1.51E-13 | 5.23E-12 |
| *LOC_Os07g07540.1* | 1008 | 217 | 81 | 11.63517501 | 4.779737218 | -1.283489702 | 6.13E-13 | 2.03E-11 |
| *LOC_Os11g05260.1* | 1488 | 198 | 74 | 7.191773105 | 2.95806915 | -1.281691727 | 6.60E-12 | 1.98E-10 |
| *LOC_Os03g14400.1* | 1506 | 155 | 58 | 5.56263347 | 2.290775636 | -1.279931883 | 1.33E-09 | 3.20E-08 |
| *LOC_Os11g05360.1* | 1059 | 267 | 100 | 13.62664744 | 5.616730337 | -1.278628215 | 1.66E-15 | 6.71E-14 |
| *LOC_Os02g17060.1* | 1707 | 128 | 48 | 4.052753288 | 1.67258135 | -1.276825972 | 3.89E-08 | 7.74E-07 |
| *LOC_Os04g43420.1* | 1104 | 128 | 48 | 6.266349513 | 2.586138012 | -1.276825972 | 3.89E-08 | 7.74E-07 |
| *LOC_Os01g03890.1* | 924 | 296 | 111 | 17.31384232 | 7.14546574 | -1.276825972 | 5.37E-17 | 2.42E-15 |
| *LOC_Os07g36390.1* | 1938 | 93 | 35 | 2.593599382 | 1.074221414 | -1.271664267 | 3.13E-06 | 4.55E-05 |
| *LOC_Os01g43851.1* | 1587 | 85 | 32 | 2.894781025 | 1.199368353 | -1.271179409 | 8.47E-06 | 0.000112065 |
| *LOC_Os03g61220.1* | 2277 | 77 | 29 | 1.827685275 | 0.757555579 | -1.270594018 | 2.30E-05 | 0.000277079 |
| *LOC_Os01g16870.1* | 2715 | 90 | 34 | 1.791622029 | 0.74488395 | -1.266178728 | 4.88E-06 | 6.84E-05 |
| *LOC_Os05g47510.1* | 1323 | 82 | 31 | 3.349868249 | 1.393738777 | -1.265144167 | 1.32E-05 | 0.000168189 |
| *LOC_Os07g40120.1* | 2355 | 74 | 28 | 1.698300457 | 0.707207168 | -1.263886916 | 3.59E-05 | 0.000413282 |
| *LOC_Os11g34610.1* | 1005 | 95 | 36 | 5.108945405 | 2.130668929 | -1.26171908 | 2.82E-06 | 4.13E-05 |
| *LOC_Os11g04290.1* | 1629 | 87 | 33 | 2.886502158 | 1.204959331 | -1.260337849 | 7.61E-06 | 0.000102003 |
| *LOC_Os12g05440.1* | 1530 | 87 | 33 | 3.073275827 | 1.282927288 | -1.260337849 | 7.61E-06 | 0.000101963 |
| *LOC_Os02g37380.1* | 285 | 630 | 239 | 119.4729006 | 49.88070404 | -1.260129683 | 9.25E-34 | 9.23E-32 |
| *LOC_Os04g11030.1* | 246 | 282 | 107 | 61.95662034 | 25.87189287 | -1.259872839 | 5.85E-16 | 2.44E-14 |
| *LOC_Os06g43430.1* | 1563 | 195 | 74 | 6.742940875 | 2.816127253 | -1.259665421 | 1.79E-11 | 5.19E-10 |
| *LOC_Os03g62060.1* | 1254 | 4534 | 1724 | 195.4149103 | 81.77475633 | -1.256813089 | 5.73E-231 | 4.82E-228 |
| *LOC_Os09g20024.1* | 201 | 71 | 27 | 19.0913223 | 7.990008484 | -1.25664809 | 5.62E-05 | 0.00062218 |
| *LOC_Os04g32150.1* | 744 | 84 | 32 | 6.102110514 | 2.558330076 | -1.254105895 | 1.19E-05 | 0.000152631 |
| *LOC_Os01g20110.1* | 1338 | 307 | 117 | 12.40097924 | 5.201268602 | -1.253518598 | 4.04E-17 | 1.84E-15 |
| *LOC_Os04g59200.1* | 1059 | 774 | 295 | 39.50196672 | 16.56935449 | -1.253407085 | 9.04E-41 | 1.07E-38 |
| *LOC_Os05g39000.1* | 2610 | 76 | 29 | 1.573790079 | 0.660901936 | -1.251734991 | 3.22E-05 | 0.000375706 |
| *LOC_Os03g63620.1* | 858 | 149 | 57 | 9.385830324 | 3.951546542 | -1.248066979 | 5.59E-09 | 1.25E-07 |
| *LOC_Os07g38000.1* | 567 | 86 | 33 | 8.197645064 | 3.461867285 | -1.243659108 | 1.06E-05 | 0.000138001 |
| *LOC_Os04g08390.1* | 3045 | 138 | 53 | 2.449432679 | 1.035304511 | -1.242392475 | 2.30E-08 | 4.71E-07 |
| *LOC_Os12g36730.1* | 2205 | 1278 | 493 | 31.32535333 | 13.29896549 | -1.236016757 | 8.42E-65 | 1.65E-62 |
| *LOC_Os08g39860.1* | 1500 | 93 | 36 | 3.350930402 | 1.427548182 | -1.231022282 | 5.49E-06 | 7.61E-05 |
| *LOC_Os06g43800.1* | 1038 | 124 | 48 | 6.456513299 | 2.750574533 | -1.231022282 | 1.47E-07 | 2.69E-06 |
| *LOC_Os04g50870.1* | 1971 | 93 | 36 | 2.550175344 | 1.086414142 | -1.231022282 | 5.49E-06 | 7.61E-05 |
| *LOC_Os08g34984.1* | 576 | 1612 | 624 | 151.2572751 | 64.43793879 | -1.231022282 | 9.22E-81 | 2.37E-78 |
| *LOC_Os10g38660.1* | 705 | 85 | 33 | 6.516336861 | 2.784225178 | -1.226785289 | 1.48E-05 | 0.000187103 |
| *LOC_Os01g41565.1* | 1122 | 954 | 371 | 45.95462601 | 19.66801752 | -1.224358552 | 4.02E-48 | 5.54E-46 |
| *LOC_Os12g36210.1* | 234 | 513 | 200 | 118.4882338 | 50.83861049 | -1.220747299 | 1.60E-26 | 1.18E-24 |
| *LOC_Os12g36060.1* | 354 | 82 | 32 | 12.51942286 | 5.376829312 | -1.219340477 | 2.31E-05 | 0.000278204 |
| *LOC_Os08g02700.1* | 1089 | 156 | 61 | 7.742307869 | 3.331819679 | -1.216453354 | 5.03E-09 | 1.13E-07 |
| *LOC_Os01g74600.1* | 2442 | 115 | 45 | 2.545223351 | 1.096090435 | -1.215425427 | 5.45E-07 | 9.03E-06 |
| *LOC_Os04g25800.1* | 1524 | 97 | 38 | 3.440016182 | 1.483126393 | -1.213773801 | 4.39E-06 | 6.22E-05 |
| *LOC_Os01g17150.1* | 432 | 209 | 82 | 26.14786641 | 11.29040808 | -1.2115956 | 1.46E-11 | 4.28E-10 |
| *LOC_Os10g30790.1* | 1626 | 158 | 62 | 5.251825214 | 2.268039855 | -1.211372911 | 4.50E-09 | 1.02E-07 |
| *LOC_Os03g59030.1* | 1452 | 456 | 179 | 16.9735211 | 7.332734293 | -1.21086271 | 1.81E-23 | 1.16E-21 |
| *LOC_Os10g39750.1* | 1143 | 163 | 64 | 7.707527665 | 3.330529443 | -1.210516627 | 2.62E-09 | 6.10E-08 |
| *LOC_Os07g08160.1* | 579 | 145 | 57 | 13.53515261 | 5.855659643 | -1.208807549 | 2.06E-08 | 4.26E-07 |
| *LOC_Os10g31780.1* | 786 | 638 | 251 | 43.87042593 | 18.99462435 | -1.207657533 | 4.50E-32 | 4.24E-30 |
| *LOC_Os02g54900.1* | 1917 | 127 | 50 | 3.580596034 | 1.551412996 | -1.20661697 | 1.63E-07 | 2.96E-06 |
| *LOC_Os01g37950.1* | 213 | 226 | 89 | 57.34592389 | 24.8536362 | -1.206234004 | 2.60E-12 | 8.13E-11 |
| *LOC_Os01g42350.1* | 4440 | 312 | 123 | 3.797915887 | 1.647789287 | -1.204676186 | 2.04E-16 | 8.81E-15 |
| *LOC_Os01g28089.1* | 1698 | 76 | 30 | 2.419076623 | 1.050904139 | -1.202825391 | 5.60E-05 | 0.000619928 |
| *LOC_Os03g24730.1* | 2559 | 233 | 92 | 4.921067855 | 2.138440028 | -1.202412661 | 1.36E-12 | 4.36E-11 |
| *LOC_Os07g34710.1* | 1020 | 162 | 64 | 8.583977311 | 3.732152111 | -1.201638476 | 3.61E-09 | 8.29E-08 |
| *LOC_Os04g52210.1* | 2220 | 101 | 40 | 2.458907081 | 1.07173287 | -1.198071861 | 3.50E-06 | 5.04E-05 |
| *LOC_Os09g15240.1* | 1647 | 328 | 130 | 10.76351109 | 4.694931788 | -1.196972664 | 5.03E-17 | 2.27E-15 |
| *LOC_Os12g38300.1* | 240 | 54141 | 21492 | 12192.38729 | 5326.539156 | -1.194710264 | 0 | 0 |
| *LOC_Os05g40400.1* | 636 | 224 | 89 | 19.03551456 | 8.323623443 | -1.193409964 | 4.91E-12 | 1.49E-10 |
| *LOC_Os02g39740.1* | 1179 | 78 | 31 | 3.575646001 | 1.563966414 | -1.192994381 | 4.99E-05 | 0.000556553 |
| *LOC_Os12g38051.1* | 240 | 40479 | 16096 | 9115.746757 | 3989.204088 | -1.192259912 | 0 | 0 |
| *LOC_Os08g04310.1* | 525 | 294 | 117 | 30.26646815 | 13.25580455 | -1.191096098 | 2.64E-15 | 1.05E-13 |
| *LOC_Os09g10760.1* | 738 | 339 | 135 | 24.826589 | 10.88070261 | -1.190114339 | 2.12E-17 | 9.81E-16 |
| *LOC_Os12g17600.1* | 528 | 133 | 53 | 13.61417838 | 5.970648175 | -1.189150454 | 1.16E-07 | 2.15E-06 |
| *LOC_Os06g38760.1* | 3615 | 158 | 63 | 2.362231756 | 1.036601377 | -1.188289297 | 7.64E-09 | 1.68E-07 |
| *LOC_Os12g34980.1* | 1017 | 654 | 261 | 34.75605803 | 15.26508012 | -1.187029302 | 5.33E-32 | 4.96E-30 |
| *LOC_Os01g59060.1* | 504 | 110 | 44 | 11.79602996 | 5.192800928 | -1.183716568 | 1.61E-06 | 2.46E-05 |
| *LOC_Os09g24590.1* | 252 | 115 | 46 | 24.66442628 | 10.85767467 | -1.183716568 | 9.28E-07 | 1.48E-05 |
| *LOC_Os02g37580.1* | 2022 | 165 | 66 | 4.410385089 | 1.941522009 | -1.183716568 | 3.97E-09 | 9.07E-08 |
| *LOC_Os01g51530.1* | 924 | 314 | 126 | 18.36671111 | 8.111069218 | -1.179129298 | 5.17E-16 | 2.17E-14 |
| *LOC_Os10g42130.1* | 990 | 304 | 122 | 16.59633174 | 7.330003294 | -1.178978649 | 1.51E-15 | 6.13E-14 |
| *LOC_Os11g47640.1* | 1044 | 97 | 39 | 5.021632817 | 2.221997889 | -1.176299096 | 7.48E-06 | 0.000100416 |
| *LOC_Os12g36240.1* | 258 | 293 | 118 | 61.3792578 | 27.20456808 | -1.173902278 | 6.04E-15 | 2.35E-13 |
| *LOC_Os09g36040.1* | 690 | 196 | 79 | 15.35255631 | 6.810163431 | -1.172717569 | 1.88E-10 | 4.93E-09 |
| *LOC_Os10g05250.1* | 1953 | 129 | 52 | 3.569942205 | 1.583728142 | -1.17257601 | 2.47E-07 | 4.34E-06 |
| *LOC_Os06g29790.1* | 2583 | 186 | 75 | 3.891905229 | 1.727095652 | -1.172128593 | 5.55E-10 | 1.39E-08 |
| *LOC_Os07g02800.2* | 1068 | 166 | 67 | 8.400604789 | 3.731496888 | -1.170738714 | 4.85E-09 | 1.09E-07 |
| *LOC_Os12g07830.1* | 963 | 84 | 34 | 4.714403138 | 2.100062228 | -1.166643054 | 3.51E-05 | 0.000404836 |
| *LOC_Os01g48820.1* | 906 | 79 | 32 | 4.712730573 | 2.100880327 | -1.165569221 | 6.12E-05 | 0.000670676 |
| *LOC_Os06g10870.1* | 858 | 421 | 171 | 26.51969508 | 11.85463963 | -1.161612381 | 1.68E-20 | 9.28E-19 |
| *LOC_Os10g04450.1* | 1194 | 677 | 275 | 30.64488953 | 13.69960044 | -1.161512688 | 5.29E-32 | 4.93E-30 |
| *LOC_Os11g45990.1* | 1902 | 593 | 242 | 16.85069815 | 7.568056873 | -1.15481353 | 5.46E-28 | 4.29E-26 |
| *LOC_Os06g18670.1* | 1446 | 196 | 80 | 7.325908611 | 3.290798023 | -1.154570222 | 3.13E-10 | 8.05E-09 |
| *LOC_Os01g02300.1* | 1980 | 142 | 58 | 3.876116953 | 1.742377832 | -1.153554597 | 8.98E-08 | 1.69E-06 |
| *LOC_Os09g28420.1* | 1314 | 88 | 36 | 3.619603714 | 1.629621213 | -1.15129509 | 2.77E-05 | 0.00032786 |
| *LOC_Os01g73730.1* | 1026 | 454 | 186 | 23.91565117 | 10.78313686 | -1.149178149 | 1.22E-21 | 7.24E-20 |
| *LOC_Os01g52260.1* | 912 | 261 | 107 | 15.46747374 | 6.978602683 | -1.148227483 | 4.68E-13 | 1.56E-11 |
| *LOC_Os03g52460.1* | 1536 | 217 | 89 | 7.635583598 | 3.446500332 | -1.147606274 | 4.38E-11 | 1.22E-09 |
| *LOC_Os03g04210.1* | 912 | 95 | 39 | 5.629923391 | 2.543602847 | -1.146241862 | 1.41E-05 | 0.000178459 |
| *LOC_Os10g05069.3* | 2448 | 146 | 60 | 3.223407118 | 1.457871918 | -1.144722436 | 7.11E-08 | 1.36E-06 |
| *LOC_Os03g17580.1* | 663 | 102 | 42 | 8.314963777 | 3.768038189 | -1.141896392 | 7.21E-06 | 9.72E-05 |
| *LOC_Os04g41960.1* | 1038 | 148 | 61 | 7.706161034 | 3.495521802 | -1.140504501 | 6.32E-08 | 1.22E-06 |
| *LOC_Os09g13400.1* | 954 | 786 | 324 | 44.52950727 | 20.20115352 | -1.140323972 | 7.17E-36 | 7.66E-34 |
| *LOC_Os11g15300.1* | 1167 | 327 | 135 | 15.14434919 | 6.880855635 | -1.138119701 | 8.69E-16 | 3.57E-14 |
| *LOC_Os09g23570.1* | 1947 | 104 | 43 | 2.886962256 | 1.313657162 | -1.135963436 | 6.39E-06 | 8.76E-05 |
| *LOC_Os07g34900.1* | 1368 | 746 | 309 | 29.4731428 | 13.43544068 | -1.133357265 | 8.19E-34 | 8.22E-32 |
| *LOC_Os05g50700.1* | 990 | 82 | 34 | 4.476642114 | 2.042787803 | -1.131877636 | 6.61E-05 | 0.00071603 |
| *LOC_Os01g43070.1* | 948 | 94 | 39 | 5.359116949 | 2.447010334 | -1.130975106 | 1.93E-05 | 0.00023684 |
| *LOC_Os04g41340.1* | 1098 | 147 | 61 | 7.235835964 | 3.304509682 | -1.13072348 | 8.60E-08 | 1.63E-06 |
| *LOC_Os07g06900.1* | 1548 | 89 | 37 | 3.107368569 | 1.421707654 | -1.128068538 | 3.36E-05 | 0.000389676 |
| *LOC_Os03g18779.1* | 714 | 101 | 42 | 7.645341344 | 3.498892604 | -1.127682533 | 9.84E-06 | 0.000128287 |
| *LOC_Os02g02780.1* | 1752 | 281 | 117 | 8.668539577 | 3.972201706 | -1.125850073 | 1.47E-13 | 5.11E-12 |
| *LOC_Os01g14370.2* | 3831 | 120 | 50 | 1.692944857 | 0.776313942 | -1.124822879 | 1.49E-06 | 2.29E-05 |
| *LOC_Os12g06190.1* | 489 | 84 | 35 | 9.284192683 | 4.257343762 | -1.124822879 | 5.85E-05 | 0.000644757 |
| *LOC_Os10g40600.1* | 1791 | 403 | 168 | 12.16138895 | 5.579473633 | -1.124107078 | 9.18E-19 | 4.60E-17 |
| *LOC_Os12g41510.1* | 1971 | 187 | 78 | 5.127771928 | 2.353897307 | -1.123280714 | 1.84E-09 | 4.34E-08 |
| *LOC_Os10g25000.1* | 666 | 170 | 71 | 13.79584831 | 6.341086146 | -1.121432289 | 1.05E-08 | 2.26E-07 |
| *LOC_Os02g57670.1* | 564 | 122 | 51 | 11.69107496 | 5.378616822 | -1.120100468 | 1.32E-06 | 2.04E-05 |
| *LOC_Os06g17410.1* | 870 | 98 | 41 | 6.088082673 | 2.803135799 | -1.118946312 | 1.51E-05 | 0.000190439 |
| *LOC_Os04g29210.1* | 1773 | 141 | 59 | 4.298175015 | 1.979350977 | -1.118696776 | 2.01E-07 | 3.60E-06 |
| *LOC_Os10g02880.1* | 1101 | 903 | 378 | 44.327593 | 20.42132959 | -1.118128226 | 9.61E-40 | 1.12E-37 |
| *LOC_Os10g04800.1* | 156 | 2383 | 998 | 825.6066117 | 380.5269995 | -1.117455704 | 7.01E-102 | 2.54E-99 |
| *LOC_Os11g24140.1* | 375 | 8687 | 3646 | 1252.022899 | 578.3156303 | -1.114331949 | 0 | 0 |
| *LOC_Os01g65830.1* | 1146 | 257 | 108 | 12.12054711 | 5.60555569 | -1.11252552 | 2.58E-12 | 8.08E-11 |
| *LOC_Os07g03810.1* | 2046 | 354 | 149 | 9.351286242 | 4.33171797 | -1.110225502 | 2.30E-16 | 9.85E-15 |
| *LOC_Os10g05660.1* | 978 | 178 | 75 | 9.8368232 | 4.561439744 | -1.108703213 | 6.51E-09 | 1.44E-07 |
| *LOC_Os08g25700.1* | 1074 | 185 | 78 | 9.30981745 | 4.319861818 | -1.107767714 | 3.36E-09 | 7.76E-08 |
| *LOC_Os09g30130.1* | 2187 | 372 | 157 | 9.193224697 | 4.270024856 | -1.106326535 | 4.93E-17 | 2.23E-15 |
| *LOC_Os04g13140.1* | 1050 | 1820 | 770 | 93.68192522 | 43.6195278 | -1.102796572 | 1.21E-76 | 2.76E-74 |
| *LOC_Os08g09080.2* | 675 | 451 | 191 | 36.11157972 | 16.83096931 | -1.101343268 | 3.41E-20 | 1.85E-18 |
| *LOC_Os07g44920.1* | 282 | 621 | 263 | 119.0189762 | 55.47357742 | -1.101318942 | 3.26E-27 | 2.47E-25 |
| *LOC_Os01g36640.1* | 2718 | 595 | 252 | 11.83153878 | 5.514810859 | -1.101254407 | 3.87E-26 | 2.80E-24 |
| *LOC_Os05g49370.1* | 765 | 432 | 183 | 30.52080822 | 14.22882992 | -1.100976137 | 2.13E-19 | 1.10E-17 |
| *LOC_Os05g02390.1* | 597 | 361 | 153 | 32.68184674 | 15.24391903 | -1.100255657 | 1.93E-16 | 8.35E-15 |
| *LOC_Os06g51050.1* | 963 | 243 | 103 | 13.63809479 | 6.361953219 | -1.100100449 | 1.55E-11 | 4.52E-10 |
| *LOC_Os01g11830.1* | 1050 | 1014 | 430 | 52.19421548 | 24.35895708 | -1.09943756 | 2.81E-43 | 3.58E-41 |
| *LOC_Os02g49326.1* | 1509 | 271 | 115 | 9.706301321 | 4.533025209 | -1.098447463 | 1.12E-12 | 3.64E-11 |
| *LOC_Os10g32300.1* | 1095 | 106 | 45 | 5.231972641 | 2.444431819 | -1.097855831 | 9.28E-06 | 0.000121717 |
| *LOC_Os04g34600.1* | 690 | 823 | 350 | 64.46507061 | 30.17161014 | -1.095325981 | 3.07E-35 | 3.20E-33 |
| *LOC_Os03g16900.1* | 1344 | 87 | 37 | 3.49859525 | 1.637502565 | -1.095278603 | 6.22E-05 | 0.000679662 |
| *LOC_Os04g33240.1* | 828 | 148 | 63 | 9.660622166 | 4.52574152 | -1.093961915 | 1.68E-07 | 3.04E-06 |
| *LOC_Os06g22960.1* | 747 | 10434 | 4444 | 754.9252454 | 353.8612295 | -1.093150089 | 0 | 0 |
| *LOC_Os07g37220.1* | 1296 | 432 | 184 | 18.01575485 | 8.444858075 | -1.093114019 | 3.42E-19 | 1.74E-17 |
| *LOC_Os01g36720.1* | 1458 | 115 | 49 | 4.262987259 | 1.999024375 | -1.09256868 | 4.19E-06 | 5.96E-05 |
| *LOC_Os05g44340.1* | 2739 | 527 | 225 | 10.39901731 | 4.886186276 | -1.089666433 | 5.79E-23 | 3.64E-21 |
| *LOC_Os12g16080.1* | 2538 | 98 | 42 | 2.086931413 | 0.984322033 | -1.084180894 | 2.47E-05 | 0.000295292 |
| *LOC_Os12g08270.1* | 1083 | 329 | 141 | 16.41879043 | 7.744086401 | -1.084180894 | 8.53E-15 | 3.27E-13 |
| *LOC_Os08g20130.1* | 1008 | 247 | 106 | 13.24372455 | 6.254964754 | -1.08223525 | 1.95E-11 | 5.64E-10 |
| *LOC_Os08g35880.1* | 1818 | 368 | 158 | 10.94026037 | 5.169430987 | -1.081569681 | 2.57E-16 | 1.10E-14 |
| *LOC_Os04g14410.1* | 1356 | 170 | 73 | 6.775837001 | 3.202157612 | -1.08135485 | 2.74E-08 | 5.56E-07 |
| *LOC_Os06g35560.1* | 1605 | 353 | 152 | 11.88703077 | 5.633108093 | -1.077385333 | 1.27E-15 | 5.17E-14 |
| *LOC_Os12g44020.1* | 1392 | 701 | 302 | 27.21776756 | 12.90468005 | -1.076654367 | 1.79E-29 | 1.48E-27 |
| *LOC_Os08g43654.1* | 1569 | 460 | 199 | 15.845597 | 7.544138738 | -1.070653903 | 1.02E-19 | 5.40E-18 |
| *LOC_Os07g43540.1* | 888 | 201 | 87 | 12.23367137 | 5.827547479 | -1.069896668 | 2.05E-09 | 4.83E-08 |
| *LOC_Os05g29735.1* | 447 | 122 | 53 | 14.75115498 | 7.05257771 | -1.064605356 | 3.44E-06 | 4.96E-05 |
| *LOC_Os01g70540.1* | 4686 | 520 | 226 | 5.997562434 | 2.868703667 | -1.063977323 | 7.03E-22 | 4.23E-20 |
| *LOC_Os07g46610.1* | 1437 | 193 | 84 | 7.258957591 | 3.476978872 | -1.061928087 | 5.32E-09 | 1.19E-07 |
| *LOC_Os06g48010.1* | 945 | 657 | 286 | 37.57571726 | 18.00170988 | -1.061666696 | 3.88E-27 | 2.94E-25 |
| *LOC_Os08g44270.1* | 1158 | 2168 | 945 | 101.186934 | 48.54033651 | -1.059766995 | 2.21E-85 | 6.19E-83 |
| *LOC_Os03g09070.1* | 1875 | 250 | 109 | 7.20630194 | 3.457838931 | -1.059388433 | 3.26E-11 | 9.21E-10 |
| *LOC_Os04g01520.1* | 1242 | 1311 | 573 | 57.04989036 | 27.44179779 | -1.055849114 | 4.87E-52 | 7.31E-50 |
| *LOC_Os03g20100.1* | 1209 | 702 | 307 | 31.38228264 | 15.10398718 | -1.055020848 | 1.31E-28 | 1.06E-26 |
| *LOC_Os02g42585.1* | 1422 | 160 | 70 | 6.08126746 | 2.928046553 | -1.054433551 | 1.29E-07 | 2.37E-06 |
| *LOC_Os10g03320.1* | 1479 | 96 | 42 | 3.508138875 | 1.689120567 | -1.054433551 | 4.48E-05 | 0.000506616 |
| *LOC_Os02g06300.1* | 2019 | 347 | 152 | 9.288955324 | 4.478027979 | -1.052652812 | 7.21E-15 | 2.78E-13 |
| *LOC_Os05g29710.1* | 603 | 940 | 413 | 84.25278387 | 40.73917906 | -1.048307448 | 2.23E-37 | 2.47E-35 |
| *LOC_Os01g61230.1* | 981 | 364 | 160 | 20.05423476 | 9.701312827 | -1.047655018 | 2.04E-15 | 8.16E-14 |
| *LOC_Os05g12210.1* | 1179 | 150 | 66 | 6.876242309 | 3.329734946 | -1.046213044 | 3.79E-07 | 6.45E-06 |
| *LOC_Os04g30570.1* | 1338 | 102 | 45 | 4.120195055 | 2.000487924 | -1.042360718 | 3.06E-05 | 0.000358921 |
| *LOC_Os01g40280.1* | 1116 | 215 | 95 | 10.41233143 | 5.063361609 | -1.040125714 | 1.37E-09 | 3.29E-08 |
| *LOC_Os08g37630.1* | 441 | 138 | 61 | 16.91274945 | 8.227554717 | -1.039575592 | 1.27E-06 | 1.97E-05 |
| *LOC_Os03g38390.1* | 1140 | 95 | 42 | 4.503938712 | 2.191411684 | -1.039326658 | 6.02E-05 | 0.000660937 |
| *LOC_Os12g28770.1* | 1137 | 496 | 220 | 23.57734672 | 11.50911024 | -1.03462507 | 4.45E-20 | 2.40E-18 |
| *LOC_Os10g01060.1* | 2436 | 2525 | 1121 | 56.02189778 | 27.37208389 | -1.033285582 | 3.37E-95 | 1.06E-92 |
| *LOC_Os08g19140.1* | 1827 | 225 | 100 | 6.656067063 | 3.255674563 | -1.031713474 | 7.37E-10 | 1.83E-08 |
| *LOC_Os02g42960.1* | 834 | 234 | 104 | 15.16434041 | 7.417316695 | -1.031713474 | 3.37E-10 | 8.67E-09 |
| *LOC_Os03g50220.1* | 684 | 256 | 114 | 20.22821597 | 9.913529045 | -1.028898459 | 5.54E-11 | 1.54E-09 |
| *LOC_Os02g58139.3* | 576 | 121 | 54 | 11.35367884 | 5.576360088 | -1.025764208 | 7.29E-06 | 9.82E-05 |
| *LOC_Os09g16160.1* | 2127 | 103 | 46 | 2.617239421 | 1.286381766 | -1.024727044 | 3.61E-05 | 0.000414744 |
| *LOC_Os05g31740.1* | 1581 | 94 | 42 | 3.213436349 | 1.580145047 | -1.024059902 | 8.07E-05 | 0.000859396 |
| *LOC_Os05g07060.1* | 828 | 212 | 95 | 13.83818851 | 6.824530864 | -1.019853319 | 3.22E-09 | 7.44E-08 |
| *LOC_Os07g09630.1* | 960 | 145 | 65 | 8.163388916 | 4.027371174 | -1.01932975 | 1.02E-06 | 1.61E-05 |
| *LOC_Os12g29340.1* | 330 | 165 | 74 | 27.02363227 | 13.33820271 | -1.018657321 | 1.85E-07 | 3.32E-06 |
| *LOC_Os08g44000.1* | 1161 | 399 | 179 | 18.57438291 | 9.170654775 | -1.018217632 | 4.62E-16 | 1.94E-14 |
| *LOC_Os06g38294.1* | 1773 | 238 | 107 | 7.255075557 | 3.589670415 | -1.01513925 | 4.09E-10 | 1.04E-08 |
| *LOC_Os05g47490.1* | 3909 | 120 | 54 | 1.659163916 | 0.821689284 | -1.013791566 | 9.72E-06 | 0.000126933 |
| *LOC_Os04g33150.1* | 972 | 1228 | 553 | 68.28193505 | 33.84062692 | -1.012747648 | 9.50E-46 | 1.28E-43 |
| *LOC_Os03g49710.1* | 516 | 344 | 155 | 36.0315097 | 17.867407 | -1.011928822 | 6.35E-14 | 2.27E-12 |
| *LOC_Os11g16550.1* | 822 | 195 | 88 | 12.82143137 | 6.367814277 | -1.009687168 | 1.80E-08 | 3.76E-07 |
| *LOC_Os09g23780.1* | 741 | 458 | 207 | 33.40573166 | 16.61619848 | -1.007505303 | 6.30E-18 | 2.99E-16 |
| *LOC_Os01g03630.1* | 1605 | 1918 | 867 | 64.58732299 | 32.13095208 | -1.007287295 | 7.46E-70 | 1.56E-67 |
| *LOC_Os01g59570.1* | 2814 | 6939 | 3149 | 133.2743315 | 66.56226644 | -1.001622482 | 2.11E-245 | 1.97E-242 |
| *LOC_Os01g23850.1* | 1716 | 260 | 118 | 8.188979477 | 4.090197298 | -1.001513236 | 1.03E-10 | 2.79E-09 |
| *LOC_Os04g54830.1* | 1914 | 1736 | 788 | 49.02092542 | 24.48859212 | -1.001287886 | 8.57E-63 | 1.61E-60 |
| *LOC_Os03g50150.1* | 2880 | 163 | 74 | 3.058925042 | 1.528335728 | -1.001063261 | 3.25E-07 | 5.61E-06 |
| *LOC_Os03g24820.1* | 2553 | 229 | 104 | 4.847952833 | 2.423048227 | -1.000552543 | 1.38E-09 | 3.30E-08 |
