# Supplementary material for: Transcriptome analysis of phosphorus stress responsiveness in the seedlings of Dongxiang wild rice (Oryza rufipogon Griff.)
Source: Biol Res. 2018 Mar 15;51:7. doi: 10.1186/s40659-018-0155-x (PMC5853122; doi:10.1186/s40659-018-0155-x)
Supplement: Supplementary file 6 — Additional file 6: Table S5. List of down-regulated genes in RLP vs. RCK. [file 40659_2018_155_MOESM6_ESM.docx]

| **Table S5** List of down-regulated genes in RLP vs. RCK. | | | | | | | | |
| --- | --- | --- | --- | --- | --- | --- | --- | --- |
| Gene ID | Gene  Length | RLP-  Expression | RCK-  Expression | RLP-RPKM | RCK-RPKM | Log_2_ Ratio  (RCK/RLP) | *P*-value | FDR |
| *LOC_Os09g03210.1* | 1077 | 0 | 165 | 0.001 | 9.112714721 | 13.15366519 | 6.22E-54 | 9.85E-52 |
| *LOC_Os03g19427.1* | 999 | 0 | 128 | 0.001 | 7.621211518 | 12.89580464 | 5.25E-42 | 6.49E-40 |
| *LOC_Os07g27030.1* | 846 | 0 | 60 | 0.001 | 4.218522998 | 12.04252225 | 4.35E-20 | 2.35E-18 |
| *LOC_Os01g60740.1* | 369 | 0 | 15 | 0.001 | 2.417933913 | 11.2395591 | 1.39E-05 | 0.000176333 |
| *LOC_Os12g32400.1* | 597 | 0 | 15 | 0.001 | 1.494501866 | 10.54544898 | 1.39E-05 | 0.000176398 |
| *LOC_Os04g01710.1* | 1152 | 0 | 18 | 0.001 | 0.929393348 | 9.860145509 | 1.50E-06 | 2.31E-05 |
| *LOC_Os01g31020.1* | 2253 | 0 | 33 | 0.001 | 0.871228917 | 9.76690803 | 2.20E-11 | 6.33E-10 |
| *LOC_Os01g51380.2* | 1467 | 0 | 20 | 0.001 | 0.810922621 | 9.663420448 | 3.40E-07 | 5.86E-06 |
| *LOC_Os02g19410.1* | 1296 | 0 | 16 | 0.001 | 0.734335485 | 9.520295506 | 6.63E-06 | 9.05E-05 |
| *LOC_Os02g05190.1* | 1080 | 0 | 13 | 0.001 | 0.715977098 | 9.48376963 | 6.14E-05 | 0.000672169 |
| *LOC_Os12g21720.1* | 1533 | 0 | 13 | 0.001 | 0.504406566 | 8.978443245 | 6.14E-05 | 0.000671956 |
| *LOC_Os11g21970.1* | 1734 | 0 | 13 | 0.001 | 0.445937293 | 8.800697044 | 6.14E-05 | 0.000672382 |
| *LOC_Os02g37950.3* | 2301 | 0 | 14 | 0.001 | 0.361901973 | 8.499455163 | 2.92E-05 | 0.000344707 |
| *LOC_Os02g43410.1* | 2019 | 21 | 5497 | 0.562155798 | 161.945525 | 8.170322869 | 0 | 0 |
| *LOC_Os03g19420.2* | 981 | 4 | 973 | 0.220376206 | 58.99610863 | 8.064507522 | 6.00E-305 | 6.90E-302 |
| *LOC_Os10g11889.1* | 180 | 6 | 333 | 1.801575485 | 110.0401724 | 5.932627394 | 1.89E-97 | 6.35E-95 |
| *LOC_Os11g15624.1* | 390 | 4 | 182 | 0.554330918 | 27.75788132 | 5.646006167 | 7.82E-53 | 1.20E-50 |
| *LOC_Os11g04020.1* | 1422 | 20 | 819 | 0.760158432 | 34.25814467 | 5.494003074 | 2.43E-230 | 1.99E-227 |
| *LOC_Os10g11889.2* | 180 | 4 | 139 | 1.201050323 | 45.93268457 | 5.2571526 | 1.99E-39 | 2.29E-37 |
| *LOC_Os02g43370.1* | 2025 | 2 | 64 | 0.053380014 | 1.879898841 | 5.138211527 | 1.40E-18 | 6.87E-17 |
| *LOC_Os10g11870.1* | 4845 | 2 | 47 | 0.022310532 | 0.577010359 | 4.692800379 | 2.35E-13 | 8.07E-12 |
| *LOC_Os02g36210.1* | 2403 | 4 | 79 | 0.089966316 | 1.955477639 | 4.441992275 | 4.98E-21 | 2.85E-19 |
| *LOC_Os11g44820.1* | 840 | 1 | 19 | 0.064341982 | 1.345407513 | 4.386139041 | 8.21E-06 | 0.000109095 |
| *LOC_Os03g46454.1* | 705 | 9 | 155 | 0.689965079 | 13.07742129 | 4.244410931 | 6.53E-39 | 7.44E-37 |
| *LOC_Os05g11070.1* | 1239 | 1 | 17 | 0.043621682 | 0.816125878 | 4.225674369 | 3.29E-05 | 0.000382588 |
| *LOC_Os09g26500.1* | 1374 | 2 | 29 | 0.078671419 | 1.255425076 | 3.996192522 | 6.17E-08 | 1.19E-06 |
| *LOC_Os11g39420.1* | 1425 | 2 | 29 | 0.07585581 | 1.210494073 | 3.996192522 | 6.17E-08 | 1.19E-06 |
| *LOC_Os08g26580.1* | 222 | 2 | 21 | 0.486912293 | 5.626597566 | 3.53052895 | 1.33E-05 | 0.000168901 |
| *LOC_Os09g06540.1* | 1725 | 2 | 21 | 0.062663495 | 0.724118643 | 3.53052895 | 1.33E-05 | 0.000168964 |
| *LOC_Os07g34140.1* | 1197 | 2 | 20 | 0.090304536 | 0.993837498 | 3.460139622 | 2.57E-05 | 0.000306504 |
| *LOC_Os10g20610.1* | 1800 | 2 | 18 | 0.060052516 | 0.594811743 | 3.308136529 | 9.48E-05 | 0.000989944 |
| *LOC_Os08g25860.1* | 510 | 32 | 255 | 3.391200913 | 29.74058713 | 3.132564964 | 2.27E-49 | 3.21E-47 |
| *LOC_Os01g72370.3* | 744 | 5 | 39 | 0.363220864 | 3.11796478 | 3.101685651 | 1.38E-08 | 2.93E-07 |
| *LOC_Os01g65110.1* | 1647 | 18 | 140 | 0.590680487 | 5.056080387 | 3.097569543 | 1.72E-27 | 1.33E-25 |
| *LOC_Os01g72360.1* | 657 | 8 | 62 | 0.658109766 | 5.613139733 | 3.092407838 | 6.71E-13 | 2.21E-11 |
| *LOC_Os07g15460.1* | 1557 | 17 | 131 | 0.590111431 | 5.004517552 | 3.084171688 | 1.04E-25 | 7.31E-24 |
| *LOC_Os04g11750.1* | 588 | 3 | 23 | 0.27575135 | 2.326644572 | 3.076810983 | 1.73E-05 | 0.000214123 |
| *LOC_Os08g26350.1* | 312 | 60 | 441 | 10.39370472 | 84.07435209 | 3.015955777 | 3.72E-81 | 9.72E-79 |
| *LOC_Os08g25990.1* | 348 | 3 | 22 | 0.465924694 | 3.76030412 | 3.012680645 | 3.24E-05 | 0.000376993 |
| *LOC_Os02g36140.2* | 2490 | 7 | 48 | 0.151940101 | 1.146625046 | 2.915819106 | 9.22E-10 | 2.28E-08 |
| *LOC_Os07g03770.1* | 1068 | 12 | 82 | 0.607272635 | 4.566906639 | 2.910801031 | 9.46E-16 | 3.88E-14 |
| *LOC_Os05g50340.1* | 273 | 9 | 57 | 1.781777952 | 12.41914628 | 2.80117654 | 5.94E-11 | 1.64E-09 |
| *LOC_Os02g20360.1* | 1485 | 33 | 209 | 1.201050323 | 8.371424527 | 2.80117654 | 1.63E-36 | 1.76E-34 |
| *LOC_Os05g38580.1* | 591 | 4 | 25 | 0.365802129 | 2.516124123 | 2.782067717 | 1.97E-05 | 0.000241755 |
| *LOC_Os03g12760.1* | 969 | 6 | 36 | 0.334657985 | 2.209826908 | 2.723174028 | 3.47E-07 | 5.96E-06 |
| *LOC_Os04g45340.1* | 579 | 5 | 29 | 0.4667294 | 2.979195257 | 2.674264428 | 6.38E-06 | 8.75E-05 |
| *LOC_Os08g25850.1* | 207 | 7 | 40 | 1.827685275 | 11.49394672 | 2.6527847 | 1.14E-07 | 2.11E-06 |
| *LOC_Os08g26520.1* | 678 | 24 | 137 | 1.913177506 | 12.01905734 | 2.65128111 | 4.10E-23 | 2.59E-21 |
| *LOC_Os08g30450.1* | 2202 | 5 | 28 | 0.122723126 | 0.75634554 | 2.623638354 | 1.16E-05 | 0.000148843 |
| *LOC_Os02g13410.1* | 1527 | 7 | 38 | 0.247760872 | 1.480212588 | 2.578784119 | 3.69E-07 | 6.29E-06 |
| *LOC_Os01g39350.1* | 1554 | 5 | 27 | 0.173897248 | 1.033456696 | 2.571170935 | 2.08E-05 | 0.000253507 |
| *LOC_Os03g20350.1* | 1299 | 5 | 27 | 0.208034121 | 1.236329257 | 2.571170935 | 2.08E-05 | 0.000253418 |
| *LOC_Os02g49920.1* | 1458 | 12 | 62 | 0.444833453 | 2.529377781 | 2.507445337 | 1.32E-10 | 3.55E-09 |
| *LOC_Os09g31442.1* | 786 | 6 | 31 | 0.412574539 | 2.345949621 | 2.507445337 | 6.59E-06 | 9.00E-05 |
| *LOC_Os11g05390.1* | 1356 | 160 | 817 | 6.377258354 | 35.83784615 | 2.490475701 | 3.98E-121 | 1.72E-118 |
| *LOC_Os12g14330.1* | 2817 | 59 | 300 | 1.131980337 | 6.334523351 | 2.484387168 | 1.97E-45 | 2.62E-43 |
| *LOC_Os06g47890.2* | 1893 | 8 | 38 | 0.228408936 | 1.194022516 | 2.386139041 | 1.16E-06 | 1.83E-05 |
| *LOC_Os09g08190.1* | 843 | 92 | 420 | 5.898396606 | 29.63474875 | 2.328895089 | 2.13E-58 | 3.72E-56 |
| *LOC_Os09g36350.2* | 1188 | 6 | 27 | 0.272965983 | 1.35184487 | 2.308136529 | 6.40E-05 | 0.000697906 |
| *LOC_Os02g18180.1* | 1827 | 271 | 1158 | 8.016862996 | 37.70071144 | 2.233482024 | 5.01E-150 | 2.70E-147 |
| *LOC_Os01g13610.1* | 981 | 19 | 81 | 1.046786979 | 4.911289618 | 2.230134017 | 7.04E-12 | 2.10E-10 |
| *LOC_Os10g37290.1* | 303 | 12 | 50 | 2.140485725 | 9.815375292 | 2.197105216 | 1.04E-07 | 1.94E-06 |
| *LOC_Os01g62100.1* | 5409 | 14 | 58 | 0.139889389 | 0.637808857 | 2.1888376 | 1.05E-08 | 2.27E-07 |
| *LOC_Os07g10030.1* | 1284 | 8 | 33 | 0.336743081 | 1.528721769 | 2.182605647 | 1.84E-05 | 0.000227075 |
| *LOC_Os03g53060.1* | 1002 | 17 | 70 | 0.916969558 | 4.155371456 | 2.180031703 | 3.42E-10 | 8.77E-09 |
| *LOC_Os10g38189.1* | 672 | 58 | 235 | 4.664793666 | 20.80070826 | 2.156747479 | 1.70E-30 | 1.49E-28 |
| *LOC_Os08g05480.1* | 900 | 148 | 598 | 8.887772393 | 39.52193579 | 2.152759836 | 6.98E-75 | 1.55E-72 |
| *LOC_Os12g31729.1* | 2337 | 14 | 56 | 0.323774798 | 1.425308412 | 2.138211527 | 3.05E-08 | 6.14E-07 |
| *LOC_Os03g03540.1* | 1101 | 13 | 52 | 0.638160254 | 2.809283435 | 2.138211527 | 9.62E-08 | 1.80E-06 |
| *LOC_Os11g47150.1* | 2085 | 63 | 248 | 1.633082814 | 7.074979002 | 2.115127914 | 2.40E-31 | 2.16E-29 |
| *LOC_Os01g41240.1* | 909 | 9 | 35 | 0.535121431 | 2.290254235 | 2.097569543 | 1.67E-05 | 0.000207508 |
| *LOC_Os08g13870.1* | 2373 | 8 | 31 | 0.182207382 | 0.777040203 | 2.092407838 | 5.37E-05 | 0.000597432 |
| *LOC_Os04g47390.1* | 1104 | 10 | 38 | 0.489558556 | 2.047359259 | 2.064210946 | 8.82E-06 | 0.000116339 |
| *LOC_Os02g16940.1* | 2208 | 8 | 30 | 0.195823422 | 0.808168129 | 2.045102123 | 9.10E-05 | 0.000957061 |
| *LOC_Os04g50060.1* | 1911 | 8 | 30 | 0.226257518 | 0.933770397 | 2.045102123 | 9.10E-05 | 0.000956769 |
| *LOC_Os09g28570.2* | 1791 | 8 | 30 | 0.24141715 | 0.996334577 | 2.045102123 | 9.10E-05 | 0.000957353 |
| *LOC_Os12g37450.1* | 3114 | 10 | 37 | 0.173562185 | 0.706744845 | 2.025736798 | 1.48E-05 | 0.000187122 |
| *LOC_Os04g08800.1* | 360 | 45 | 157 | 6.755908069 | 25.940401 | 1.94097918 | 2.16E-18 | 1.05E-16 |
| *LOC_Os01g58670.1* | 1689 | 19 | 66 | 0.607991727 | 2.324308764 | 1.934678133 | 1.71E-08 | 3.57E-07 |
| *LOC_Os10g34040.1* | 1458 | 59 | 202 | 2.187097811 | 8.240875996 | 1.913779961 | 7.93E-23 | 4.96E-21 |
| *LOC_Os01g01660.1* | 945 | 434 | 1431 | 24.82170668 | 90.07149246 | 1.859468252 | 8.09E-146 | 4.29E-143 |
| *LOC_Os04g45590.1* | 528 | 75 | 245 | 7.67716826 | 27.60016609 | 1.846030776 | 3.99E-26 | 2.88E-24 |
| *LOC_Os08g02820.1* | 1221 | 68 | 222 | 3.010003267 | 10.81475896 | 1.845164552 | 8.38E-24 | 5.44E-22 |
| *LOC_Os03g60570.1* | 435 | 12 | 39 | 1.490959022 | 5.332794934 | 1.838651245 | 3.04E-05 | 0.000356185 |
| *LOC_Os10g38314.1* | 495 | 25 | 81 | 2.729659826 | 9.733283062 | 1.83420534 | 1.62E-09 | 3.85E-08 |
| *LOC_Os05g06560.1* | 1209 | 13 | 42 | 0.581153382 | 2.066343523 | 1.830089232 | 1.58E-05 | 0.0001979 |
| *LOC_Os01g65100.1* | 1638 | 109 | 352 | 3.596551792 | 12.78227921 | 1.829458821 | 2.10E-36 | 2.26E-34 |
| *LOC_Os12g32480.1* | 1344 | 18 | 57 | 0.723847293 | 2.522639087 | 1.80117654 | 6.13E-07 | 1.01E-05 |
| *LOC_Os04g02040.1* | 315 | 13 | 41 | 2.230522029 | 7.741994111 | 1.795323814 | 2.57E-05 | 0.00030655 |
| *LOC_Os01g57540.1* | 537 | 251 | 760 | 25.26231546 | 84.18192261 | 1.736523582 | 2.25E-71 | 4.80E-69 |
| *LOC_Os03g32490.1* | 939 | 17 | 51 | 0.978491477 | 3.230606909 | 1.723174028 | 4.87E-06 | 6.83E-05 |
| *LOC_Os04g02450.1* | 2283 | 19 | 57 | 0.449802026 | 1.48507531 | 1.723174028 | 1.33E-06 | 2.06E-05 |
| *LOC_Os08g07340.1* | 2703 | 21 | 63 | 0.419901056 | 1.386353673 | 1.723174028 | 3.64E-07 | 6.24E-06 |
| *LOC_Os10g39510.1* | 1128 | 125 | 374 | 5.989280203 | 19.72159501 | 1.719321703 | 1.64E-35 | 1.72E-33 |
| *LOC_Os09g11440.2* | 1494 | 19 | 56 | 0.687348077 | 2.229548701 | 1.697638936 | 2.12E-06 | 3.18E-05 |
| *LOC_Os02g08230.1* | 1887 | 17 | 50 | 0.486912293 | 1.57607775 | 1.694604876 | 7.77E-06 | 0.000103783 |
| *LOC_Os06g07250.1* | 552 | 2364 | 6858 | 231.4632851 | 738.9889368 | 1.674769396 | 0 | 0 |
| *LOC_Os10g35500.1* | 918 | 309 | 893 | 18.1923799 | 57.86131658 | 1.669264865 | 4.40E-79 | 1.07E-76 |
| *LOC_Os04g56030.1* | 585 | 34 | 98 | 3.141208538 | 9.964367655 | 1.66545853 | 5.43E-10 | 1.37E-08 |
| *LOC_Os09g25200.1* | 1863 | 22 | 63 | 0.638239302 | 2.011440676 | 1.656059832 | 7.60E-07 | 1.23E-05 |
| *LOC_Os01g13520.2* | 1644 | 14 | 40 | 0.460256511 | 1.447230517 | 1.6527847 | 8.79E-05 | 0.000928737 |
| *LOC_Os03g59100.1* | 1632 | 35 | 98 | 1.159101875 | 3.5717862 | 1.623638354 | 1.10E-09 | 2.69E-08 |
| *LOC_Os12g12600.1* | 465 | 56 | 156 | 6.508917881 | 19.95497459 | 1.616258824 | 1.67E-14 | 6.27E-13 |
| *LOC_Os12g37580.1* | 3516 | 31 | 86 | 0.476525939 | 1.454886515 | 1.610279972 | 1.42E-08 | 3.00E-07 |
| *LOC_Os01g01410.2* | 1665 | 16 | 44 | 0.519373113 | 1.571874876 | 1.597643146 | 5.89E-05 | 0.00064831 |
| *LOC_Os04g35280.1* | 1725 | 24 | 66 | 0.751961942 | 2.27580145 | 1.597643146 | 8.07E-07 | 1.30E-05 |
| *LOC_Os10g28240.1* | 3108 | 55 | 151 | 0.956434862 | 2.889851131 | 1.595256553 | 7.57E-14 | 2.69E-12 |
| *LOC_Os01g02770.1* | 1992 | 53 | 145 | 1.438004529 | 4.32970395 | 1.590200163 | 2.67E-13 | 9.11E-12 |
| *LOC_Os02g07310.1* | 2631 | 34 | 93 | 0.698444316 | 2.102527255 | 1.589907497 | 5.06E-09 | 1.14E-07 |
| *LOC_Os08g31250.1* | 810 | 67 | 183 | 4.470576203 | 13.43833937 | 1.587822175 | 2.26E-16 | 9.71E-15 |
| *LOC_Os02g19060.1* | 753 | 17 | 45 | 1.220190568 | 3.554651849 | 1.542601782 | 7.46E-05 | 0.000801075 |
| *LOC_Os10g20440.1* | 5127 | 22 | 58 | 0.231917265 | 0.67289021 | 1.536760904 | 7.05E-06 | 9.56E-05 |
| *LOC_Os07g38860.1* | 1437 | 19 | 50 | 0.714612405 | 2.069630281 | 1.534140204 | 3.16E-05 | 0.000368779 |
| *LOC_Os04g07980.1* | 2784 | 29 | 76 | 0.562992339 | 1.623767688 | 1.528158046 | 2.92E-07 | 5.08E-06 |
| *LOC_Os11g04550.1* | 1017 | 245 | 638 | 13.02023581 | 37.3146403 | 1.518986202 | 6.59E-50 | 9.47E-48 |
| *LOC_Os11g42200.1* | 1773 | 33 | 85 | 1.005955855 | 2.851607339 | 1.503208344 | 8.29E-08 | 1.57E-06 |
| *LOC_Os11g15670.1* | 2658 | 159 | 407 | 3.233075645 | 9.107914946 | 1.494213556 | 1.06E-31 | 9.71E-30 |
| *LOC_Os03g52680.1* | 747 | 199 | 508 | 14.39813339 | 40.45038357 | 1.490271594 | 5.19E-39 | 5.92E-37 |
| *LOC_Os12g04350.1* | 873 | 101 | 257 | 6.252890858 | 17.5104946 | 1.485624594 | 1.95E-20 | 1.07E-18 |
| *LOC_Os03g08410.1* | 1620 | 26 | 66 | 0.867425234 | 2.4233071 | 1.482165929 | 3.03E-06 | 4.41E-05 |
| *LOC_Os12g05310.1* | 7326 | 232 | 587 | 1.711570485 | 4.765963595 | 1.477447225 | 2.91E-44 | 3.78E-42 |
| *LOC_Os01g09990.1* | 1320 | 34 | 86 | 1.392126511 | 3.875288627 | 1.477013441 | 1.03E-07 | 1.91E-06 |
| *LOC_Os08g44820.5* | 1533 | 20 | 50 | 0.705117607 | 1.940025253 | 1.460139622 | 6.02E-05 | 0.000660998 |
| *LOC_Os02g50710.1* | 279 | 21 | 52 | 4.068073676 | 11.086097 | 1.446333823 | 4.84E-05 | 0.000541403 |
| *LOC_Os03g57720.1* | 435 | 80 | 198 | 9.939726814 | 27.07418967 | 1.445640052 | 1.66E-15 | 6.71E-14 |
| *LOC_Os10g40290.1* | 1422 | 24 | 59 | 0.912190119 | 2.467924952 | 1.435892076 | 1.65E-05 | 0.000205559 |
| *LOC_Os08g38092.1* | 1245 | 41 | 100 | 1.779869756 | 4.777604359 | 1.424515712 | 2.29E-08 | 4.71E-07 |
| *LOC_Os09g11970.1* | 504 | 32 | 78 | 3.431572352 | 9.205419827 | 1.423613746 | 8.29E-07 | 1.33E-05 |
| *LOC_Os12g25490.1* | 1095 | 101 | 244 | 4.985181479 | 13.25425253 | 1.410737382 | 3.88E-18 | 1.86E-16 |
| *LOC_Os09g34160.1* | 2928 | 245 | 587 | 4.522397478 | 11.92467531 | 1.398790281 | 6.88E-41 | 8.16E-39 |
| *LOC_Os06g29950.1* | 1488 | 28 | 67 | 1.017018419 | 2.678251798 | 1.396945796 | 6.81E-06 | 9.28E-05 |
| *LOC_Os01g69050.3* | 1257 | 31 | 74 | 1.332907877 | 3.50167613 | 1.393468583 | 2.34E-06 | 3.48E-05 |
| *LOC_Os02g37880.1* | 2004 | 26 | 62 | 0.701212015 | 1.84023593 | 1.39196812 | 1.60E-05 | 0.000199755 |
| *LOC_Os07g02480.1* | 1977 | 172 | 409 | 4.702139354 | 12.30541238 | 1.387903806 | 1.15E-28 | 9.28E-27 |
| *LOC_Os07g47780.2* | 564 | 134 | 318 | 12.84101675 | 33.53725783 | 1.385005292 | 1.42E-22 | 8.71E-21 |
| *LOC_Os12g23520.1* | 5541 | 22 | 52 | 0.214589392 | 0.558206292 | 1.379219627 | 8.85E-05 | 0.000933972 |
| *LOC_Os01g02840.1* | 1911 | 69 | 163 | 1.95147109 | 5.073485822 | 1.378415225 | 3.23E-12 | 1.00E-10 |
| *LOC_Os02g29160.1* | 1344 | 26 | 61 | 1.045557201 | 2.699666392 | 1.368509147 | 2.39E-05 | 0.000287553 |
| *LOC_Os09g19970.1* | 804 | 65 | 151 | 4.369492781 | 11.17121557 | 1.354248454 | 3.73E-11 | 1.05E-09 |
| *LOC_Os02g32770.1* | 1542 | 730 | 1683 | 25.5865779 | 64.92011433 | 1.343278335 | 3.74E-107 | 1.45E-104 |
| *LOC_Os12g16480.1* | 720 | 43 | 99 | 3.227822744 | 8.178661462 | 1.341303393 | 1.09E-07 | 2.03E-06 |
| *LOC_Os11g47630.1* | 513 | 33 | 75 | 3.47672462 | 8.696078109 | 1.322636098 | 4.93E-06 | 6.90E-05 |
| *LOC_Os12g03320.1* | 654 | 27 | 61 | 2.231309087 | 5.547938273 | 1.314061363 | 4.23E-05 | 0.000479477 |
| *LOC_Os04g58390.1* | 660 | 209 | 471 | 17.11496711 | 42.44792891 | 1.310435645 | 3.92E-30 | 3.37E-28 |
| *LOC_Os01g25280.1* | 435 | 1264 | 2848 | 157.0476837 | 389.4307685 | 1.31016421 | 3.20E-173 | 2.01E-170 |
| *LOC_Os07g08680.1* | 366 | 213 | 476 | 31.45373593 | 77.35802992 | 1.29831967 | 5.05E-30 | 4.32E-28 |
| *LOC_Os02g53180.2* | 966 | 76 | 169 | 4.252165741 | 10.40612676 | 1.29116345 | 1.52E-11 | 4.46E-10 |
| *LOC_Os10g06090.1* | 2247 | 271 | 601 | 6.518383931 | 15.90929494 | 1.287283667 | 5.33E-37 | 5.86E-35 |
| *LOC_Os06g19110.1* | 2568 | 36 | 79 | 0.757671933 | 1.829833632 | 1.272067274 | 5.39E-06 | 7.47E-05 |
| *LOC_Os01g01650.1* | 957 | 53 | 116 | 2.993213188 | 7.209839305 | 1.268272068 | 3.66E-08 | 7.30E-07 |
| *LOC_Os02g56720.2* | 1005 | 27 | 59 | 1.452016063 | 3.491929634 | 1.265967074 | 9.17E-05 | 0.000963844 |
| *LOC_Os08g36820.1* | 1197 | 38 | 83 | 1.715786176 | 4.124425618 | 1.265323445 | 3.41E-06 | 4.91E-05 |
| *LOC_Os01g52340.1* | 1524 | 28 | 61 | 0.992994362 | 2.380808156 | 1.261593943 | 7.27E-05 | 0.000782822 |
| *LOC_Os02g49230.2* | 1443 | 28 | 61 | 1.04873417 | 2.514450194 | 1.261593943 | 7.27E-05 | 0.000783066 |
| *LOC_Os11g37950.1* | 453 | 115 | 250 | 13.720608 | 32.82625511 | 1.258505761 | 8.52E-16 | 3.51E-14 |
| *LOC_Os03g13390.2* | 957 | 622 | 1344 | 35.12789817 | 83.53468988 | 1.24975818 | 5.27E-77 | 1.23E-74 |
| *LOC_Os12g12720.1* | 783 | 38 | 82 | 2.622983465 | 6.229190664 | 1.247836018 | 4.97E-06 | 6.94E-05 |
| *LOC_Os11g34880.1* | 1764 | 66 | 141 | 2.022176565 | 4.754447603 | 1.23336876 | 2.81E-09 | 6.54E-08 |
| *LOC_Os02g29210.1* | 1116 | 77 | 164 | 3.729067536 | 8.740961093 | 1.228976991 | 1.63E-10 | 4.31E-09 |
| *LOC_Os03g18960.1* | 1527 | 42 | 89 | 1.486565233 | 3.466813693 | 1.221627535 | 2.88E-06 | 4.20E-05 |
| *LOC_Os07g08320.1* | 1953 | 56 | 118 | 1.549742353 | 3.593844631 | 1.213499655 | 8.13E-08 | 1.54E-06 |
| *LOC_Os11g45400.1* | 1632 | 39 | 82 | 1.29157066 | 2.988637433 | 1.210361313 | 8.29E-06 | 0.000109912 |
| *LOC_Os12g13730.1* | 504 | 120 | 248 | 12.86839632 | 29.26851432 | 1.185517242 | 2.10E-14 | 7.81E-13 |
| *LOC_Os01g67820.1* | 1779 | 249 | 514 | 7.564794195 | 17.18567936 | 1.183834144 | 3.86E-28 | 3.06E-26 |
| *LOC_Os01g09220.1* | 1362 | 79 | 163 | 3.134900073 | 7.118525261 | 1.183158933 | 6.27E-10 | 1.57E-08 |
| *LOC_Os05g33900.1* | 1263 | 509 | 1049 | 21.78151833 | 49.4028122 | 1.181488644 | 1.69E-55 | 2.74E-53 |
| *LOC_Os07g44360.1* | 1899 | 34 | 70 | 0.967670877 | 2.192565665 | 1.180031703 | 5.49E-05 | 0.000608872 |
| *LOC_Os12g06480.1* | 2187 | 36 | 74 | 0.889666906 | 2.012623181 | 1.177739891 | 3.44E-05 | 0.000397605 |
| *LOC_Os10g10300.1* | 3918 | 72 | 148 | 0.9932116 | 2.246864163 | 1.177739891 | 4.37E-09 | 9.92E-08 |
| *LOC_Os08g03880.1* | 4353 | 59 | 121 | 0.732549646 | 1.653393542 | 1.174431715 | 1.20E-07 | 2.22E-06 |
| *LOC_Os04g31290.1* | 1059 | 124 | 253 | 6.328480457 | 14.21032775 | 1.167008792 | 2.45E-14 | 8.99E-13 |
| *LOC_Os09g13890.1* | 1311 | 248 | 506 | 10.22404394 | 22.95764621 | 1.167008792 | 3.88E-27 | 2.94E-25 |
| *LOC_Os01g64470.1* | 717 | 65 | 132 | 4.899682281 | 10.95050907 | 1.160237834 | 4.35E-08 | 8.58E-07 |
| *LOC_Os07g07719.1* | 381 | 34 | 69 | 4.823115472 | 10.77218117 | 1.159273143 | 7.87E-05 | 0.000839896 |
| *LOC_Os12g30760.1* | 3168 | 108 | 219 | 1.842520382 | 4.111861479 | 1.158111085 | 1.80E-12 | 5.71E-11 |
| *LOC_Os03g37930.1* | 2400 | 112 | 227 | 2.522205679 | 5.625927733 | 1.157405093 | 7.31E-13 | 2.40E-11 |
| *LOC_Os11g37090.1* | 2241 | 38 | 77 | 0.916464102 | 2.043752976 | 1.157070555 | 3.09E-05 | 0.000362028 |
| *LOC_Os02g36600.1* | 1137 | 60 | 121 | 2.852098393 | 6.33001063 | 1.150184169 | 1.94E-07 | 3.46E-06 |
| *LOC_Os07g10840.1* | 2292 | 134 | 268 | 3.159831348 | 6.955041319 | 1.138211527 | 1.45E-14 | 5.48E-13 |
| *LOC_Os03g06040.1* | 750 | 250 | 499 | 18.01575485 | 39.57480795 | 1.135323248 | 1.07E-25 | 7.56E-24 |
| *LOC_Os03g13050.1* | 1053 | 70 | 139 | 3.592885583 | 7.851740953 | 1.127869583 | 3.94E-08 | 7.82E-07 |
| *LOC_Os09g12350.1* | 2001 | 47 | 93 | 1.269475979 | 2.764492357 | 1.122781487 | 7.73E-06 | 0.000103367 |
| *LOC_Os08g01520.1* | 1575 | 380 | 749 | 13.03997494 | 28.28660287 | 1.117177827 | 8.10E-37 | 8.87E-35 |
| *LOC_Os01g42690.1* | 765 | 59 | 116 | 4.168351122 | 9.019367601 | 1.113549473 | 6.88E-07 | 1.12E-05 |
| *LOC_Os05g39540.1* | 1089 | 71 | 139 | 3.523742684 | 7.592179268 | 1.107405481 | 6.21E-08 | 1.20E-06 |
| *LOC_Os12g06480.2* | 2376 | 44 | 86 | 1.000875269 | 2.152938126 | 1.105044663 | 2.19E-05 | 0.000264947 |
| *LOC_Os10g34790.1* | 1032 | 63 | 123 | 3.299396964 | 7.089326003 | 1.103446109 | 3.86E-07 | 6.57E-06 |
| *LOC_Os04g27070.1* | 1572 | 928 | 1811 | 31.90576431 | 68.52443168 | 1.102801363 | 9.37E-85 | 2.59E-82 |
| *LOC_Os01g14650.1* | 756 | 287 | 560 | 20.51794302 | 44.06012909 | 1.102587618 | 2.16E-27 | 1.66E-25 |
| *LOC_Os01g02400.1* | 1869 | 116 | 226 | 3.354458367 | 7.192480142 | 1.100409495 | 6.34E-12 | 1.90E-10 |
| *LOC_Os04g56040.1* | 663 | 57 | 111 | 4.646597405 | 9.958386642 | 1.099737379 | 1.53E-06 | 2.35E-05 |
| *LOC_Os02g51550.1* | 444 | 82 | 159 | 9.981702011 | 21.30069079 | 1.093542478 | 9.95E-09 | 2.15E-07 |
| *LOC_Os01g21650.1* | 1488 | 65 | 126 | 2.360935615 | 5.036712337 | 1.093123638 | 3.43E-07 | 5.89E-06 |
| *LOC_Os10g02840.1* | 651 | 43 | 83 | 3.569942205 | 7.583621297 | 1.086986204 | 3.88E-05 | 0.000444161 |
| *LOC_Os02g53410.1* | 306 | 88 | 169 | 15.54300418 | 32.85071389 | 1.079659345 | 4.96E-09 | 1.11E-07 |
| *LOC_Os06g23140.1* | 1785 | 242 | 464 | 7.327416258 | 15.46177303 | 1.077329285 | 3.53E-22 | 2.14E-20 |
| *ChrUn.fgenesh.mRNA.18* | 354 | 205 | 393 | 31.29855715 | 66.03418499 | 1.07711693 | 5.10E-19 | 2.58E-17 |
| *LOC_Os03g06050.1* | 750 | 47 | 90 | 3.386961912 | 7.137740912 | 1.075475772 | 2.15E-05 | 0.000260903 |
| *LOC_Os05g39800.1* | 1686 | 66 | 126 | 2.115729217 | 4.445212312 | 1.071097331 | 5.32E-07 | 8.82E-06 |
| *LOC_Os06g43470.1* | 984 | 64 | 122 | 3.515269239 | 7.374698436 | 1.068948865 | 8.40E-07 | 1.35E-05 |
| *LOC_Os01g41950.1* | 810 | 53 | 101 | 3.536425952 | 7.416788396 | 1.068502555 | 7.52E-06 | 0.000100882 |
| *LOC_Os04g11524.1* | 465 | 84 | 160 | 9.763376822 | 20.46664061 | 1.067822199 | 1.71E-08 | 3.57E-07 |
| *LOC_Os08g10500.1* | 276 | 103 | 196 | 20.16981249 | 42.24025419 | 1.066420844 | 4.47E-10 | 1.13E-08 |
| *LOC_Os06g40570.1* | 3012 | 41 | 78 | 0.735703136 | 1.540349134 | 1.066061742 | 8.67E-05 | 0.00091717 |
| *LOC_Os01g65190.1* | 1989 | 59 | 112 | 1.60321197 | 3.349367279 | 1.0629234 | 2.64E-06 | 3.90E-05 |
| *LOC_Os07g12240.1* | 453 | 108 | 205 | 12.88544055 | 26.91752919 | 1.062804125 | 2.00E-10 | 5.24E-09 |
| *LOC_Os06g22600.1* | 2007 | 58 | 110 | 1.561904008 | 3.260054394 | 1.061590246 | 3.32E-06 | 4.80E-05 |
| *LOC_Os09g37540.1* | 684 | 67 | 127 | 5.294103399 | 11.0440192 | 1.060807024 | 5.89E-07 | 9.71E-06 |
| *LOC_Os01g70930.1* | 1101 | 806 | 1527 | 39.56593572 | 82.49568856 | 1.060059846 | 2.18E-67 | 4.39E-65 |
| *LOC_Os02g13290.1* | 891 | 72 | 136 | 4.367455721 | 9.079056903 | 1.055749367 | 2.61E-07 | 4.58E-06 |
| *LOC_Os04g15660.1* | 2676 | 375 | 705 | 7.573887969 | 15.67048874 | 1.048944189 | 1.59E-31 | 1.44E-29 |
| *LOC_Os12g34440.1* | 1428 | 389 | 730 | 14.72295932 | 30.40704287 | 1.046337836 | 1.86E-32 | 1.78E-30 |
| *LOC_Os03g46470.1* | 1125 | 1947 | 3621 | 93.53779918 | 191.4500729 | 1.03334682 | 8.19E-151 | 4.56E-148 |
| *LOC_Os12g16490.1* | 3570 | 71 | 132 | 1.074889575 | 2.199303923 | 1.032858527 | 6.28E-07 | 1.03E-05 |
| *LOC_Os08g28670.1* | 456 | 826 | 1533 | 97.90140464 | 199.9663161 | 1.030355538 | 1.21E-64 | 2.35E-62 |
| *LOC_Os03g61470.1* | 453 | 76 | 141 | 9.067532242 | 18.51400788 | 1.029835366 | 2.78E-07 | 4.86E-06 |
| *LOC_Os04g01320.1* | 2442 | 522 | 968 | 11.55310078 | 23.57812313 | 1.029168768 | 2.15E-41 | 2.60E-39 |
| *LOC_Os04g02120.1* | 1020 | 48 | 89 | 2.543400685 | 5.190024029 | 1.028982458 | 4.61E-05 | 0.000519595 |
| *LOC_Os03g63970.1* | 1119 | 321 | 595 | 15.50417509 | 31.62761277 | 1.028527898 | 4.65E-26 | 3.34E-24 |
| *LOC_Os11g03940.1* | 6084 | 398 | 736 | 3.535636307 | 7.195618715 | 1.025148863 | 1.11E-31 | 1.01E-29 |
| *LOC_Os08g27170.1* | 1563 | 119 | 220 | 4.114922893 | 8.37227021 | 1.024753478 | 1.60E-10 | 4.22E-09 |
| *LOC_Os11g15700.1* | 1227 | 45 | 83 | 1.982173516 | 4.023583915 | 1.021397862 | 9.23E-05 | 0.000967778 |
| *LOC_Os08g35630.1* | 699 | 178 | 327 | 13.76310886 | 27.82595706 | 1.015624922 | 9.69E-15 | 3.70E-13 |
| *LOC_Os05g10330.1* | 927 | 94 | 172 | 5.480520893 | 11.03642068 | 1.00988743 | 2.33E-08 | 4.77E-07 |
| *LOC_Os01g72530.1* | 456 | 158 | 289 | 18.72690307 | 37.6974986 | 1.009356462 | 4.44E-13 | 1.48E-11 |
| *LOC_Os01g14990.1* | 528 | 218 | 398 | 22.31496908 | 44.83618818 | 1.006651823 | 2.22E-17 | 1.03E-15 |
| *LOC_Os02g09530.1* | 648 | 63 | 115 | 5.254595164 | 10.55607259 | 1.006421655 | 5.34E-06 | 7.42E-05 |
| *LOC_Os06g48180.2* | 693 | 288 | 525 | 22.46120085 | 45.06149566 | 1.004460138 | 2.36E-22 | 1.44E-20 |
| *LOC_Os07g48750.1* | 1245 | 325 | 591 | 14.10872368 | 28.23564176 | 1.00092994 | 7.92E-25 | 5.34E-23 |
| *LOC_Os06g37590.1* | 882 | 126 | 229 | 7.721037793 | 15.44352484 | 1.000135392 | 1.60E-10 | 4.22E-09 |
