# Supplementary material for: Transcriptome analysis of phosphorus stress responsiveness in the seedlings of Dongxiang wild rice (Oryza rufipogon Griff.)
Source: Biol Res. 2018 Mar 15;51:7. doi: 10.1186/s40659-018-0155-x (PMC5853122; doi:10.1186/s40659-018-0155-x)
Supplement: Supplementary file 7 — Additional file 7: Table S6. List of up-regulated genes both in the LLP vs. LCK and RLP vs. RCK. [file 40659_2018_155_MOESM7_ESM.docx]

| **Table S6**  List of up-regulated genes both in the LLP vs. LCK and RLP vs. RCK. | |
| --- | --- |
| Gene ID | Description |
| *LOC_Os01g27590* | transposon protein, putative, Pong sub-class, expressed |
| *LOC_Os01g03280* | hypothetical protein |
| *LOC_Os03g05334* | expressed protein |
| *LOC_Os01g52240* | chlorophyll A-B binding protein, putative, expressed |
| *LOC_Os02g48850* | plant-specific domain TIGR01615 family protein, expressed |
| *LOC_Os01g02460* | receptor kinase LRK10, putative, expressed |
| *LOC_Os06g21950* | inorganic phosphate transporter, putative, expressed |
| *LOC_Os01g71340* | glycosyl hydrolases family 17, putative, expressed |
| *LOC_Os02g22020* | MYB family transcription factor, putative, expressed |
| *LOC_Os11g43760* | lipase class 3 family protein, putative, expressed |
| *LOC_Os02g53240* | expressed protein |
| *LOC_Os08g20420* | MGD2, putative, expressed |
| *LOC_Os01g64120* | 2Fe-2S iron-sulfur cluster binding domain containing protein, expressed |
| *LOC_Os11g06980* | BURP domain containing protein, expressed |
| *LOC_Os01g52230* | phosphoethanolamine/phosphocholine phosphatase, putative, expressed |
| *LOC_Os05g15770* | glycosyl hydrolase, putative, expressed |
| *LOC_Os06g40120* | SPX domain containing protein, putative, expressed |
| *LOC_Os02g31030* | glycerophosphoryl diester phosphodiesterase family protein, putative, expressed |
| *LOC_Os12g36410* | transposon protein, putative, Pong sub-class, expressed |
| *LOC_Os03g40670* | glycerophosphoryl diester phosphodiesterase family protein, putative, expressed |
| *LOC_Os09g23300* | integral membrane protein, putative, expressed |
| *LOC_Os02g52730* | ferredoxin--nitrite reductase, putative, expressed |
| *LOC_Os08g06010* | transporter, major facilitator family, putative, expressed |
| *LOC_Os06g51060* | CHIT8 - Chitinase family protein precursor, expressed |
| *LOC_Os01g04920* | glycosyl transferase, group 1 domain containing protein, expressed |
| *LOC_Os04g55600* | expressed protein |
| *LOC_Os02g10780* | SPX domain containing protein, putative, expressed |
| *LOC_Os01g25484* | ferredoxin--nitrite reductase, putative, expressed |
| *LOC_Os03g04060* | CHIT16 - Chitinase family protein precursor, expressed |
| *LOC_Os05g46460* | hydrolase, alpha/beta fold family domain containing protein, expressed |
| *LOC_Os09g26670* | expressed protein |
| *LOC_Os08g31670* | transporter, putative, expressed |
| *LOC_Os10g39680* | CHIT14 - Chitinase family protein precursor, expressed |
| *LOC_Os05g02900* | expressed protein |
| *LOC_Os11g24630* | magnesium-dependent phosphatase 1, putative, expressed |
| *LOC_Os03g41330* | DUF260 domain containing protein, putative, expressed |
| *LOC_Os04g08390* | Leucine Rich Repeat family protein, expressed |
| *LOC_Os06g38760* | retrotransposon protein, putative, unclassified, expressed |
| *LOC_Os11g45990* | von Willebrand factor type A domain containing protein, putative, expressed |
| *LOC_Os10g04800* | expressed protein |
| *LOC_Os01g65830* | acyl-desaturase, chloroplast precursor, putative, expressed |
| *LOC_Os04g34600* | abscisic stress-ripening, putative, expressed |
| *LOC_Os01g36720* | transporter, major facilitator family, putative, expressed |
