# Supplementary material for: Transcriptome analysis of phosphorus stress responsiveness in the seedlings of Dongxiang wild rice (Oryza rufipogon Griff.)
Source: Biol Res. 2018 Mar 15;51:7. doi: 10.1186/s40659-018-0155-x (PMC5853122; doi:10.1186/s40659-018-0155-x)
Supplement: Supplementary file 8 — Additional file 8: Table S7. List of down-regulated genes both in the LLP vs. LCK and RLP vs. RCK. [file 40659_2018_155_MOESM8_ESM.docx]

| **Table S7** List of down-regulated genes both in the LLP vs. LCK and RLP vs. RCK. | |
| --- | --- |
| Gene ID | Description |
| *LOC_Os07g27030.1* | *OsFBX236* - F-box domain containing protein, expressed |
| *LOC_Os04g01710.1* | cysteine proteinase *At4g11310* precursor, putative, expressed |
| *LOC_Os02g43410.1* | transposon protein, putative, unclassified, expressed |
| *LOC_Os10g11889.1* | expressed protein |
| *LOC_Os11g15624.1* | expressed protein |
| *LOC_Os10g11889.2* | expressed protein |
| *LOC_Os02g43370.1* | transposon protein, putative, unclassified, expressed |
| *LOC_Os01g72370.3* | helix-loop-helix DNA-binding domain containing protein, expressed |
| *LOC_Os01g72360.1* | expressed protein |
| *LOC_Os07g15460.1* | metal transporter Nramp6, putative, expressed |
| *LOC_Os02g36140.2* | terpene synthase, putative, expressed |
| *LOC_Os07g03770.1* | Homeobox domain containing protein, expressed |
| *LOC_Os01g13610.1* | isoflavone reductase homolog IRL, putative, expressed |
| *LOC_Os01g62100.1* | retrotransposon protein, putative, unclassified, expressed |
| *LOC_Os08g05480.1* | *OsFBX261* - F-box domain containing protein, expressed |
| *LOC_Os01g41240.1* | hydrolase, alpha/beta fold family domain containing protein, expressed |
| *LOC_Os01g65100.1* | peptide transporter, putative, expressed |
| *LOC_Os03g59100.1* | pheophorbide a oxygenase, chloroplast precursor, putative, expressed |
| *LOC_Os12g12600.1* | dirigent, putative, expressed |
| *LOC_Os01g02770.1* | resistance-related receptor-like kinase, putative, expressed |
| *LOC_Os03g52680.1* | expressed protein |
| *LOC_Os03g57720.1* | aldehyde oxidase 2, putative, expressed |
| *LOC_Os01g02840.1* | resistance-related receptor-like kinase, putative, expressed |
| *LOC_Os02g53180.2* | 1-aminocyclopropane-1-carboxylate oxidase protein, putative, expressed |
| *LOC_Os01g52340.1* | NB-ARC domain containing protein, expressed |
| *LOC_Os01g09220.1* | transposon protein, putative, CACTA, En/Spm sub-class, expressed |
| *LOC_Os12g06480.1* | PHD-finger family protein, expressed |
| *LOC_Os01g64470.1* | harpin-induced protein 1 domain containing protein, expressed |
| *LOC_Os07g10840.1* | uncharacterized glycosyltransferase, putative, expressed |
| *LOC_Os12g06480.2* | PHD-finger family protein, expressed |
| *LOC_Os01g14650.1* | expansin precursor, putative, expressed |
| *LOC_Os04g56040.1* | glycine rich protein family protein, putative, expressed |
| *LOC_Os02g13290.1* | phosphoethanolamine/phosphocholine phosphatase, putative, expressed |
| *LOC_Os12g16490.1* | transposon protein, putative, unclassified, expressed |
| *LOC_Os01g72530.1* | *OsCML31* - Calmodulin-related calcium sensor protein, expressed |
