# Supplementary material for: Transcriptome analysis of phosphorus stress responsiveness in the seedlings of Dongxiang wild rice (Oryza rufipogon Griff.)
Source: Biol Res. 2018 Mar 15;51:7. doi: 10.1186/s40659-018-0155-x (PMC5853122; doi:10.1186/s40659-018-0155-x)
Supplement: Supplementary file 9 — Additional file 9: Table S8. List of down-regulated genes in the LLP vs. LCK but up-regulated in the RLP vs. RCK. [file 40659_2018_155_MOESM9_ESM.docx]

| **Table S8** List of down-regulated genes in the LLP vs. LCK but up-regulated in the RLP vs. RCK. | |
| --- | --- |
| Gene ID | Description |
| *LOC_Os07g08669.1* | expressed protein |
| *LOC_Os08g29809.1* | resistance protein LR10, putative, expressed |
| *LOC_Os05g45170.1* | glucosyl transferase, putative, expressed |
| *LOC_Os12g26850.1* | retrotransposon protein, putative, unclassified, expressed |
| *LOC_Os03g41170.1* | expressed protein |
| *LOC_Os01g61044.2* | transmembrane amino acid transporter protein, putative, expressed |
| *LOC_Os01g06900.1* | verticillium wilt disease resistance protein Ve2, putative, expressed |
| *LOC_Os11g30240.1* | expressed protein |
| *LOC_Os07g12810.1* | expressed protein |
| *LOC_Os11g09820.1* | expressed protein |
| *LOC_Os05g25650.1* | expressed protein |
| *LOC_Os08g34300.1* | retrotransposon protein, putative, unclassified, expressed |
| *LOC_Os07g46060.1* | expressed protein |
| *LOC_Os10g18820.1* | dirigent, putative, expressed |
| *LOC_Os07g10230.1* | glucosyltransferase, putative, expressed |
| *LOC_Os10g09990.1* | cytokinin-O-glucosyltransferase 3, putative, expressed |
| *LOC_Os12g16340.1* | retrotransposon protein, putative, unclassified, expressed |
| *LOC_Os04g09390.1* | HEV3 - Hevein family protein precursor, expressed |
| *LOC_Os12g31200.1* | NB-ARC domain containing protein, expressed |
| *LOC_Os12g36220.1* | inhibitor I family protein, putative, expressed |
| *LOC_Os12g31160.1* | *MLA10*, putative, expressed |
| *LOC_Os10g38340.1* | glutathione S-transferase GSTU6, putative, expressed |
| *LOC_Os10g34930.1* | secretory protein, putative, expressed |
| *LOC_Os12g36880.1* | pathogenesis-related Bet v I family protein, putative, expressed |
| *LOC_Os06g31800.1* | *THION2* - Plant thionin family protein precursor, expressed |
| *LOC_Os01g03340.1* | *BBTI4* - Bowman-Birk type bran trypsin inhibitor precursor, expressed |
| *LOC_Os04g27060.1* | oxidoreductase, aldo/keto reductase family protein, putative, expressed |
| *LOC_Os06g48020.1* | peroxidase precursor, putative, expressed |
| *LOC_Os04g45810.1* | homeobox associated leucine zipper, putative, expressed |
| *LOC_Os11g05360.1* | *RCLEA9* - Root cap and Late embryogenesis related family protein precursor, putative, expressed |
| *LOC_Os01g43851.1* | cytochrome P450 72A1, putative, expressed |
| *LOC_Os04g11030.1* | expressed protein |
| *LOC_Os12g36210.1* | inhibitor I family protein, putative, expressed |
| *LOC_Os07g08160.1* | early light-induced protein, chloroplast precursor, putative, expressed |
| *LOC_Os02g54900.1* | *STE_PAK_Ste20_STLK.2 - STE* kinases include homologs to sterile 7, sterile 11 and sterile 20 from yeast, expressed |
| *LOC_Os12g36240.1* | inhibitor I family protein, putative, expressed |
| *LOC_Os08g20130.1* | flavonol sulfotransferase, putative, expressed |
| *LOC_Os05g29735.1* | expressed protein |
