# Supplementary material for: Transcriptome analysis of phosphorus stress responsiveness in the seedlings of Dongxiang wild rice (Oryza rufipogon Griff.)
Source: Biol Res. 2018 Mar 15;51:7. doi: 10.1186/s40659-018-0155-x (PMC5853122; doi:10.1186/s40659-018-0155-x)
Supplement: Supplementary file 10 — Additional file 10: Table S9. Significant GO terms of DEGs in the biological process, cellular component and molecular function category for LLP vs. LCK. [file 40659_2018_155_MOESM10_ESM.docx]

**Table S9** Significant GO terms of DEGs in the biological process, cellular component and molecular function category for LLP vs. LCK.

| GO term | Category | GO term annotation | *P*-value |
| --- | --- | --- | --- |
| GO:0080027 | biological process | [response to herbivore](http://amigo.geneontology.org/cgi-bin/amigo/go.cgi?action=query&view=query&query=GO:0080027&search_constraint=terms) | 2.37e-05 |
| GO:0055114 | biological process | oxidation-reduction process | 0.02592 |
| GO:0019755 | biological process | one-carbon compound transport | 0.04803 |
| GO:0016023 | cellular component | cytoplasmic membrane-bounded vesicle | 2.03e-15 |
| GO:0031410 | cellular component | cytoplasmic vesicle | 2.06e-15 |
| GO:0031988 | cellular component | membrane-bounded vesicle | 2.57e-15 |
| GO:0031982 | cellular component | vesicle | 2.85e-15 |
| GO:0043231 | cellular component | intracellular membrane-bounded organelle | 0.00129 |
| GO:0043227 | cellular component | membrane-bounded organelle | 0.00131 |
| GO:0005576 | cellular component | extracellular region | 0.01215 |
| GO:0016165 | molecular function | lipoxygenase activity | 3.03e-05 |
| GO:0016701 | molecular function | oxidoreductase activity, acting on single donors with incorporation of molecular oxygen | 0.00119 |
| GO:0042887 | molecular function | amide transmembrane transporter activity | 0.00133 |
| GO:0016491 | molecular function | oxidoreductase activity | 0.00237 |
| GO:0016702 | molecular function | oxidoreductase activity, acting on single donors with incorporation of molecular oxygen, incorporation of two atoms of oxygen | 0.00442 |
| GO:0004556 | molecular function | alpha-amylase activity | 0.00505 |
| GO:0005506 | molecular function | iron ion binding | 0.00516 |
| GO:0033926 | molecular function | glycopeptide alpha-N-acetylgalactosaminidase activity | 0.00690 |
| GO:0004867 | molecular function | serine-type endopeptidase inhibitor activity | 0.02722 |
| GO:0015204 | molecular function | urea transmembrane transporter activity | 0.03524 |
| GO:0051213 | molecular function | dioxygenase activity | 0.04076 |
