# Supplementary material for: Transcriptome analysis of phosphorus stress responsiveness in the seedlings of Dongxiang wild rice (Oryza rufipogon Griff.)
Source: Biol Res. 2018 Mar 15;51:7. doi: 10.1186/s40659-018-0155-x (PMC5853122; doi:10.1186/s40659-018-0155-x)
Supplement: Supplementary file 11 — Additional file 11: Table S10. Significant GO terms of DEGs in the biological process, cellular component and molecular function category for RLP vs. RCK. [file 40659_2018_155_MOESM11_ESM.docx]

**Table S10**  Significant GO terms of DEGs in the biological process, cellular component and molecular function category for RLP vs. RCK.

| GO term | Category | GO term annotation | *P*-value |
| --- | --- | --- | --- |
| GO:0015979 | biological process | photosynthesis | 1.68e-52 |
| GO:0006412 | biological process | translation | 4.83e-49 |
| GO:0019684 | biological process | photosynthesis, light reaction | 7.03e-33 |
| GO:0009765 | biological process | photosynthesis, light harvesting | 1.69e-30 |
| GO:0006091 | biological process | generation of precursor metabolites and energy | 5.63e-25 |
| GO:0010467 | biological process | gene expression | 3.07e-24 |
| GO:0034645 | biological process | cellular macromolecule biosynthetic process | 9.30e-20 |
| GO:0010114 | biological process | response to red light | 2.18e-19 |
| GO:0010218 | biological process | response to far red light | 6.30e-19 |
| GO:0009059 | biological process | macromolecule biosynthetic process | 1.41e-18 |
| GO:1901576 | biological process | organic substance biosynthetic process | 7.88e-16 |
| GO:0044249 | biological process | cellular biosynthetic process | 1.03e-15 |
| GO:0009058 | biological process | biosynthetic process | 3.03e-15 |
| GO:0009637 | biological process | response to blue light | 5.62e-14 |
| GO:0042254 | biological process | ribosome biogenesis | 3.98e-13 |
| GO:0009639 | biological process | response to red or far red light | 6.82e-13 |
| GO:0022613 | biological process | ribonucleoprotein complex biogenesis | 7.77e-13 |
| GO:0009416 | biological process | response to light stimulus | 3.20e-09 |
| GO:0006364 | biological process | rRNA processing | 5.67e-08 |
| GO:0009314 | biological process | response to radiation | 9.58e-08 |
| GO:0016072 | biological process | rRNA metabolic process | 1.19e-07 |
| GO:0010207 | biological process | photosystem II assembly | 1.48e-07 |
| GO:0009628 | biological process | response to abiotic stimulus | 3.38e-07 |
| GO:0006817 | biological process | phosphate ion transport | 1.11e-05 |
| GO:0010038 | biological process | response to metal ion | 1.12e-05 |
| GO:0015977 | biological process | carbon fixation | 1.34e-05 |
| GO:0022900 | biological process | electron transport chain | 3.82e-05 |
| GO:0044085 | biological process | cellular component biogenesis | 6.23e-05 |
| GO:0009773 | biological process | photosynthetic electron transport in photosystem I | 6.50e-05 |
| GO:0016036 | biological process | cellular response to phosphate starvation | 9.51e-05 |
| GO:0055080 | biological process | cation homeostasis | 0.00015 |
| GO:0019253 | biological process | reductive pentose-phosphate cycle | 0.00030 |
| GO:0050801 | biological process | ion homeostasis | 0.00034 |
| GO:0034470 | biological process | ncRNA processing | 0.00038 |
| GO:0019685 | biological process | photosynthesis, dark reaction | 0.00042 |
| GO:0044237 | biological process | cellular metabolic process | 0.00132 |
| GO:0048878 | biological process | chemical homeostasis | 0.00153 |
| GO:0080167 | biological process | response to karrikin | 0.00196 |
| GO:0019344 | biological process | cysteine biosynthetic process | 0.00239 |
| GO:0009267 | biological process | cellular response to starvation | 0.00304 |
| GO:0006971 | biological process | hypotonic response | 0.00315 |
| GO:0042539 | biological process | hypotonic salinity response | 0.00315 |
| GO:0006534 | biological process | cysteine metabolic process | 0.00359 |
| GO:0042594 | biological process | response to starvation | 0.00375 |
| GO:0009767 | biological process | photosynthetic electron transport chain | 0.00480 |
| GO:0050896 | biological process | response to stimulus | 0.00498 |
| GO:0005513 | biological process | detection of calcium ion | 0.00502 |
| GO:0006950 | biological process | response to stress | 0.00503 |
| GO:0031669 | biological process | cellular response to nutrient levels | 0.00589 |
| GO:0009069 | biological process | serine family amino acid metabolic process | 0.00664 |
| GO:0009070 | biological process | serine family amino acid biosynthetic process | 0.00896 |
| GO:0006873 | biological process | cellular ion homeostasis | 0.01038 |
| GO:0009409 | biological process | response to cold | 0.01224 |
| GO:0034622 | biological process | cellular macromolecular complex assembly | 0.01372 |
| GO:0044267 | biological process | cellular protein metabolic process | 0.01774 |
| GO:0035304 | biological process | regulation of protein dephosphorylation | 0.01971 |
| GO:0006414 | biological process | translational elongation | 0.02136 |
| GO:0051592 | biological process | response to calcium ion | 0.02712 |
| GO:0030003 | biological process | cellular cation homeostasis | 0.02717 |
| GO:0043434 | biological process | response to peptide hormone stimulus | 0.03160 |
| GO:0060416 | biological process | response to growth hormone stimulus | 0.03160 |
| GO:1901652 | biological process | response to peptide | 0.03160 |
| GO:0055082 | biological process | cellular chemical homeostasis | 0.03593 |
| GO:0035303 | biological process | regulation of dephosphorylation | 0.03676 |
| GO:0031668 | biological process | cellular response to extracellular stimulus | 0.04502 |
| GO:0071496 | biological process | cellular response to external stimulus | 0.04502 |
| GO:0010035 | biological process | response to inorganic substance | 0.04641 |
| GO:0005840 | cellular component | ribosome | 1.96e-56 |
| GO:0009579 | cellular component | thylakoid | 5.30e-53 |
| GO:0030529 | cellular component | ribonucleoprotein complex | 5.58e-51 |
| GO:0034357 | cellular component | photosynthetic membrane | 2.26e-50 |
| GO:0044436 | cellular component | thylakoid part | 1.44e-48 |
| GO:0022626 | cellular component | cytosolic ribosome | 7.52e-47 |
| GO:0044445 | cellular component | cytosolic part | 1.03e-44 |
| GO:0009534 | cellular component | chloroplast thylakoid | 2.81e-42 |
| GO:0031976 | cellular component | plastid thylakoid | 2.81e-42 |
| GO:0042651 | cellular component | thylakoid membrane | 3.38e-42 |
| GO:0009535 | cellular component | chloroplast thylakoid membrane | 4.95e-42 |
| GO:0055035 | cellular component | plastid thylakoid membrane | 5.96e-42 |
| GO:0031984 | cellular component | organelle subcompartment | 2.48e-41 |
| GO:0044391 | cellular component | ribosomal subunit | 7.60e-38 |
| GO:0043228 | cellular component | non-membrane-bounded organelle | 9.15e-38 |
| GO:0043232 | cellular component | intracellular non-membrane-bounded organelle | 9.15e-38 |
| GO:0032991 | cellular component | macromolecular complex | 3.68e-37 |
| GO:0009521 | cellular component | photosystem | 2.00e-31 |
| GO:0009507 | cellular component | chloroplast | 3.97e-28 |
| GO:0009522 | cellular component | photosystem I | 1.66e-26 |
| GO:0044446 | cellular component | intracellular organelle part | 4.31e-26 |
| GO:0044422 | cellular component | organelle part | 5.76e-26 |
| GO:0022625 | cellular component | cytosolic large ribosomal subunit | 6.70e-25 |
| GO:0015934 | cellular component | large ribosomal subunit | 2.89e-22 |
| GO:0010287 | cellular component | plastoglobule | 3.27e-21 |
| GO:0005730 | cellular component | nucleolus | 3.85e-21 |
| GO:0044434 | cellular component | chloroplast part | 2.61e-20 |
| GO:0005829 | cellular component | cytosol | 4.55e-20 |
| GO:0044435 | cellular component | plastid part | 9.78e-20 |
| GO:0044444 | cellular component | cytoplasmic part | 1.08e-18 |
| GO:0005737 | cellular component | cytoplasm | 5.32e-18 |
| GO:0009526 | cellular component | plastid envelope | 1.60e-17 |
| GO:0009941 | cellular component | chloroplast envelope | 1.47e-16 |
| GO:0009536 | cellular component | plastid | 1.20e-14 |
| GO:0031967 | cellular component | organelle envelope | 1.57e-14 |
| GO:0015935 | cellular component | small ribosomal subunit | 4.08e-14 |
| GO:0005618 | cellular component | cell wall | 8.13e-14 |
| GO:0031975 | cellular component | envelope | 1.19e-13 |
| GO:0009523 | cellular component | photosystem II | 4.64e-13 |
| GO:0030312 | cellular component | external encapsulating structure | 1.05e-12 |
| GO:0022627 | cellular component | cytosolic small ribosomal subunit | 1.23e-12 |
| GO:0070013 | cellular component | intracellular organelle lumen | 1.93e-12 |
| GO:0043233 | cellular component | organelle lumen | 1.98e-12 |
| GO:0009532 | cellular component | plastid stroma | 2.82e-12 |
| GO:0009570 | cellular component | chloroplast stroma | 3.56e-12 |
| GO:0031981 | cellular component | nuclear lumen | 3.77e-12 |
| GO:0031974 | cellular component | membrane-enclosed lumen | 4.01e-12 |
| GO:0044428 | cellular component | nuclear part | 1.21e-10 |
| GO:0044424 | cellular component | intracellular part | 4.89e-08 |
| GO:0009538 | cellular component | photosystem I reaction center | 5.00e-08 |
| GO:0016020 | cellular component | membrane | 1.97e-07 |
| GO:0005773 | cellular component | vacuole | 2.02e-07 |
| GO:0048046 | cellular component | apoplast | 2.35e-07 |
| GO:0005622 | cellular component | intracellular | 2.43e-07 |
| GO:0009573 | cellular component | chloroplast ribulose bisphosphate carboxylase complex | 3.16e-07 |
| GO:0048492 | cellular component | ribulose bisphosphate carboxylase complex | 3.16e-07 |
| GO:0031977 | cellular component | thylakoid lumen | 6.98e-07 |
| GO:0005576 | cellular component | extracellular region | 3.46e-06 |
| GO:0043229 | cellular component | intracellular organelle | 4.72e-05 |
| GO:0043226 | cellular component | organelle | 4.84e-05 |
| GO:0005623 | cellular component | cell | 5.22e-05 |
| GO:0044464 | cellular component | cell part | 5.22e-05 |
| GO:0005884 | cellular component | actin filament | 0.00088 |
| GO:0009654 | cellular component | oxygen evolving complex | 0.00524 |
| GO:0030093 | cellular component | chloroplast photosystem I | 0.01152 |
| GO:0010319 | cellular component | stromule | 0.01156 |
| GO:0071944 | cellular component | cell periphery | 0.01232 |
| GO:0015629 | cellular component | actin cytoskeleton | 0.02364 |
| GO:0030684 | cellular component | preribosome | 0.02984 |
| GO:0030076 | cellular component | light-harvesting complex | 0.04715 |
| GO:0005198 | molecular function | structural molecule activity | 3.90e-61 |
| GO:0003735 | molecular function | structural constituent of ribosome | 8.16e-60 |
| GO:0016168 | molecular function | chlorophyll binding | 4.49e-13 |
| GO:0016984 | molecular function | ribulose-bisphosphate carboxylase activity | 2.11e-08 |
| GO:0046906 | molecular function | tetrapyrrole binding | 6.43e-06 |
| GO:0008187 | molecular function | poly-pyrimidine tract binding | 5.36e-05 |
| GO:0008266 | molecular function | poly(U) RNA binding | 5.36e-05 |
| GO:0003727 | molecular function | single-stranded RNA binding | 0.00070 |
| GO:0005507 | molecular function | copper ion binding | 0.00180 |
| GO:0005381 | molecular function | iron ion transmembrane transporter activity | 0.00534 |
| GO:0015603 | molecular function | iron chelate transmembrane transporter activity | 0.00548 |
| GO:0051980 | molecular function | iron-nicotianamine transmembrane transporter activity | 0.00548 |
| GO:0005315 | molecular function | inorganic phosphate transmembrane transporter activity | 0.00711 |
| GO:0004364 | molecular function | glutathione transferase activity | 0.00754 |
| GO:0003723 | molecular function | RNA binding | 0.01050 |
| GO:0016831 | molecular function | carboxy-lyase activity | 0.01424 |
| GO:0015293 | molecular function | symporter activity | 0.03754 |
| GO:0016830 | molecular function | carbon-carbon lyase activity | 0.03794 |
