# Supplementary material for: Transcriptome analysis of phosphorus stress responsiveness in the seedlings of Dongxiang wild rice (Oryza rufipogon Griff.)
Source: Biol Res. 2018 Mar 15;51:7. doi: 10.1186/s40659-018-0155-x (PMC5853122; doi:10.1186/s40659-018-0155-x)
Supplement: Supplementary file 12 — Additional file 12: Table S11. Significant KO terms of DEGs in the LLP vs. LCK (Q-value < 0.05). [file 40659_2018_155_MOESM12_ESM.docx]

**Table S11** Significant KO terms of DEGs in the LLP vs. LCK (*Q*-value < 0.05).

| KO term | KO annotation | *P*-value | *Q*-value |
| --- | --- | --- | --- |
| KO00902 | Monoterpenoid biosynthesis | 1.71E-11 | 1.95E-09 |
| KO03013 | RNA transport | 1.28E-06 | 7.32E-05 |
| KO03015 | mRNA surveillance pathway | 1.10E-05 | 4.19E-04 |
| KO01110 | Biosynthesis of secondary metabolites | 1.89E-05 | 5.38E-04 |
| KO00591 | Linoleic acid metabolism | 2.47E-05 | 5.63E-04 |
| KO00944 | Flavone and flavonol biosynthesis | 0.000171264 | 3.05E-03 |
| KO00945 | Stilbenoid, diarylheptanoid and gingerol biosynthesis | 0.000187365 | 3.05E-03 |
| KO04626 | Plant-pathogen interaction | 0.000231564 | 3.30E-03 |
| KO00909 | Sesquiterpenoid and triterpenoid biosynthesis | 0.000505311 | 6.40E-03 |
| KO00592 | alpha-Linolenic acid metabolism | 0.000597596 | 6.81E-03 |
| KO00906 | Carotenoid biosynthesis | 0.00070709 | 7.19E-03 |
| KO00402 | Benzoxazinoid biosynthesis | 0.000756355 | 7.19E-03 |
| KO00350 | Tyrosine metabolism | 0.002294794 | 2.01E-02 |
| KO00941 | Flavonoid biosynthesis | 0.002577536 | 2.10E-02 |
| KO00360 | Phenylalanine metabolism | 0.005223549 | 3.86E-02 |
| KO01100 | Metabolic pathways | 0.005412373 | 3.86E-02 |
