# Supplementary material for: Transcriptome analysis of phosphorus stress responsiveness in the seedlings of Dongxiang wild rice (Oryza rufipogon Griff.)
Source: Biol Res. 2018 Mar 15;51:7. doi: 10.1186/s40659-018-0155-x (PMC5853122; doi:10.1186/s40659-018-0155-x)
Supplement: Supplementary file 13 — Additional file 13: Table S12. Significant KO terms of DEGs in the RLP vs. RCK (Q-value < 0.05). [file 40659_2018_155_MOESM13_ESM.docx]

**Table S12**  Significant KO terms of DEGs in the RLP vs. RCK (Q-value < 0.05).

| KO term | KO annotation | *P*-value | *Q*-value |
| --- | --- | --- | --- |
| KO03010 | Ribosome | 1.43E-48 | 1.67E-46 |
| KO00196 | Photosynthesis - antenna proteins | 6.86E-35 | 4.01E-33 |
| KO00195 | Photosynthesis | 4.60E-34 | 1.80E-32 |
| KO04145 | Phagosome | 9.08E-12 | 2.65E-10 |
| KO00710 | Carbon fixation in photosynthetic organisms | 1.54E-08 | 3.20E-07 |
| KO00630 | Glyoxylate and dicarboxylate metabolism | 1.64E-08 | 3.20E-07 |
| KO00910 | Nitrogen metabolism | 1.30E-05 | 2.17E-04 |
