# Supplementary material for: Transcriptome analysis of phosphorus stress responsiveness in the seedlings of Dongxiang wild rice (Oryza rufipogon Griff.)
Source: Biol Res. 2018 Mar 15;51:7. doi: 10.1186/s40659-018-0155-x (PMC5853122; doi:10.1186/s40659-018-0155-x)
Supplement: Supplementary file 14 — Additional file 14: Table S13. Previously identified P-deficiency responses related QTL intervals. [file 40659_2018_155_MOESM14_ESM.docx]

| **Table S13** Previously identified P-deficiency responses related QTL intervals. | | | | | | | |
| --- | --- | --- | --- | --- | --- | --- | --- |
| QTL_accession_ID | Chromosome | Interval | Position (bp) | Sart_position | | Stop_position | Map_units |
| AQCI006 | 4 | E21087S-C946 | 186,806-4,439,573 | 0 | 10.7 | | cM |
| AQCI011 | 4 | C1399-S733 | 24,471,340-27,866,310 | 76.5 | 87.1 | | cM |
| AQCI009 | 6 | C425-R2349 | 1,644,474-5,894,449 | 10.7 | 19.1 | | cM |
| AQCI013 | 12 | E60377-C443 | 5,115,486-12,239,912 | 47 | 64.7 | | cM |
| AQCI003 | 10 | R2194-R2447 | 7,831,094-14,271,753 | 15.7 | 30.2 | | cM |
| AQCI002 | 6 | C226-R2349 | 3,536,517-5,894,449 | 13.5 | 19.1 | | cM |
| AQCI005 | 2 | S14115-C12706S | 8,730,165-10,538,504 | 42.1 | 62.2 | | cM |
| AQBD003 | 12 | RG241-RZ76 | 1,548,040-20,014,601 | 17.1 | 33.3 | | cM |
| AQCI012 | 12 | C1069-RG181 | 25,047,073-27,489,485 | 97.3 | 109.5 | | cM |
| AQCI008 | 3 | C169-C2184 | 6,753,756-10,394 ,582 | 31.3 | 46.6 | | cM |
