# Supplementary material for: Transcriptome analysis of phosphorus stress responsiveness in the seedlings of Dongxiang wild rice (Oryza rufipogon Griff.)
Source: Biol Res. 2018 Mar 15;51:7. doi: 10.1186/s40659-018-0155-x (PMC5853122; doi:10.1186/s40659-018-0155-x)
Supplement: Supplementary file 15 — Additional file 15: Table S14. DEGs mapped to previously identified P-deficiency responses related QTL intervals. [file 40659_2018_155_MOESM15_ESM.docx]

| **Table S14** DEGs mapped to previously identified P-deficiency responses related QTL intervals. | |
| --- | --- |
| Gene ID | Putative Function |
| **AQBD003** (86 genes) | |
| *LOC_Os12g12600.1* | dirigent, putative, expressed |
| *LOC_Os12g12290.1* | exostosin family domain containing protein, expressed |
| *LOC_Os12g31160.1* | MLA10, putative, expressed |
| *LOC_Os12g05990.1* | no apical meristem protein, putative, expressed |
| *LOC_Os12g28710.1* | ATPase 3, putative, expressed |
| *LOC_Os12g31200.1* | NB-ARC domain containing protein, expressed |
| *LOC_Os12g25450.1* | O-methyltransferase, putative, expressed |
| *LOC_Os12g17430.1* | NBS-LRR disease resistance protein, putative, expressed |
| *LOC_Os12g28177.1* | ATPase 3, putative, expressed |
| *LOC_Os12g08025.1* | expressed protein |
| *LOC_Os12g05180.1* | avr9/Cf-9 rapidly elicited protein, putative, expressed |
| *LOC_Os12g17160.1* | flavonol sulfotransferase, putative, expressed |
| *LOC_Os12g16520.1* | wall-associated receptor kinase 3 precursor, putative, expressed |
| *LOC_Os12g16340.1* | retrotransposon protein, putative, unclassified, expressed |
| *LOC_Os12g06480.1* | PHD-finger family protein, expressed |
| *LOC_Os12g06480.2* | PHD-finger family protein, expressed |
| *LOC_Os12g26850.1* | retrotransposon protein, putative, unclassified, expressed |
| *LOC_Os12g06870.1* | OsNucAP2 - Putative Nucleoporin Autopeptidase homologue, expressed |
| *LOC_Os12g22145.1* | expressed protein |
| *LOC_Os12g15680.1* | laccase precursor protein, putative, expressed |
| *LOC_Os12g06464.1* | expressed protein |
| *LOC_Os12g08220.1* | histone deacetylase, putative, expressed |
| *LOC_Os12g09130.1* | expressed protein |
| *LOC_Os12g18080.1* | retrotransposon protein, putative, unclassified, expressed |
| *LOC_Os12g16540.1* | OsWAK124 - OsWAK receptor-like protein OsWAK-RLP, expressed |
| *LOC_Os12g16490.1* | transposon protein, putative, unclassified, expressed |
| *LOC_Os12g06910.2* | nucleolar protein family 6, putative, expressed |
| *LOC_Os12g17410.1* | NB-ARC domain containing protein, expressed |
| *LOC_Os12g30180.1* | protein kinase domain containing protein, expressed |
| *LOC_Os12g25200.1* | chloride transporter, chloride channel family, putative, expressed |
| *LOC_Os12g09700.1* | Jacalin-like lectin domain containing protein, putative, expressed |
| *LOC_Os12g29710.1* | NBS-LRR disease resistance protein, putative, expressed |
| *LOC_Os12g06150.1* | DUF623 domain containing protein, expressed |
| *LOC_Os12g04880.1* | retrotransposon protein, putative, unclassified, expressed |
| *LOC_Os12g03870.1* | major facilitator superfamily antiporter, putative, expressed |
| *LOC_Os12g29690.1* | NBS-LRR disease resistance protein, putative, expressed |
| *LOC_Os12g07160.2* | plant protein of unknown function DUF869 domain containing protein, expressed |
| *LOC_Os12g12990.1* | transposon protein, putative, CACTA, En/Spm sub-class, expressed |
| *LOC_Os12g32400.1* | helix-loop-helix DNA-binding domain containing protein, expressed |
| *LOC_Os12g21720.1* | retrotransposon protein, putative, unclassified, expressed |
| *LOC_Os12g14330.1* | disease resistance protein RPM1, putative, expressed |
| *LOC_Os12g31729.1* | transposon protein, putative, unclassified, expressed |
| *LOC_Os12g32480.1* | expressed protein |
| *LOC_Os12g04350.1* | sulfotransferase domain containing protein, expressed |
| *LOC_Os12g05310.1* | retrotransposon protein, putative, unclassified, expressed |
| *LOC_Os12g25490.1* | O-methyltransferase, putative, expressed |
| *LOC_Os12g23520.1* | retrotransposon protein, putative, Ty3-gypsy subclass, expressed |
| *LOC_Os12g16480.1* | expressed protein |
| *LOC_Os12g12720.1* | jasmonate-induced protein, putative, expressed |
| *LOC_Os12g13730.1* | pleiotropic drug resistance protein 2, putative, expressed |
| *LOC_Os12g30760.1* | disease resistance protein, putative, expressed |
| *LOC_Os12g10330.1* | NB-ARC domain containing protein, expressed |
| *LOC_Os12g10340.1* | NBS-LRR type resistance protein, putative, expressed |
| *LOC_Os12g10410.1* | NB-ARC/LRR disease resistance protein, putative, expressed |
| *LOC_Os12g32900.1* | plant protein of unknown function domain containing protein, expressed |
| *LOC_Os12g09540.1* | phosphoribosylamine--glycine ligase, putative, expressed |
| *LOC_Os12g23980.1* | OsSub63 - Putative Subtilisin homologue, expressed |
| *LOC_Os12g23200.1* | photosystem I reaction center subunit XI, chloroplast precursor, putative, expressed |
| *LOC_Os12g08770.1* | photosystem I reaction center subunit N, chloroplast precursor, putative, expressed |
| *LOC_Os12g32850.1* | cytochrome P450, putative, expressed |
| *LOC_Os12g31460.1* | heat shock protein DnaJ, putative, expressed |
| *LOC_Os12g19470.2* | ribulose bisphosphate carboxylase small chain, chloroplast precursor, putative, expressed |
| *LOC_Os12g19381.1* | ribulose bisphosphate carboxylase small chain, chloroplast precursor, putative, expressed |
| *LOC_Os12g23180.1* | 3-beta hydroxysteroid dehydrogenase/isomerase family protein, putative, expressed |
| *LOC_Os12g12120.1* | verticillium wilt disease resistance protein precursor, putative, expressed |
| *LOC_Os12g15314.1* | staphylococcal nuclease homologue, putative, expressed |
| *LOC_Os12g31410.1* | expressed protein |
| *LOC_Os12g14580.1* | tetraspanin family protein, putative, expressed |
| *LOC_Os12g26380.1* | dirigent, putative, expressed |
| *LOC_Os12g08730.1* | thioredoxin, putative, expressed |
| *LOC_Os12g13470.1* | hypothetical protein |
| *LOC_Os12g14699.1* | protein kinase domain containing protein, expressed |
| *LOC_Os12g26290.1* | alpha-DOX2, putative, expressed |
| *LOC_Os12g12130.1* | verticillium wilt disease resistance protein, putative, expressed |
| *LOC_Os12g31660.1* | transposon protein, putative, CACTA, En/Spm sub-class, expressed |
| *LOC_Os12g22284.1* | white-brown complex homolog protein 11, putative, expressed |
| *LOC_Os12g08760.1* | carboxyvinyl-carboxyphosphonate phosphorylmutase, putative, expressed |
| *LOC_Os12g09620.1* | expressed protein |
| *LOC_Os12g05440.1* | cytochrome P450, putative, expressed |
| *LOC_Os12g17600.1* | ribulose bisphosphate carboxylase small chain, chloroplast precursor, putative, expressed |
| *LOC_Os12g07830.1* | OsAPx5 - Stromal Ascorbate Peroxidase encoding gene 5,8, expressed |
| *LOC_Os12g06190.1* | expressed protein |
| *LOC_Os12g16080.1* | expressed protein |
| *LOC_Os12g08270.1* | inositol-1-monophosphatase, putative, expressed |
| *LOC_Os12g28770.1* | POEI19 - Pollen Ole e I allergen and extensin family protein precursor, expressed |
| *LOC_Os12g29340.1* | expressed protein |
| **AQCI002** (16 genes) | |
| *LOC_Os06g09610.1* | peroxiredoxin, putative, expressed |
| *LOC_Os06g10830.1* | retrotransposon protein, putative, unclassified, expressed |
| *LOC_Os06g10870.1* | retrotransposon protein, putative, unclassified, expressed |
| *LOC_Os06g10930.1* | xyloglucan fucosyltransferase, putative, expressed |
| *LOC_Os06g08640.1* | transferase family protein, putative, expressed |
| *LOC_Os06g09310.1* | zinc finger, C3HC4 type domain containing protein, expressed |
| *LOC_Os06g10300.1* | OsFBL29 - F-box domain and LRR containing protein, expressed |
| *LOC_Os06g09220.1* | cytochrome P450 72A1, putative, expressed |
| *LOC_Os06g08710.1* | receptor-like protein kinase precursor, putative, expressed |
| *LOC_Os06g11150.1* | DUF1645 domain containing protein, putative, expressed |
| *LOC_Os06g07760.1* | sulfiredoxin-1, putative, expressed |
| *LOC_Os06g10820.1* | helix-loop-helix DNA-binding domain containing protein, expressed |
| *LOC_Os06g08580.1* | transferase family protein, putative, expressed |
| *LOC_Os06g09600.1* | expressed protein |
| *LOC_Os06g10590.1* | expressed protein |
| *LOC_Os06g07790.1* | retrotransposon protein, putative, unclassified, expressed |
| **AQCI003** (20 genes) | |
| *LOC_Os10g20610.1* | laccase-15 precursor, putative, expressed |
| *LOC_Os10g20440.1* | retrotransposon protein, putative, unclassified, expressed |
| *LOC_Os10g15300.1* | expressed protein |
| *LOC_Os10g25430.1* | Ser/Thr protein phosphatase family protein, putative, expressed |
| *LOC_Os10g18820.1* | dirigent, putative, expressed |
| *LOC_Os10g23900.1* | decarboxylase, putative, expressed |
| *LOC_Os10g26700.1* | YGL010w, putative, expressed |
| *LOC_Os10g17489.1* | UDP-glucoronosyl and UDP-glucosyl transferase domain containing protein, expressed |
| *LOC_Os10g25000.1* | expressed protein |
| *LOC_Os10g26340.1* | cytochrome P450, putative, expressed |
| *LOC_Os10g21090.1* | ATP binding protein, putative, expressed |
| *LOC_Os10g20390.1* | MATE efflux family protein, putative, expressed |
| *LOC_Os10g20380.1* | uncharacterized transporter C11D3.06, putative, expressed |
| *LOC_Os10g27040.1* | retrotransposon protein, putative, unclassified, expressed |
| *LOC_Os10g25030.1* | red chlorophyll catabolite reductase, putative, expressed |
| *LOC_Os10g18370.1* | transcriptional regulator, putative, expressed |
| *LOC_Os10g25040.2* | red chlorophyll catabolite reductase, putative, expressed |
| *LOC_Os10g26410.1* | bHLH family protein, putative, expressed |
| *LOC_Os10g19960.1* | beta-galactosidase, putative, expressed |
| *LOC_Os10g20890.1* | LTPL137 - Protease inhibitor/seed storage/LTP family protein precursor, expressed |
| **AQCI005** (9 genes) | |
| *LOC_Os02g17240.1* | ATROPGEF7/ROPGEF7, putative, expressed |
| *LOC_Os02g15870.3* | molybdenum cofactor biosynthesis protein 1, putative, expressed |
| *LOC_Os02g15560.1* | transposon protein, putative, Mutator sub-class, expressed |
| *LOC_Os02g16940.1* | OsSub13 - Putative Subtilisin homologue, expressed |
| *LOC_Os02g17760.1* | cytochrome P450, putative, expressed |
| *LOC_Os02g15750.1* | expressed protein |
| *LOC_Os02g18080.1* | NB-ARC domain containing protein, expressed |
| *LOC_Os02g16800.1* | expansin precursor, putative, expressed |
| *LOC_Os02g17060.1* | OsSub15 - Putative Subtilisin homologue, expressed |
| **AQCI006** (19 genes) | |
| *LOC_Os04g03790.1* | retrotransposon protein, putative, unclassified, expressed |
| *LOC_Os04g01810.1* | terpene synthase, putative, expressed |
| *LOC_Os04g01740.1* | heat shock protein, putative, expressed |
| *LOC_Os04g05520.1* | transposon protein, putative, unclassified, expressed |
| *LOC_Os04g04390.1* | retrotransposon protein, putative, unclassified, expressed |
| *LOC_Os04g01710.1* | cysteine proteinase At4g11310 precursor, putative, expressed |
| *LOC_Os04g05650.1* | expressed protein |
| *LOC_Os04g02850.1* | pentatricopeptide, putative, expressed |
| *LOC_Os04g02530.1* | expressed protein |
| *LOC_Os04g02040.1* | NBS-LRR, putative, expressed |
| *LOC_Os04g02450.1* | rust-resistance protein Lr21, putative, expressed |
| *LOC_Os04g07980.1* | transposon protein, putative, CACTA, En/Spm sub-class, expressed |
| *LOC_Os04g01320.1* | serine/threonine-protein kinase receptor precursor, putative, expressed |
| *LOC_Os04g02120.1* | expressed protein |
| *LOC_Os04g02880.1* | expressed protein |
| *LOC_Os04g06700.1* | expressed protein |
| *LOC_Os04g03450.1* | retrotransposon protein, putative, unclassified, expressed |
| *LOC_Os04g07600.1* | AGAP002737-PA, putative, expressed |
| *LOC_Os04g01520.1* | expressed protein |
| **AQCI008** (33 genes) | |
| *LOC_Os03g15340.1* | plastocyanin-like domain containing protein, putative, expressed |
| *LOC_Os03g12879.1* | expressed protein |
| *LOC_Os03g14060.1* | retrotransposon protein, putative, Ty1-copia subclass, expressed |
| *LOC_Os03g12950.1* | AP2-like ethylene-responsive transcription factor AINTEGUMENTA, putative, expressed |
| *LOC_Os03g17250.1* | ankyrin repeat-containing protein, putative, expressed |
| *LOC_Os03g12730.1* | receptor protein kinase CLAVATA1 precursor, putative, expressed |
| *LOC_Os03g17870.1* | metallothionein, putative, expressed |
| *LOC_Os03g14910.1* | expressed protein |
| *LOC_Os03g14915.1* | expressed protein |
| *LOC_Os03g18550.1* | mitochondrial carrier protein, putative, expressed |
| *LOC_Os03g12670.1* | expressed protein |
| *LOC_Os03g14110.1* | expressed protein |
| *LOC_Os03g16230.2* | oxidoreductase, short chain dehydrogenase/reductase family protein, putative, expressed |
| *LOC_Os03g18560.1* | DUF538 domain containing protein, putative, expressed |
| *LOC_Os03g18150.1* | protein phosphatase 2C, putative, expressed |
| *LOC_Os03g18060.1* | transposon protein, putative, unclassified, expressed |
| *LOC_Os03g14642.1* | LTPL107 - Protease inhibitor/seed storage/LTP family protein precursor, expressed |
| *LOC_Os03g14140.1* | POEI16 - Pollen Ole e I allergen and extensin family protein precursor, expressed |
| *LOC_Os03g15320.1* | glyoxal oxidase-related, putative, expressed |
| *LOC_Os03g16790.1* | DHHC zinc finger domain containing protein, expressed |
| *LOC_Os03g13030.1* | lecithin cholesterol acyltransferase, putative, expressed |
| *LOC_Os03g17260.1* | expressed protein |
| *LOC_Os03g14840.1* | AGC_PVPK_like_kin82y.8 - ACG kinases include homologs to PKA, PKG and PKC, expressed |
| *LOC_Os03g12760.1* | helix-loop-helix DNA-binding domain containing protein, expressed |
| *LOC_Os03g13390.2* | oxidoreductase, aldo/keto reductase family protein, putative, expressed |
| *LOC_Os03g13050.1* | E2F-related protein, putative, expressed |
| *LOC_Os03g16050.1* | fructose-1,6-bisphosphatase, putative, expressed |
| *LOC_Os03g13140.1* | non-symbiotic hemoglobin 2, putative, expressed |
| *LOC_Os03g17070.1* | ATP synthase B chain, chloroplast precursor, putative, expressed |
| *LOC_Os03g15870.1* | ribosomal protein L4, putative, expressed |
| *LOC_Os03g14400.1* | cytochrome P450, putative, expressed |
| *LOC_Os03g17580.1* | ribosomal protein L10, putative, expressed |
| *LOC_Os03g16900.1* | rab GDP dissociation inhibitor alpha, putative, expressed |
| **AQCI009** (21 genes) | |
| *LOC_Os06g10930.1* | xyloglucan fucosyltransferase, putative, expressed |
| *LOC_Os06g08640.1* | transferase family protein, putative, expressed |
| *LOC_Os06g09310.1* | zinc finger, C3HC4 type domain containing protein, expressed |
| *LOC_Os06g07220.1* | LTPL128 - Protease inhibitor/seed storage/LTP family protein precursor, expressed |
| *LOC_Os06g10300.1* | OsFBL29 - F-box domain and LRR containing protein, expressed |
| *LOC_Os06g09220.1* | cytochrome P450 72A1, putative, expressed |
| *LOC_Os06g08710.1* | receptor-like protein kinase precursor, putative, expressed |
| *LOC_Os06g11150.1* | DUF1645 domain containing protein, putative, expressed |
| *LOC_Os06g07760.1* | sulfiredoxin-1, putative, expressed |
| *LOC_Os06g10820.1* | helix-loop-helix DNA-binding domain containing protein, expressed |
| *LOC_Os06g05250.2* | GTP-binding protein GUF1, putative, expressed |
| *LOC_Os06g08580.1* | transferase family protein, putative, expressed |
| *LOC_Os06g07040.1* | OsIAA20 - Auxin-responsive Aux/IAA gene family member, expressed |
| *LOC_Os06g09600.1* | expressed protein |
| *LOC_Os06g10590.1* | expressed protein |
| *LOC_Os06g07790.1* | retrotransposon protein, putative, unclassified, expressed |
| *LOC_Os06g07250.1* | jacalin-like lectin domain containing protein, expressed |
| *LOC_Os06g09610.1* | peroxiredoxin, putative, expressed |
| *LOC_Os06g10830.1* | retrotransposon protein, putative, unclassified, expressed |
| *LOC_Os06g04150.1* | magnesium-protoporphyrin O-methyltransferase, putative, expressed |
| *LOC_Os06g10870.1* | retrotransposon protein, putative, unclassified, expressed |
| **AQCI011** (29 genes) | |
| *LOC_Os04g41750.1* | expressed protein |
| *LOC_Os04g42570.1* | AP2/EREBP transcription factor BABY BOOM, putative, expressed |
| *LOC_Os04g45240.1* | expressed protein |
| *LOC_Os04g45890.1* | retrotransposon protein, putative, unclassified, expressed |
| *LOC_Os04g46220.1* | ethylene-responsive transcription factor, putative, expressed |
| *LOC_Os04g45330.1* | YABBY domain containing protein, putative, expressed |
| *LOC_Os04g46980.1* | cis-zeatin O-glucosyltransferase, putative, expressed |
| *LOC_Os04g46630.1* | expansin precursor, putative, expressed |
| *LOC_Os04g44950.1* | short-chain dehydrogenase/reductase, putative, expressed |
| *LOC_Os04g43840.1* | possible lysine decarboxylase domain containing protein, expressed |
| *LOC_Os04g44924.1* | short-chain dehydrogenase/reductase, putative, expressed |
| *LOC_Os04g44180.1* | expressed protein |
| *LOC_Os04g41410.1* | expressed protein |
| *LOC_Os04g45810.1* | homeobox associated leucine zipper, putative, expressed |
| *LOC_Os04g44900.1* | lectin-like receptor kinase, putative, expressed |
| *LOC_Os04g43360.1* | Os4bglu14 - monolignol beta-glucoside homologue without catalytic acid/base, expressed |
| *LOC_Os04g42830.1* | transferase family protein, putative, expressed |
| *LOC_Os04g46079.1* | ELMO/CED-12 family protein, putative, expressed |
| *LOC_Os04g45340.1* | expressed protein |
| *LOC_Os04g45590.1* | glyoxalase family protein, putative, expressed |
| *LOC_Os04g46880.1* | transporter, major facilitator family, putative, expressed |
| *LOC_Os04g45090.2* | cytochrome b561, putative, expressed |
| *LOC_Os04g45520.1* | integral membrane protein, putative, expressed |
| *LOC_Os04g44150.1* | gibberellin 2-beta-dioxygenase 7, putative, expressed |
| *LOC_Os04g46990.1* | cis-zeatin O-glucosyltransferase, putative, expressed |
| *LOC_Os04g45490.1* | elongation factor, putative, expressed |
| *LOC_Os04g43420.1* | PTAC5, putative, expressed |
| *LOC_Os04g41960.1* | NADP-dependent oxidoreductase, putative, expressed |
| *LOC_Os04g41340.1* | 4-nitrophenylphosphatase, putative, expressed |
| **AQCI012** (14 genes) | |
| *LOC_Os12g42200.1* | ATCHX, putative, expressed |
| *LOC_Os12g44110.2* | ligA, putative, expressed |
| *LOC_Os12g43660.1* | receptor-like protein kinase HAIKU2 precursor, putative, expressed |
| *LOC_Os12g42220.1* | expressed protein |
| *LOC_Os12g43640.1* | receptor-like protein kinase HAIKU2 precursor, putative, expressed |
| *LOC_Os12g43380.1* | thaumatin, putative, expressed |
| *LOC_Os12g42020.1* | AGC_PVPK_like_kin82y.20 - ACG kinases include homologs to PKA, PKG and PKC, expressed |
| *LOC_Os12g44210.1* | ATPase, AAA family domain containing protein, expressed |
| *LOC_Os12g42010.1* | lipase class 3 family protein, putative, expressed |
| *LOC_Os12g44050.1* | purple acid phosphatase precursor, putative, expressed |
| *LOC_Os12g43410.1* | thaumatin, putative, expressed |
| *LOC_Os12g42044.3* | OsWAK127b - OsWAK short gene, expressed |
| *LOC_Os12g41510.1* | cysteine-rich receptor-like protein kinase 31 precursor, putative, expressed |
| *LOC_Os12g44020.1* | Ser/Thr protein phosphatase family protein, putative, expressed |
| **AQCI013** (31 genes) | |
| *LOC_Os12g12600.1* | dirigent, putative, expressed |
| *LOC_Os12g12290.1* | exostosin family domain containing protein, expressed |
| *LOC_Os12g17430.1* | NBS-LRR disease resistance protein, putative, expressed |
| *LOC_Os12g17160.1* | flavonol sulfotransferase, putative, expressed |
| *LOC_Os12g16520.1* | wall-associated receptor kinase 3 precursor, putative, expressed |
| *LOC_Os12g16340.1* | retrotransposon protein, putative, unclassified, expressed |
| *LOC_Os12g15680.1* | laccase precursor protein, putative, expressed |
| *LOC_Os12g18080.1* | retrotransposon protein, putative, unclassified, expressed |
| *LOC_Os12g16540.1* | OsWAK124 - OsWAK receptor-like protein OsWAK-RLP, expressed |
| *LOC_Os12g16490.1* | transposon protein, putative, unclassified, expressed |
| *LOC_Os12g17410.1* | NB-ARC domain containing protein, expressed |
| *LOC_Os12g09700.1* | Jacalin-like lectin domain containing protein, putative, expressed |
| *LOC_Os12g12990.1* | transposon protein, putative, CACTA, En/Spm sub-class, expressed |
| *LOC_Os12g21720.1* | retrotransposon protein, putative, unclassified, expressed |
| *LOC_Os12g14330.1* | disease resistance protein RPM1, putative, expressed |
| *LOC_Os12g16480.1* | expressed protein |
| *LOC_Os12g12720.1* | jasmonate-induced protein, putative, expressed |
| *LOC_Os12g13730.1* | pleiotropic drug resistance protein 2, putative, expressed |
| *LOC_Os12g10330.1* | NB-ARC domain containing protein, expressed |
| *LOC_Os12g10340.1* | NBS-LRR type resistance protein, putative, expressed |
| *LOC_Os12g10410.1* | NB-ARC/LRR disease resistance protein, putative, expressed |
| *LOC_Os12g19470.2* | ribulose bisphosphate carboxylase small chain, chloroplast precursor, putative, expressed |
| *LOC_Os12g19381.1* | ribulose bisphosphate carboxylase small chain, chloroplast precursor, putative, expressed |
| *LOC_Os12g12120.1* | verticillium wilt disease resistance protein precursor, putative, expressed |
| *LOC_Os12g15314.1* | staphylococcal nuclease homologue, putative, expressed |
| *LOC_Os12g14580.1* | tetraspanin family protein, putative, expressed |
| *LOC_Os12g13470.1* | hypothetical protein |
| *LOC_Os12g14699.1* | protein kinase domain containing protein, expressed |
| *LOC_Os12g12130.1* | verticillium wilt disease resistance protein, putative, expressed |
| *LOC_Os12g17600.1* | ribulose bisphosphate carboxylase small chain, chloroplast precursor, putative, expressed |
| *LOC_Os12g16080.1* | expressed protein |
