# Supplementary material for: Transcriptome analysis of phosphorus stress responsiveness in the seedlings of Dongxiang wild rice (Oryza rufipogon Griff.)
Source: Biol Res. 2018 Mar 15;51:7. doi: 10.1186/s40659-018-0155-x (PMC5853122; doi:10.1186/s40659-018-0155-x)
Supplement: Supplementary file 16 — Additional file 16: Table S15. Co-localized DEGs within the qFWS-4 interval. [file 40659_2018_155_MOESM16_ESM.docx]

| **Table S15** Co-localized DEGs within the *qFWS-4* interval. | |
| --- | --- |
| Gene ID | Function |
| *LOC_Os11g05640.1* | bZIP transcription factor domain containing protein, expressed |
| *LOC_Os11g03440.1* | myb-like DNA-binding domain containing protein, putative, expressed |
| *LOC_Os11g04860.1* | anthocyanin 5-O-glucosyltransferase, putative, expressed |
| *LOC_Os11g44380.1* | expressed protein |
| *LOC_Os11g44810.2* | auxin-repressed protein, putative, expressed |
| *LOC_Os11g06020.1* | homeobox domain containing protein, expressed |
| *LOC_Os11g44310.1* | calmodulin binding protein, putative, expressed |
| *LOC_Os11g02240.1* | CAMK_KIN1/SNF1/Nim1_like.4 - CAMK includes calcium/calmodulin depedent protein kinases, expressed |
| *LOC_Os11g05740.1* | B3 DNA binding domain containing protein, expressed |
| *LOC_Os11g04894.1* | expressed protein |
| *LOC_Os11g04480.1* | peflin, putative, expressed |
| *LOC_Os11g04060.1* | major facilitator superfamily antiporter, putative, expressed |
| *LOC_Os11g03640.1* | expressed protein |
