# Supplementary material for: Transcriptome analysis of phosphorus stress responsiveness in the seedlings of Dongxiang wild rice (Oryza rufipogon Griff.)
Source: Biol Res. 2018 Mar 15;51:7. doi: 10.1186/s40659-018-0155-x (PMC5853122; doi:10.1186/s40659-018-0155-x)
Supplement: Supplementary file 17 — Additional file 17: Table S16. Co-localized DEGs within the qFWR-4 interval. [file 40659_2018_155_MOESM17_ESM.docx]

| **Table S16** Co-localized DEGs within the *qFWR-4* interval. | |
| --- | --- |
| Gene ID | Function |
| *LOC_Os11g04020.1* | major facilitator superfamily antiporter, putative, expressed |
| *LOC_Os11g05390.1* | transporter, major facilitator family, putative, expressed |
| *LOC_Os11g04550.1* | sulfotransferase domain containing protein, expressed |
| *LOC_Os11g03940.1* | retrotransposon protein, putative, Ty1-copia subclass, expressed |
| *LOC_Os11g04290.1* | cytochrome P450, putative, expressed |
| *LOC_Os11g05260.1* | phosphoglycerate mutase, putative, expressed |
| *LOC_Os11g03290.1* | nucleoside-triphosphatase, putative, expressed |
| *LOC_Os11g04490.1* | astaxanthin synthase KC28, putative, expressed |
| *LOC_Os11g05380.1* | cytochrome P450, putative, expressed |
| *LOC_Os11g05400.1* | Ser/Thr protein phosphatase family protein, putative, expressed |
| *LOC_Os11g04300.1* | retrotransposon protein, putative, unclassified, expressed |
